# C–C Coupling in sterically demanding porphyrin environments

**DOI:** 10.3762/bjoc.20.234

**Published:** 2024-11-04

**Authors:** Liam Cribbin, Brendan Twamley, Nicolae Buga, John E O’ Brien, Raphael Bühler, Roland A Fischer, Mathias O Senge

**Affiliations:** 1 School of Chemistry, Chair of Organic Chemistry, Trinity Biomedical Sciences Institute, 152-160 Pearse Street, Trinity College Dublin, The University of Dublin, Dublin, D02 R590, Ireland; 2 School of Chemistry, Trinity College Dublin, The University of Dublin, Dublin 2, Ireland; 3 TUM School of Natural Sciences, Technical University of Munich, Lichtenbergstr. 4, 85748 Garching, Germany; 4 Institute for Advanced Study (TUM-IAS), Technical University of Munich, Lichtenberg, Str. 2a, 85748 Garching, Germany

**Keywords:** C–C coupling, conformational analysis, nonplanar porphyrin, Pd-catalysis, porphyrin

## Abstract

Unlike their planar counterparts, classic synthetic protocols for C–C bond forming reactions on nonplanar porphyrins are underdeveloped. The development of C–C bond forming reactions on nonplanar porphyrins is critical in advancing this field of study for more complex porphyrin architectures, which could be used in supramolecular assemblies, catalysis, or sensing. In this work a library of arm-extended dodecasubstituted porphyrins was synthesized through the optimization of the classic Suzuki–Miyaura coupling of peripheral haloaryl substituents with a range of boronic acids. We report on palladium-catalyzed coupling attempts on the *ortho*-, *meta*-, and *para*-meso-phenyl position of sterically demanding dodecasubstituted saddle-shaped porphyrins. While *para-* and *meta-*substitutions could be achieved, *ortho*-functionalization in these systems remains elusive. Furthermore, borylation of a dodecasubstituted porphyrin’s meso-phenyl position was explored and a subsequent C–C coupling showed the polarity of the reaction can be reversed resulting in higher yields. X-ray analysis of the target compounds revealed the formation of supramolecular assemblies, capable of accommodating substrates in their void.

## Introduction

**Figure 1:**
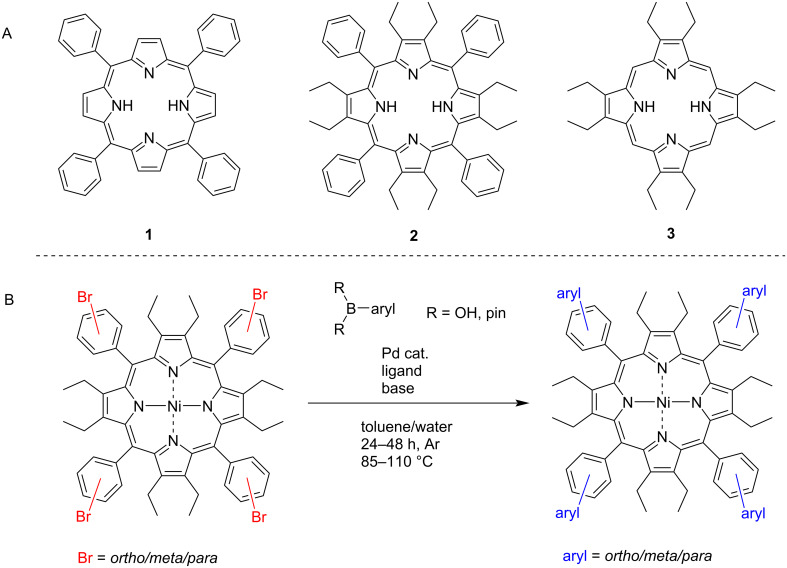
(A) Structures of tetrasubstituted 5,10,15,20-tetraphenylporphyrin (TPP, **1**), dodecasubstituted 2,3,7,8,12,13,17,18-octaethyl-5,10,15,20-tetraphenylporphyrin (OETPP, **2**), and octasubstituted 2,3,7,8,12,13,17,18-octaethylporphyrin (OEP, **3**). (B) Aim of this work, the arm extension of the meso-phenyl position of dodecasubstituted porphyrins.

Porphyrins are tetrapyrrolic macrocycles that perform essential processes in nature, such as oxygen transport in hemoglobin and photosynthesis [1]. Porphyrins are often described as planar 18π aromatic macrocycles; however, molecular structure analysis frequently reveals nonplanar ring distortion [2–3]. In fact, porphyrins with nonplanar ring distortions are vital for many natural processes to occur, e.g., nonplanarity can alter oxygen affinity of the metal iron core [4–5]. Nonplanar porphyrins offer a marked difference in chemical and physical properties when compared to their planar compatriots [6], with relatively smaller HOMO–LUMO gaps resulting in an observed bathochromic shift in the UV–vis absorption spectrum [7]. The phenomenon of nonplanarity results from the porphyrin ring deforming from the mean porphyrin plane either by steric repulsion in the core of the macrocycle or by bulky substituents at the porphyrin periphery [3]. This affords four principle distortion modes, saddle, dome, ruffle or wave [8], which can be quantified by the normal-coordinate structural decomposition (NSD) method developed by Shelnutt and co-workers [9] and further implemented and visualized by us [8,10]. Of the four main quantifiable distortion modes, saddle-shaped porphyrins can be afforded by *peri*-interactions between β-substituents and the meso-substituents [3,11], or alternatively by core protonation, whereby all four-core nitrogen atoms are protonated to produce the diacid [12–13]; these diacids can tilt the pyrrole rings 20–40° [14] from the mean-porphyrin plane. Norvaiša et al. showed that a saddle-shaped porphyrin as a dodecasubstituted diacid can bind anions via two independent faces and trap anions such as pyrophosphate [15]. Saddle-shaped porphyrins have also been exploited by researchers for the use in organocatalysis as bifunctional system [16–17]. Dodecasubstitution of porphyrin, as seen in Figure 1, often results in saddle-shaped distortion; however, ruffled [18] and almost planar [19] dodecasubstituted porphyrins have been reported.

Despite the increasing interest in the chemical and physical properties of nonplanar porphyrins only limited synthetic methods are available for the functionalization of these macrocycles [6]. An attractive approach to accomplish further substitution directly on the meso- or a meso-phenyl *ortho/meta/para* positions of a porphyrin, is the introduction of C–C bond forming chemistry. This is typically achieved using palladium and/or another transition-metal catalyst [20]. Sonagashira [21], Suzuki–Miyaura [22], Heck [23], Stille [24–25], Negishi [26], and Kumada [27] coupling reactions, as well as modern iridium and rhodium-based coupling techniques [28], are just some examples of the C–C bond formations that have been implemented to achieve complex substitution patterns and functional arrangements on porphyrins.

Of these named coupling reactions, Suzuki–Miyaura couplings are known to be a robust tool when functionalizing porphyrins [29–30]. Many complex porphyrinoid architectures have been synthesized in this manner, from functional porphyrin arrays [31–33] to sterically challenging meso-substituted aryl bis-pocket porphyrins [34] and tetrabromoanthracenyl porphyrins [35]. In general, the halogen atom needed for the Suzuki coupling reaction resides on the porphyrin; however, Suzuki–Miyaura reactivity has also been shown to be reversed whereby the synthesis of borolanylporphyrins leads to a different approach to reactivity [36]. Borolanylporphyrins can be synthesized by Miyaura-borylation of the halogenated porphyrin [24,37]. There are also reported instances of borolanylporphyrins being synthesized under condensation conditions [36,38]. Despite the many synthetic advancements for the decoration of porphyrins, many of these strategies are utilized only with planar porphyrins. Apart from the arylation of the β-position of 2,3,5,7,8,10,12,13,15,17,18,20-dodecaarylporphyrins, developed by Smith and co-workers [39] few reports on synthetic techniques for dodecasubstituted nonplanar porphyrins can be found in literature. In light of the promise of appropriately designed nonplanar porphyrins as receptors and catalysts we report here on our efforts to use the Suzuki–Miyaura reaction for the modification of the *o,m,p*-phenyl positions in 5,10,15,20-tetraryl-2,3,7,8,12,13,17,18-octaethylporphyrins.

## Results and Discussion

### Investigation of the Suzuki coupling reaction at the meso-phenyl position of dodecasubstituted porphyrins

#### Synthesis of porphyrin precursors

**Scheme 1:**
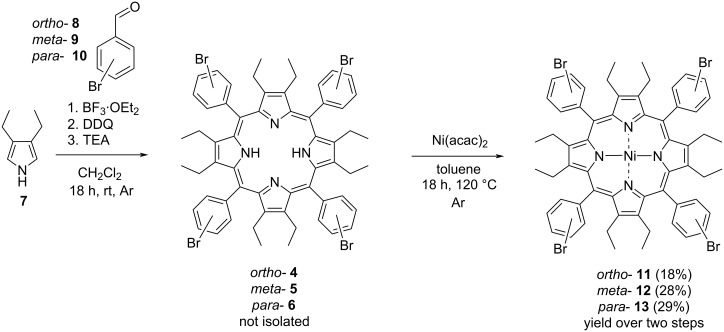
Reaction scheme for the synthesis of OET-xBrPPs and subsequent Ni(II) metalation.

To investigate the Suzuki coupling at the *ortho*-, *meta*- and *para*-position of a dodecasubstituted saddle-shaped porphyrin, first the precursor porphyrins **11**, **12**, and **13** had to be synthesized (Scheme 1). The synthetic route to achieve OET-xBrPPs (2,3,7,8,12,13,17,18-octaethyl-5,10,15,20-tetra(x-bromo)phenylporphyrin, where x = *ortho/meta*/*para*) pyrrole **7** was synthesized through literature procedures [40–41]. Pyrrole **7** was then subjected to condensation with aldehydes **8**, **9**, and **10** under Lindsey conditions [42] utilizing BF_3_·OEt_2_ and DDQ [43] to achieve porphyrins **4**, **5**, and **6**, which were not isolated and instead reacted immediately.

Ni(II)porphyrins **11**, **12**, and **13** were prepared by reacting porphyrins **4**, **5**, and **6** in toluene for 18 hours using Ni(II)(acac)_2_ under an inert atmosphere [43] attaining a 18%, 28%, and 29% yield for porphyrins **11**, **12**, and **13**, respectively, over two steps. Porphyrins **6** and **13** had previously been described in literature [43].

#### Coupling at the meso-*para-*phenyl position

**Figure 2:**
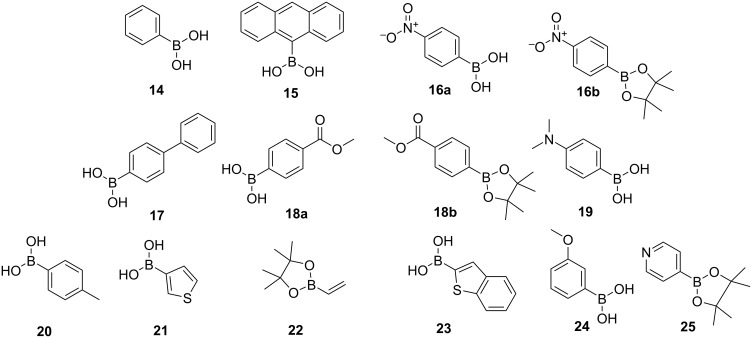
Substrates used for the investigations for the Suzuki–Miyaura coupling reactions.

**Scheme 2:**
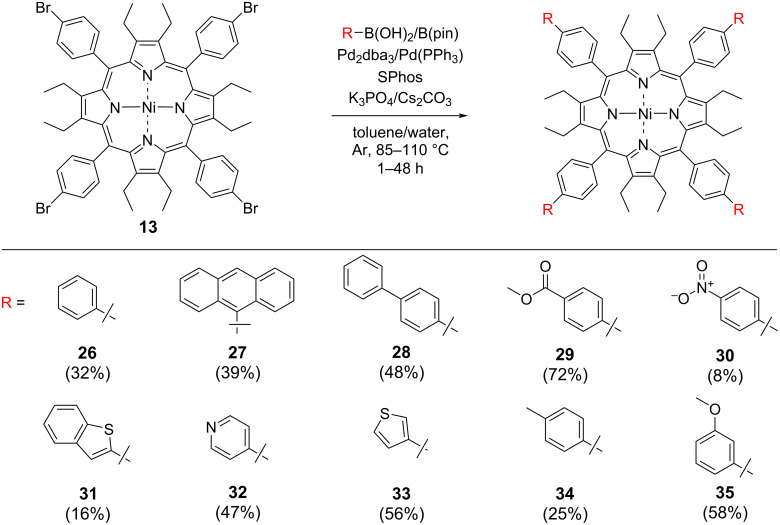
Scope of arm-extended dodecasubstituted porphyrins synthesized via modification of the meso-*para*-phenyl position of porphyrin **13**.

The exploration of aryl substitution of OET-xBrPPs using the Suzuki coupling began with investigating first the Suzuki reaction compatibility of boronic acid **14** with porphyrin **13**. Porphyrin **13** and phenylboronic acid (**14)** were subjected to coupling at 85 °C for 48 hours using Pd_2_dba_3_/SPhos as a catalyst/ligand giving porphyrin **26** in a 32% yield, based on a literature procedure [35]. With initial success in the synthesis porphyrin **26**, this Suzuki coupling reaction was performed on **13**, for a range of boronic acids/esters as shown in Figure 2 and Scheme 2. Boronic acids/esters were chosen based on their electronic properties (activating/deactivating) as well as their steric bulk (e.g., 9-anthracenylboronic acid (**15**)). Table 1 lists all attempts at the meso-*para*-phenyl position.

**Table 1:** Optimization table for the Suzuki–Miyaura coupling reactions with porphyrin **13**.

Entry	Catalyst/ligand SPhos (1 equiv)	Cat. mol % per C–Br	Base (24 equiv)	Temperature	Time	Boronic acid/ester (3 equiv per C–Br)	Yield % (porphyrin)

1	Pd_2_dba_3_/Sphos	6.25%	K_3_PO_4_	85 °C	48 h	**14**	32% (**26**)
2	Pd_2_dba_3_/Sphos	6.25%	K_3_PO_4_	85 °C	48 h	**15**	trace
3	Pd_2_dba_3_/SPhos	6.25%	K_3_PO_4_	110 °C	48 h	**15**	39% (**27**)
4	Pd_2_dba_3_/Sphos	6.25%	K_3_PO_4_	85 °C	48 h	**16a**	0
5	Pd_2_dba_3_/Sphos	6.25%	Cs_2_CO_3_	85 °C	48 h	**16a**	0
6	Pd_2_dba_3_/SPhos	6.25%	Cs_2_CO_3_	85 °C	48 h	**16b**	8% (**30**)
7	Pd_2_dba_3_/SPhos	6.25%	K_3_PO_4_	110 °C	48 h	**17**	48% (**28**)
8	Pd_2_dba_3_/SPhos	12.5%	K_3_PO_4_	110 °C	48 h	**18a**	0
9	Pd_2_dba_3_/SPhos	6.25%	Cs_2_CO_3_	85 °C	48 h	**18b**	72% (**29**)
10	Pd_2_dba_3_/Sphos	6.25%	K_3_PO_4_	85 °C	48 h	**19**	0
11	Pd_2_dba_3_/SPhos	6.25%	K_3_PO_4_	110 °C	48 h	**19** ^a^	trace
12	Pd_2_dba_3_/SPhos	12.5%	K_3_PO_4_	110 °C	24 h	**20**	25% (**34**)
13	Pd_2_dba_3_/SPhos	6.25%	Cs_2_CO_3_	110 °C	24 h	**21**	56% (**33**)
14	Pd_2_dba_3_/SPhos	6.25%	K_3_PO_4_	110 °C	48 h	**23**	trace
15	Pd_2_dba_3_/SPhos	6.25%	Cs_2_CO_3_	85 °C	48 h	**23**	trace
16	Pd(PPh_3_)_4_	10%	Na_2_CO_3_	100 °C	1 h	**23** ^b^	0
17	Pd_2_dba_3_/SPhos	6.25%	Cs_2_CO_3_	85 °C	24 h	**23**	trace
18	Pd_2_dba_3_/SPhos	6.25%	Cs_2_CO_3_	110 °C	24 h	**23**	trace
19	Pd_2_dba_3_/SPhos	25%	Cs_2_CO_3_	110 °C	24 h	**23**	16% (**31**)
20	Pd_2_dba_3_/SPhos	12.5%	Cs_2_CO_3_	110 °C	24 h	**24**	58% (**35**)
21	Pd_2_dba_3_/SPhos	6.25%	Cs_2_CO_3_	85 °C	24 h	**25**	47% (**32**)

**^a^**5 equiv of boronic acid used in this reaction per C–Br. ^b^Microwave irradiation instead of conventional heating was used.

When attempting the synthesis of tetra(*p-*phenylanthracene)porphyrin (**27**) the conditions used before (Table 1, entry 1) resulted only in trace amounts of porphyrin **27** (Table 1, entry 2).

The reaction temperature was increased to 110 °C, affording the desired porphyrin **27** in a 39% yield (Table 1, entry 3). A temperature of 110 °C was also used for the synthesis of terphenylporphyrin **28** using boronic acid **17**, affording terphenylporphyrin **28** in 48% yield (Table 1, entry 7).

Boronic acids with heteroatoms and activating/deactivating electronic properties were investigated next. Attempts to introduce electron-withdrawing groups at the *para*-position with substrate boronic acid **16a** (Table 1, entries 4 and 5) yielded no tetracoupled product. Similarly, coupling with **18a** resulted in most of the starting material porphyrin **13** being left unreacted. On switching the substrate from boronic acid to the boronic acid ester and opting for the weaker base Cs_2_CO_3_ instead of K_3_PO_4_, a significant difference in reactivity was observed with a 72% yield accomplished in the synthesis of porphyrin **29** (Table 1, entry 9), bearing a methoxycarbonyl electron-withdrawing group utilizing boronic acid pinacol ester **18b**. Following on from these results porphyrin **30** was synthesized in an 8% yield, when switching to weaker base Cs_2_CO_3_ using pinacol ester **16b** (Table 1, entry 6). Switching the base to a weaker one, may have slowed down the protodeboronation process, as substrates with electron-withdrawing groups are postulated to increase the Lewis acidity of the boronic acid, which may allow an increased incidence of protodeboronation to occur. It is also known that aryl–B(Pin) complexes have a greater stability than boronic acids and other employed esters as the four methyl groups protect the boron center from attack of water [44–45], preventing protodeboronation from the hydrolysis route. However, protodeboronation can be complex when it comes to p*K*_a_ considerations, for example, 3,5-dinitrophenylboronic acid has a marginally lower p*K*_a_ than pentafluorophenyl boronic acid [46]; however, it undergoes protodeboronation, several orders of magnitude slower [47].

The synthesis of porphyrin **31** with a benzothiophene moiety, proved challenging (Table 1, entries 14–19). Use of a microwave-assisted procedure [48], switching catalyst to Pd(PPh)_3_, and base to Na_2_CO_3_ (Table 1, entry 16) gave no product.

Ultimately, an increased catalyst loading of 25 mol % per C–Br bond gave the desired porphyrin in a 16% yield when using Cs_2_CO_3_ as base. The synthesis of other heterocycle-appended dodecasubstituted porphyrins, achieved porphyrins **32** and **33** in a 47% and 56% yield, respectively (Table 1, entries 13 and 21), using Cs_2_CO_3_ as the base. Electron-withdrawing sulfur-containing heterocyclic substrates **21** and **23** do not readily undergo protodeboronation even at high pH [44,47] making the yields of porphyrins **31** and **33** higher than expected considering the electronic similarities between substrates 4-nitrophenylboronic acid and 3-thiaphenylboronic acid (**16a** and **21**) and the yields obtained when coupling. The weakly electron-withdrawing boronic acid **24** when coupled with porphyrin **13**, resulted in porphyrin **35** in a 58% yield (Table 1, entry 20). Reactivity with the electron-donating 4-methylphenylboronic acid (**34**) was established using K_3_PO_4_ at 110 °C (Table 1, entry 12). No product was obtained in the coupling of electron-donating (4-(dimethylamino)phenyl)boronic acid (**19**), even upon increasing the number of equivalents of boronic acid (Table 1, entries 10 and 11).

#### Coupling at the meso*-meta*-phenyl position

**Scheme 3:**
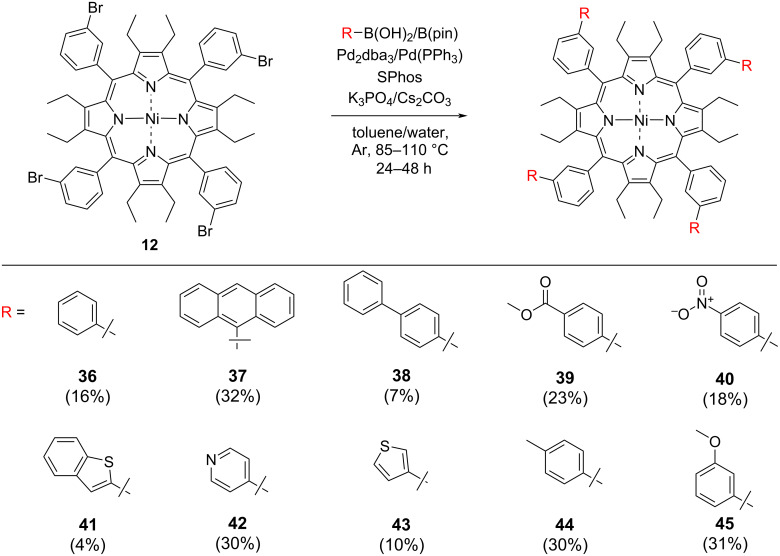
Scope of arm-extended dodecasubstituted porphyrins synthesized via reaction at the meso-*meta-*phenyl position of porphyrin **12**.

Optimization of conditions for OET-*meta-*BrPPs **12** (Scheme 3) were investigated next. Table 2 summarizes the reaction conditions used to synthesize a library of OET-*meta*-arylPPs as shown in Scheme 3. As a starting point initial conditions used in the synthesis for porphyrin **26** were used (Table 1, entry 1). This gave biphenylporphyrin **36** in a 16% yield (Table 2, entry 1). The lower yield is expected due to the increased steric demand at the *meta*-positions. Coupling of sterically bulky 9-anthracenylboronic acid (**15**) and porphyrin **12** gave no conversion when the base was switched from K_3_PO_4_ to Cs_2_CO_3_ (Table 2, entry 2). K_3_PO_4_ was reimplemented in the reaction and trace reactivity was observed by TLC (Table 2, entry 3). Next, the catalyst loading was increased to 12.5 mol % (Table 2, entry 4). Formation of palladium black was observed but product formation was also indicated by TLC and ^1^H NMR. For a final attempt at establishing reactivity with boronic acid **15** the temperature was increased to 110 °C and gave the desired anthracenylporphyrin **37** in a 32% yield. In the case of boronic acids with larger π-systems, e.g., **15**, K_3_PO_4_ was required to achieve the tetra-coupled product. This trend is consistent in reactivity observed with porphyrins **12** and **13.** Similarly, no terphenyl product was formed in the coupling reaction between **12** and **17** (Table 2, entry 6) when using Cs_2_CO_3_. Similar to the reactivity observed with 9-anthracenylboronic acid (**15**), no conversion to the desired product was established. Increasing the temperature and catalyst loading (Table 2, entry 5) gave the terphenylporphyrin **38** in a 7% yield (Table 2, entry 7).

**Table 2:** Optimization table for the Suzuki-coupling reaction on porphyrin **12**.

Entry	Catalyst/ligand SPhos (1 equiv)	Cat. mol % per C–Br	Base (24 equiv)	Temp.	Time	Boronic acid/ester (3 equiv per C–Br)	Yield % (porphyrin)

1	Pd_2_dba_3_/SPhos	6.25%	K_3_PO_4_	85 °C	48 h	**14**	16% (**36**)
2	Pd_2_dba_3_/SPhos	6.25%	Cs_2_CO_3_	85 °C	48 h	**15**	0
3	Pd_2_dba_3_/SPhos	6.25%	K_3_PO_4_	85 °C	48 h	**15**	trace
4	Pd_2_dba_3_/SPhos	12.5%	K_3_PO_4_	85 °C	48 h	**15**	trace
5	Pd_2_dba_3_/SPhos	12.5%	K_3_PO_4_	110 °C	48 h	**15**	32% (**37**)
6	Pd_2_dba_3_/SPhos	6.25%	Cs_2_CO_3_	85 °C	48 h	**17**	0
7	Pd_2_dba_3_/SPhos	12.5%	K_3_PO_4_	110 °C	24 h	**17**	7% (**38**)
8	Pd_2_dba_3_/SPhos	6.25%	Cs_2_CO_3_	85 °C	48 h	**18b**	0
9	Pd_2_dba_3_/SPhos	12.5%	Cs_2_CO_3_	85 °C	48 h	**18b**	23% (**39**)
10	Pd_2_dba_3_/SPhos	12.5%	Cs_2_CO_3_	85 °C	24 h	**16b**	4% (**40**)
11	Pd_2_dba_3_/SPhos	6.25%	Cs_2_CO_3_	85 °C	48 h	**20**	0
12	Pd_2_dba_3_/SPhos	12.5%	K_3_PO_4_	110 °C	24 h	**20**	30% (**44**)
13	Pd_2_dba_3_/SPhos	12.5%	Cs_2_CO_3_	110 °C	24 h	**21**	10% (**43**)
14	Pd_2_dba_3_/SPhos	25%	Cs_2_CO_3_	110 °C	24 h	**23**	4% (**41**)
15	Pd_2_dba_3_/SPhos	12.5%	Cs_2_CO_3_	110 °C	24 h	**24**	31% (**45**)
16	Pd_2_dba_3_/SPhos	12.5%	Cs_2_CO_3_	110 °C	24 h	**25**	30% (**42**)
17	Pd_2_dba_3_/SPhos	12.5%	NaOAc	110 °C	24 h	**16a**	18% (**40**)

The use of Cs_2_CO_3_ is still required for boronic acids bearing electron-withdrawing functionalities at the *meta*-phenyl position (Table 2, entries 9 and 10). However, an increase in catalyst loading to 12.5% was required per C–Br bond when coupling at the meso-*meta*-phenyl position in **12** compared to the corresponding *para*- position in **13** (Table 2**,** entry 8). Porphyrins **39** and initially **40** were synthesized in a 23% and 4% yield, respectively. This is significantly lower than for the corresponding *para-*products. Porphyrin **45** was synthesized in a 31% yield (Table 2, entry 15), with a 3-methoxy electron-withdrawing group, again a lower yield compared to the *para*-analogue porphyrin **35**. Use of the electron-donating *p*-tolylboronic acid (**20**), resulted in a 30% yield (Table 2, entry 12) again requiring an increase of catalyst loading and a change of base to K_3_PO_4_. Heterocyclic boronic acids/esters were again investigated for coupling reactivity with a consistent trend of lower yields experienced for porphyrins **41**, **42**, and **43** of 4%, 30%, and 10%, respectively. Overall, a general trend of decreased yield moving from the *para-* to *meta*-position was observed, also a general increased catalyst concentration was needed for reactivity to occur at the *meta*-position.

Lastly, decreasing the basicity of the solution further by switching to sodium acetate as a basic source increased the yield of porphyrin **40** from 4% with Cs_2_CO_3_ to 18% using sodium acetate (Table 2, entry 17). This indicates that a further decrease in basicity may improve yields.

#### Coupling at the meso-*ortho-*phenyl position

**Scheme 4:**
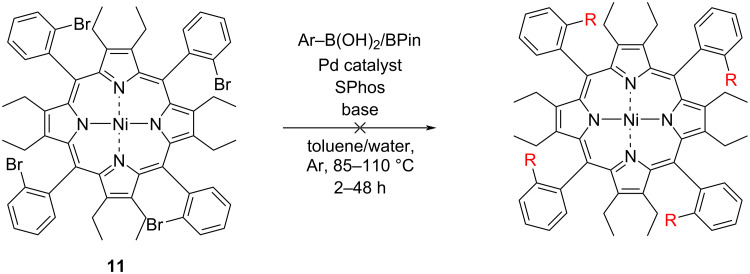
Attempts of arm-extension of dodecasubstituted porphyrins at the meso-*ortho*-phenyl position.

Unlike the success achieved in the synthesis of OET-*meta/para*-arylPPs, the *ortho*-position on the meso-phenyl proved much more intractable (Scheme 4). Table 3 provides a summary of the attempts made to achieve Suzuki-coupling reactivity in OET-*ortho*-BrPPs.

**Table 3:** Optimization table for the Suzuki-coupling reaction on porphyrin **11**.

Entry	Catalyst/ligand	Cat. mol % per C–Br	Base (24 equiv)	Temp. (ºC)	Time	Boronic acid/ester (3 equiv per C–Br)	Product detected by HRMS

1	Pd_2_dba_3_/SPhos	6.25%	K_3_PO_4_	110 °C	48 h	**14**	no
2	Pd_2_dba_3_/SPhos	6.25%	K_3_PO_4_	130 ºC	48 h	**14**	no
3	Pd_2_dba_3_/SPhos	12.5%	Cs_2_CO_3_	110 °C	48 h	**14**	no
4	Pd_2_dba_3_/Xantphos	12.5%	Cs_2_CO_3_	110 °C	48 h	**14**	no
5	Pd_2_dba_3_/Rac BINAP	12.5%	Cs_2_CO_3_	110 °C	48 h	**14**	no
6	Pd_2_dba_3_/SPhos	100%	Cs_2_CO_3_	85 °C	7 days	**14** **^a^**	no
7	Pd(dppf)Cl_2_	2%	Cs_2_CO_3_	100 °C	20 h	**20** **^b^**	no
8	Pd(PPh_3_)_4_	5%	Cs_2_CO_3_	100 °C	20 h	**20** **^b^**	no
9	Pd(PPh_3_)_4_	12.5%	Na_2_CO_3_	120 °C	2 h	**21** **^c^**	no
10	Pd(PPh_3_)_4_	12.5%	Cs_2_CO_3_	100 °C	48 h	**21**	no
11	Pd_2_dba_3_/SPhos	12.5%	Cs_2_CO_3_	85 °C	48 h	**22**	no
12	Pd_2_dba_3_/SPhos	6.25%	Cs_2_CO_3_	85 °C	24 h	**25**	no

^a^12 equiv of boronic acid used in this reaction per C–Br. ^b^Alternative procedure for Suzuki–Miyaura coupling [34]. ^c^Microwave irradiation instead of conventional heating was used [48].

Increasing the reaction temperature compared to the 85 °C used in the synthesis of the *para*-equivalent **26** gave no conversion and was accompanied by the formation of palladium black [49]. No reactivity was observed by either TLC or mass spectrometry when switching back to Pd_2_(dba)_3_ with three different ligands SPhos, XantPhos, and *Rac*-Binap (Table 3, entries 3–5). Increasing the time of reaction, catalyst loading, and equivalents of boronic acid significantly also resulted in no product formation (Table 3, entry 6).

Next, a change in the catalyst was investigated, based on a literature procedure which was developed by Johnstone and co-workers for the synthesis of meso-substituted aryl bis-pocket porphyrins [34]. Therein catalysts Pd(dppf)Cl_2_ and Pd(PPh_3_)_3_ were identified to be the most effective for accomplishing the Suzuki–Miyaura coupling at the *ortho*-position of the meso-phenyl position in sterically hindered planar porphyrins (Table 3, entries 7 and 8). The same success could not be replicated for OET-*o*-BrPPs with no reactivity being observed by TLC or by mass spectrometry. Likewise, a microwave-assisted coupling [48], resulted in no product formation (Table 3, entry 9). Thiophene-3-ylboronic acid (**21**) was also chosen for this reaction due to the smaller size compared to the phenyl- and *p*-tolylboronic acids **14** and **20**. Using **21** as the starting material and the procedure by Droege et al. [34] it was anticipated the less steric substrate size would possibly allow conversion to occur; however, no product formation was observed (Table 3, entries 9 and 10). 4-Pyridylboronic acid pinacol ester (**25**) was also attempted; however, no product was formed. Vinylboronic acid ester **22**, was also explored as a substrate, with multiple porphyrin products being observed by TLC and by ^1^H NMR. Desymmetrization of the porphyrin was also observed with the β-ethyl CH_3_ resonances splitting into two separate chemical environments; however, the identity of the product synthesized was not fully characterized. In future, if reactivity for OET-*o*-BrPPs were to be further explored a larger library of ligands whether biphenyl-based or other could be explored, or further changes in the pH of the solution. Enrichment to the αβαβ-atropisomer may also be favorable [50], as to alleviate the steric hindrance caused by the short distances (4.3–4.4 Å) between bromines in the α_2_β_2_-atropisomers (cf., Figure 5).

#### Borylation and further coupling of dodecasubstituted porphyrins

**Scheme 5:**
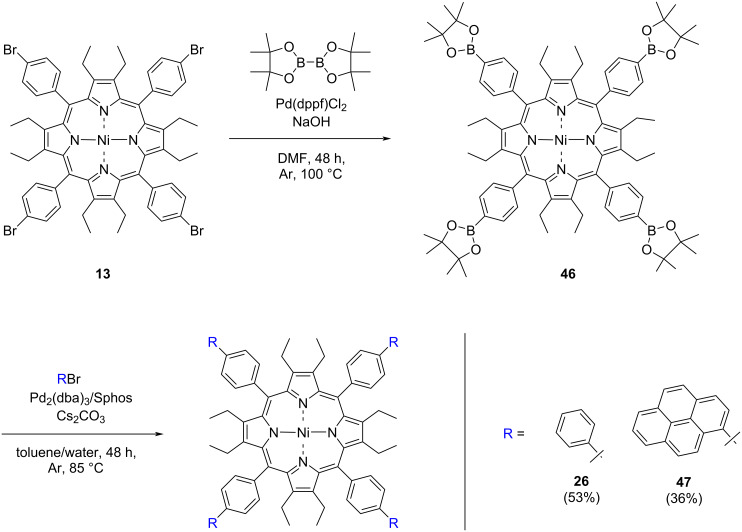
Borylation and subsequent Suzuki–Miyaura coupling of porphyrin **13**.

A Miyaura borylation was performed on porphyrin **13**, using bis(pinacolato)diboron (B_2_Pin_2_), adapting a procedure from the literature [51]. This procedure was further optimized (Table 4) by utilizing conditions in the synthesis of **29** (Table 1, entry 9). Next, a reversed polarity Suzuki reaction was performed on the borolanyl porphyrin **46** (Scheme 5). This reaction yielded porphyrin **26** in a 53% yield and tetrapyrenylporphyrin **47** in a 36% yield, respectively. Compared to the synthesis of **26** by Suzuki coupling of *para*-bromo-phenylporphyrin **13** a significant increase in yield was observed. Furthermore, pyrene was installed on the *para*-phenyl position, showing large aromatic systems can also be installed through this route. Failure of the similar anthracenylboronic acid **15** to react in the presence of Cs_2_CO_3_ at 85 °C (Table 2, entry 2) shows reversing the polarity of the reaction can induce reactivity, where not previously possible.

**Table 4:** Optimization of the borylation of porphyrin **13** to yield **46**.

Entry	Catalyst mol % per C–Br bond	Catalyst	Equiv of B_2_Pin_2_ per C–Br bond	Temp.	Time	Product

1	20%	Pd(dppf)Cl_2_	1	80 °C	48 h	0
2	20%	Pd(dppf)Cl_2_	10	80 °C	48 h	0
3	20%	Pd(dppf)Cl_2_	10	80 °C	48 h	trace
4	40%	Pd(dppf)Cl_2_	20	100 °C	48 h	30%

#### X-ray crystal structure analysis

Despite the many examples in literature of the crystal structure and packing of nonplanar porphyrins [3,6,8,13,52–53], few examples of crystal structure and packing analysis exist for arm-extended porphyrins. One of the few examples are azide-porphyrin derivatives reported by Flanagan et al. [43]. Here, five crystal structures were obtained of meso-*para*-phenyl arm-extended porphyrins (**26**, **27**, **28**, **29**, **33**) and two crystal structures for meso-*meta-*phenyl derivatives **36** and **37**. In addition, single crystal structures of **11** and **46** were determined. All structures were investigated using the NSD method [8–9], which allows a quantification and visualization of distortion modes.

It can be observed from the crystal structures that the porphyrins’ rings all exhibit the typical saddle-shaped conformation. Interestingly, substitution at the *para*- or *meta*-position can also induce partial ruffling of the porphyrin core (Table 5). Of all *para*-functionalized structures, porphyrin **33** bears the most similarity to that of compound **26**, with minimal ruffling observed and the overall magnitudes of out-of-plane and in-plane distortions are comparable.

**Table 5:** Mean geometrical parameters of OET-*meta*/*para*-ArylPP and out-of-plane and in-plane distortion magnitudes.

Compound	**26**	**27**	**28**	**29**	**33**	**36**	**37**	Units

pyrrole tilt	28.0	28.4	31.2	32.9	29.0	29.0	31.1	º
N–N dist. (adj)	2.70	2.71	2.71	2.72	2.73	2.71	2.71	Å
N–N dist. (opp)	3.77	3.80	3.80	3.81	3.83	3.78	3.79	Å
ΔC_meso_^a^	0.03	0.32	0.13	0.15	0.03	0.22	0.22	Å
ΔC_alpha_^b^	0.5	0.47	0.51	0.53	0.5	0.5	0.5	Å
ΔC_beta_^c^	1.21	1.13	1.28	1.32	1.21	1.21	1.26	Å
Δip^d^	1.06	1.03	1.22	1.28	1.09	1.14	1.24	Å
B_1g_	0.07	0.05	0.02	0.00	0.06	0.12	0.04	Å
E_u_(x)	0.08	0.00	0.07	0.00	0.05	0.00	0.05	Å
A_1g_	1.06	0.99	1.21	1.27	1.08	1.11	1.21	Å
A_2g_	0.00	0.27	0.11	0.17	0.04	0.21	0.22	Å
Δoop^e^	3.73	3.58	3.93	4.06	3.72	3.78	3.91	Å
B_2U_ (sad)	3.73	3.46	3.91	4.04	3.72	3.72	3.86	Å
B_1u_ (ruf)	0.00	0.92	0.35	0.45	0.1	0.66	0.61	Å
A_2U_ (dom)	0.11	0.06	0.01	0.00	0.04	0.14	0.04	Å

^a^Average displacement of meso-carbon atoms from the *xy* plane, (C5, C10, C15, and C20) relative to the 24-atom mean porphyrin plane (mean plane defined as Δ*z* = 0). ^b^Average displacement of α-carbon atoms from the *xy* plane (C1, C4, C6, C9, C11, C14, C16, C19) relative to the 24-atom mean porphyrin plane (Δ*z* = 0). ^c^Average displacement of β-carbon atoms from the *xy* plane (C2, C3, C7, C8, C12, C13, C17, C18) relative to the mean porphyrin plane (Δ*z* = 0). ^d^Average deviation of the 24-atom macrocycle (*x*,*y* coordinates) from the mean porphyrin plane, based on the least-squares method (mean plane defined as Δ*x* and Δ*y* = 0). ^e^Average deviation of the 24-atom macrocycle (*z* coordinates) with respect to the least-squares plane (mean plane defined as Δ*z* = 0) [54].

**Figure 3:**
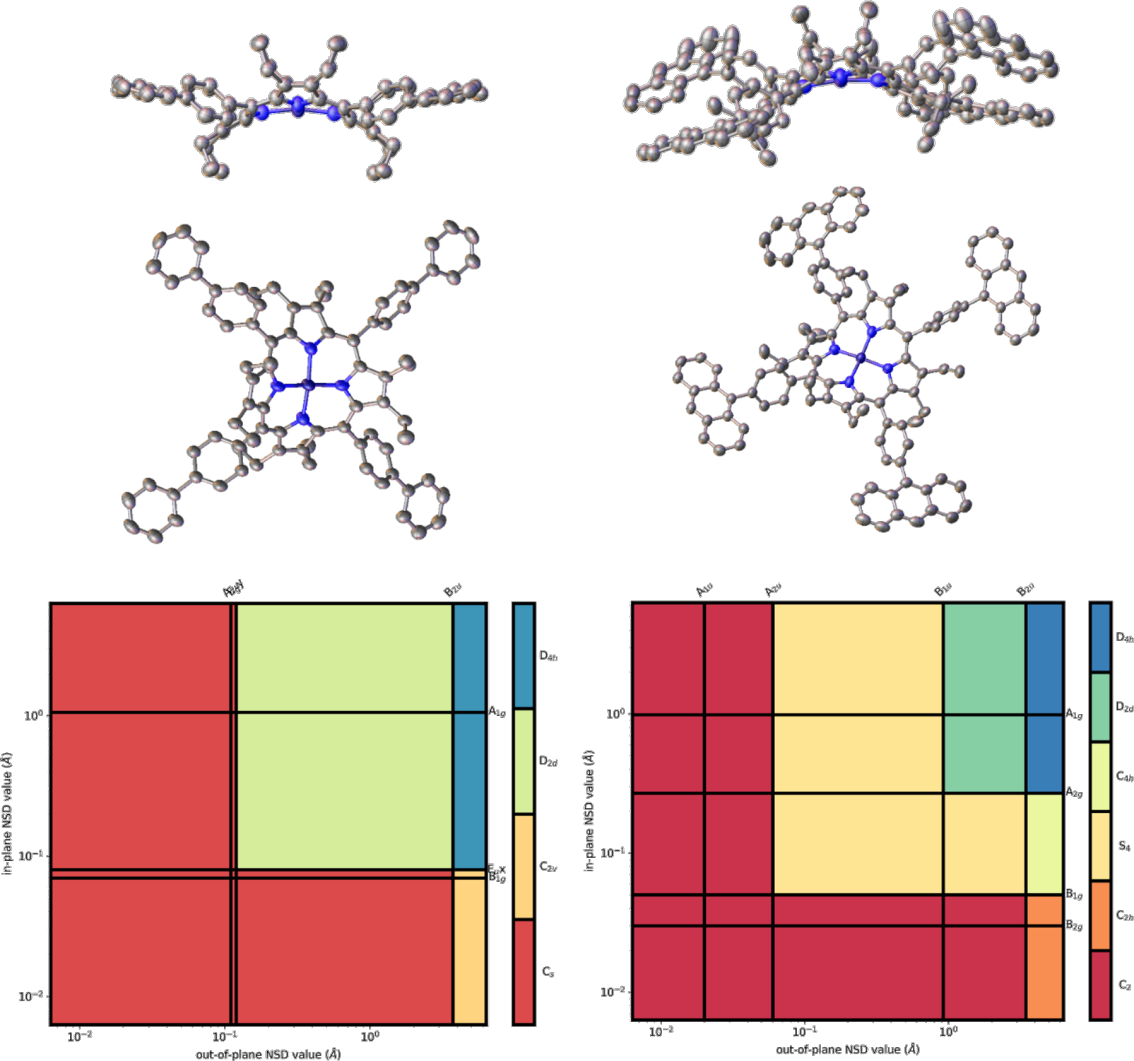
View of the molecular structure of compounds **26** (top left) and **27** (top right) with atomic displacements at 50% probability and hydrogen atoms omitted for clarity. Bottom left: Neoplastic representation of the molecular symmetry of compound **26**. Bottom right: Neoplastic representation of the molecular symmetry of compound **27**.

With compound **26**, no ruffling of the porphyrin ring is observed; however, with anthracene residues (**27**) a ruffling distortion of almost 1 Å is observed. This is not obvious at first, but differences in molecular symmetry [55] can be easily visualized using the neoplastic NSD plot [10] shown in Figure 3. Furthermore, the mean pyrrole ring tilt increases by 3–5° in the case of compounds **28** and **29** compared to that of compound **26**. Saddle-shape distortion is reduced compared to that of biphenyl **26**; this may be due to the proximity of the anthracene moiety to the β-ethyl positions, with C_23_–C_14B_ within 3.63 Å and their respective hydrogen atoms 1.97 Å away from each other. However, this does not account for the increased ruffling observed in porphyrins **28** and **29**, with similar distances to the β-ethyl groups as in porphyrin **26**, and despite this there is almost 0.5 Å magnitude of ruffle distortion. This may also be due to crystal packing effects or the Ni(II) metal center [56–57], as well as the crystallization solvent [43]. It is not possible to ascertain whether steric effects of the β-ethyl and the anthracenyl carbons are causing the ruffling observed, and a full statistical model of a large library of dodecasubstituted porphyrins is needed to understand these observed effects.

When comparing *meta*-substituted porphyrins **36** and **37**, ruffle distortion of the porphyrin ring is also observed. Interestingly in the case of the *meta*-anthracenyl derivative, the *para*-anthracenylporphyrin **27** experiences a magnitude of ≈0.3 Å less ruffling when compared to that of the *meta*-substituted porphyrin **37**. In terms of *meta*-phenyl-substituted porphyrin **36** a contribution of ruffling is observed, but no ruffling is observed in the planar analogue.

#### Crystal packing analysis of arm-extended *para*-substituted porphyrins

Nonplanar porphyrins are known to form supramolecular assemblies [6], either through hydrogen-bonding networks or through π–π interactions. Examples of this can be seen in the trapping of Keggin-type heteropolyoxometalate (POM) through nonplanar Mo(V)–porphyrin complexes [58], or porphyrin nanotubes/nanochannels by intermolecular π–π interactions of the peripheral phenyl groups [59]. Additionally nonplanar supramolecular assemblies have found use in anion capture [12,15], and sensing [60], making the synthesis of these structures desirable from a supramolecular standpoint.

**Figure 4:**
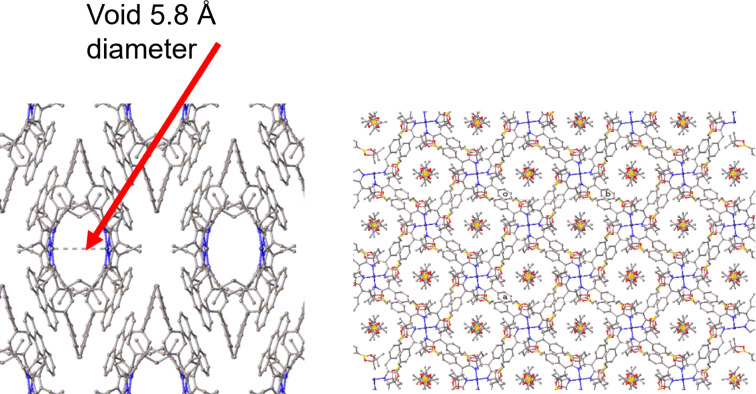
Left: packing diagram of **27** viewed normal to the *c*-axis showing the channels in the lattice with the solvent molecule density removed using masking in OLEX2. Hydrogen atoms omitted for clarity. Right: packing diagram of **46**, viewed normal to the *c*-axis, with bis(pinacolato)diboron occupying the cavities of the major **46**. Hydrogen atoms omitted for clarity.

Two especially interesting crystal packing features were that observed in the structures of compound **27** and borylated porphyrin **46**. In the case of porphyrin **27**, when crystallized by slow evaporation from CDCl_3_, a crystal structure with a void diameter of approximately 5.8 Å was obtained (Figure 4). The void was measured from the Ni(II)^...^Ni(II) vector approximately perpendicular to the metals through the *c*-axis.

Upon co-crystallization of borylated porphyrin **46** and bis(pinacolato)diboron, the accommodation of bis(pinacolato)diboron in the void of the lattice was observed (Figure 4). The crystal packing of this structure is quite similar to the supramolecular assembly of borylated porphyrin 5,10,15,20-tetrakis(5,5-dimethyl-1,3,2-dioxaborinan-2-yl)porphyrin, where nitrobenzene accommodated tunnels of width of 7–8 Å [61]. The assembly of compound **46** also presents channel-type voids, making it part of only a few porphyrins appended with boronic ester groups to be structurally disseminated by X-ray crystallography [62–63]. Compound **46** was found to crystallize in a 1:1 ratio with bis(pinacolato)diboron, with a void size of 8–9 Å. The formation of channel-type lattice structures is thermodynamically unfavorable, when compared to tightly packed arrangements, similar to nitrobenzene, bis(pinacolato)diboron may be templating the formation of these channels [61]. However, more research is needed to understand the formation of these supramolecular assemblies.

#### X-ray crystal structure analysis of compound **11**

**Figure 5:**
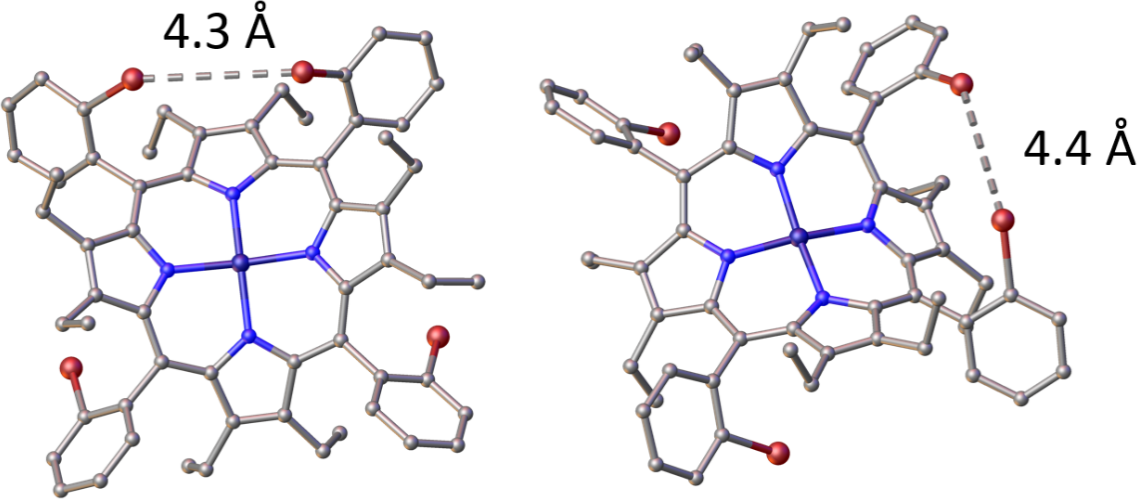
Left: view of part 0 2 in the molecular structure of the α_2_β_2_-atropisomer, **11** in the crystal, hydrogen atoms omitted for clarity. Displacement parameters shown at 50% probability and heteroatoms labelled only. Right: view of part 0 1 of the molecular structure of the α_2_β_2_-atropisomer, **11** in the crystal. Displacement parameters shown at 50% probability and heteroatoms labelled only.

As observed in the single crystal X-ray structure of **11** (Figure 5), the environment around the *ortho-*bromo-position is extremely sterically hindered.

Figure 5, shows the Br^…^Br separations in the α_2_β_2_-atropsiomer of compound **11** to be 4.3–4.4 Å. While only an illustration of the situation in the solid phase this illustrates that coupling phenyl, thiophene or other aryl moieties at this position would be extremely difficult. Furthermore, the distance between the *o*-bromine atoms and the nearest carbon neighbor of the β-ethyl groups is 3.7 Å, further complicates the success of coupling at this position. As discussed previously, enrichment to the αβαβ, isomer may be necessary to remove the impact of opposing bromine atoms on the coupling reaction. Separation of the four individual atropisomers (αβαβ, α_2_β_2_, α_3_β, α_4_) has been accomplished before in dodecasubstituted porphyrins through Ni(II) metalation [15]. The core metalation effect prevents inner core N–H tautomerism [64] and thus increases the structural symmetry of the macrocycle [65–66], leading to more facile atropisomeric separation. However, in the case of compound **11** the atropisomers could not be separated due to low rotational barriers and similar polarities, even with Ni(II) metalation. There are many other methods available to achieve different desired atropisomeric ratios. These include thermal enrichment [67–68], photoracemization [69–70], axial-ligand coordination [71], precise separation techniques [50] or a combination of the procedures mentioned [72]. Many more examples of atropisomeric enrichment methods can be found in a 2024 review on atropisomerism by Maguire et al. [73] and could be further explored to isolate the αβαβ-atropisomer of porphyrin **11**.

#### X-ray crystal structure analysis of compound **37**

**Figure 6:**
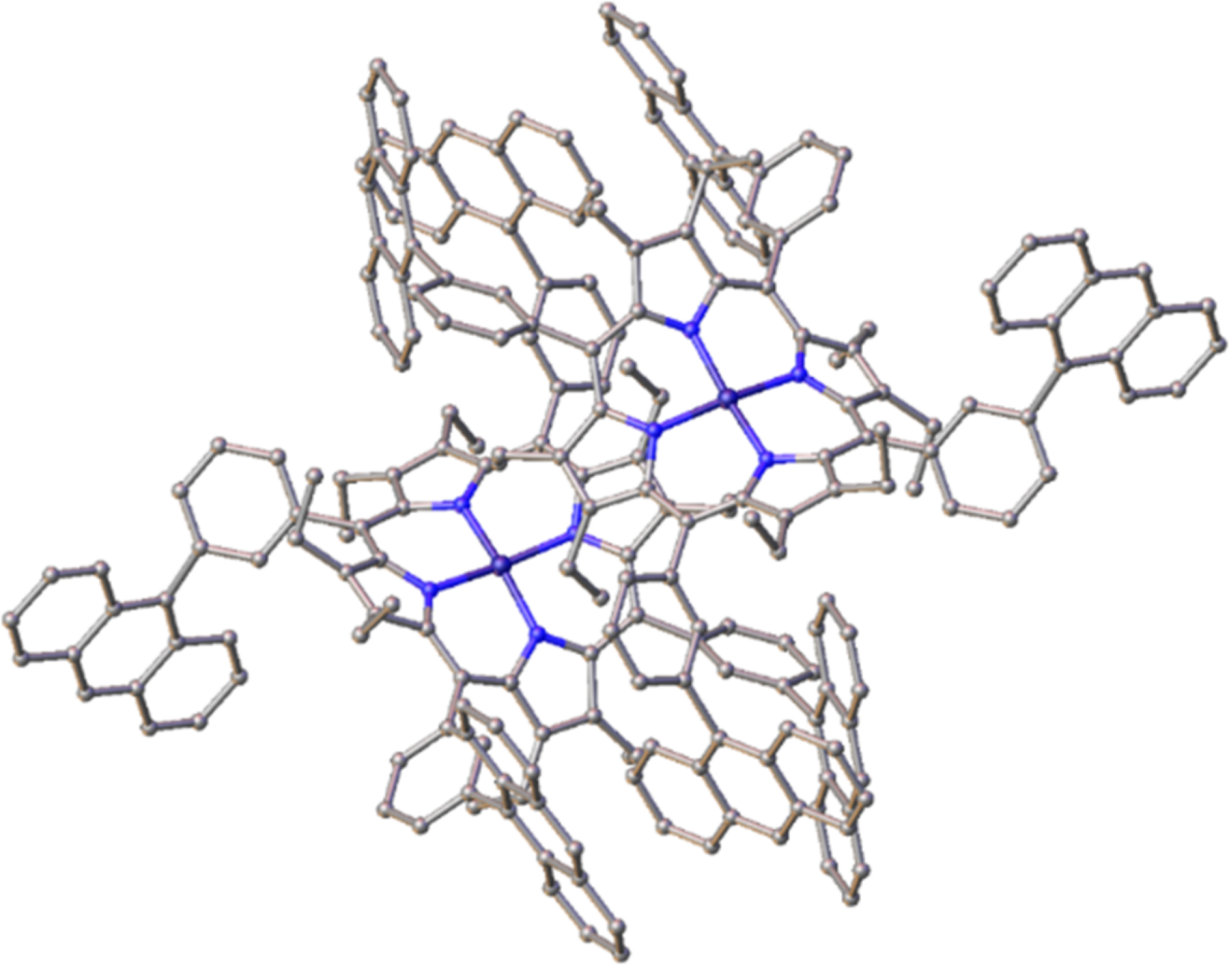
Schematic representation of porphyrin **37** showing a doubly intercalated structure.

Interestingly the anthracenyl arm-extension on the meso-*meta*-phenyl position revealed a doubly sandwiched, intercalated dimeric structure, wherein by two anthracenyl units is sandwiched a single anthracenylphenyl arm whilst a anthracenylphenyl arm is sandwiched on the opposing side of the molecules in the same fashion (Figure 6). Support of the existence of this structure in solution was obtained from VT-NMR studies (Figure S51 and Figure S52 in Supporting Information File 1)**,** with asymmetry observed in the β-ethyl CH_3_ resonances δ_H_ = 0.58 and 0.73 ppm and peak broadening in both the aromatic region and the {^1^H}^13^C NMR spectra. Coalescence of the β-ethyl resonances was observed when heating the sample to 70 °C and the same ^1^H NMR spectrum was observed after subsequent cooling of the sample as before heating. This indicates the thermodynamically favored dimeric structure to reassemble when cooling, reverting to the previous NMR trace.

## Conclusion

When considering sterically demanding systems with haloaryl and boronic acids as substrates for the Suzuki–Miyaura coupling, many may consider 2,6-alkyl-disubstituted phenyl rings as a model sterically demanding system to test the robust nature of both metal catalyst and ligand, for example, much work has been done on the synthesis of *ortho*-substituted biaryls, by the groups of Buchwald [74], Snieckus [75], Ackermann [76], and Tang [77] among others. Many of these examples have steric hindrance ‘adjacent’ to that of the reactive halogen/boronic site as opposed to the ‘adjacent’ and ‘opposite’ steric demand as seen with compound **11** with opposing bromines, coupled with the added complication of being a rotameric mixture, as well as adjacent hindrance of the nearby β-ethyl groups. Examples of palladium coupling on ‘opposing’ halogen atoms can be seen in the annulation of *vic*-bis(pinacolatoboryl)alkenes and -phenanthrenes [78]; yet, adjacent hindrance is not a problem in this case. Clearly, more work is required on the Suzuki–Miyaura coupling of molecules with sterically demanding ‘pockets’ with opposing and adjacent hindrance.

In conclusion, a library of arm-extended dodecasubstituted porphyrins was synthesized through classic C–C coupling reactions at the meso-phenyl position. It was found varying temperature and the pH of the solution are effective mitigations to overcome unfavorable reaction electronics or demanding sterics presented at the meso-phenyls’ *meta*- or *para*-position. Functionalization at the meso-phenyls’ *ortho*- position was not manageable and more research is needed to optimize conditions. Comparing the yields in coupling of borylated porphyrins and the halogenated analogues revealed a greater yield, when the polarity of the reaction was reversed; however, due to tedious synthesis and a lower yield over two steps, this synthetic approach is disadvantageous.

X-ray crystal structures were reported for almost half of these compounds. Crystal packing arrangements revealed this new library of arm-extended porphyrins as interesting candidates for the formation of supramolecular assemblies possibly capable of carrying out sensing and or capturing molecules of interest, as well as a dimeric intercalated structure.

## Supporting Information

File 1 Experimental methods, synthetic procedures, ^1^H, ^11^B and ^13^C NMR, VT-NMR, UV–vis, IR, HRMS (*m*/*z*)-APCI and HRMS (*m*/*z*)-LIFDI spectra and X-ray crystallographic data.

File 2 Crystallographic information files for porphyrins **11** (tcd2100), **28** (tcd2038), **27** (tcd2036), **36** (tcd2056), **26** (tcd2017), **29** (tcd2127), **37** (tcd2277), **46** (tcd2153) and **33** (tcd2288).

## Data Availability

All data that supports the findings of this study is available in the published article and/or the supporting information of this article.
