# Supplementary material for: C–C Coupling in sterically demanding porphyrin environments
Source: Beilstein J Org Chem. 2024 Nov 4;20:2784–98. doi: 10.3762/bjoc.20.234 (PMC11552435; doi:10.3762/bjoc.20.234)
Supplement: File 1 — Experimental methods, synthetic procedures, 1H, 11B and 13C NMR, VT-NMR, UV–vis, IR, HRMS (m/z)-APCI and HRMS (m/z)-LIFDI spectra and X-ray crystallographic data. [file Beilstein_J_Org_Chem-20-2784-s001.pdf]

BEILSTEIN JOURNAL OF ORGANIC CHEMISTRY

Supporting Information

for

C–C Coupling in sterically demanding porphyrin environments

Liam Cribbin, Brendan Twamley, Nicolae Buga, John E. O' Brien, Raphael Bühler, Roland A. Fischer and Mathias O. Senge

Beilstein J. Org. Chem. **2024,** *20,* 2784–2798. doi:10.3762/bjoc.20.234

Experimental methods, synthetic procedures, ^{1}H, ^{11}B and ^{13}C NMR, VT-NMR, UV–vis, IR, HRMS (*m*/*z*)-APCI and HRMS (*m*/*z*)-LIFDI spectra and X-ray crystallographic data

License and Terms: This is a supporting information file under the terms of the Creative Commons Attribution License (https://creativecommons.org/licenses/by/4.0). Please note that the reuse, redistribution and reproduction in particular requires that the author(s) and source are credited and that individual graphics may be subject to special legal provisions.
The license is subject to the *Beilstein Journal of Organic Chemistry* terms and conditions: (https://www.beilstein-journals.org/bjoc/terms)

Table of contents

Analytical methods and materials S2

Experimental methods S3

^{1}H and $\{^{1}H\}^{13}C$ NMR spectra: S32

Variable temperature NMR spectra: S81

Mass spectra: S82

UV–vis spectra: S90

IR spectra: S98

Single crystal X-ray crystallographic data and NSD analysis: S106

- Refinement details: S106
- X-ray crystallographic data refinement tables: S111
- Molecular structure view and NSD analysis of porphyrin **26**: S114
- Molecular structure view and NSD analysis of porphyrin **27:** S116
- Molecular structure view and NSD analysis of porphyrin **28:** S119
- Molecular structure view and NSD analysis of porphyrin **36:** S121
- Molecular structure view and NSD analysis of porphyrin **11:** S124
- Molecular structure view and NSD analysis of porphyrin **33:** S126
- Molecular structure view and NSD analysis of porphyrin **46:** S129
- Molecular structure view and NSD analysis of porphyrin **29:** S131

References S136

Analytical methods and materials

NMR spectra were recorded on a Bruker Advance III 400 MHz, a Bruker Advance HD 400, and an Agilent 400 spectrometer for ^{1}H (400.13 MHz), ^{11}B (128.41 MHz), and ^{13}C (100.61 MHz) NMR spectra. A Bruker Ultrashield 600 spectrometer was employed for ^{1}H (600.13 MHz) and ^{13}C (150.90 MHz) NMR and 2D NMR spectra. NMR experiments were in general performed at 25 °C. Resonances δ are given in ppm units and referenced to the deuterium peak in the NMR solvents, $CDCl_3$ (δ_H = 7.26 ppm, δ_C = 77.2 ppm). Variable temperature NMR experiments were conducted at 25 °C, 50 °C, and 70 °C. Signal multiplicities are abbreviated as follows: singlet = s, doublet = d, triplet = t, multiplet = m, quintet = qui, apparent broad singlet = appbrs, apparent doublet = appd, apparent doublet of doublets = appdd, apparent triplet = appt.

Atmospheric pressure chemical ionization (APCI) experiments were performed on a Bruker microTOF-Q III spectrometer interfaced to a Dionex UltiMate 3000 LC. Liquid injection field desorption ionization mass spectrometry (LIFDI-MS) data were measured on an Exactive Plus Orbitrap system by Thermo Fisher Scientific equipped with an LIFDI ion source from LINDEN CMS GmbH. Melting points are uncorrected and were measured with a Stuart SP-10 melting point apparatus. Analytical thin layer chromatography was performed using silica gel 60 (fluorescence indicator F254, precoated sheets, 0.2 mm thickness, 20 cm × 20 cm; Merck) and visualized by UV irradiation (λ = 254 nm). UV–vis absorption measurements were recorded in solutions using a Shimadzu UV-2600i spectrophotometer with quartz glass 10 mm cuvettes (1 cm path length quartz cell) and analyzed using Spectragryph version 1.2.16.1 software. IR measurements were conducted on a PerkinElmer Spectrum 100 FT-IR. Details of X-ray data collections are included in the single X-ray crystallographic data section as well as details on NSD symmetry analysis.

All chemicals were supplied by Sigma Aldrich, Acros Organics, Fluka, Frontier Scientific, Fluorochem, Enamine, and Fischer and handled without further purification unless otherwise stated. Starting materials 3,4-diethylpyrrole and [5,10,15,20-tetrakis(4-bromophenyl)-2,3,7,8,12,13,17,18-octaethylporphyrinato]nickel(II) were synthesized and purified prior to use. CH_2Cl_2 and $CHCl_3$ used in large scale reactions was distilled over P_2O_5. Toluene, THF, MeOH, and EtOH were also used as commercially available HPLC grade solvents. Reactions involving moisture and/or air-sensitive reagents were carried out in pre-dried glassware and with standard Schlenk line techniques. Microwave-assisted synthesis was performed with a Biotage Initiator$^+$ using Biotage microwave vials (2–5 mL) at high microwave adsorption conditions. Yields refer to chromatographically and spectroscopically (^{1}H NMR) homogeneous material, unless otherwise noted. Column chromatography was carried out using Fluka Silica Gel 60 (230–400 mesh; Merck).

Experimental methods

General procedure A for Suzuki–Miyaura cross coupling

Porphyrin **12**/**13** (30 mg, 0.025 mmol), 6.25 mol % per C–Br bond of palladium catalyst, tris(dibenzylideneacetone)dipalladium(0) (Pd_2dba_3, 6 mg, 0.01 mmol)*, SPhos (10 mg, 0.025 mmol, 1 equiv)*, K_3PO_4 (128 mg, 0.6 mmol, 24 equiv)/Cs_2CO_3 (325 mg, 0.6 mmol, 24 equiv)/NaOAc (49 mg, 0.6 mmol, 24 equiv)*, boronic acid/ester (0.3 mmol, 12 equiv)* were dissolved in an Ar-degassed solution of toluene and deionized water (20 mL, 1:1, v/v), shielded from ambient light, and stirred at 85 °C/110 °C for 24/48 h (Table S1, Figure S1). The resulting reaction mixture was washed with first deionized water (3 × 40 mL) and then with a saturated solution of sodium chloride (3 × 40 mL) before drying over Na_2SO_4. The solvent was removed at reduced pressure. The crude mixture was then purified by column chromatography using SiO_2.

General procedure B for Nickel (II) metalation of porphyrins

Crude porphyrin (1.0 g) and Ni(II)(acac)$_2$ (1.1 g, 4.31 mmol) were added to a flask containing 100 mL of toluene. The resulting solution was stirred at 120 °C for 18 h under an Ar atmosphere. The solvent was removed under reduced pressure. The resulting purple solid was purified by filtration through a silica plug using CH_2Cl_2 as an eluent.

Table S1: Conditions used as part of the Suzuki–Miyaura coupling studies on dodecasubstituted porphyrins.

Entry	Catalyst/ligand (SPhos 1 equiv used)	Cat. mol % per C–Br	Base (24 equiv)	Temperature	Time	Boronic acid/ester (3 equiv per C–Br)	Yield of product (%) (porphyrin)
1	Pd_2dba_3/Sphos	6.25%	K_3PO_4	85 ˚C	48 h	**14**	32% **(26)**
2	Pd_2dba_3/SPhos	6.25%	K_3PO_4	110 ˚C	48 h	**15**	39% **(27)**
3	Pd_2dba_3/SPhos	6.25%	K_3PO_4	110 ˚C	48 h	**17**	48% **(28)**[a]
4	Pd_2dba_3/SPhos	6.25%	Cs_2CO_3	85 ˚C	48 h	**18b**	72% **(29)**[b]
5	Pd_2dba_3/SPhos	6.25%	Cs_2CO_3	85 ˚C	48 h	**16b**	8% **(30)**
6	Pd_2dba_3/SPhos	25%	Cs_2CO_3	110 ˚C	24 h	**23**	16% **(31)**
7	Pd_2dba_3/SPhos	6.25%	Cs_2CO_3	85 ˚C	24 h	**25**	47% **(32)**
8	Pd_2dba_3/SPhos	6.25%	Cs_2CO_3	110 ˚C	24 h	**21**	56% **(33)**
9	Pd_2dba_3/SPhos	12.5%	K_3PO_4	110 ˚C	24 h	**20**	25% **(34)**
10	Pd_2dba_3/SPhos	12.5%	Cs_2CO_3	110 ˚C	24 h	**24**	58% **(35)**
11	Pd_2dba_3/SPhos	6.25%	K_3PO_4	85 ˚C	48 h	**14**	16% **(36)**[a]
12	Pd_2dba_3/SPhos	12.5%	K_3PO_4	110 ˚C	48 h	**15**	32% **(37)**
13	Pd_2dba_3/SPhos	12.5%	K_3PO_4	110 ˚C	24 h	**17**	7% **(38)**
14	Pd_2dba_3/SPhos	12.5%	Cs_2CO_3	85 ˚C	48 h	**18b**	23% **(39)**
15	Pd_2dba_3/SPhos	12.5%	NaOAc	110 ˚C	24 h	**16a**	18% **(40)**[c]

16	Pd_2dba_3/SPhos	25%	Cs_2CO_3	110 ˚C	24 h	**23**	4% **(41)**[c]
17	Pd_2dba_3/SPhos	12.5%	Cs_2CO_3	110 ˚C	24 h	**25**	30% **(42)**[b]
18	Pd_2dba_3/SPhos	12.5%	Cs_2CO_3	110 ˚C	24 h	**21**	10% **(43)**[b]
19	Pd_2dba_3/SPhos	12.5%	K_3PO_4	110 ˚C	24 h	**20**	30% **(44)**[b]
20	Pd_2dba_3/SPhos	12.5%	Cs_2CO_3	110 ˚C	24 h	**24**	31% **(45)**[b]

Note* Catalyst loading and ligand loading scaled accordingly with Table S1 as well as base used and conditions of temperature and time of the reaction. [a]Two times scale. [b]Three times scale. [c]Five times scale.

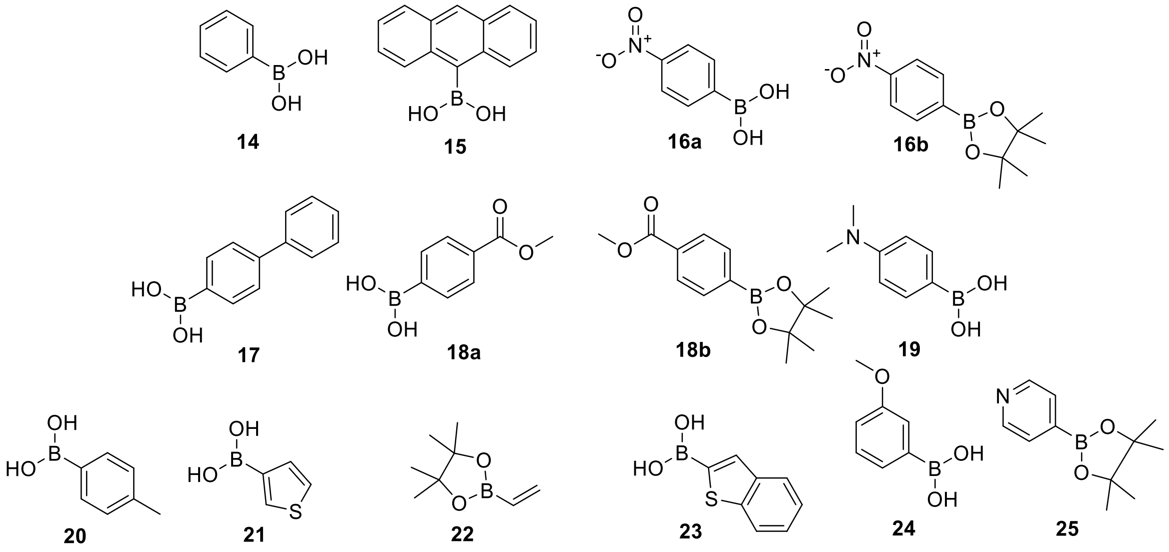

Figure S1: Boronic acids and boronic esters used as part of the scope of the study.

[5,10,15,20-Tetrakis(4-biphenyl)-2,3,7,8,12,13,17,18-octaethylporphyrinato]nickel(II) (26)

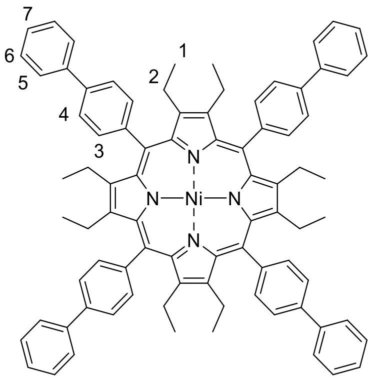

The title compound **26** was synthesized through general procedure A and then purified by column chromatography (SiO_2, DCM/hexane 50:50, v/v, R_f 0.6). The product eluted as the first band from the column and the solvent was removed at reduced pressure. Compound **26** was then redissolved in the minimum amount of DCM and recrystallized by layering

with MeOH. The product was isolated via gravity filtration as purple crystals (16 mg, 32% yield), mp >300 ˚C (from methanol). ^{1}H NMR ($CDCl_3$, 600 MHz): δ = 0.60 (t, J = 7.3 Hz, 24H, H-1), 2.35 (appbrs, 16H, H-2), 7.48 (t, J = 7.6 Hz, 4H, H-7), 7.60 (appt, J = 7.5 Hz, 8H, H-6), 7.91 (d, J = 7.1 Hz, 8H, H-5), 7.93 (d, J = 7.8 Hz, 8H, H-4) 8.19 ppm (d, J = 7.8 Hz, 8H, H-3). ^{13}C NMR ($CDCl_3$, 150MHz): δ_c = 17.2, 19.9, 125.7 (q), 127.4, 127.8, 129.0, 129.2, 134.9, 139.4(q), 140.8(q), 149.1(q), 145.7 (q) ppm. HRMS (m/z)-APCI: (M+H) Calcd for $(C_{84}H_{77}N_4Ni)^+$: 1199.5496; Found 1199.5494. UV/Vis ($CHCl_3$): λ_{max} (log ε) = 438 (5.28), 554 (4.51), 587 nm (4.44). IR (ATR): ṽ = 2923.8, 2854.6, 2156.9, 1720.9, 1600.9, 1448.9, 1372.9, 1316.9, 1260.8, 1169.9, 1018.8, 886.9, 793.5, 754.8, 694.8 cm^{-1}. A crystal of **26** was grown at ambient temperature, in an LC/GC vial using 0.5–1 mg of porphyrin, with chloroform as solvent and methanol as an anti-solvent in a 1:1 ratio. Crystals grew upon diffusion of chloroform. For details on XRD determination, see the refinement details section and Table S2. A neoplastic representation (Figure S126) and in-plane and out-of-plane skeletal images of the porphyrin (Figure S127) were generated and magnitudes of distortion (Table S5) were calculated using the NSD symmetry tool.

[2,3,7,8,12,13,17,18-Octaethyl-5,10,15,20-tetrakis(9-phenylanthracene)porphyrinato] nickel(II) (27)

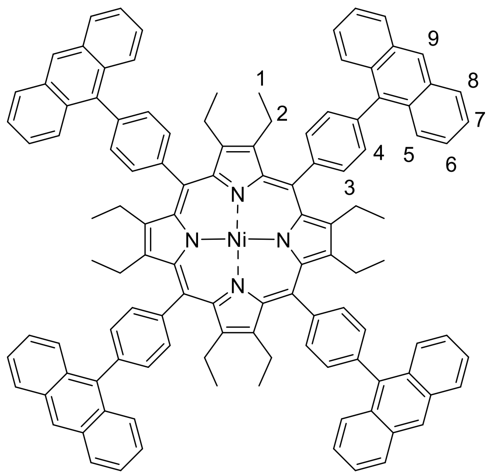

The title compound **27** was synthesized by general procedure A and then purified by column chromatography (SiO_2, DCM/hexane 75:25, v/v, R_f 0.2). The product eluted as the first band from the column and the solvent was removed at reduced pressure. Compound **27** was then redissolved in the minimum amount of DCM and recrystallized by layering with MeOH. The product was isolated via gravity filtration as purple crystals (15 mg, 39% yield) mp > 300 ˚C (from methanol). ^{1}H NMR ($CDCl_3$, 600 MHz): δ = 0.91 (t, J = 7.0 Hz, 24H, H-1, overlapping w/ hexane), 2.77 (appbrs, 16H, H-2), 7.54 (ddd, J = 8.8, 6.3, 1.4 Hz, 8H, H-7), 7.58 (ddd, J = 8.3, 6.3, 1.2 Hz, 8H, H-6) 7.77 (d, J = 7.9 Hz, 8H, H-3), 8.06 (d, J = 8.8 Hz, 8H, H-5), 8.18 (d, J = 9.0 Hz, 8H, H-8), 8.44 (d, J = 7.9 Hz, 8H, H-4), 8.64 ppm (s, 4H, H-9). ^{13}C NMR ($CDCl_3$, 150 MHz): δ_c = 17.6, 117.2(q), 125.4, 125.8, 127.1, 128.8, 130.4, 130.5, 131.7 (q), 134.6, 137.1 (q), 138.8 (q), 139.6 (q), 144.9 (q), 146.0 (q) ppm. HRMS (m/z)-LIFDI: (M+H) Calcd for $(C_{116}H_{93}N_4Ni)^+$: 1599.6709; Found 1599.6559. UV/Vis (CH_2Cl_2): λ_{max} (log ε) = 437 (5.48), 553 (4.10), 589 nm (3.83). IR (ATR): ṽ = 3270.2, 2979.7, 1699.9, 1584.5, 1519.3, 1366.1, 1311.1, 1229.7, 1153.1, 1049.4, 956.3, 848.01, 785.39, 731.93 cm^{-1}. The crystal of porphyrin **27** suitable for XRD, was grown at ambient temperature in an NMR tube using approx. 5 mg of porphyrin, with deuterated chloroform as solvent. Crystals grew on slow evaporation of chloroform. For details on XRD determination, see the refinement details section and Table S2**.** A neoplastic representation (Figure S130) and in-plane and out-of-plane skeletal images of the porphyrin (Figure S131) were generated and magnitudes of distortion (Table S6) were calculated using the NSD analyzer tool.

[2,3,7,8,12,13,17,18-Octaethyl-5,10,15,20-tetrakis(4,4'-triphenyl)porphyrinato] nickel(II) (28)

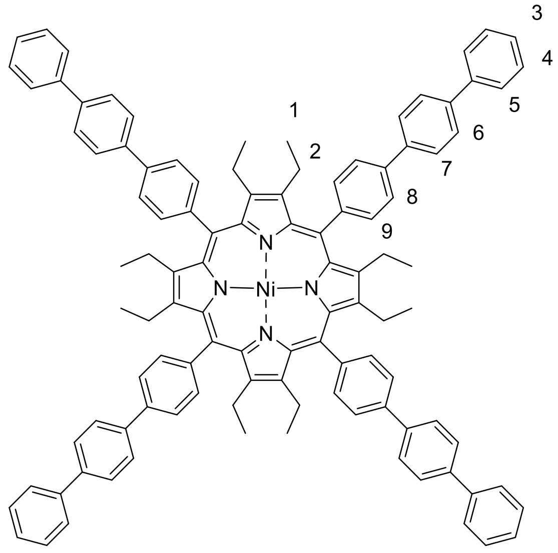

The title compound **28** was synthesized by general procedure A and then purified by column chromatography (SiO_2, DCM/hexane 25:75, v/v, R_f 0.25) eluting as the first band from the column and the solvent was removed at reduced pressure. Compound **28** was then redissolved in the minimum amount of DCM and recrystallized by layering with MeOH. The product was isolated via gravity filtration as purple crystals (30 mg, 48% yield) mp >300 ˚C (from methanol). ^{1}H NMR ($CDCl_3$, 400 MHz): δ = 0.63 (t, J = 7.4 Hz, 24H, H-1), 2.31 (appbrs, 16H, H-2), 7.44 (t, J = 7.4Hz, 4H, H-3), 7.54 (dd, J = 8.4, 6.9 Hz, 8H, H-4), 7.75 (m, 8H, H-5), 7.84 (d, J = 8.4 Hz, 8H, H-7), 7.98 (d, J = 8.2 Hz, 8H, H-9), 8.02 (d, J = 8.3 Hz, 8H, H-6), 8.22 ppm (d, J = 8.2 Hz, 8H, H-8). ^{13}C NMR ($CDCl_3$, 100MHz): δ_c = 17.6, 116.8 (q), 125.9, 127.6, 128.1, 128.2, 129.4, 135.4, 139.8 (q), 140.2 (q), 140.5 (q), 141.0(q), 141.3(q), 145.2 (q), 146.1(q) ppm. HRMS (m/z)-LIFDI: (M+H) Calcd for $(C_{108}H_{93}N_4Ni)^+$: 1503.6748.; Found 1503.6803. UV/Vis (CH_2Cl_2): λ_{max} (log ε) = 440 (5.39), 553 (4.90), 586 nm (4.87). IR (ATR): ṽ = 2970.8, 2928.1, 1699.5, 1608.7, 1584.9, 1519.7, 1540.0, 1453.9, 1391.8, 1366.3, 1311.1, 1288.5, 1230.9, 1154.3, 1109.3, 1049.5, 1017.3, 973.1, 956.5, 903.8, 849.1, 826.2, 791.5, 733.8, 690.6 cm^{-1}. Crystals of porphyrin **28** were grown at ambient temperature in a LC/GC vial using approx. 0.5–1 mg of porphyrin, with

dichloromethane. Crystals grew on slow evaporation of dichloromethane. For details on XRD determination, see the refinement details section and Table S2**.** A neoplastic representation (Figure S134) and in-plane and out-of-plane skeletal images of the porphyrin (Figure S135) were generated and magnitudes of distortion (Table S7) were calculated using the NSD analyzer tool.

[5,10,15,20-Tetrakis(3-biphenyl)-2,3,7,8,12,13,17,18-octaethylporphyrinato]nickel(II) (36)

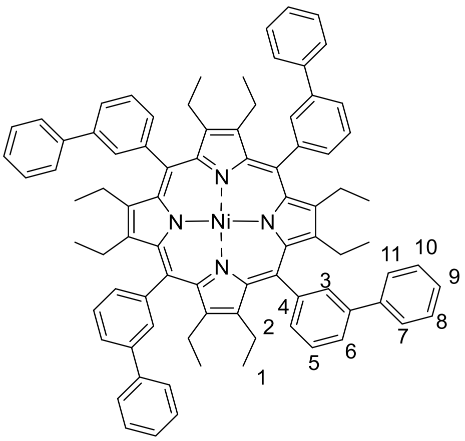

The title compound **36** was synthesized by general procedure A and then purified by column chromatography (SiO_2, DCM/hexane 25:75, v/v, R_f 0.7). The compound eluted as the second band from the column and the solvent was removed at reduced pressure. Compound **36** was then redissolved in the minimum amount of DCM and recrystallized by layering with MeOH. The product was isolated via gravity filtration as purple crystals, as a rotameric mixture (8 mg, 16% yield). mp >300 ˚C (from methanol). ^{1}H NMR ($CDCl_3$, 600 MHz): δ_c = 0.60 (t, J = 7.4 Hz, 24H, H-1), 2.55 (appbrs, 16H, H-2), 7.42 (m, 4H, H-9), 7.51 (d, J = 8.1 Hz, 4H, H-11), 7.54 (d, J = 9.1 Hz, 4H, H-7), 7.70 (m, 4H, H-6), 7.79 (m, 4H, H-10), 7.81 (m, 4H, H-8), 7.93 (d, J = 7.7, 4H, H-5), 8.11 (m, 4H, H-4), 8.36 ppm (m, 4H, H-3). ^{13}C NMR ($CDCl_3$, 150MHz): δ_c = 17.2, 19.8, 127.0, 127.6, 127.7, 127.9, 129.0, 129.1, 133.3, 133.7, 139.8 (q), 140.7q), 141.4 (q), 144.6 (q), 145.7 (q) ppm. HRMS (m/z)-

APCI: (M+H) Calcd for $(C_{84}H_{77}N_4Ni)^+$: 1199.5496; Found 1199.5427. UV/Vis (CH_2Cl_2): λ_{max} (log ε) 441 (4.95), 555 (4.30), 587 nm (4.25). Single crystals of porphyrin **36** were grown at ambient temperature, in a crystallization tube using approx. 3–5 mg of porphyrin and DCM as a solvent, layering with MeOH. Crystals grew at the interface between the two layers. For details of XRD determination, see the refinement details section and Table S3**.** A neoplastic representation (Figure S138) and in-plane and out-of-plane skeletal images of the porphyrin (Figure S139) were generated and magnitudes of distortion (Table S8) were calculated using the NSD analyzer tool.

[2,3,7,8,12,13,17,18-Octaethyl-5,10,15,20-tetrakis(3-methoxycarbonylbiphenyl)porphyrinato]nickel(II) (39)

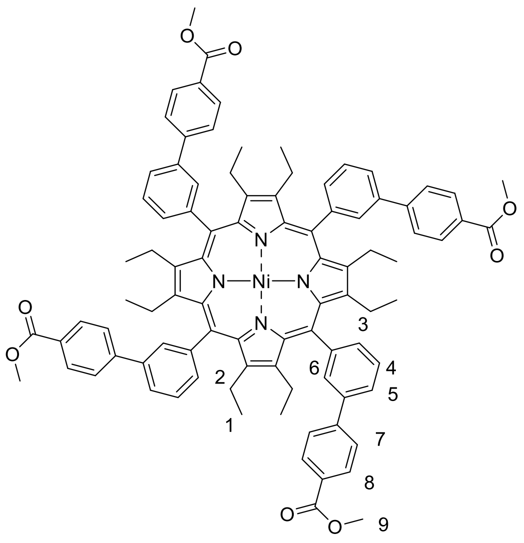

The title compound **39** was synthesized by general procedure A and then was purified by column chromatography (SiO_2, DCM/MeOH 95:5, v/v, R_f 0.2). The product eluted as the fourth band from the column. The solvent was removed at reduced pressure. Compound **39** was then redissolved in the minimum amount of DCM and recrystallized by layering with MeOH. The product was isolated via gravity filtration as purple crystals, as a rotameric mixture (8 mg, 23% yield) mp > 300 ˚C (from methanol). ^{1}H NMR ($CDCl_3$, 400 MHz): δ =

0.59 (t, 24H, J = 6.8Hz, H-1), 2.03-2.55 (appbr d, 16H, H-2), 3.98 (s, 12H, H-9) 7.74 (m, 4H, H-5) 7.85 (appdd, J = 8.5, 3.5 Hz, 8H, H-8), 7.96 (dd, 4H, J = 7.5, 1.6 Hz, H-4), 8.10 (m, 4H, H-3) 8.18 (m, 8H, H-7) 8.37 ppm (m, 4H, H-6). ^{13}C NMR ($CDCl_3$, 100MHz): δ_C = 17.2, 20.0, 52.4, 116.9 (q), 127.3, 127.5, 128.0, 129.3, 130.5, 133.0, 134.1 (q), 138.7 (q), 140.9 (q), 144.6 (q), 145.8 (q), 167.2 (q) ppm. UV/Vis (CH_2Cl_2): λ_{max} (log ε) =435 (5.10), 554 (3.93), 588 (3.85). HRMS (m/z)-APCI: (M+H) Calcd for $(C_{92}H_{85}N_4O_8Ni)^+$: 1431.5715; Found 1431.5719. IR (ATR): ṽ = 3987.6, 3355.9, .2927.6, 2536.9, 2158.9, 1595.6 (C=O bond stretch strong), 1255.2, 808.01 cm^{-1}.

[2,3,7,8,12,13,17,18-Octaethyl-5,10,15,20-tetrakis(4-methoxycarbonylbiphenyl)porphyrinato]nickel(II) (29)

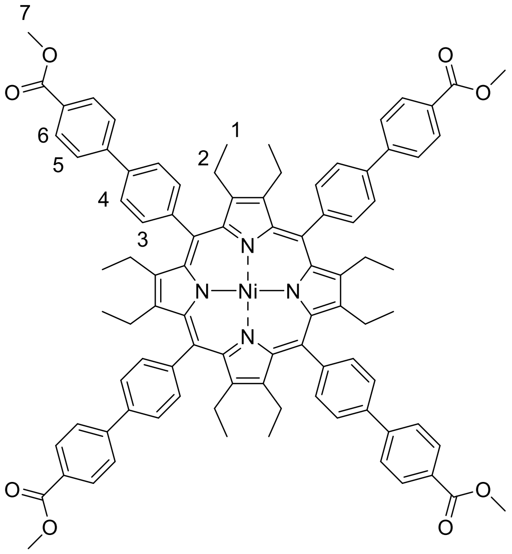

The title compound **29** was synthesized by general procedure A. The crude mixture was purified by column chromatography (SiO_2, DCM/MeOH 95:5, v/v, R_f 0.4). The solvent was then removed at reduced pressure. Compound **29** was then redissolved in the minimum amount of DCM and recrystallized by layering with hexane. The product was isolated via gravity filtration as purple crystals (78 mg, 72% yield) mp > 300 ˚C (from hexane). ^{1}H NMR ($CDCl_3$, 400 MHz): δ = 0.59 (t, J = 7.4Hz, H-2, 24H), 2.07-2.48 (appbrs, H-1, 16H), 4.02 (s,

H-3, 12H) 7.95 (d, J = 8.0Hz, H-7, 8H), 7.98 (d, J = 8.5Hz, H-5, 8H), 8.22 (d, J = 8.0Hz, H-6, 8H), 8.26 (d, J = 8.5Hz, H-4, 8H). ^{13}C NMR ($CDCl_3$, 100MHz): δ_c = 17.2, 19.9, 116.8, 125.9, 127.4, 130.5, 135.0, 139.9, 141.0, 144.5, 145.0, 145.7, 166.6 ppm. UV/Vis (CH_2Cl_2): λ_{max} (log ε) = 440 (5.27), 555 (4.41), 587 (4.37). HRMS (m/z)-APCI: (M+H) Calcd for $(C_{92}H_{85}N_4O_8Ni)^+$: 1431.5715; Found 1431.5645. IR (ATR): ṽ = 2965.9, 2870.9, 1719.8, 1608.8, 1434.8, 1372.9, 1274.7, 1177.7, 1053.5, 815.4, 771.7, 701.9 cm^{-1}. Crystals of porphyrin **29** suitable for XRD were grown at ambient temperature in an NMR tube using approx. 5 mg of porphyrin with deuterated chloroform as solvent. Crystals grew upon slow evaporation of chloroform. For details on XRD determination, see the refinement details section and Table S3**.** A neoplastic representation (Figure S154) and in-plane and out-of-plane skeletal images of the porphyrin (Figure S155) were generated and magnitudes of distortion were calculated using the NSD symmetry tool.

[2,3,7,8,12,13,17,18-Octaethyl-5,10,15,20-tetrakis(4-nitrobiphenyl)porphyrinato]nickel(II) (30)

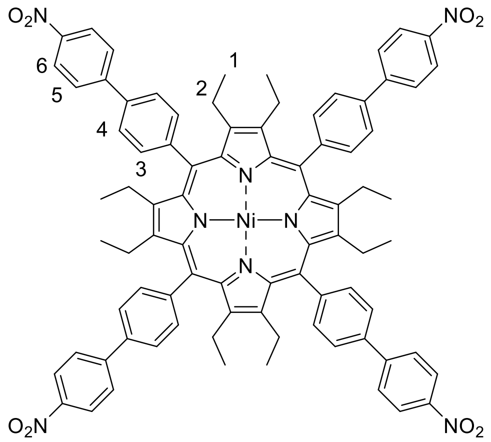

The title compound **30** was synthesized by general procedure A. The crude mixture was purified by column chromatography (SiO_2, DCM/MeOH 95:5, v/v, R_f 0.4). The solvent was removed at reduced pressure. Compound **30** was then redissolved in the minimum amount of DCM and recrystallized by layering with hexane. The product was isolated via

gravity filtration as purple crystals (8 mg, 8% yield) mp > 300ºC (from hexane). ^{1}H NMR ($CDCl_3$, 400 MHz): δ = 0.58 (t, J = 7.4 Hz, 24H, H-1), 2.07-2.45 (appbrs, 16H, H-2), 7.94 (d, J = 8.2 Hz, 8H, H-3), 8.03 (d, J = 8.8 Hz, 8H, H-5), 8.24 (d, J = 8.2 Hz, 8H, H-4), 8.43 ppm (d, J = 8.8 Hz, 8H, H-6). ^{13}C NMR ($CDCl_3$, 100 MHz): δ_c = 17.1, 20.0, 116.7 (q), 124.5, 127.5 (q), 128.1, 132.9 (q), 134.7, 137.6 (q), 140.9 (q), 143.9 (q), 145.8 (q), 147.3 (q) ppm. UV/Vis (CH_2Cl_2): λ_{max} (log ε) = 442 (5.33), 558 (4.69), 592 (4.63). HRMS (m/z)-APCI: (M+H) Calcd for $(C_{84}H_{73}N_8O_8Ni)^+$: 1379.4899; Found 1379.4891. IR (ATR): ṽ = 2969.9, 2868.9, 2161.5, 1599.8, 1450.5, 1372.2, 1259.5, 1164.2, 1107.4, 1020.3, 863.6, 775.7 cm^{-1}.

[5,10,15,20-Tetrakis(3-bromophenyl)-2,3,7,8,12,13,17,18-octaethylporphyrinato]nickel(II) (12)

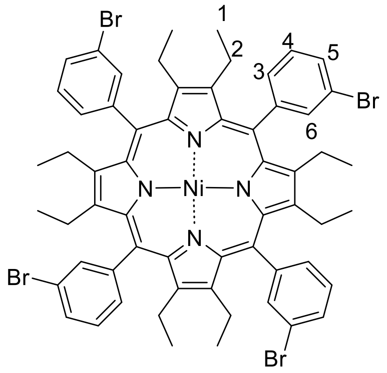

Dry CH_2Cl_2 (1.68 L), 3,4-diethylpyrrole (1.26 g, 13.64 mmol) and 3-bromoaldehyde (2.53 g, 13.64 mmol), were added to a 2L three-necked flask and degassed with argon for 30 minutes. The resulting solution was protected from ambient light and $BF_3 \cdot OEt_2$ (168 µL, 1.36 mmol) was added to the mixture and stirred for 18 h at room temperature. DDQ (3.09 g, 1.36 mol) was added, and the solution was stirred for 1 hour. The reaction was then quenched with excess triethylamine (2.57 mL). The solvent was evaporated to dryness and the residue was redissolved in CH_2Cl_2. The mixture was purified by column

chromatography (SiO_2, DCM/MeOH 95:5, v/v, R_f 0.1). The solvent was removed under reduced pressure resulting in a crude green solid. The green solid was then subjected to a nickel(II) metalation according to general procedure B, and purified by column chromatography using DCM as eluent (R_f 0.9). Compound **12** was then redissolved in the minimum amount of DCM and recrystallized by layering with MeOH. The product was isolated via gravity filtration as purple crystals, as a rotameric mixture (1.131 g, 28% yield) mp > 300 ˚C (from methanol). ^1H NMR ($CDCl_3$, 400 MHz): δ = 0.63 (t, J = 7.4 Hz, 24H, H-1), 2.01-2.56 (appbrs, 16H, H-2), 7.54 (m, 4H, H-6), 7.87 (dd, J = 7.9, 2.0 Hz, 4H, H-3), 8.08 (m, 4H, H-5), 8.25-8.39 ppm (m, 4H, H-4). ^{13}C NMR ($CDCl_3$, 100MHz): $δ_c$ = 17.1, 19.9, 116.0 (q), 121.2 (q), 128.9, 131.3, 133.1, 137.0, 142.2 (q), 144.7 (q), 147.8 (q). UV/Vis (CH_2Cl_2): $λ_{max}$ (log ε) = 434 (5.38), 555 (4.49), 591 nm (4.47). HRMS (m/z)-APCI: (M+H) Calcd for $(C_{60}H_{57}Br_4N_4Ni)^+$: 1207.0665; Found 1207.0658. IR (ATR): ṽ = 2966.7, 2867.7, 1557.7, 1449.7, 1371.8, 1326.8, 1257.8, 1167.8, 1019.7, 940.8, 872.8, 773.2, 691.7 cm^{-1}.

[5,10,15,20-Tetrakis(2-bromophenyl)-2,3,7,8,12,13,17,18-octaethylporphyrinato]nickel(II) (11)

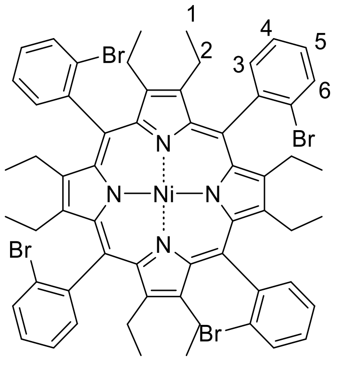

Dry CH_2Cl_2 (1.68 L), 3,4-diethylpyrrole (1.26 g, 13.64 mmol) and 2-bromoaldehyde (2.53 g, 13.64 mmol), were added to a 2L three-necked flask and degassed with argon for 30 minutes. The resulting solution was protected from ambient light and $BF_3·OEt_2$ (168 µL,

1.36 mmol) was added to the mixture and let stir for 18 h at room temperature. DDQ (3.09 g, 1.36 mol) was added, and the solution was stirred for 1 hour. The reaction was then quenched with excess triethylamine (2.57 mL). The solvent was evaporated to dryness and the residue was redissolved in CH_2Cl_2. The mixture was purified by column chromatography (SiO_2, DCM/MeOH 95:5, v/v, R_f 0.1). The solvent was evaporated at reduced pressure resulting in a crude green solid. The green solid was then subjected to a nickel(II) metalation according to general procedure B, and purified using column chromatography using an eluent of DCM (R_f = 0.8). The solvent was removed at reduced pressure. Compound **11** was then redissolved in the minimum amount of DCM and recrystallized by layering with MeOH. The product was isolated via gravity filtration as purple crystals (739 mg, 18% yield) mp > 300 ˚C (from methanol). Note* Atropoisomeric mixture could not be separated. ^{1}H NMR ($CDCl_3$, 400 MHz): δ = 0.63 (m, 24H, H-1), 2.05-2.54 (appbrs, 16H, H-2), 7.54 (m, 4H, H-5), 7.59 (m, 4H, H-4), 7.89 (m, 4H, H-3), 8.08-8.27 ppm (m, 4H, H-6). ^{13}C NMR ($CDCl_3$, 100MHz): δ_{c} = 16.8, 19.7, 115.9 (q), 125.5 (q), 126.3, 128.4 (q), 129.3, 130.0 (q), 132.3 (q), 136.1 (q), 140.7 (q), 144.3 (q), 145.2 ppm (q). UV/Vis (CH_2Cl_2): λ_{max} (log ε) = 437 (5.22), 559 (4.33), 597 nm (4.35). HRMS (m/z)-APCI: (M+H) Calcd for $(C_{60}H_{57}Br_4N_4Ni)^+$: 1207.0665; Found 1207.0674. IR (ATR): ṽ = 2965.8, 2869.8, 1585.9, 1424.8, 1372.8, 1313.8, 1261.8, 1170.8, 1021.7, 886.9, 846.3, 749.51 cm^{-1}. Single crystals of **11**, were grown at ambient temperature in a crystallisation tube, using approx. 10 mg of porphyrin in DCM as a solvent, layering with MeOH. Crystals grew at the interface between the two layers. For details on XRD determination, see the refinement details section and Table S3. A neoplastic representation (Figure S142) and in-plane and out-of- plane skeletal images of the porphyrin (Figure S143) were generated and magnitudes of distortion (Table S9) were calculated using the NSD symmetry tool.

[2,3,7,8,12,13,17,18-Octaethyl-5,10,15,20-tetrakis-(3-methoxy-4-biphenyl)-porphyrinato]nickel(II) (35)

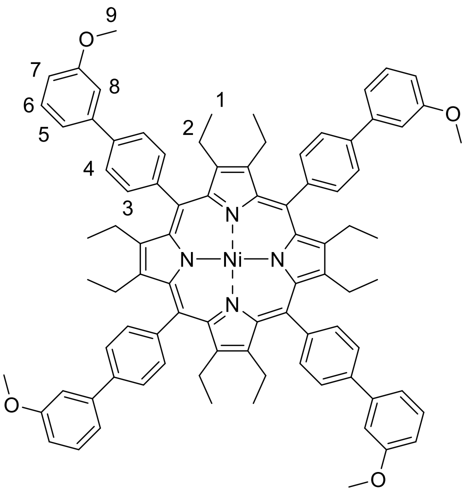

The title compound **35** was synthesized by general procedure A. The crude mixture was purified by column chromatography (SiO_2, DCM/hexane 60:40, v/v, R_f 0.4). The solvent was removed at reduced pressure. Compound **35** was then redissolved in the minimum amount of DCM and recrystallized by layering with hexane. The product was isolated via gravity filtration and isolated as purple crystals (19 mg, 58% yield) mp > 300 ˚C (from hexane). ^{1}H NMR ($CDCl_3$, 400 MHz): δ = 0.57 (t, J = 7.4 Hz, 24H, H-1), 2.05-2.54 (appbrs, 16H, H-2), 3.97 (s, 12H, H-9), 7.00 (m, 4H, H-5), 7.42 (m, 4H, H-6), 7.50-7.52 (m, 8H, H-7/8, overlapping), 7.88 (d, *J* = 8.2 Hz, 8H, H-3), 8.16 ppm (d, *J* = 8.2 Hz, 8H, H-4). ^{13}C NMR ($CDCl_3$, 100 MHz): δ_c = 17.3, 20.0, 55.7, 113.1, 113.2, 116.9 (q), 120.0, 125.7, 130.2 (q), 136.8, 139.9 (q), 140.7 (q), 142.6 (q), 144.9 (q), 145.7 (q), 160.4 (q) ppm). UV/Vis (CH_2Cl_2): λ_{max} (log ε) = 439 (5.53), 554 (4.70), 588 nm (4.62). HRMS (m/z)-APCI: (M+H) Calcd for $(C_{88}H_{85}N_4O_4Ni)^+$: 1319.5919; Found 1319.5926. IR (ATR): ṽ = 2963.8, 2867.8, 1599.8, 1446.8, 1294.9, 1210.7, 1169.8, 1017.7, 854.9, 779.9, 694.2 cm^{-1}.

[2,3,7,8,12,13,17,18-Octaethyl-5,10,15,20-tetrakis(3-methoxy-3'-biphenyl)porphyrinato]nickel(II) (45)

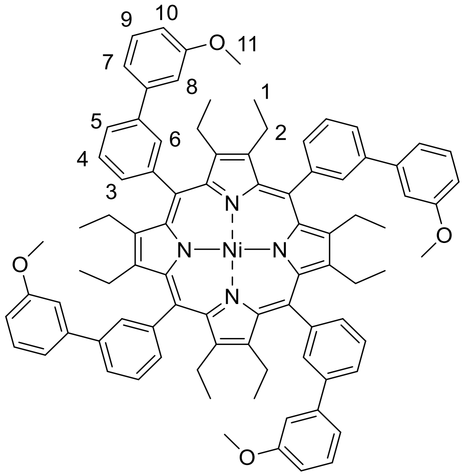

The title compound **45** was synthesized by general procedure A. The crude mixture was purified by column chromatography (SiO_2, DCM/hexane 50:50, v/v, R_f 0.3). The solvent was removed at reduced pressure. Compound **35** was then redissolved in the minimum amount of DCM and recrystallized by layering with hexane. The product was isolated via gravity filtration as purple crystals, as a rotameric mixture (19 mg, 31%) mp > 300 ˚C (from hexane). ^1H NMR ($CDCl_3$, 400 MHz): δ = 0.59 (m, 24H, H-1), 2.04-2.53 (appbrs, 16H, H-2), 3.87 (m, 12H, H-11), 6.96 (m, 4H, H-9), 7.31 (m, 4H, H-8), 7.36-7.45 (m, 8H, H-7/10), 7.68 (m, 4H, H-6), 7.91 (dd, J = 7.9, 1.6 Hz, 4H, H-5), 8.09 (m, 4H, H-3), 8.33 ppm (m, 4H, H-4). ^{13}C NMR ($CDCl_3$, 100MHz): δ_c = 17.3, 19.9, 55.6, 113.1, 117.1 (q), 120.1, 127.1, 127.7, 130.1, 132.5, 133.3, 139.7 (q), 140.7 (q), 142.9 (q), 144.6 (q), 145.8 (q) 160.3 ppm (q). UV/Vis (CH_2Cl_2): λ_{max} (log ε) = 435 (5.51), 552 (4.66), 587 nm (4.61). HRMS (m/z)-APCI: (M+H) Calcd for $(C_{88}H_{85}N_4O_4Ni)^+$: 1319.5919; Found 1319.5917. IR (ATR): ṽ = 2964.8, 2867.8, 1574.8, 1463.8, 1285.8, 1166.8, 1040.8, 944.6, 851.8, 773.2, 696.7 cm^{-1}.

[2,3,7,8,12,13,17,18-Octaethyl-5,10,15,20-tetrakis(4-(4,4,5,5-tetramethyl-1,3,2-dioxaborolan-2-yl)phenyl)porphyrinato] nickel(II) (46)

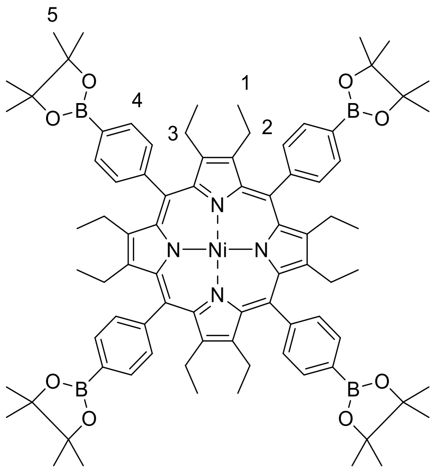

[5,10,15,20-Tetrakis(4-bromophenyl)-2,3,7,8,12,13,17,18-octaethylporphyrinato]nickel(II) (100 mg, 0.08 mmol), bis(pinacolato)diboron (4.19 g, 16.5 mmol), NaOAc (542 mg, 6.6 mmol), and Pd(dppf)Cl_2 (96.25 mg, 0.13 mmol) were dissolved in DMF (40 mL) under argon. The solution was heated to 100 °C and allowed to react while stirring for 16 h. After cooling, DCM (70 mL) was added, and the mixture was washed with brine (3 × 70 mL). The organic layers were combined and dried over $MgSO_4$, and the solvent was evaporated at reduced pressure. The remaining purple solid was dissolved in DCM and purified using silica gel column chromatography (SiO_2, DCM/hexane 3:1, v/v, R_f 0.75). The product was then dissolved in hexane and cooled to −20 °C for 16 h, the solids that precipitated were filtered then recrystallized in MeOH to yield purple crystals (34 mg, 30%) mp > 300 ˚C (from methanol). ^{1}H NMR (400 MHz, $CDCl_3$): δ = 0.49 (t, J = 7.3 Hz, 24H, H-1), 1.47 (s, 48H, H-5), 1.95-2.50 (appbrs, 16H, H-2), 8.00 (d, J = 7.5 Hz, 8H, H-3), 8.08 (d, J = 7.5 Hz, 8H, H-4) ^{11}B NMR (128 MHz, $CDCl_3$): δ 30.7 ppm. ^{13}C NMR (101 MHz, $CDCl_3$): δ_c = 17.2, 19.8, 25.1, 31.3, 84.2, 133.5, 133.9, 143.1 ppm. HRMS (m/z)-APCI: (M+H) Calcd for $(C_{84}H_{105}B_4N_4O_8Ni)^+$: 1399.7695; Found 1399.7622. UV/Vis (CH_2Cl_2): λ_{max} (log ε) = 439 (5.50), 554 (4.69), 590 nm (4.64). IR (ATR): ṽ = 2972.9, 2927.9, 2870.9, 1606.9, 1450.9, 1357.9, 1260.9, 1141.9, 1089.9, 1021.9, 960.9, 858.2, 812.2, 728.5, 657.7 cm^{-1}. Single

crystals of porphyrin **46** and bis(pinacolato)diboron suitable for XRD were grown at ambient temperature, in an NMR tube using approx. 5 mg of porphyrin with deuterated chloroform as solvent. Crystals grew on slow evaporation of chloroform. For details on XRD determination, see the refinement details section and Table S4**.** A neoplastic representation (Figure S150) and in-plane and out-of-plane skeletal images of the porphyrin (Figure S151) were generated and magnitudes of distortion (Table S11) were calculated using the NSD analyzer tool.

[2,3,7,8,12,13,17,18-Octaethyl-5,10,15,20-tetrakis(4-(pyren-1-yl)phenyl)porphyrinato]nickel(II) (47)

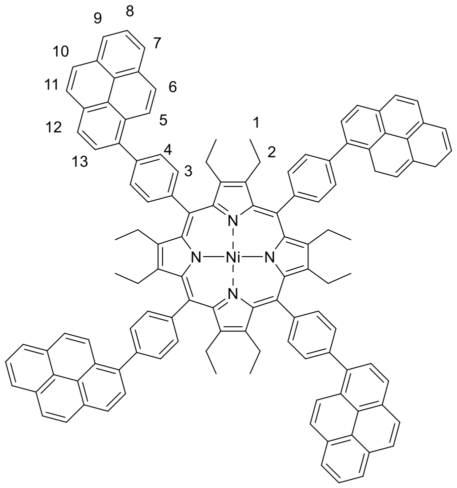

[2,3,7,8,12,13,17,18-Octaethyl-5,10,15,20-tetrakis(4-(4,4,5,5-tetramethyl-1,3,2-dioxaborolan-2-yl)phenyl)porphyrinato]nickel(II) (20 mg, 0.014 mmol), 1-bromopyrene (50.2 mg, 0.18 mmol), Cs_2CO_3 (116.3 mg, 0.36 mmol), $Pd_2(dba)_3$ (3.4 mg, 0.004 mmol), and SPhos (6.1 mg, 0.02 mmol) were added to a mixture of toluene/water 1:1 (30 mL, v/v) under argon. The biphasic mixture was heated to 85 °C and stirred for 48 h. The aqueous and organic layers were separated, and the organic layer was washed with brine (3 × 40 mL), dried over $MgSO_4$, the solvent was evaporated at reduced pressure, and the product was purified using column chromatography (SiO_2, DCM/hexane 3:7, v/v, R_f 0.55).

The solvent was removed under reduced pressure. Compound **47** was then redissolved in the minimum amount of DCM and layered with MeOH. The product was isolated via gravity filtration as a purple powder (8.6 mg, 36%) mp > 300 ˚C (from methanol). ^{1}H NMR (400 MHz, $CDCl_3$): δ = 0.84 (t, J = 7.3 Hz, 24H, H-1), 2.38-2.51 (appbrs, 16H, H-2), 7.96 (d, J = 7.9 Hz, 8H, H-3), 8.09 (t, J = 7.6 Hz, 4H, H-8), 8.21 (m, 12H, H-5/11/13), 8.28 (m, 8H, H-4), 8.31 (d, J = 7.7Hz, 4H, H-7), 8.40 (d, J = 7.8 Hz, 12H, H-6/10/12), 8.50 (d, J = 7.7 Hz, 4H, H-9). ^{13}C NMR (101 MHz, $CDCl_3$): 17.5, 20.2, 125.0, 126.2, 125.4, 125.5 (q), 126.3 (q), 127.8, 128.0, 128.1 (q), 128.9 (q), 129.5, 131.0 (q), 131.3 (q), 131.8 (q), 134.5, 137.9 (q), 139.4 (q), 141.4 (q), 145.1 (q), 146.0 (q) ppm. HRMS (m/z)-LIFDI: (M+H) Calcd for $(C_{124}H_{95}N_4Ni)^+$: 1696.6832; Found 1696.6655. UV/Vis (CH_2Cl_2): λ_{max} (log ε) = 437 (5.40), 554 (4.56), 591 nm (4.48). IR (ATR): ṽ = 2964.7, 2868.7, 1449.2, 1372.3, 1259.5, 1168.1, 1018.9, 775.8, 703.7 cm^{-1}.

[2,3,7,8,12,13,17,18-Octaethyl-5,10,15,20-tetrakis(phenyl-3-pyrid-4'-yl) porphyrinato]nickel(II) (42)

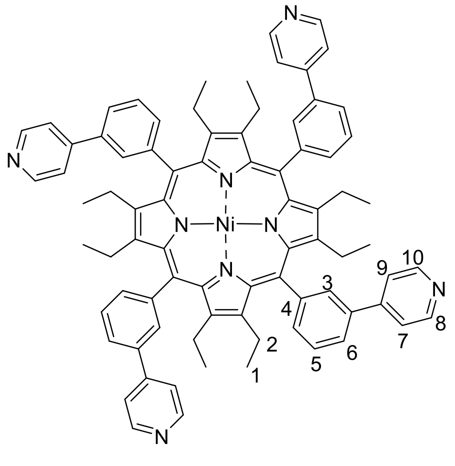

The title compound **42** was synthesized by general procedure A. The crude mixture was purified by column chromatography (SiO_2, DCM/MeOH/TEA 90:9:1, v/v, R_f 0.5). The solvent was removed at reduced pressure. Compound **42** was then redissolved in the

minimum amount of DCM and layered with hexane. The product was isolated via gravity filtration as a purple powder, as a rotameric mixture (27 mg, 30%) mp > 300 ˚C (from hexane). ^{1}H NMR ($CDCl_3$, 400 MHz): δ = 0.56 (t, J = 7.3 Hz, 24H, H-1), 1.97-2.59 (appbrs, 16H, H-2), 7.67 (dd, J = 5.8, 3.5 Hz, 8H, H-7/9), 7.75 (ddt, J = 7.7, 5.6, 3.1 Hz, 4H, H-5), 7.97 (d, J = 7.8 Hz, 4H, H-6), 8.18 (m, 4H, H-4), 8.36 (m, 4H, H-3), 8.72 ppm (d, J = 5.9 Hz, 8H, H-8/10). ^{13}C NMR (101 MHz, $CDCl_3$): $δ_C$ = 17.2, 19.9, 116.7 (q), 122.0, 127.1, 128.2, 132.6, 132.8, 136.9 (q), 137.0 (q), 141.1 (q), 144.6 (q), 145.9 (q), 148.4 (q), 150.7. ppm. UV/Vis (CH_2Cl_2): $λ_{max}$ (log ε) = 435 (5.33), 556 (4.51), 588 nm (4.48). HRMS (m/z)-APCI: (M+H) Calcd for $(C_{80}H_{73}N_8Ni)^+$: 1203.5306; Found 1203.5301. IR (ATR): ṽ = 3048.8, 2965.7, 2868.7, 1593.7, 1443.7, 1353.7, 1257.8, 1167.7, 1021.6, 952.8, 880.6, 786.8, 731.5 cm^{-1}.

[2,3,7,8,12,13,17,18-Octaethyl-5,10,15,20-tetrakis(phenyl-4-pyrid-4’-yl)porphyrinato]nickel(II) (32)

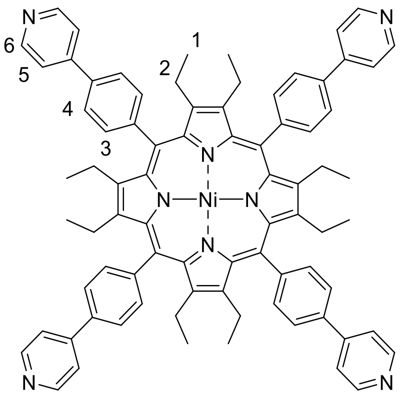

The title compound **32** was synthesized by general procedure A. The crude mixture was purified by column chromatography (SiO_2, DCM/MeOH/TEA 90:9:1, v/v, R_f 0.4). The solvent was removed at reduced pressure. Compound **32** was then redissolved in the minimum amount of DCM and layered with hexane. The product was isolated via gravity filtration as a purple powder (14 mg, 47%) mp > 300 ˚C (from hexane). ^{1}H NMR ($CDCl_3$,

400 MHz): δ = 0.56 (t, J = 7.4 Hz, 24H, H-1), 3.10 (q, J = 7.3 Hz, 8H, H-2), 3.60 (q, J = 7.3 Hz, 8H), 7.79 (d, J = 5.0 Hz, 8H, H-5), 7.95 (d, J = 7.8 Hz, 8H, H-4), 8.23 (d, J = 7.7 Hz, 8H, H-3), 8.79 ppm (d, J = 5.0 Hz, 8H). ^{13}C NMR (101 MHz, $CDCl_3$): δ_c = 17.2, 20.0, 116.7 (q), 121.9, 123.1 (q), 125.7, 135.2, 137.9 (q), 141.1 (q), 144.8 (q), 145.8 (q), 148.1 (q), 150.7 (q) ppm. UV/Vis (CH_2Cl_2): λ_{max} (log ε) = 439 (5.27), 556 (4.45), 587 nm (4.39). HRMS (m/z)-APCI: (M+H) Calcd for $(C_{80}H_{73}N_8Ni)^+$: 1203.5306; Found 1203.5309. IR (ATR): ṽ = 2965.8, 2868.1, 1448.4, 1371.8, 1259.2, 1168.1, 1068.2, 1012.5, 863.8, 800.9 cm^{-1}.

[2,3,7,8,12,13,17,18-Octaethyl-5,10,15,20-tetrakis(3-4’-triphenyl)porphyrinato]nickel(II) (38)

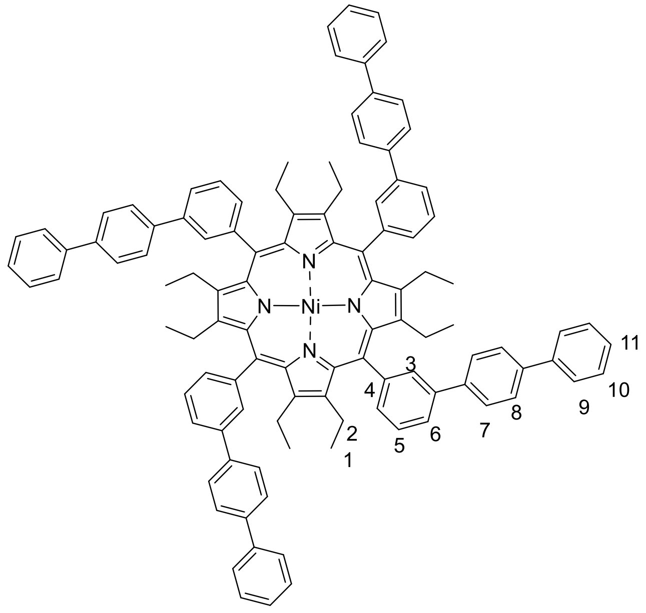

The title compound **38** was synthesized by general procedure A. The crude mixture was purified by column chromatography (SiO_2, DCM/hexane 60:40 v/v, R_f 0.6). The solvent was removed at reduced pressure. Compound **32** was then redissolved in the minimum amount of DCM and layered with MeOH. The product was isolated via gravity filtration as a purple powder, as a rotameric mixture (8 mg, 7%) mp > 300 ˚C (from methanol). ^{1}H NMR

($CDCl_3$, 400 MHz): δ = 0.60 (appbrs, 24H, H-1), 2.05-2.56 (appbrs, 16H, H-2), 7.37 (m, 4H, H-11), 7.46 (m, 8H, H-10), 7.68-7.74 (m, 20H, H-5/H-8/H-9, overlapping), 7.87 (m, 8H, H-7), 7.95 (d, J = 7.9 Hz, 4H, H-6), 8.09 (m, 4H, H-4), 8.39 (m, 4H, H-3) ppm. ^{13}C NMR (101 MHz, $CDCl_3$): δ_c = 17.3, 19.9, 117.1, 126.9, 127.3, 127.6, 127.8, 127.9, 129.0, 132.9, 133.5, 139.3, 140.1, 140.2, 140.4, 140.6, 140.9, 144.3, 145.8 ppm. UV/Vis (CH_2Cl_2): λ_{max} (log ε) = 435 (4.96), 549 (4.41), 585 nm (4.39). HRMS (m/z)-APCI: (M+H) Calcd for $(C_{108}H_{93}N_4Ni)^+$: 1503.6748; Found 1503.6719. IR (ATR): ṽ = 2963.1, 2868.4, 1447.2, 1372.1, 1258.8, 1017.7, 863.5, 792.2, 685.9 cm^{-1}.

[2,3,7,8,12,13,17,18-Octaethyl-5,10,15,20-tetrakis(4-phenyl-3'-thiophenyl)porphyrinato]nickel(II) (33)

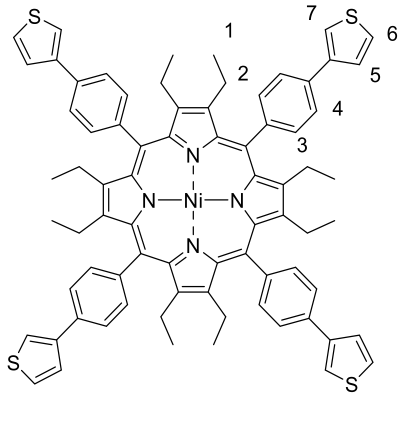

The title compound **33** was synthesized by general procedure A. The crude mixture was purified by column chromatography (SiO_2, DCM/hexane 50:50, v/v, R_f 0.3). The solvent was removed at reduced pressure. Compound **33** was then redissolved in the minimum amount of DCM and layered with hexane. The product was isolated via gravity filtration as a purple powder (17 mg, 56%) mp > 300 ˚C (from hexane). ^1H NMR ($CDCl_3$, 400 MHz): δ = 0.56 (t, J = 7.3 Hz, 24H, H-1), 2.16-2.56 (appbrs, 16H, H-2), 7.53 (dd, J = 5.0, 2.9 Hz, 4H, H-5), 7.68 (dd, J = 5.0, 1.3 Hz, 4H, H-6), 7.75 (dd, J = 2.9, 1.3 Hz, 4H, H-7), 7.89 (d, J = 8.1 Hz, 8H, H-3), 8.12 ppm (d, J = 8.1 Hz, 8H, H-4). ^{13}C NMR (101 MHz, $CDCl_3$): δ_c =

17.3, 20.0, 116.8 (q), 120.8, 125.0, 126.6, 126.7, 134.9, 135.4 (q), 139.2 (q), 142.3 (q), 144.9 (q), 145.7 (q) ppm. UV/Vis (CH_2Cl_2): λ_{max} (log ε) = 439 (5.27), 554 (4.52), 587 nm (4.46). HRMS (m/z)-APCI: (M+H) Calcd for $(C_{76}H_{69}N_4S_4Ni)^+$: 1223.3753; Found 1223.3732. IR (ATR): ṽ = 2962.9, 2867.5, 1448.4, 1352.9, 1259.7, 1166.8, 1019.1, 882.5, 774.9 cm^{-1}. Single crystals of porphyrin **33** were grown at ambient temperature in an NMR tube using approx. 0.5–1 mg of porphyrin in hexane and slow evaporation. For details on XRD determination, see the refinement details section and Table S4. A neoplastic representation (Figure S146) and in-plane and out-of-plane skeletal images of the porphyrin (Figure S147) were generated and magnitudes of distortion (Table S10) were calculated using the NSD symmetry tool.

[2,3,7,8,12,13,17,18-Octaethyl-5,10,15,20-tetrakis(3-phenyl-3'-thiophenyl)porphyrinato]nickel(II) (43)

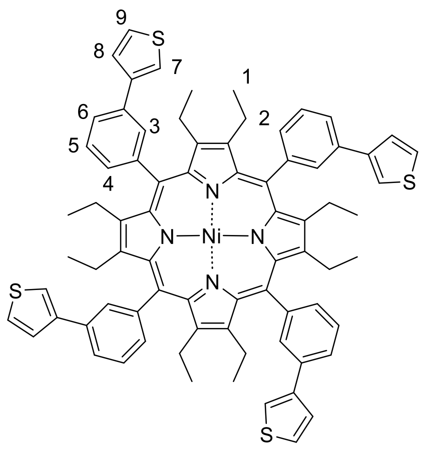

The title compound **43** was synthesized by general procedure A. The crude mixture was purified by column chromatography (SiO_2, DCM/hexane 50:50, v/v, R_f 0.6). The solvent was removed at reduced pressure. Compound **43** was then redissolved in the minimum amount of DCM and layered with hexane. The product was isolated via gravity filtration as purple crystals, as a rotameric mixture (9 mg, 10%) mp > 300 ˚C (from hexane). ^{1}H NMR

($CDCl_3$, 400 MHz) δ = 0.57 (t, J = 7.1 Hz, 24H, H-1), 2.05-2.50 (appbrs, 16H, H-2), 7.46 (m, 4H, H-8), 7.55 (m, 4H, H-9), 7.62 (m, 4H, H-5), 7.67 (m, 4H, H-7), 7.91 (dt, J = 7.9, 1.4 Hz, 4H, H-6), 8.03 (m, 4H, H-4), 8.33 ppm (m, 4H, H-3). ^{13}C NMR (101 MHz, $CDCl_3$) δ_c = 17.2, 19.9, 117.0 (q), 120.9, 126.4, 126.6, 126.8, 127.7, 132.3, 133.2, 134.5 (q), 140.8 (q), 142.5 (q), 144.6 (q), 145.9 (q) ppm. UV/Vis (CH_2Cl_2): λ_{max} (log ε) = 434 (5.12), 552 (4.23), 589 nm (4.19). HRMS (m/z)-APCI: (M+H) Calcd for $(C_{76}H_{69}N_4S_4Ni)^+$: 1223.3753; Found 1223.3723. IR (ATR): ṽ = 2968.7, 2968.6, 1449.4, 1371.5, 1257.1, 1167.3, 1054.3, 862.8, 773.9 cm^{-1}.

[2,3,7,8,12,13,17,18-Octaethyl-5,10,15,20-tetrakis(4-4'-methylbiphenyl)porphyrinato]nickel(II) (34)

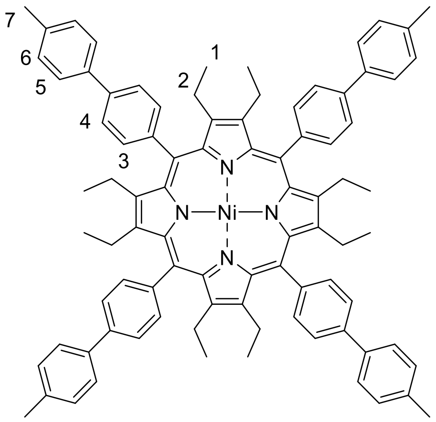

The title compound **34** was synthesized by general procedure A. The crude mixture was purified by column chromatography (SiO_2, DCM/hexane 25:75, v/v, R_f 0.2). The solvent was removed at reduced pressure. Compound **34** was then redissolved in the minimum amount of DCM and layered with hexane. The product was isolated via gravity filtration as purple crystals (8 mg, 25%) mp > 300 ˚C (from hexane). ^1H NMR ($CDCl_3$, 400 MHz): δ = 0.57 (t, J = 7.3 Hz, 24H, H-1), 2.09-2.51 (appbrs, 16H, H-2), 2.48 (s, 12H, H-7), 7.38 (d, J = 8.3 Hz, 8H, H-6), 7.79 (d, J = 8.3 Hz, 8H, H-5), 7.87 (d, J = 8.1 Hz, 8H, H-4), 8.14 ppm (d, J = 8.1 Hz, 8H, H-3). ^{13}C NMR (101 MHz, $CDCl_3$): δ_c = 17.3, 19.9, 21.4, 117.0 (q),

125.4, 127.3, 129.9, 134.8, 137.6 (q), 138.1 (q), 139.1 (q), 140.7 (q), 144.9 (q), 145.7 (q) ppm. UV/Vis (CH_2Cl_2): λ_{max} (log ε) = 439 (5.44), 556 (4.63), 588 nm (4.55). HRMS (m/z)-APCI: (M+H) Calcd for $(C_{88}H_{85}N_4Ni)^+$: 1255.6122; Found 1255.6127. IR (ATR): ṽ = 2962.9, 2867.9, 1448.9, 1352.9, 1260.9, 1167.9, 1019.9, 862.5 cm^{-1}.

[2,3,7,8,12,13,17,18-Octaethyl-5,10,15,20-tetrakis(4-methyl-3'-biphenyl)porphyrinato]nickel(II) (44)

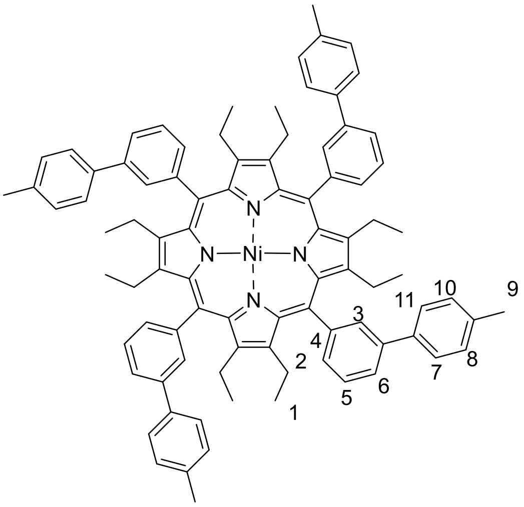

The title compound **44** was synthesized by general procedure A. The crude mixture was purified by column chromatography (SiO_2, DCM/hexane 50:50, v/v, R_f 0.6). The solvent was removed at reduced pressure. Compound **44** was then redissolved in the minimum amount of DCM and layered with MeOH. The product was isolated via gravity filtration as purple crystals, as a rotameric mixture (33 mg, 30%) mp > 300 ˚C (from methanol). ^1H NMR ($CDCl_3$, 400 MHz): δ = 0.61 (t, J = 7.3 Hz, 24H, H-1), 2.05-2.62 (appbrs, 16H, H-2), 2.46 (s, 12H, H-9), 7.34 (d, J = 8.0 Hz, 8H, H-8/10), 7.68 (m, H4, H-6), 7.73 (m, 8H, H-7/11), 7.92 (d, J = 7.9 Hz, 4H, H-5), 8.08 (m, 4H, H-4), 8.35 ppm (m, 4H, H-3). ^{13}C NMR (101 MHz, $CDCl_3$): δ_c = 17.3, 19.9, 21.4, 117.1 (q), 126.8, 127.5, 127.6, 129.9, 132.9, 133.0, 137.5 (q), 138.5 (q), 139.7 (q), 140.8 (q), 144.6 (q), 145.8 (q) ppm. UV/Vis (CH_2Cl_2): λ_{max} (log ε) = 435 (5.34), 552 (4.43), 587 nm (4.38). HRMS (m/z)-APCI: (M+H) Calcd for

$(C_{88}H_{85}N_4Ni)^+$: 1255.6122; Found 1255.6115. IR (ATR): ṽ = 2961.4, 2866.3, 1598.6, 1515.5, 1449.6, 1169.2, 1020.7, 879.1, 783.4, 704.9 cm^{-1}.

[2,3,7,8,12,13,17,18-Octaethyl-5,10,15,20-tetrakis(phenyl-4-benzothiophen-2'-yl)porphyrinato]nickel(II) (31)

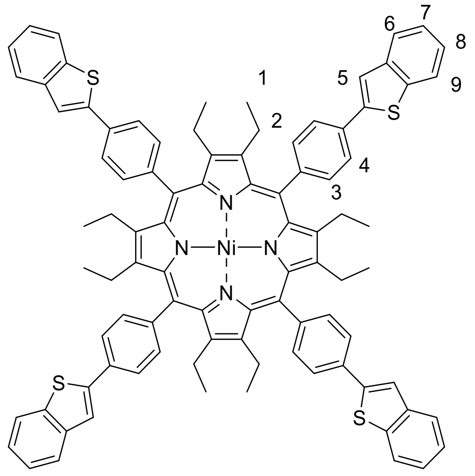

The title compound **31** was synthesized by general procedure A. The solvent was removed at reduced pressure. The crude mixture was purified by column chromatography (SiO_2, DCM/hexane 50:50, v/v, R_f 0.5). Compound **31** was then redissolved in the minimum amount of DCM and layered with MeOH. The product was isolated via gravity filtration as a purple powder (7 mg, 16%) mp > 300 ˚C (from methanol). ^1H NMR ($CDCl_3$, 400 MHz): δ = 0.59 (t, J = 7.4 Hz, 24H, H-1), 2.13-2.52 (appbrs, 16H, H-2), 7.40 (m, 8H, H-7/8), 7.83 (s, 4H, H-5), 7.88 (dd, J = 7.0, 1.4 Hz, 4H, H-6), 7.93 (dd, J = 7.8, 1.3 Hz, 4H, H-9), 8.01 (d, J = 8.2 Hz, 8H, H-4), 8.16 ppm (d, J = 8.2 Hz, 8H, H-3). ^{13}C NMR (101 MHz, $CDCl_3$): δ_c = 17.3, 20.0, 116.8 (q), 120.2, 122.6, 123.9, 124.8, 124.9, 125.1 (q), 134.2 (q), 135.0, 139.8 (q), 140.3 (q), 141.0 (q), 144.3 (q), 144.9 (q), 145.8 (q) ppm. UV/Vis (CH_2Cl_2): λ_{max} (log ε) = 434 (5.49), 557 (4.70), 590 nm (4.62). HRMS (m/z)-APCI: (M+H) Calcd for $(C_{92}H_{77}N_4S_4Ni)^+$: 1423.4379; Found 1423.4365. IR (ATR): ṽ = 2962.9, 2867.8, 2161.2, 2032.3, 1433.3, 1371.5, 1312.3, 1259.1, 1167.2, 1018.4, 792.1, 723.4 cm^{-1}.

[2,3,7,8,12,13,17,18-Octaethyl-5,10,15,20-tetrakis(phenyl-3-benzothiophen-2'-yl)porphyrinato]nickel(II) (41)

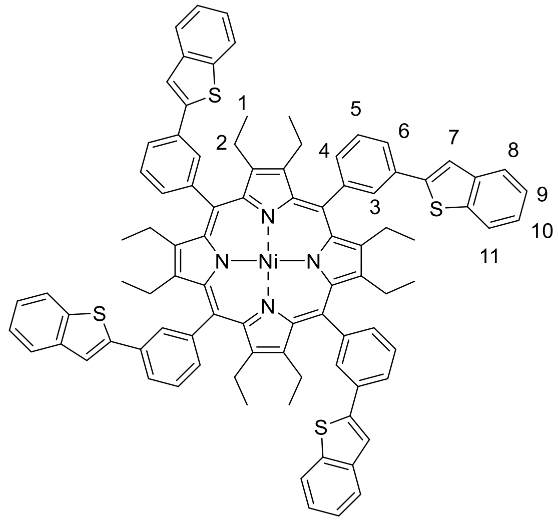

The title compound **41** was synthesized by general procedure A. The crude mixture was purified by column chromatography (SiO_2, DCM/hexane 50:50, v/v, R_f 0.3). The solvent was removed at reduced pressure. Compound **41** was then redissolved in the minimum amount of DCM and layered with MeOH. The product was isolated as a purple powder, as a rotameric mixture (3 mg, 4%) mp > 300 ˚C (from methanol). ^{1}H NMR ($CDCl_3$, 400 MHz): δ = 0.62 (t, J = 7.2 Hz, 24H, H-1), 2.16-2.51 (appbrs, 16H, H-2), 7.34 (m, 8H, H-9/10), 7.68-7.70 (m, 8H, H-5/7), 7.80 (m, 4H, H-8), 7.86 (m, 4H, H-11), 8.03 (dd, J = 8.0, 1.5 Hz, 4H, H-6), 8.12 (m, 4H, H-4), 8.47 ppm (m, 4H, H-3). ^{13}C NMR (101 MHz, $CDCl_3$): δ_C = 17.1, 20.0, 116.7 (q), 120.2, 122.6, 123.9, 124.7, 124.8 (q), 126.5, 127.9, 131.9, 134.1, 139.9 (q), 141.4 (q), 144.3 (q), 144.2 (q) ppm. UV/Vis (CH_2Cl_2): λ_{max} (log ε) = 436 (5.37), 551 (4.52), 590 nm (4.49). HRMS (m/z)-APCI: (M+H) Calcd for $(C_{92}H_{77}N_4S_4Ni)^+$: 1423.4379; Found 1423.4359. IR (ATR): ṽ = 2965.9, 2868.9, 1449.9, 1371.9, 1259.9, 1167.9, 1107.9, 1019.9, 862.8, 774.8 cm^{-1}.

[2,3,7,8,12,13,17,18-Octaethyl-5,10,15,20-tetrakis(3-phenyl-9-anthracenyl-porphyrinato] nickel(II) (37)

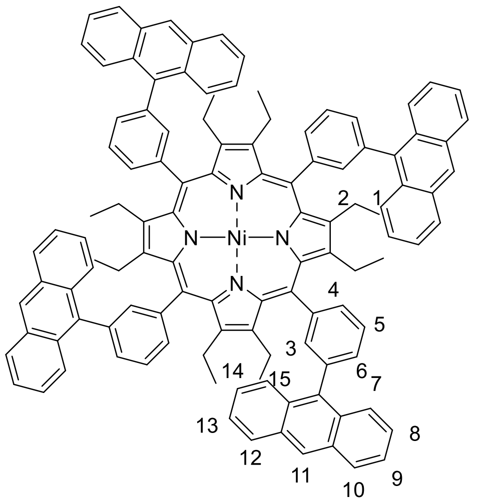

The title compound **37** was synthesized by general procedure A and purified by column chromatography (SiO_2, DCM/hexane 75:25, v/v, R_f 0.2). The product eluted as the first band from the column and the solvent was removed at reduced pressure. Compound **37** was then redissolved in the minimum amount of DCM and layered with MeOH. The product was isolated via gravity filtration as purple crystals (13 mg, 32%) mp > 300 ˚C (from methanol). NMR indicates the presence of the intercalated form at room temperature (see main manuscript) and not all NMR resonances could be assigned. ^{1}H NMR (C_6D_6, 600 MHz): δ = 0.53-74 (m, 24H, H-1), 2.40-3.02 (m, 16H, H-2), 6.94-8.41 ppm (52H, aromatic region, overlapping). ^{13}C NMR (101 MHz, $CDCl_3$): δ_c = 17.3, 20.0, 117.4, 125.3, 127.0, 128.4, 130.5, 131.2, 131.6, 133.2, 137.0, 138.0, 140.4, 145.3 ppm. HRMS (m/z)-LIFDI: (M+H) Calcd for $(C_{116}H_{93}N_4Ni)^+$: 1599.6709; Found 1599.6995. UV/Vis (CH_2Cl_2): λ_{max} (log ε) = 368 (4.58), 387 (4.55), 439 (5.00), 555 (4.36), 588 (4.33) nm. IR (ATR): ṽ = 3048.3, 2962.9, 2926.4, 2667.9, 1593.9, 1519.2, 1442.1, 1372.1, 1325.2, 1053.0, 1020.6, 925.8, 879.9, 840.8, 782.5, 731.1 cm^{-1}. Crystals of porphyrin **37** suitable for XRD were grown at ambient temperature in an NMR tube using approx. 5 mg of porphyrin with deuterated chloroform as solvent. Crystals grew on slow evaporation. For details on XRD determination, see the refinement details section and Table S4. A neoplastic

representation (Figure S157) and in- and out-of-plane skeletal images of the porphyrin (Figure S158) were generated and magnitudes of distortion (Table S13) were calculated using the NSD symmetry analyzer tool.

[2,3,7,8,12,13,17,18-Octaethyl-5,10,15,20-tetrakis(4-nitro-3'-biphenyl)porphyrinato]nickel(II) (40)

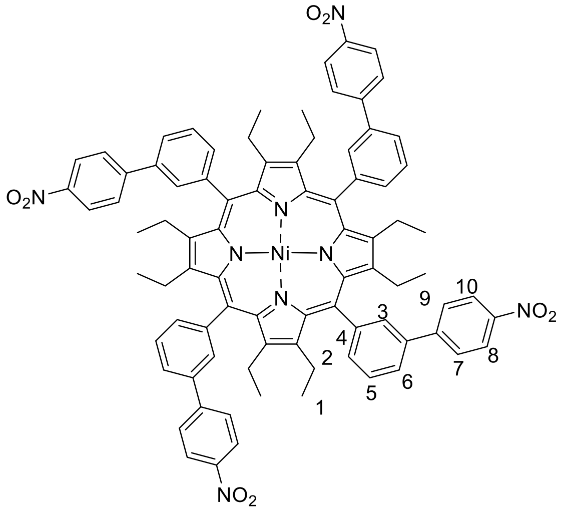

The title compound **40** was synthesized by general procedure A and purified by column chromatography (SiO_2, DCM/hexane 90:10, v/v, R_f 0.4). The product eluted as the second band from the column and the solvent was removed at reduced pressure. Compound **37** was then redissolved in the minimum amount of DCM and layered with MeOH. The product was isolated via gravity filtration as purple crystals as a rotameric mixture (21 mg, 18%) mp > 300ºC (from methanol). ^{1}H NMR ($CDCl_3$, 600 MHz): δ = 0.55 (t, J = 7.4 Hz, 24H, H-1), 1.97-2.55 (appbrs, 16H, H-2), 7.75 (m, 4H, H-6), 7.89-7.90 (m, 8H, H-7/9, overlapping), 7.95 (m, 4H, H-5), 8.15 (m, 4H, H-4), 8.33 (m, 4H, H-3, overlapping), 8.35-8.37 ppm (m, 8H, H-8/10, overlapping). ^{13}C NMR (101 MHz, $CDCl_3$): δ_c = 17.2, 19.8, 116.7 (q), 124.5 (q), 127.3, 127.5, 127.6, 128.2, 132.8, 133.1, 134.7, 137.6 (q), 141.1 (q), 144.5 (q), 145.8 (q), 147.4 (q) ppm. HRMS (m/z)-APCI: (M+H) Calcd for $(C_{84}H_{73}N_8O_8Ni)^+$:

1379.4899; Found 1379.4828. UV/Vis (CH_2Cl_2): λ_{max} (log ε) = 436 (5.09), 550 (4.56), 589 nm (4.54). IR (ATR): $\tilde{\nu}$ = 2970.9, 2871.9, 1596.9, 1519. 8, 1345.7, 1169.9, 1108.9, 1021.9, 854.8, 797.9, 751.6, 689.9 cm^{-1}.

^{1}H and $\{^{1}H\}^{13}$C NMR spectra:

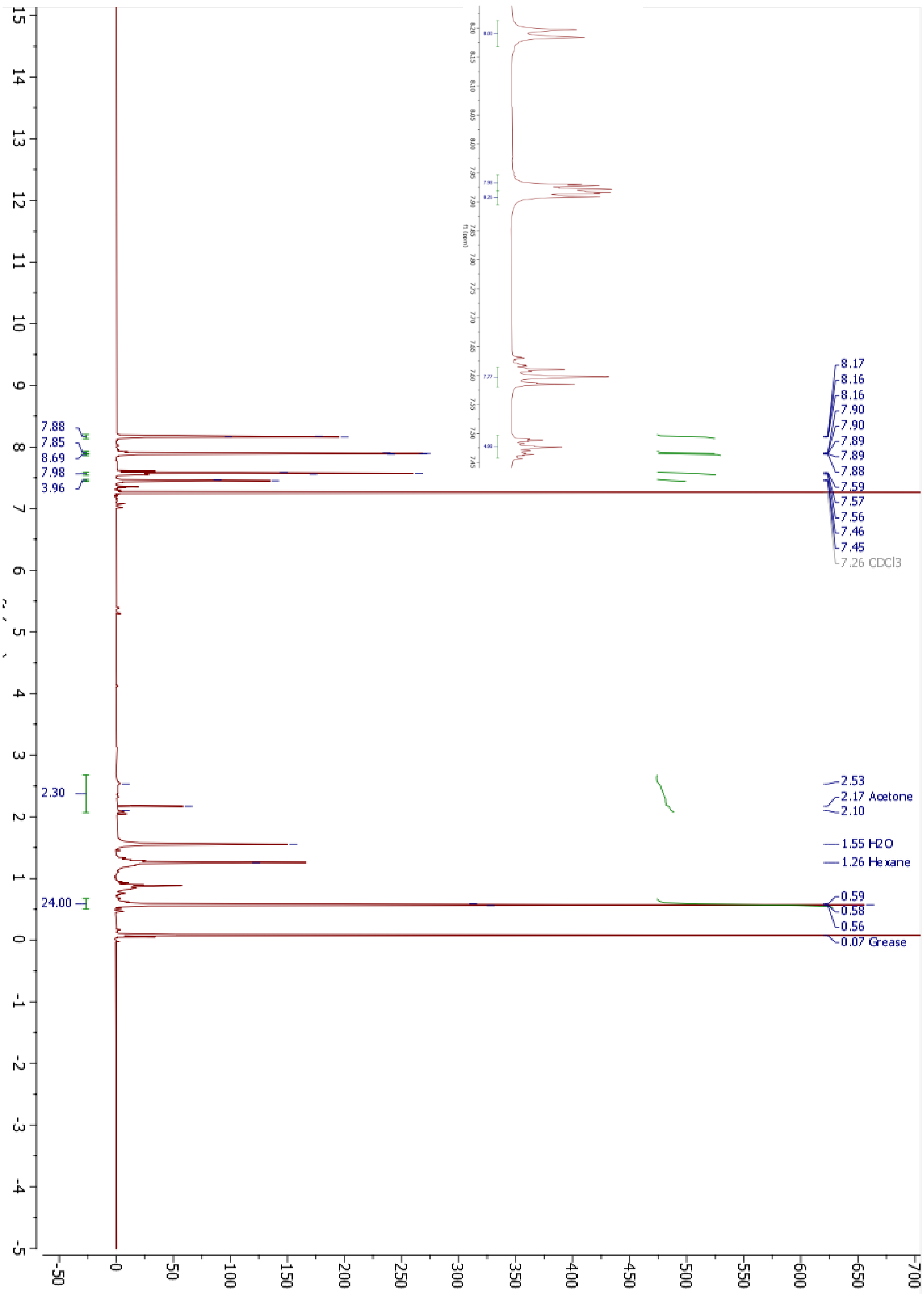

Figure S2: ^{1}H NMR ($CDCl_3$, 600 MHz) of porphyrin **26** including an embedded zoom of the aromatic region (δ = 8.70 – 7.40 ppm).

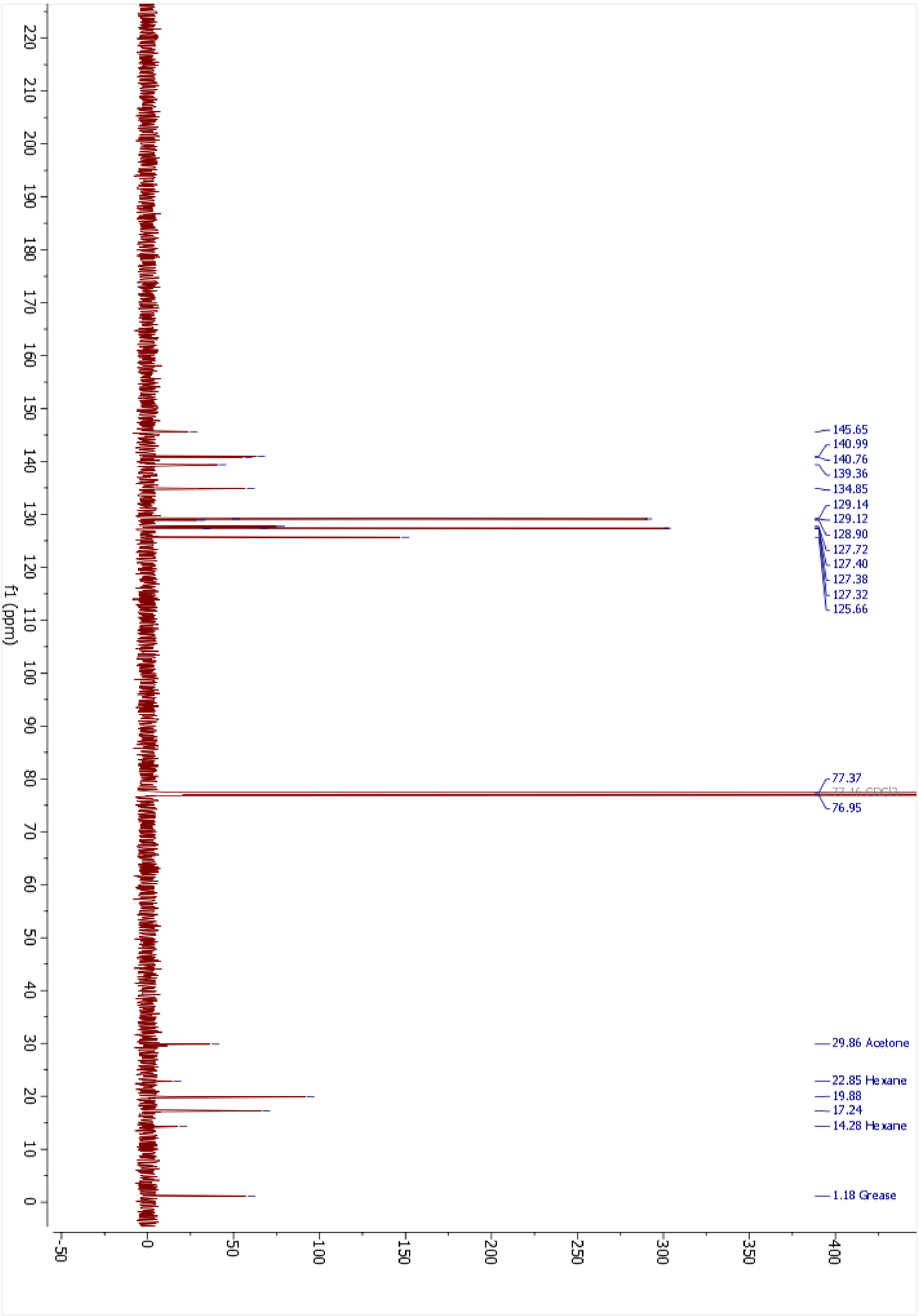

Figure S3: ^{13}C NMR ($CDCl_3$, 150 MHz) of porphyrin **26** in $CDCl_3$.

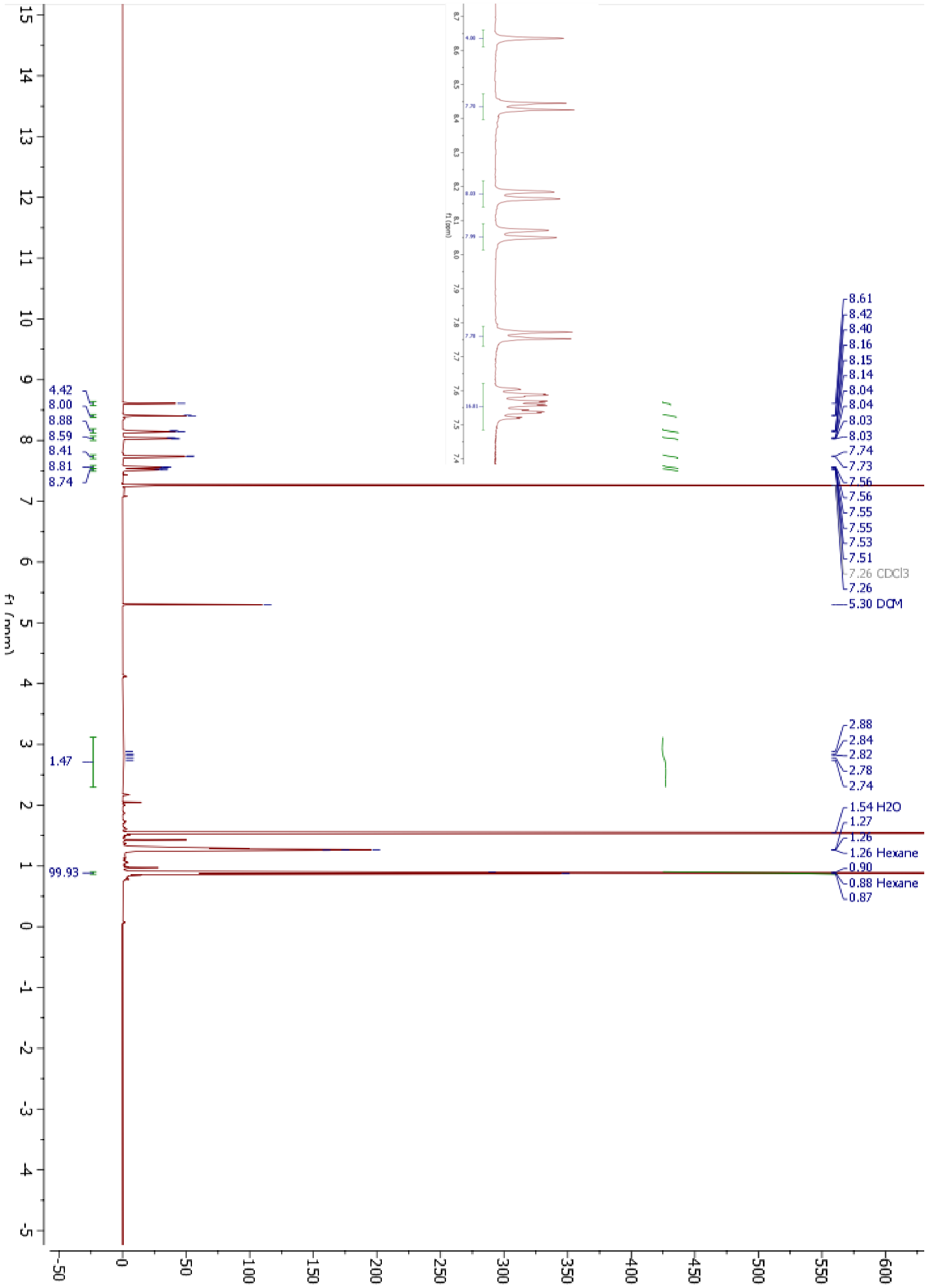

Figure S4: ^{1}H NMR ($CDCl_3$, 600 MHz) of porphyrin **27** in $CDCl_3$ including an embedded zoom of the aromatic region (δ = 8.70 – 7.40 ppm).

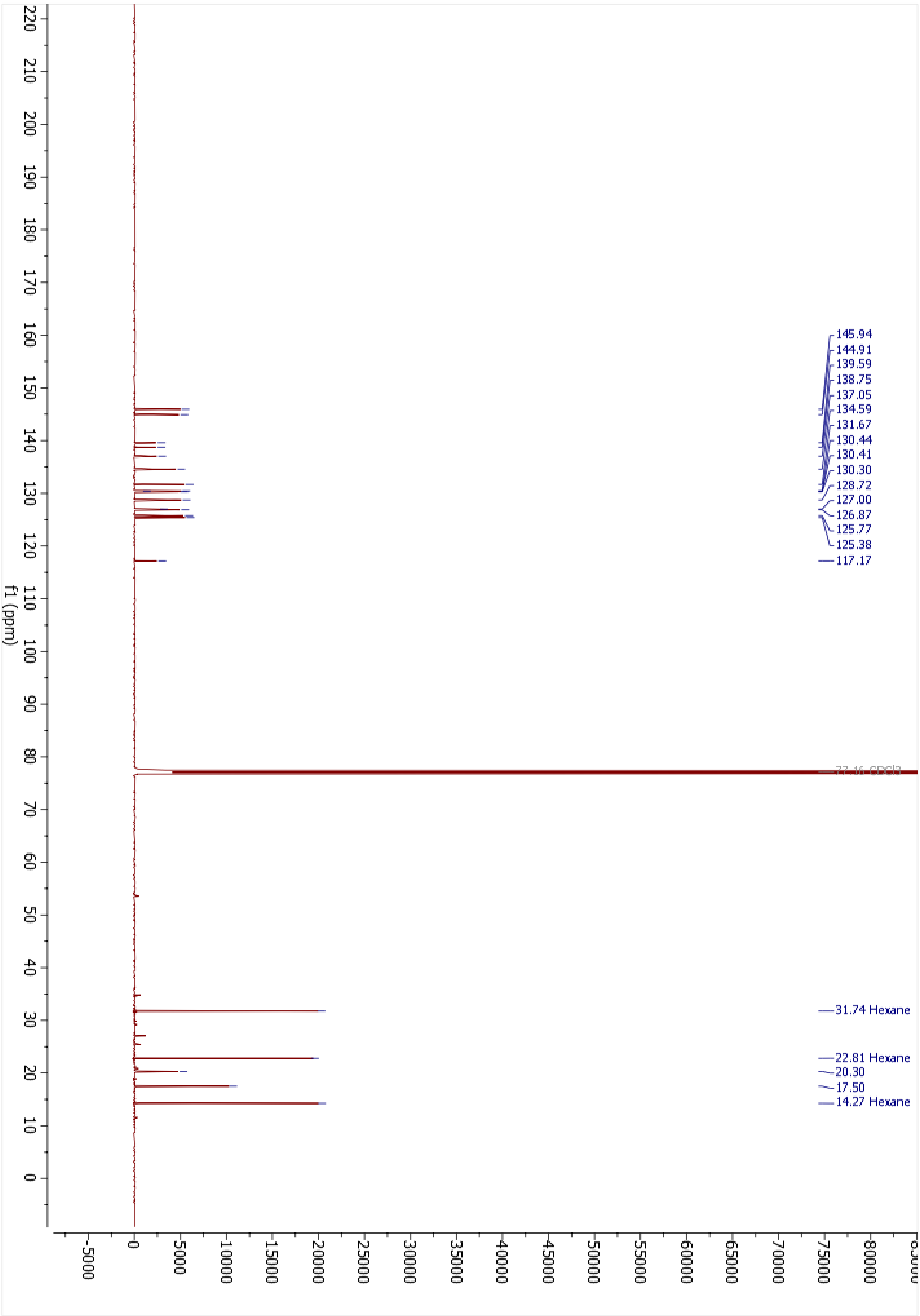

Figure S5: ^{13}C NMR ($CDCl_3$, 150 MHz) of porphyrin **27** in $CDCl_3$.

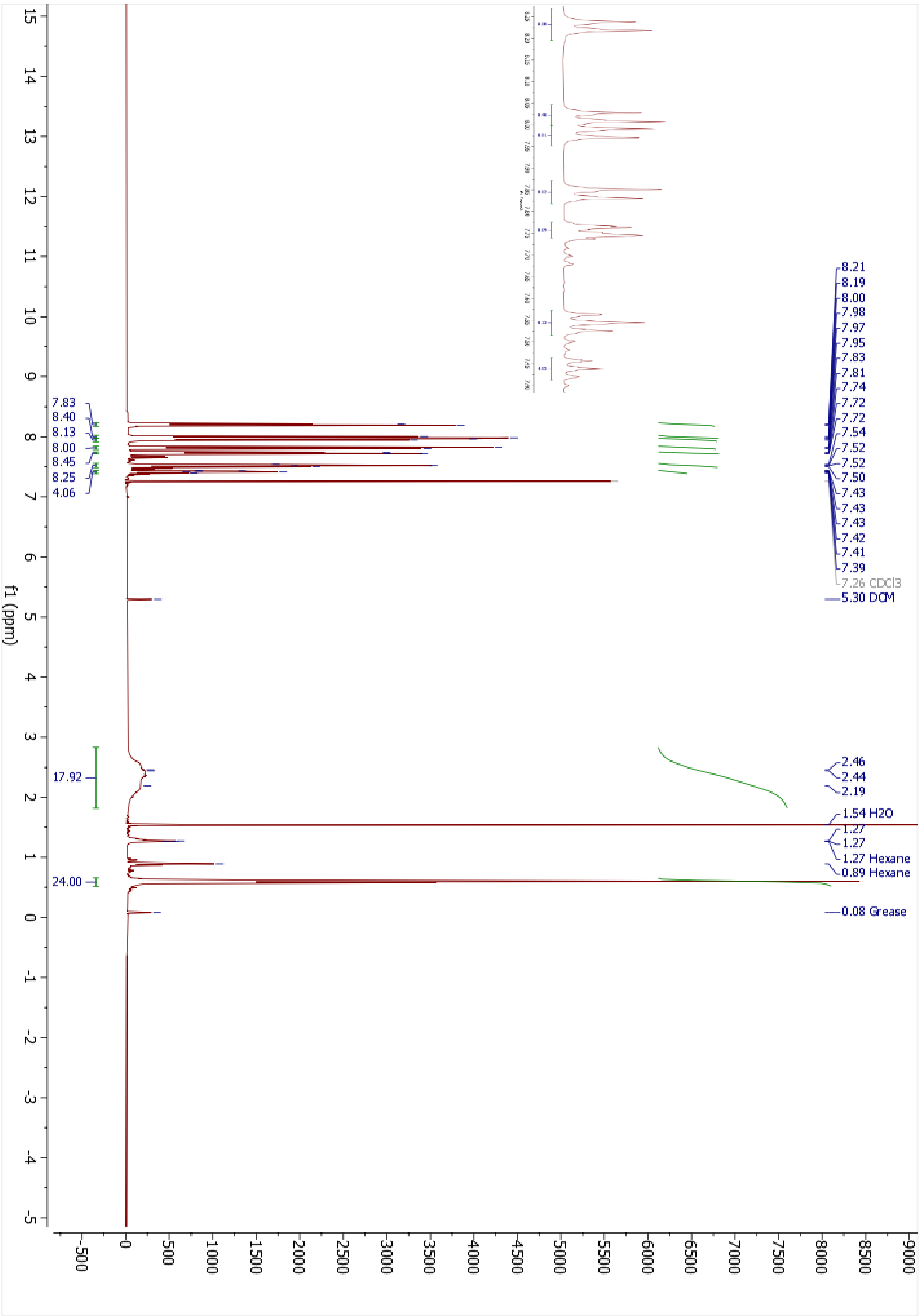

Figure S6: ^{1}H NMR ($CDCl_3$, 600 MHz) of porphyrin **28** including an embedded zoom of the aromatic region (δ = 8.25 – 7.40 ppm).

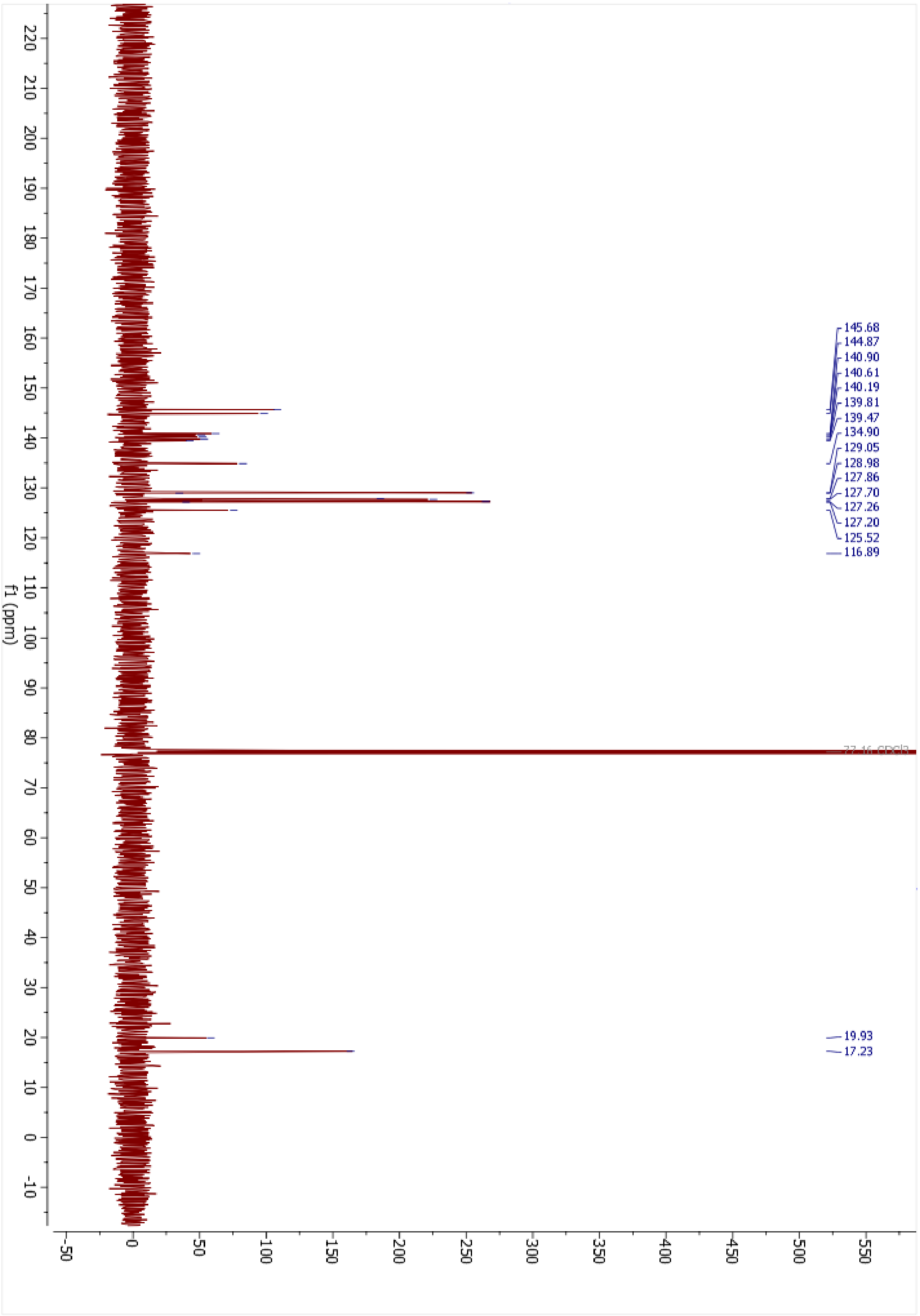

Figure S7: ^{13}C NMR ($CDCl_3$, 150 MHz) of porphyrin **28**.

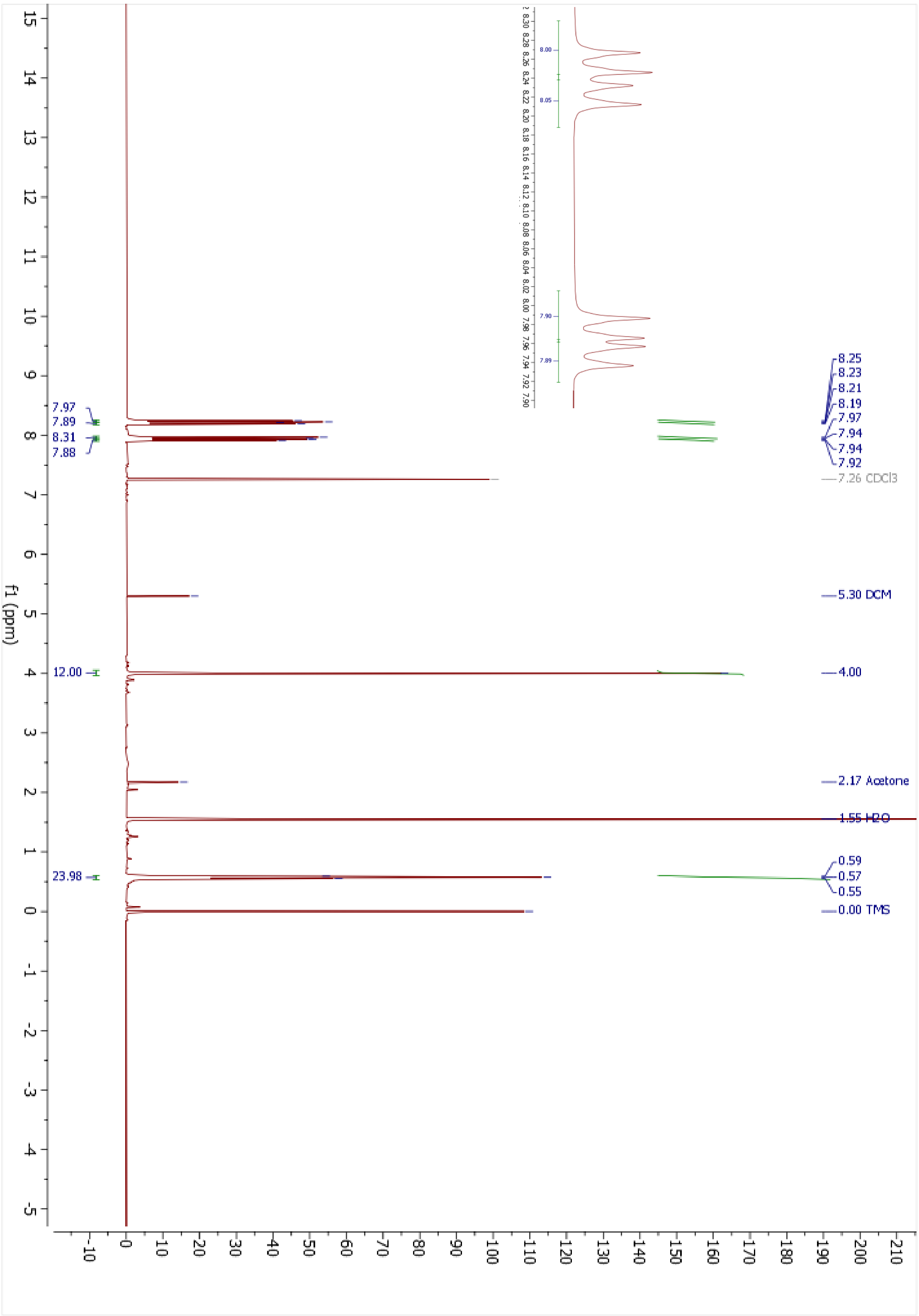

Figure S8: ^{1}H NMR ($CDCl_3$, 600 MHz) of porphyrin **29** including an embedded zoom of the aromatic region (δ = 8.30 – 7.90 ppm).

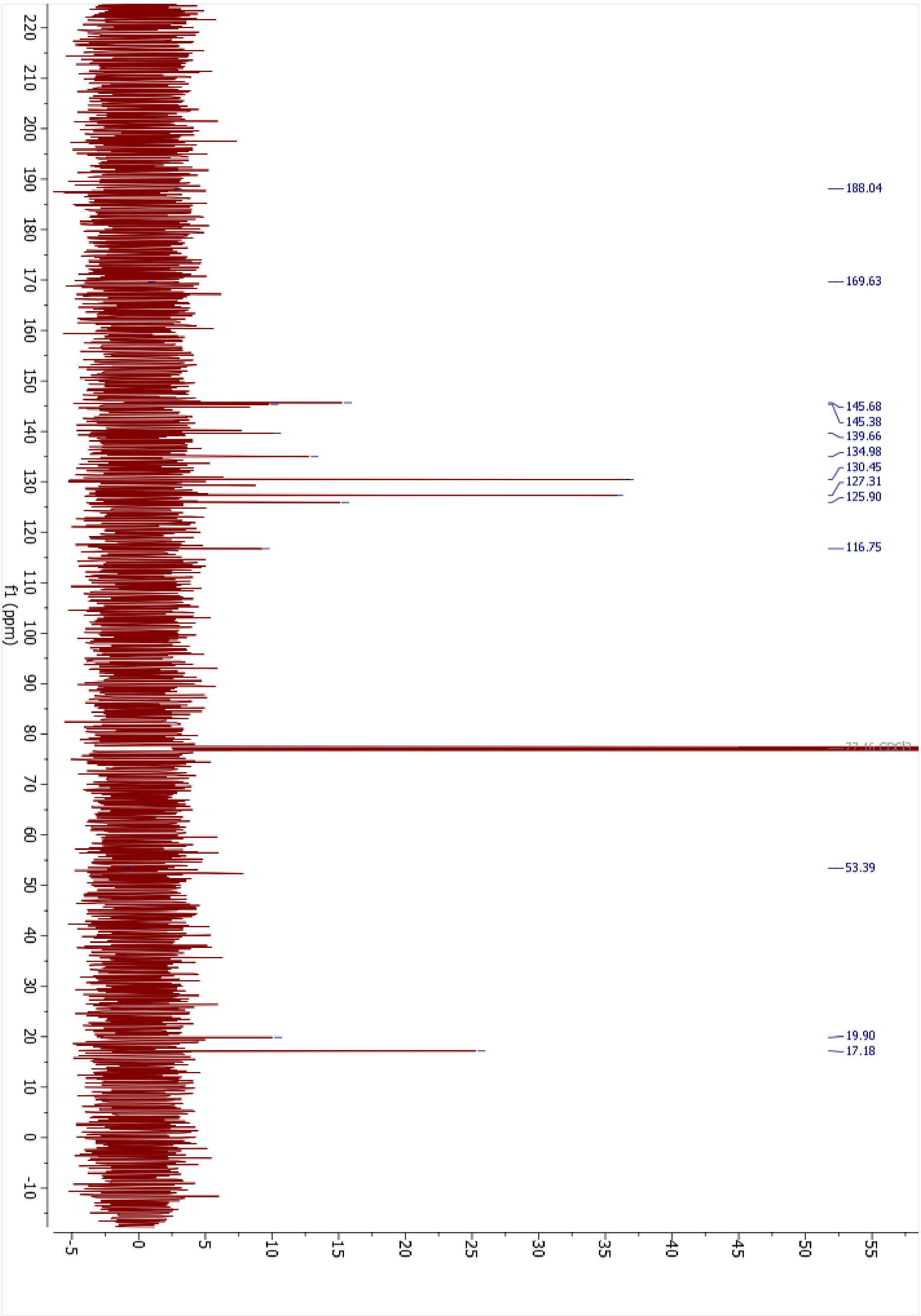

Figure S9: ^{13}C NMR ($CDCl_3$, 150 MHz) of porphyrin **29.**

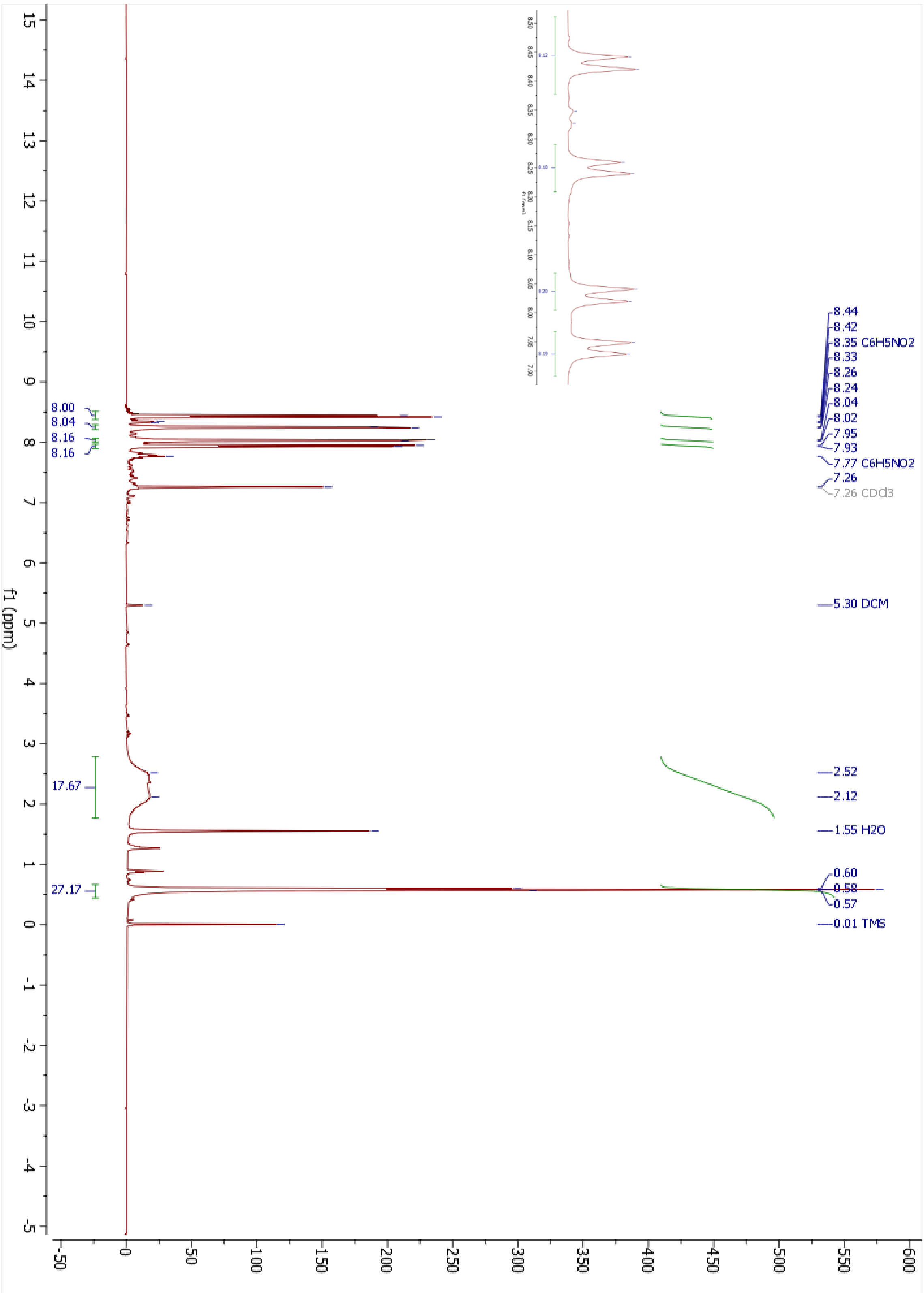

Figure S10: ^{1}H NMR ($CDCl_3$, 600 MHz) of porphyrin **30** including an embedded zoom of the aromatic region (δ = 8.50 – 7.90 ppm).

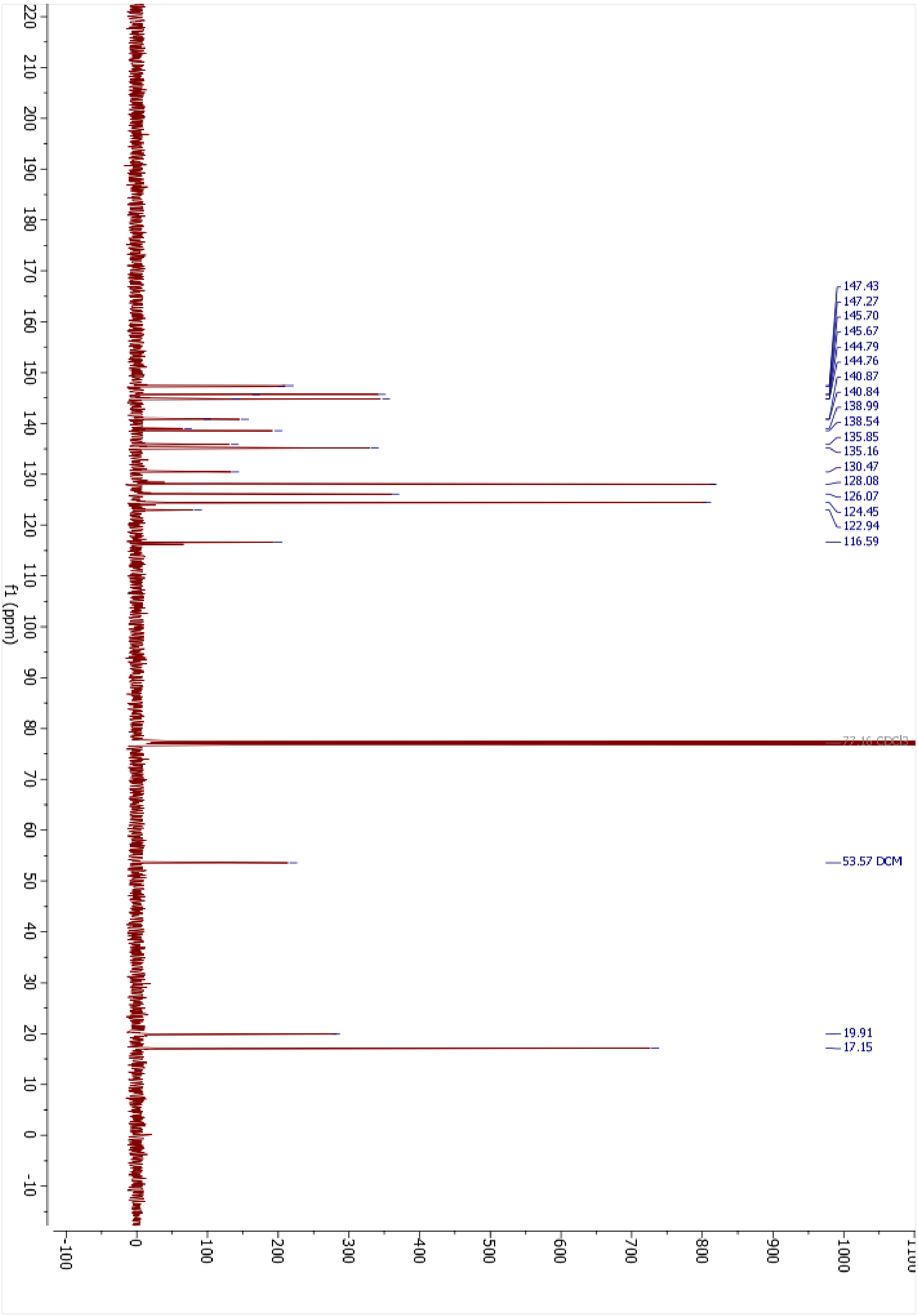

Figure S11: ^{13}C NMR ($CDCl_3$, 150 MHz) of porphyrin **30.**

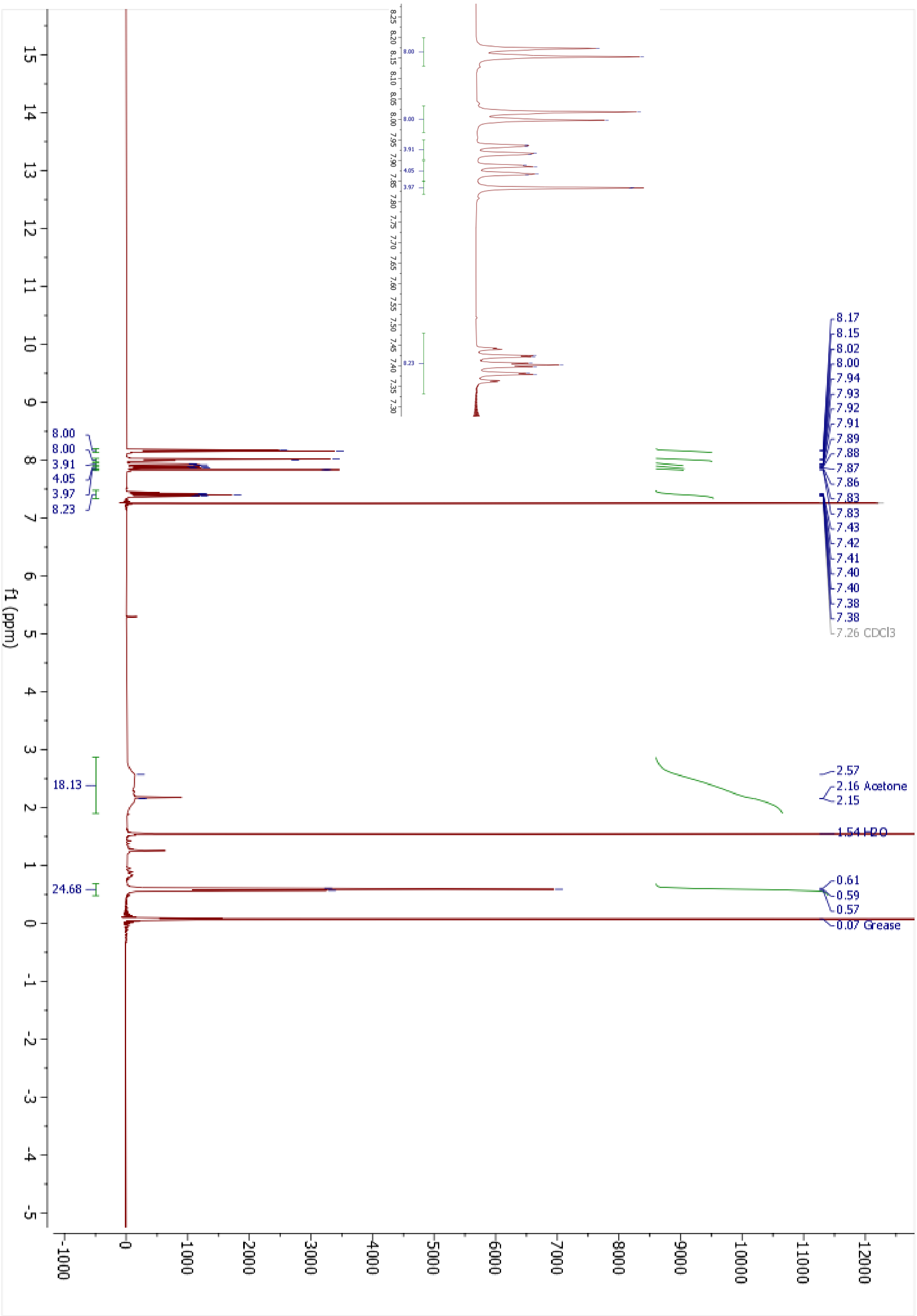

Figure S12: ^{1}H NMR ($CDCl_3$, 400 MHz) of porphyrin **31** including an embedded zoom of the aromatic region (δ = 8.30 – 7.30 ppm).

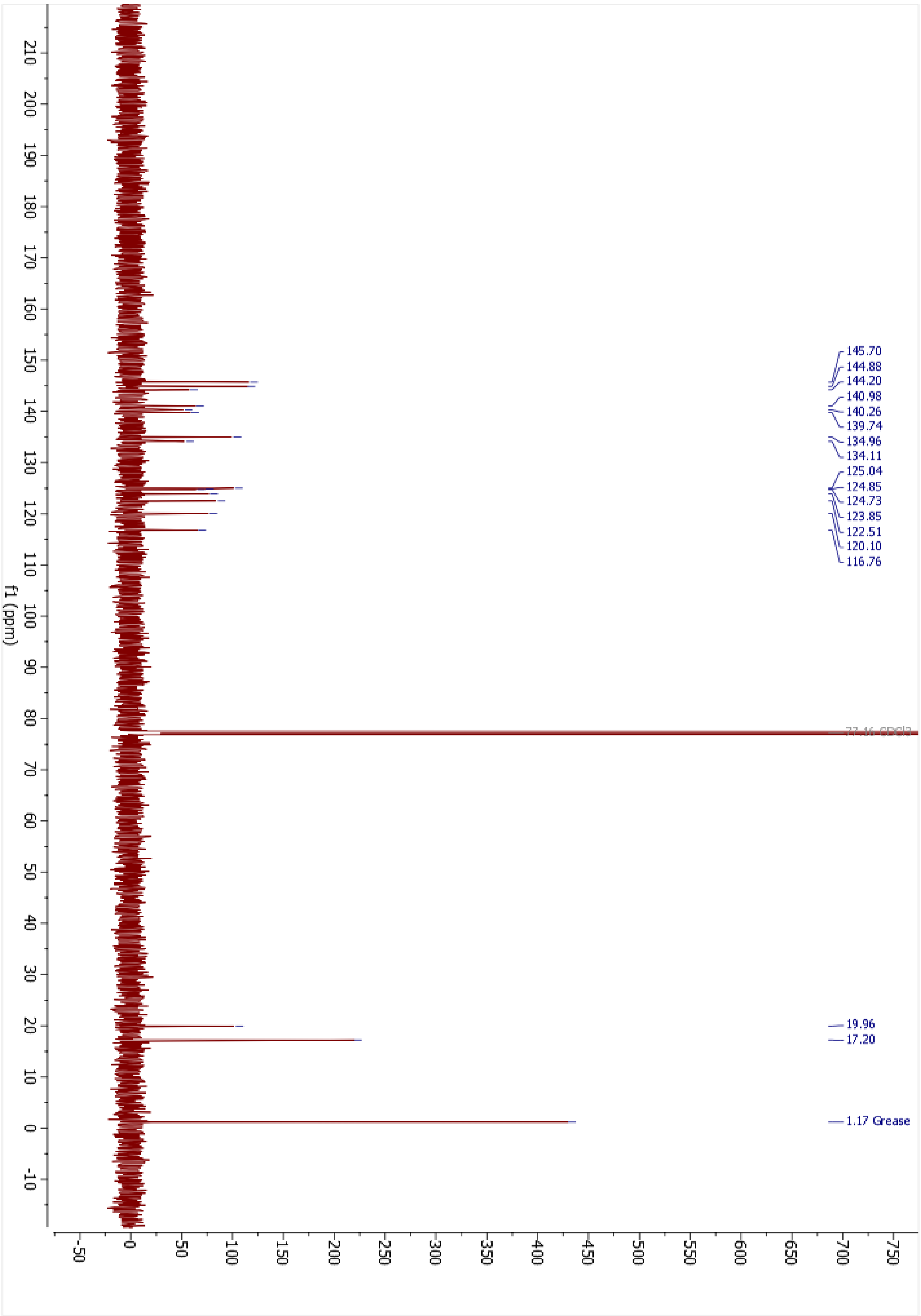

Figure S13: ^{13}C NMR ($CDCl_3$, 150 MHz) of porphyrin **31.**

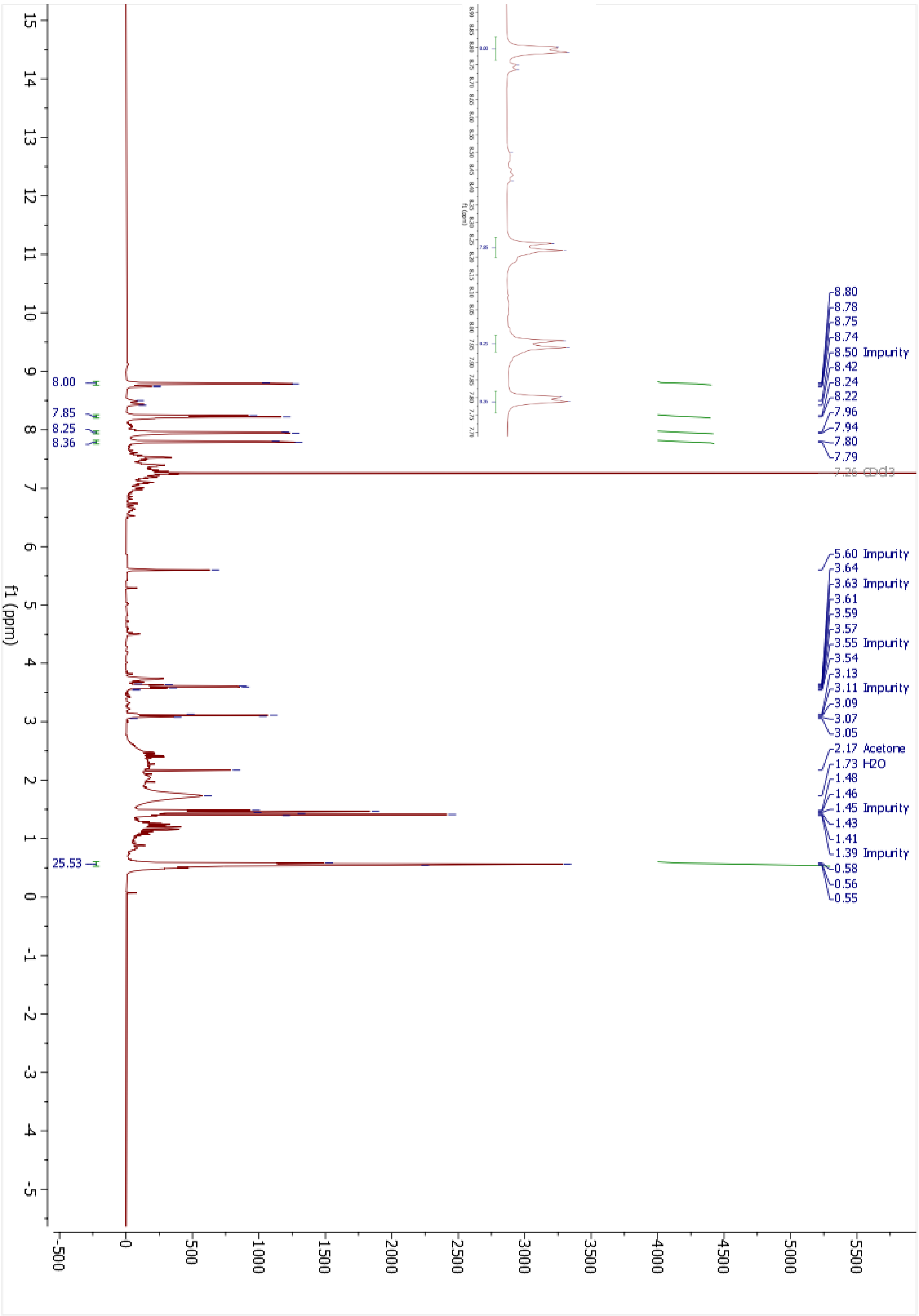

Figure S14: ^{1}H NMR ($CDCl_3$, 600 MHz) of porphyrin **32** including an embedded zoom of the aromatic region (δ = 8.90 – 7.70 ppm).

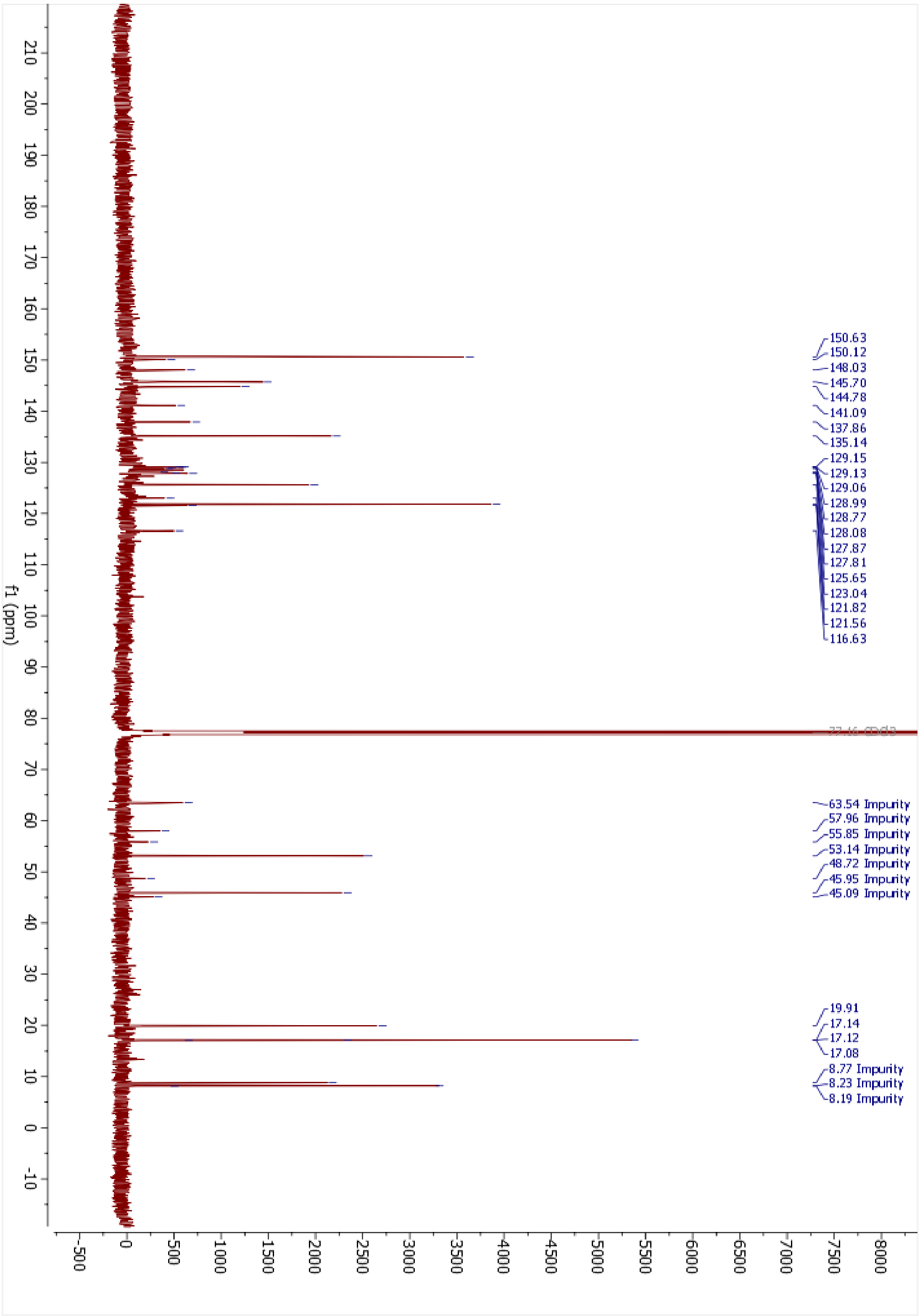

Figure S15: ^{13}C NMR ($CDCl_3$, 150 MHz) of porphyrin **32**.

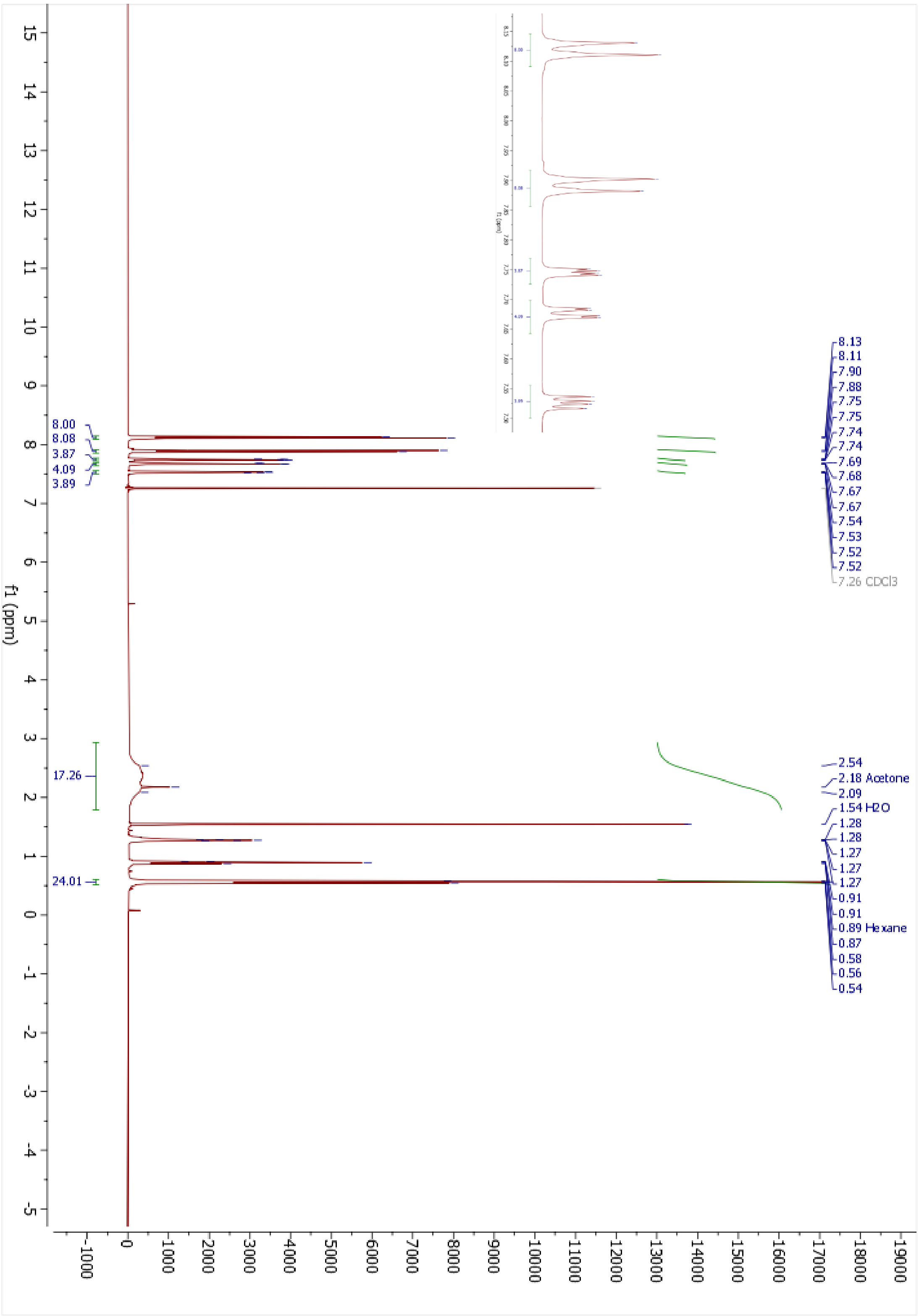

Figure S16: ^{1}H NMR ($CDCl_3$, 400 MHz) of porphyrin **33** including an embedded zoom of the aromatic region (δ = 8.15 – 7.50 ppm).

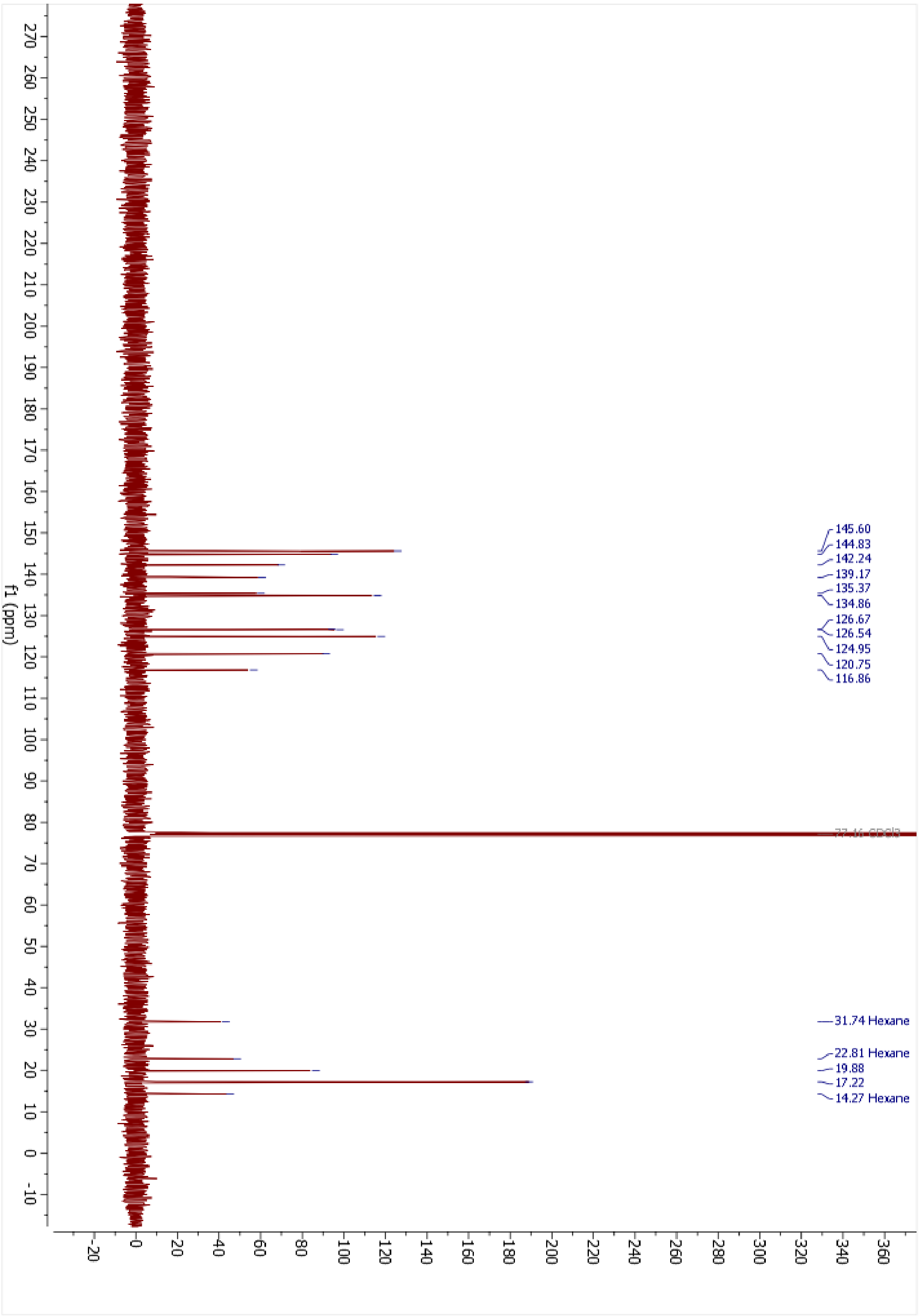

Figure S17: ^{13}C NMR ($CDCl_3$, 100 MHz) of porphyrin **33.**

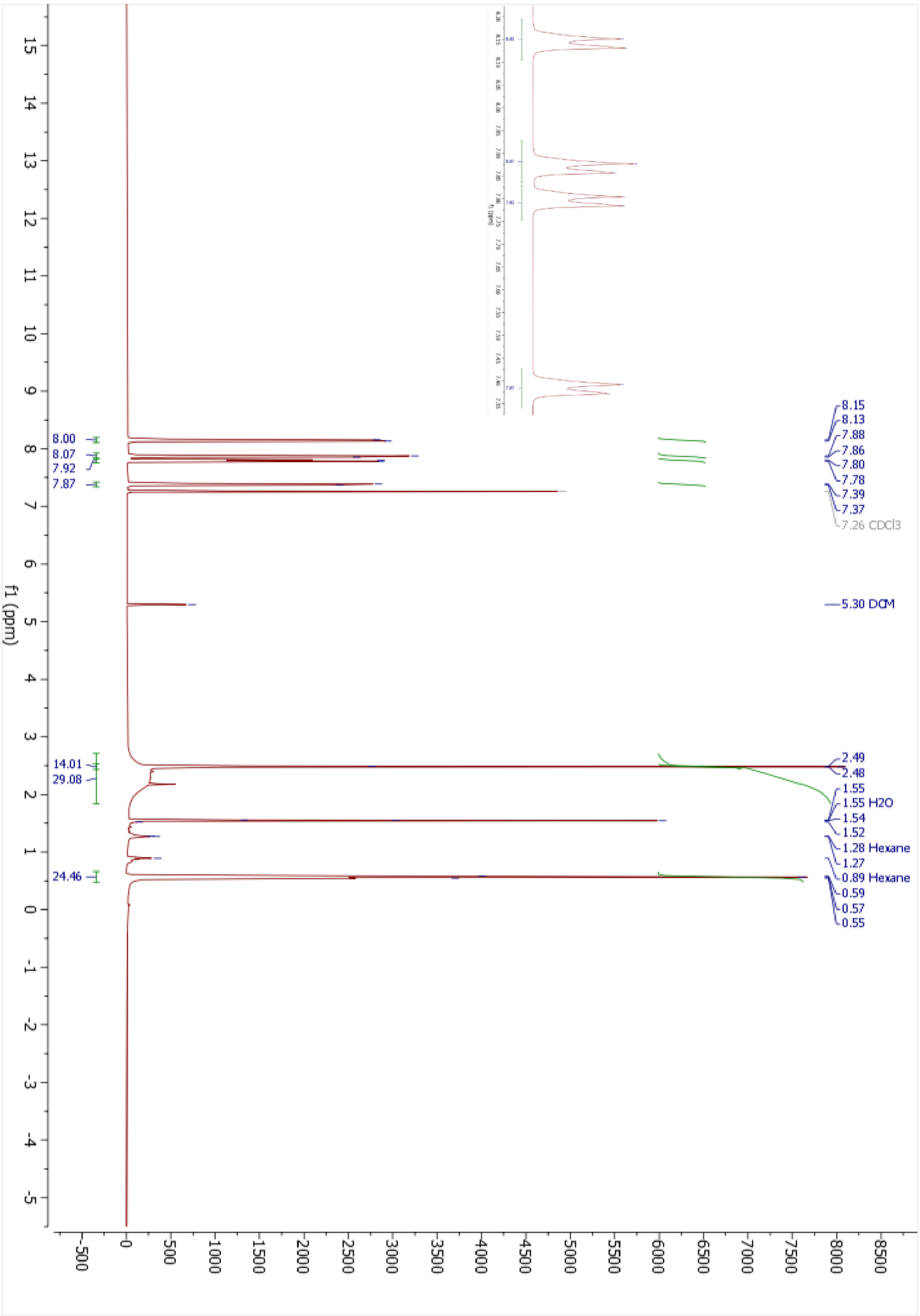

Figure S18: ^{1}H NMR ($CDCl_3$, 400 MHz) of porphyrin **34** including an embedded zoom of the aromatic region (δ = 8.20 – 7.35 ppm).

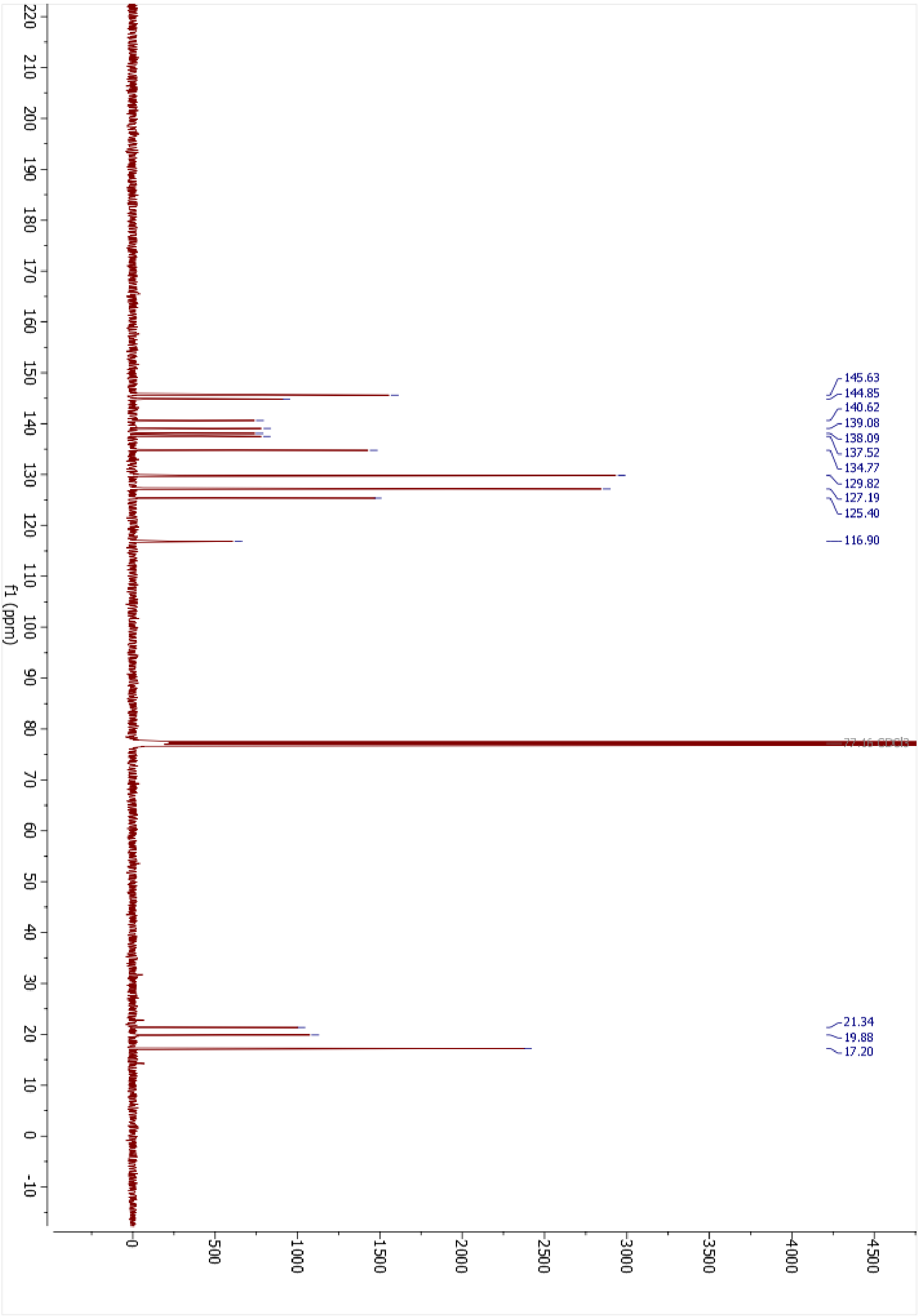

Figure S19: ^{13}C NMR ($CDCl_3$, 100 MHz) of porphyrin **34**.

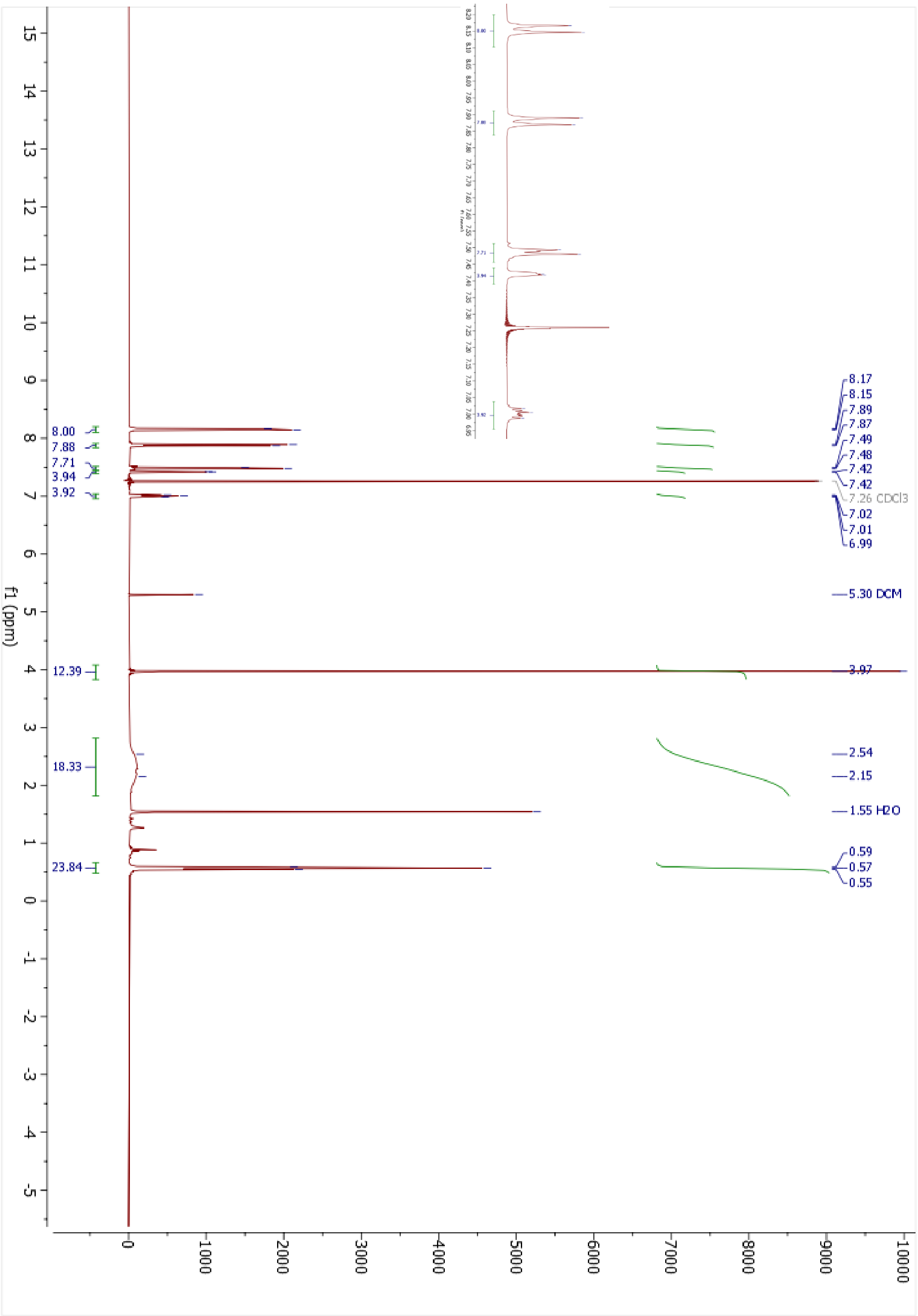

Figure S20: ^{1}H NMR ($CDCl_3$, 400 MHz) of porphyrin **35** including an embedded zoom of the aromatic region (δ = 8.20 – 6.90 ppm).

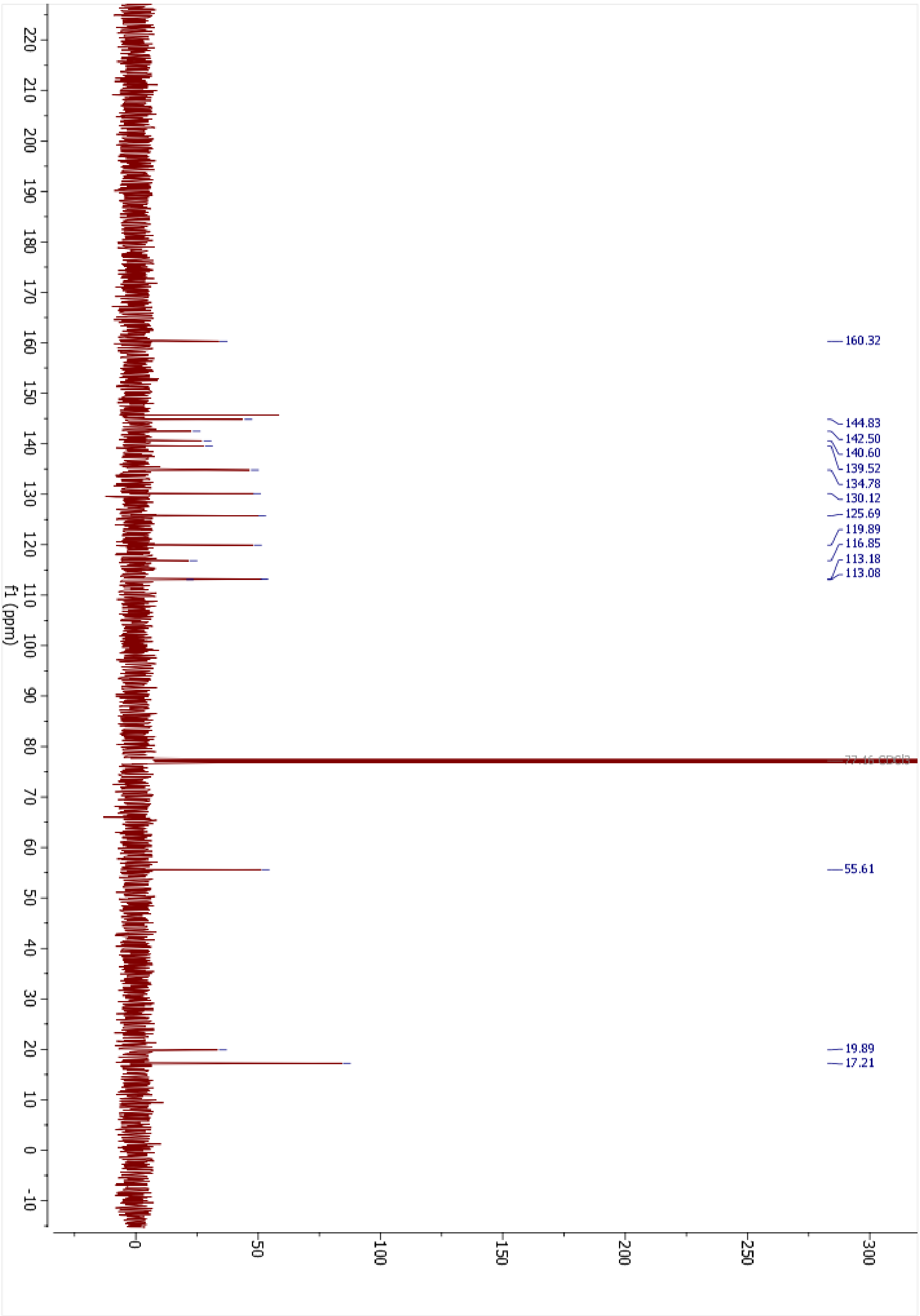

Figure S21: ^{13}C NMR ($CDCl_3$, 100 MHz) of porphyrin **35.**

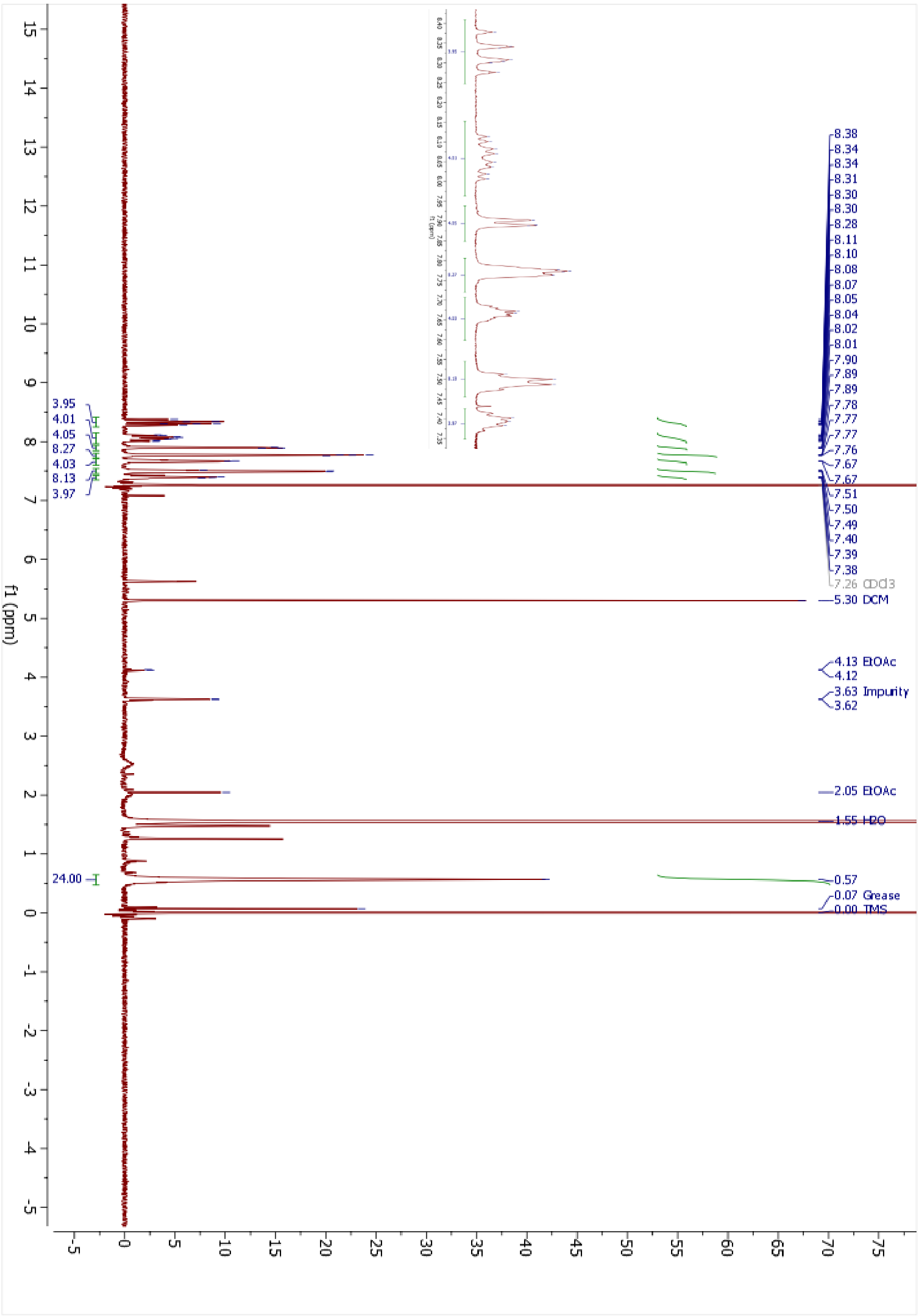

Figure S22: ^{1}H NMR ($CDCl_3$, 600 MHz) of porphyrin **36** including an embedded zoom of the aromatic region (δ = 8.40 – 7.35 ppm).

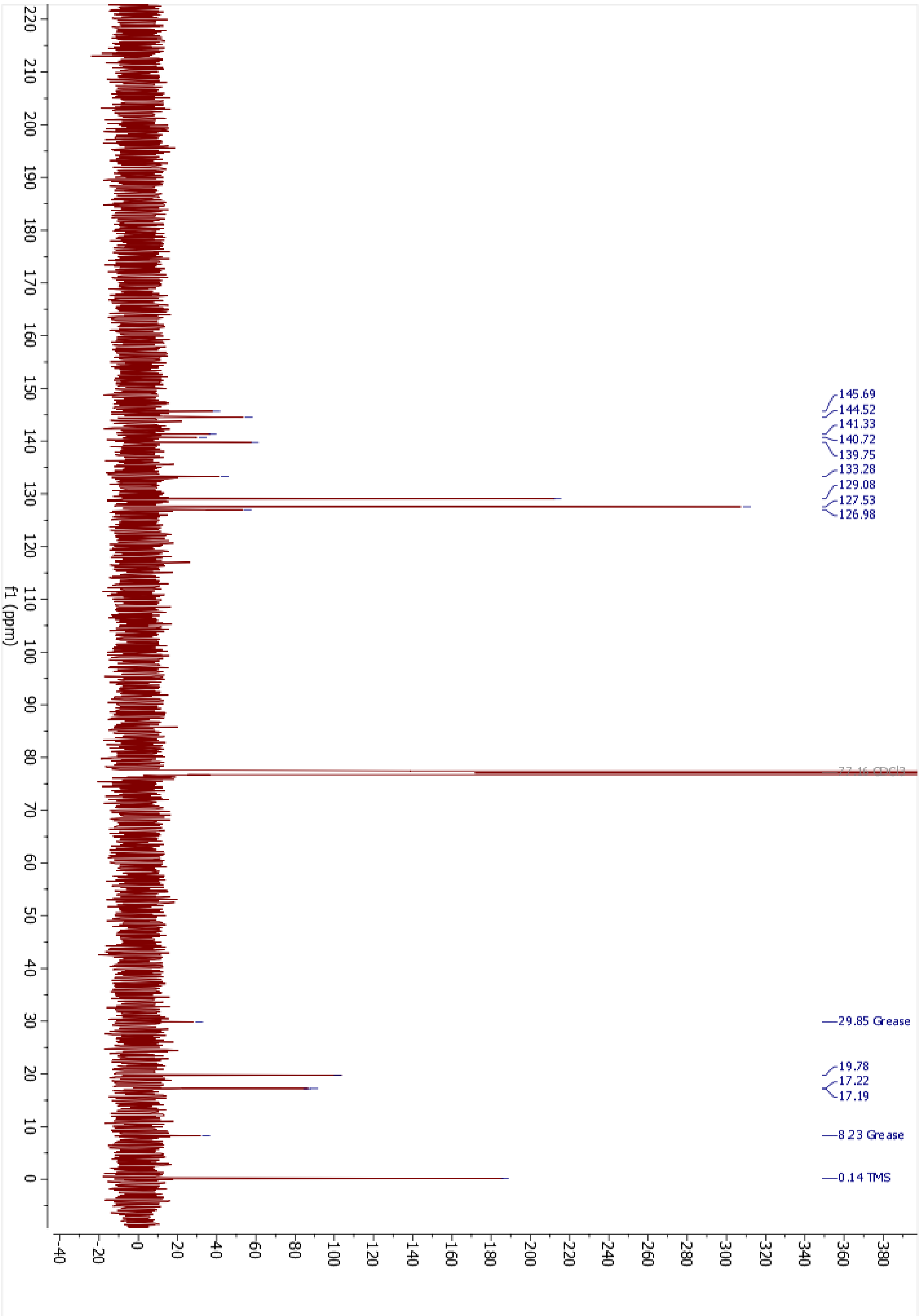

Figure S23: ^{13}C NMR ($CDCl_3$, 100 MHz) of porphyrin **36**.

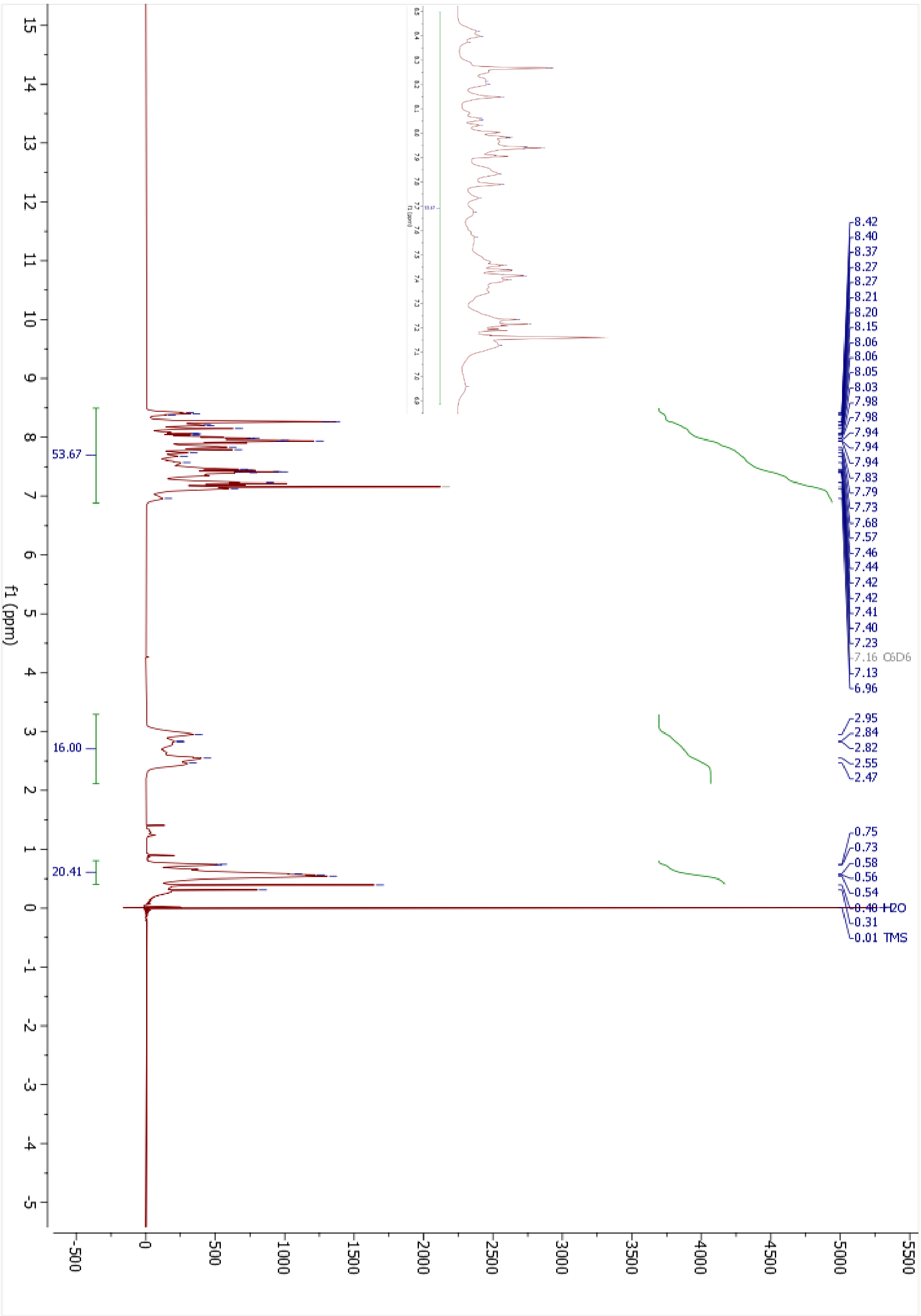

Figure S24: ^{1}H NMR (C_6D_6, 600 MHz) of porphyrin **37** including an embedded zoom of the aromatic region (δ = 8.50 – 6.90 ppm).

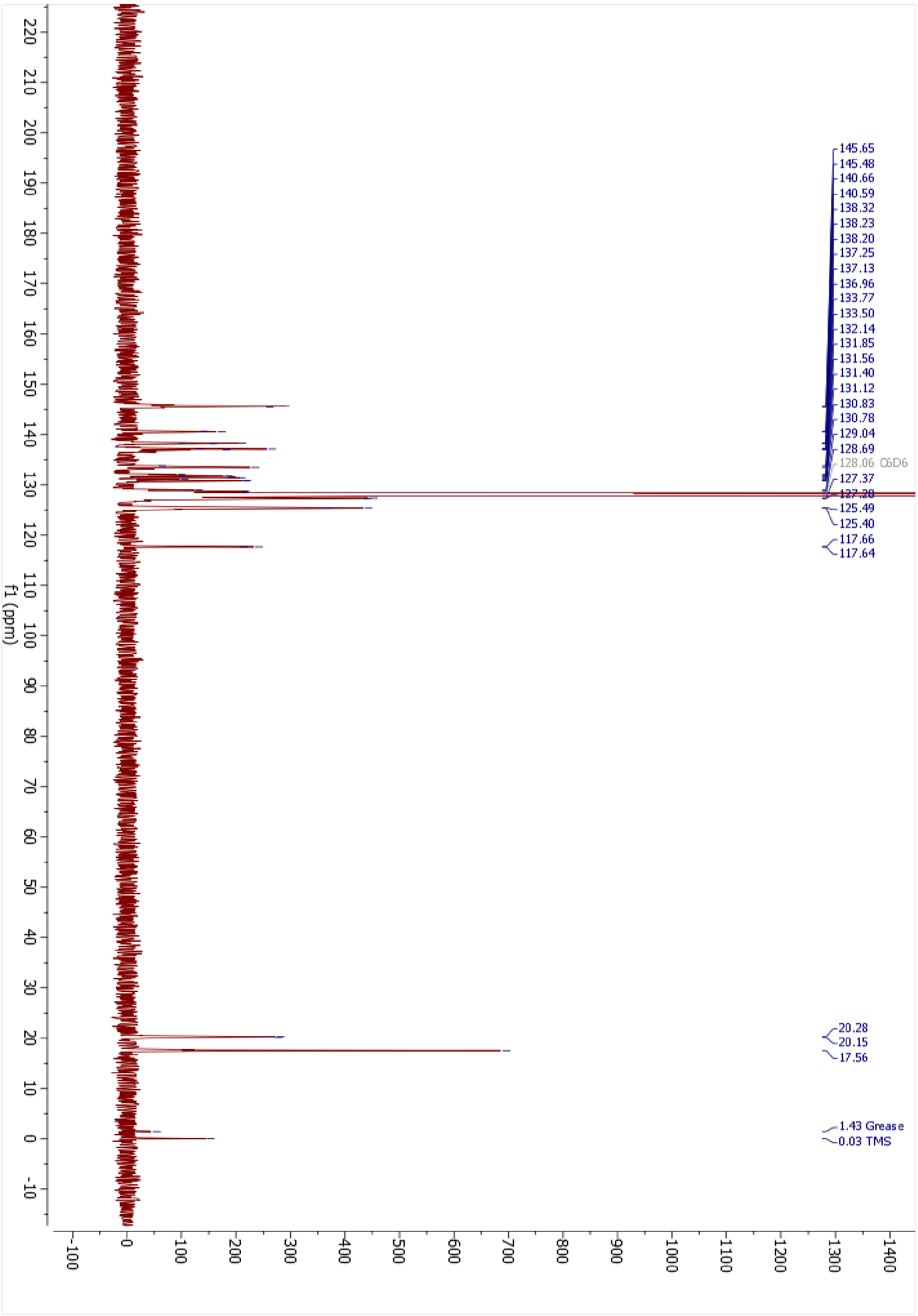

Figure S25: ^{13}C NMR (C_6D_6, 150 MHz) of porphyrin **37.**

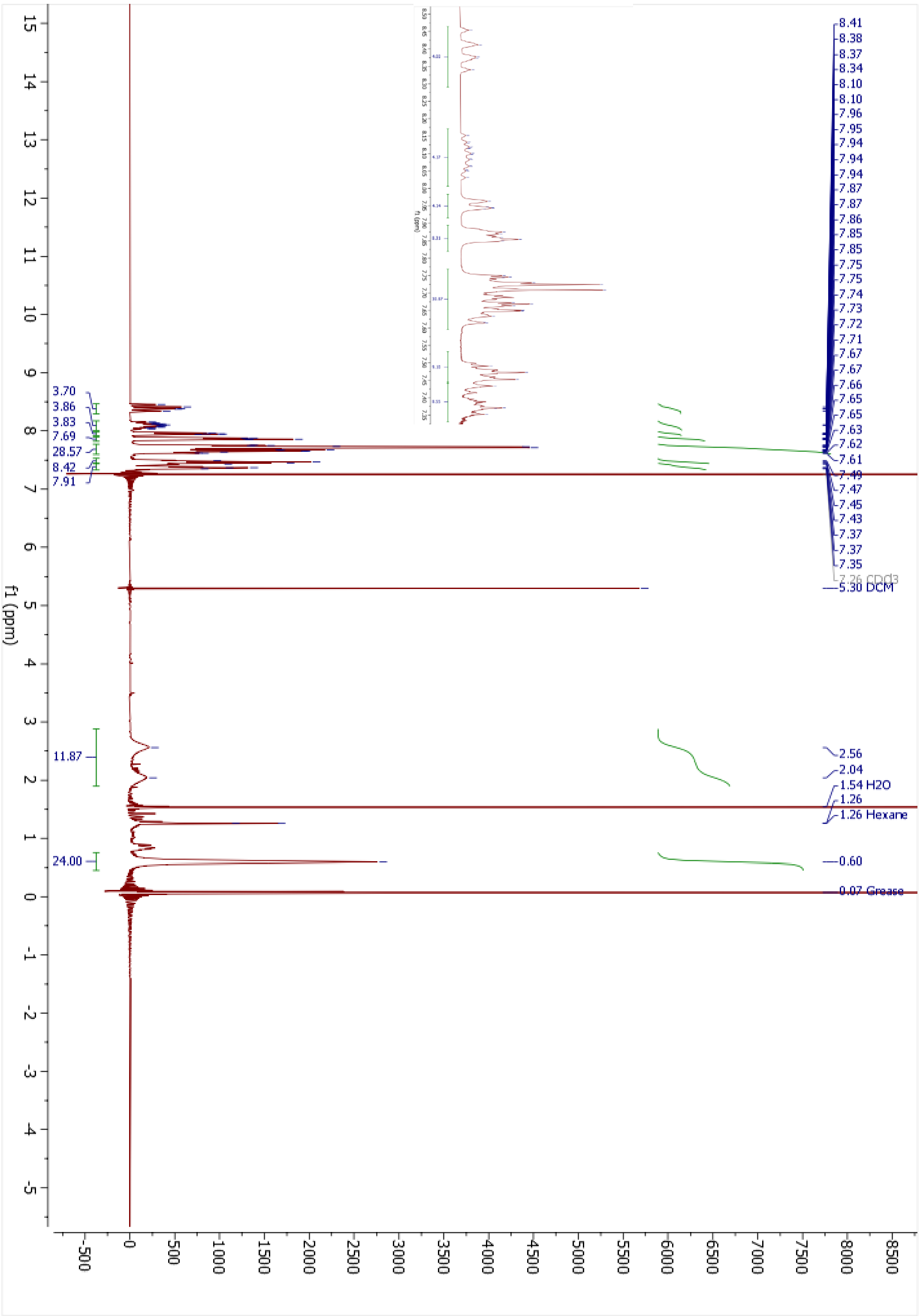

Figure S26: ^{1}H NMR ($CDCl_3$, 600 MHz) of porphyrin **38** including an embedded zoom of the aromatic region (δ = 8.50 – 7.35 ppm).

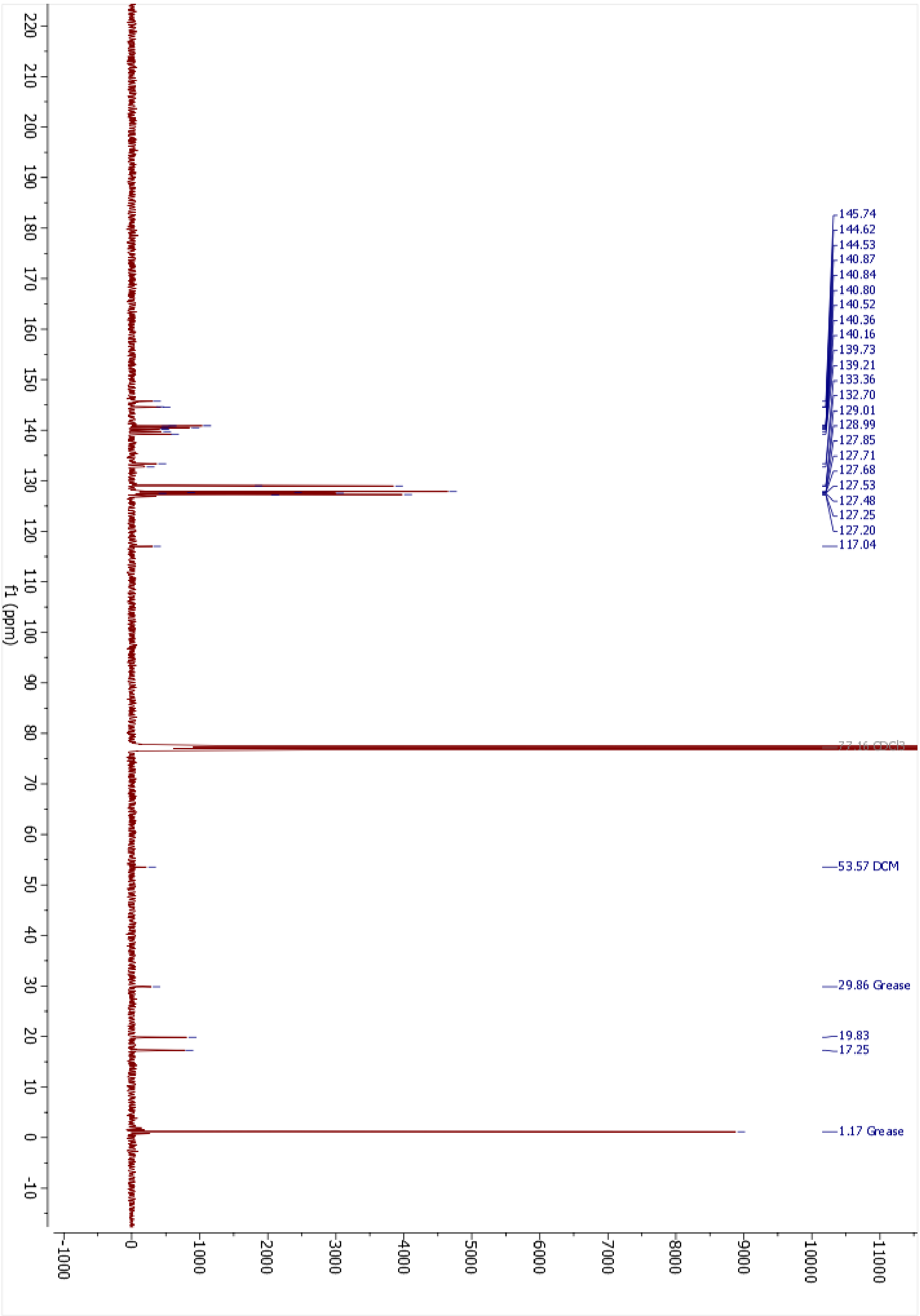

Figure S27: ^{13}C NMR ($CDCl_3$, 100 MHz) of porphyrin **38.**

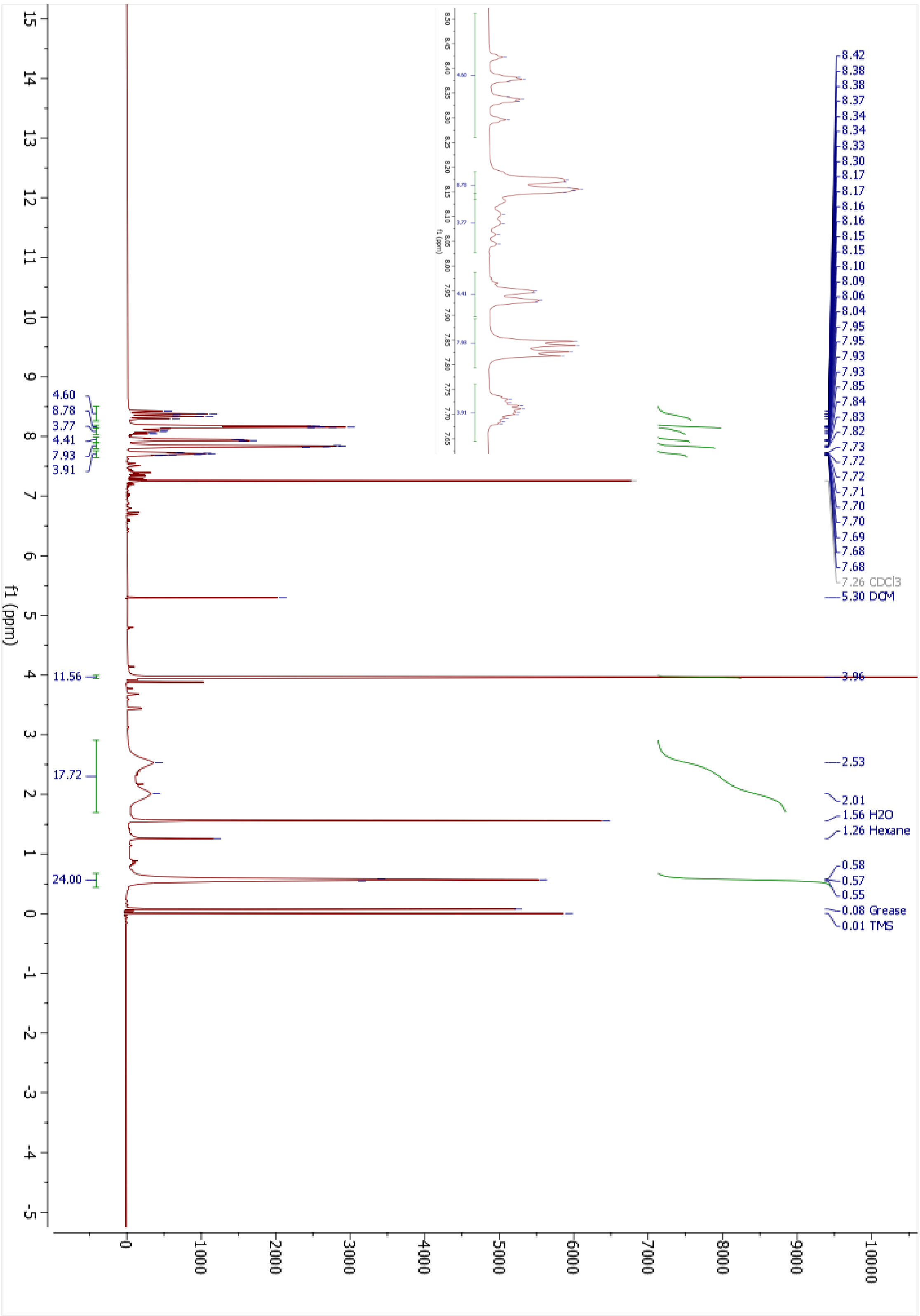

Figure S28: ^{1}H NMR ($CDCl_3$, 400 MHz) of porphyrin **39** including an embedded zoom of the aromatic region (δ = 8.50 – 7.65 ppm).

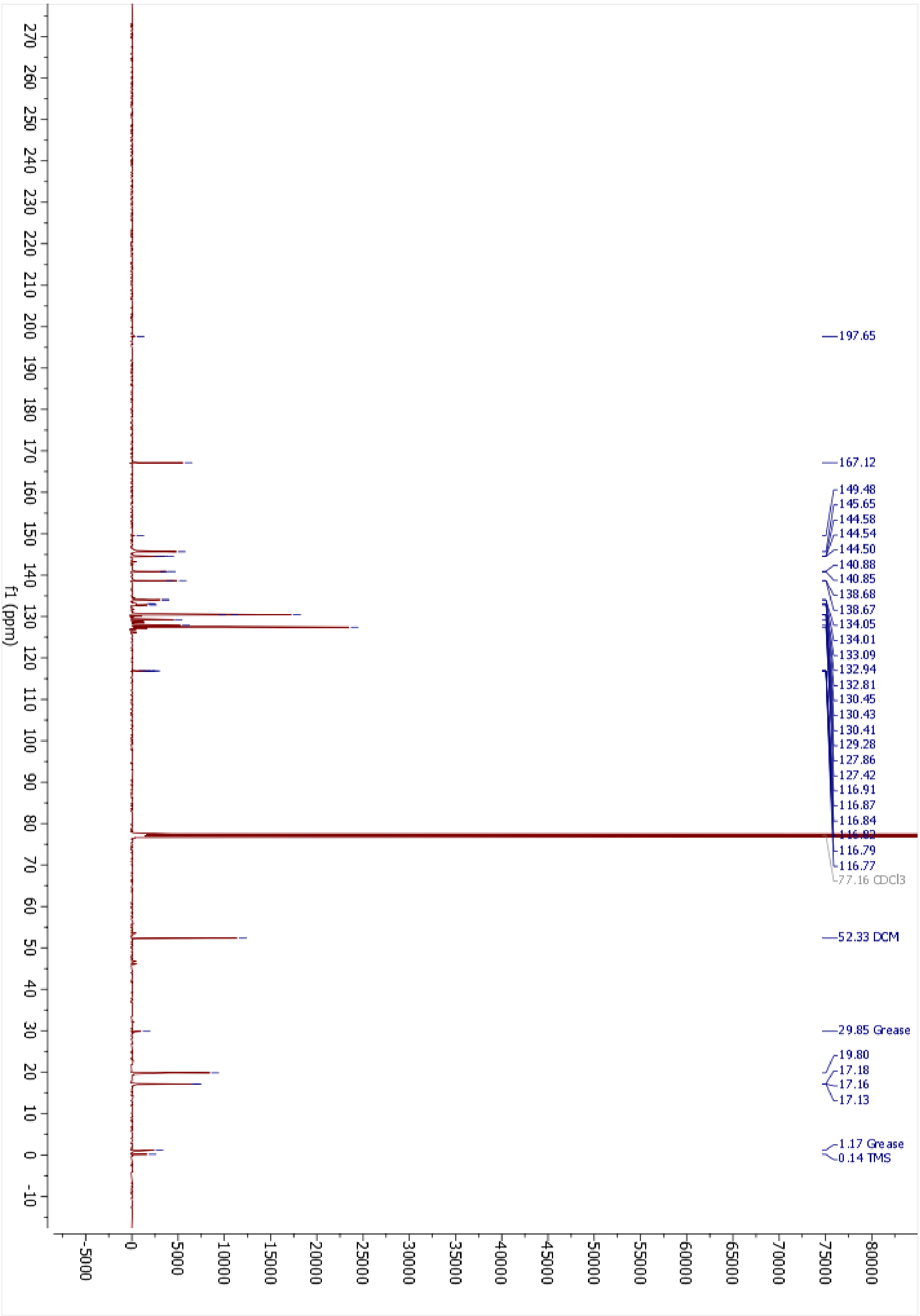

Figure S29: ^{13}C NMR ($CDCl_3$, 100 MHz) of porphyrin **39.**

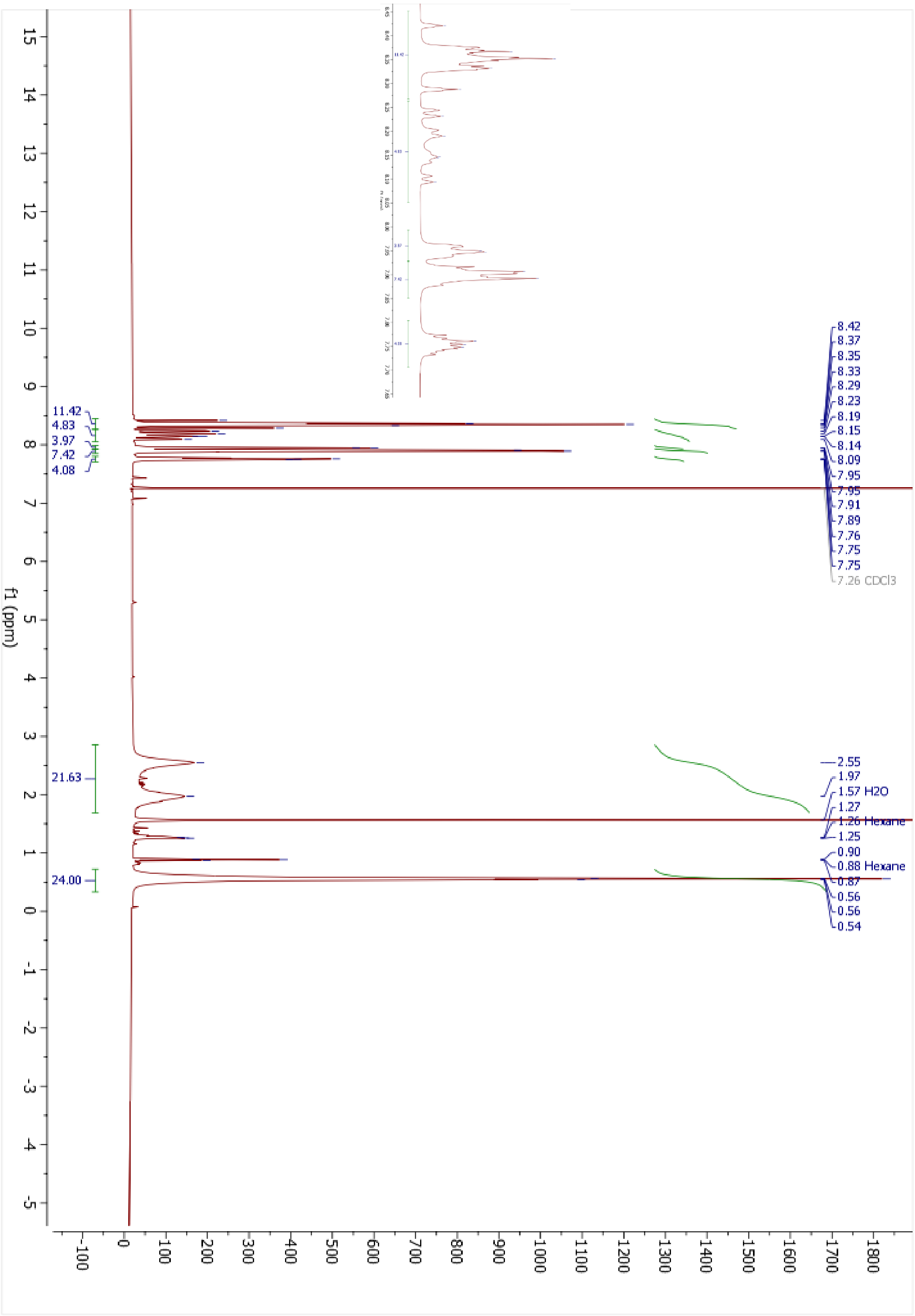

Figure S30: ^{1}H NMR ($CDCl_3$, 600 MHz) of porphyrin **40** including an embedded zoom of the aromatic region (δ = 8.45 – 7.65 ppm).

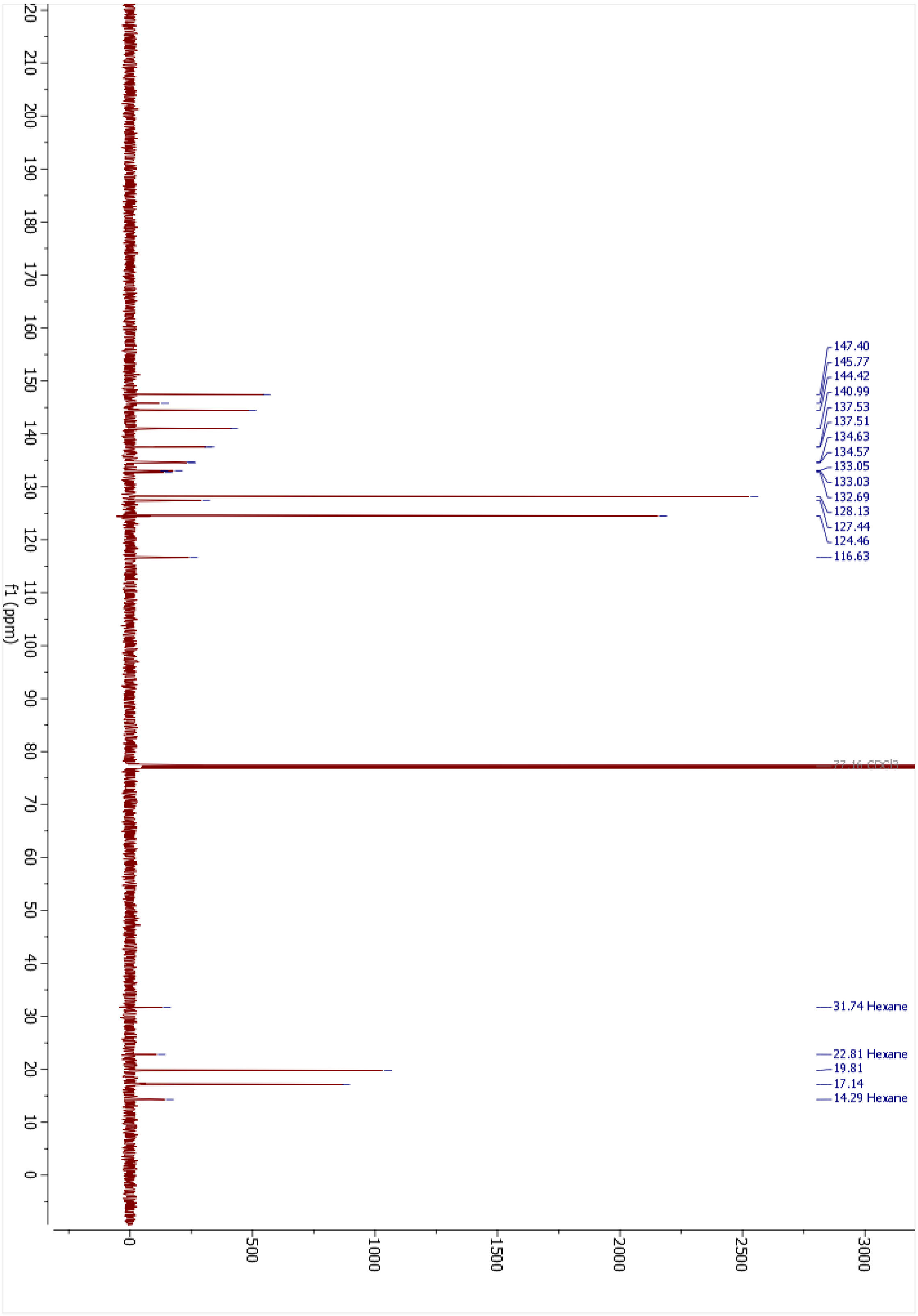

Figure S31: ^{13}C NMR ($CDCl_3$, 100 MHz) of porphyrin **40.**

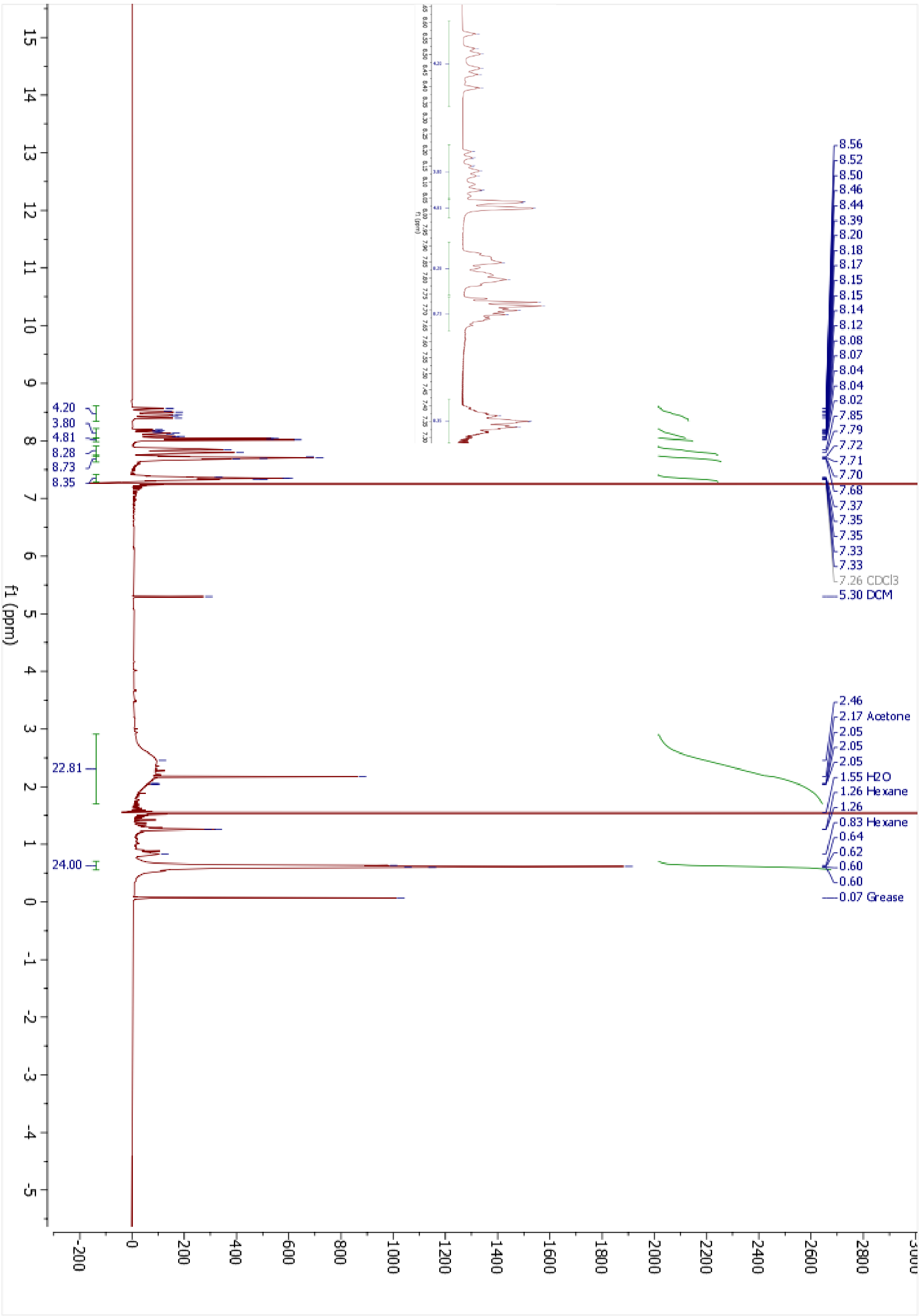

Figure S32: ^{1}H NMR ($CDCl_3$, 600 MHz) of porphyrin **41** including an embedded zoom of the aromatic region (δ = 8.65 – 7.30 ppm).

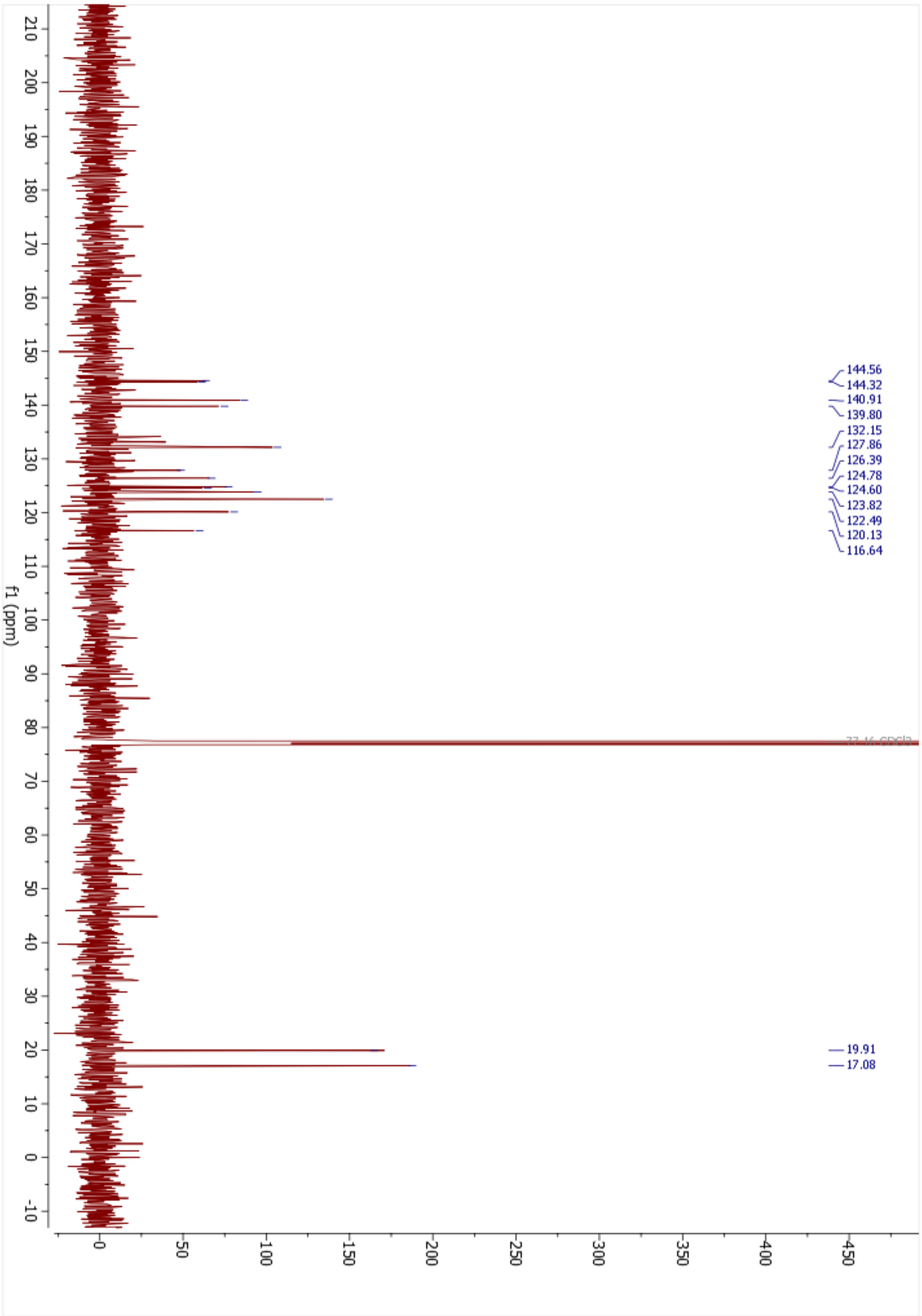

Figure S33: ^{13}C NMR ($CDCl_3$, 100 MHz) of porphyrin **41.**

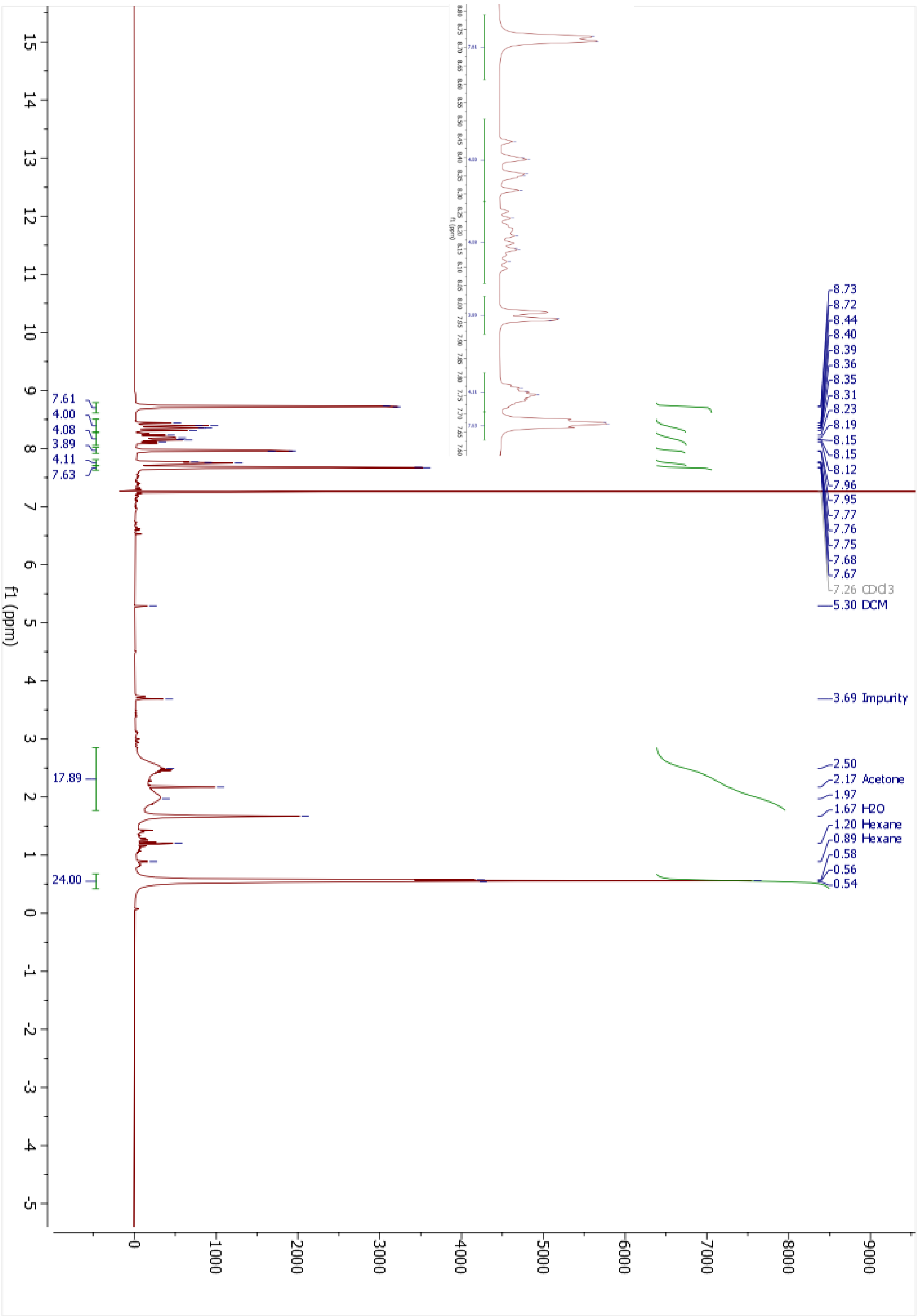

Figure S34: ^{1}H NMR ($CDCl_3$, 600 MHz) of porphyrin **42** including an embedded zoom of the aromatic region (δ = 8.80 – 7.60 ppm).

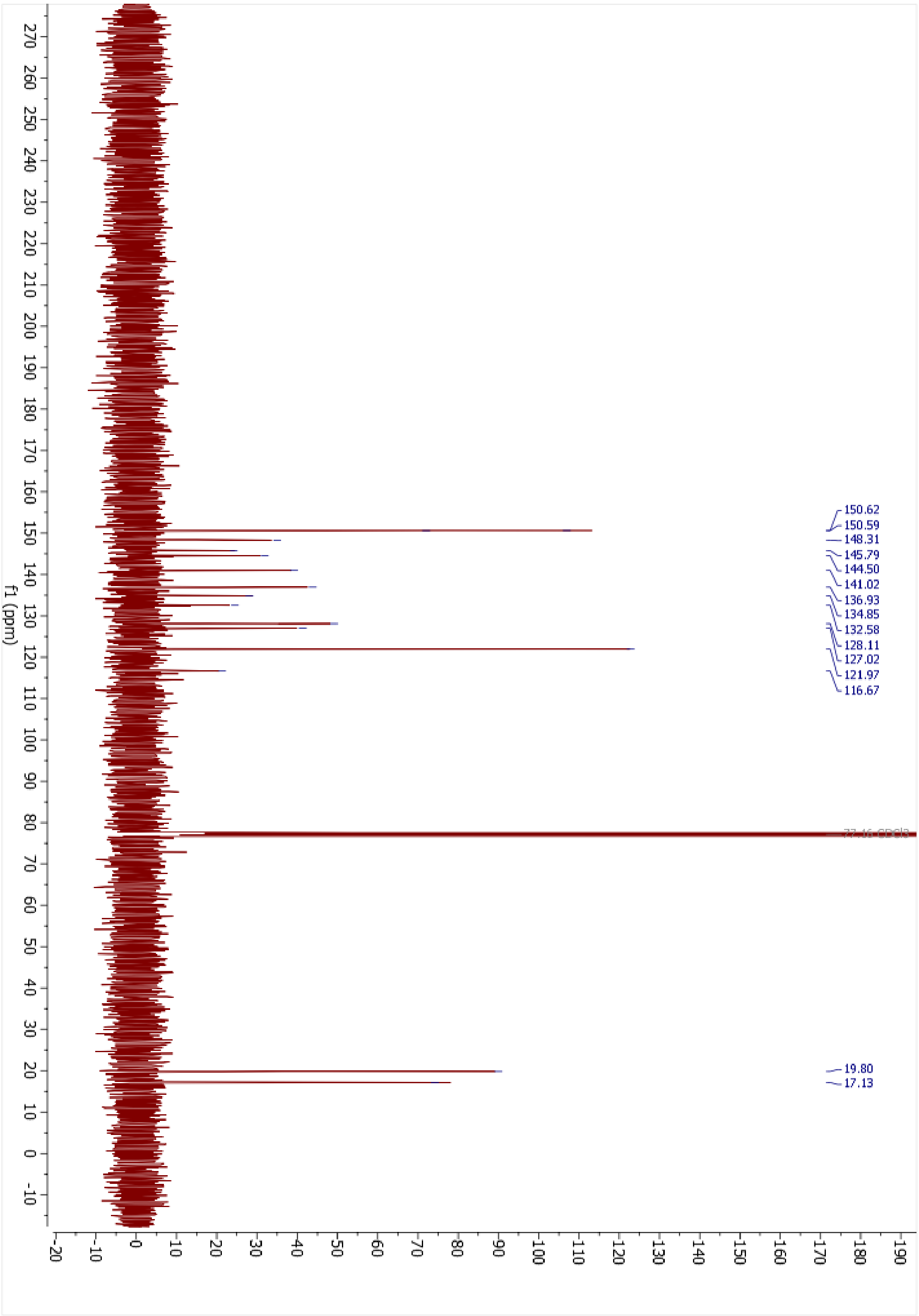

Figure S35: ^{13}C NMR ($CDCl_3$, 100 MHz) of porphyrin **42**.

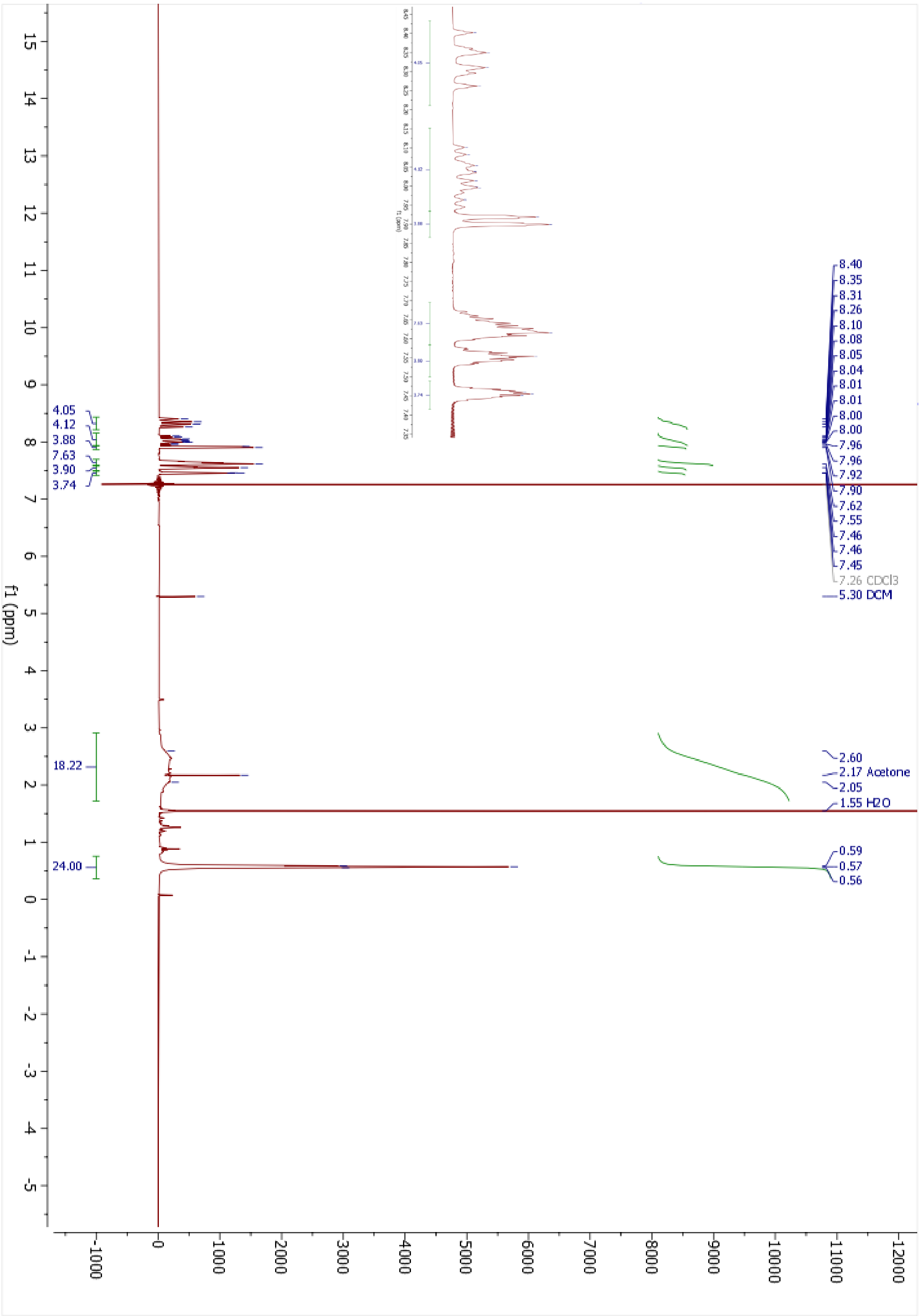

Figure S36: ^{1}H NMR ($CDCl_3$, 600 MHz) of porphyrin **43** including an embedded zoom of the aromatic region (δ = 8.40 – 7.35 ppm).

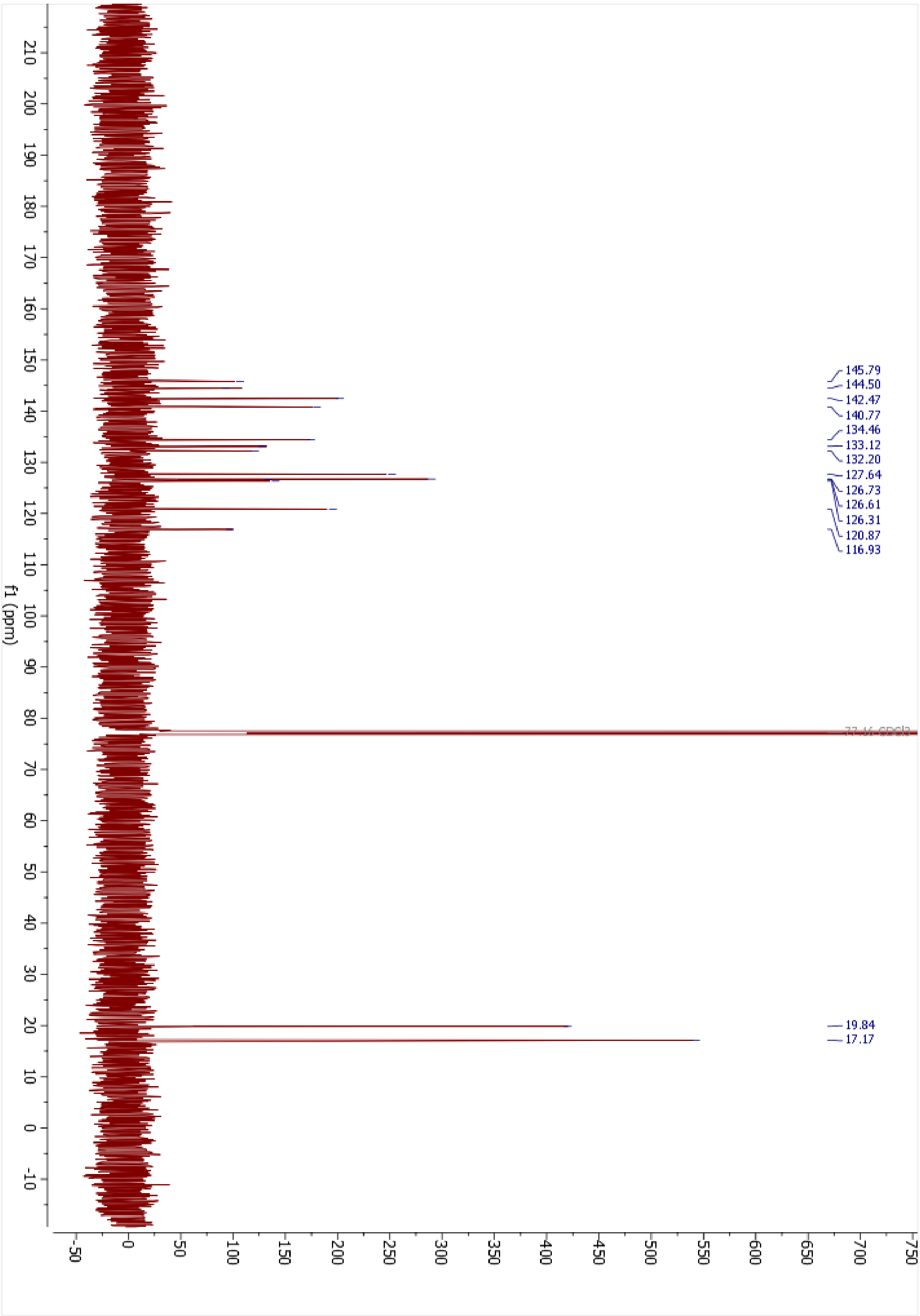

Figure S37: ^{13}C NMR ($CDCl_3$, 100 MHz) of porphyrin **43**.

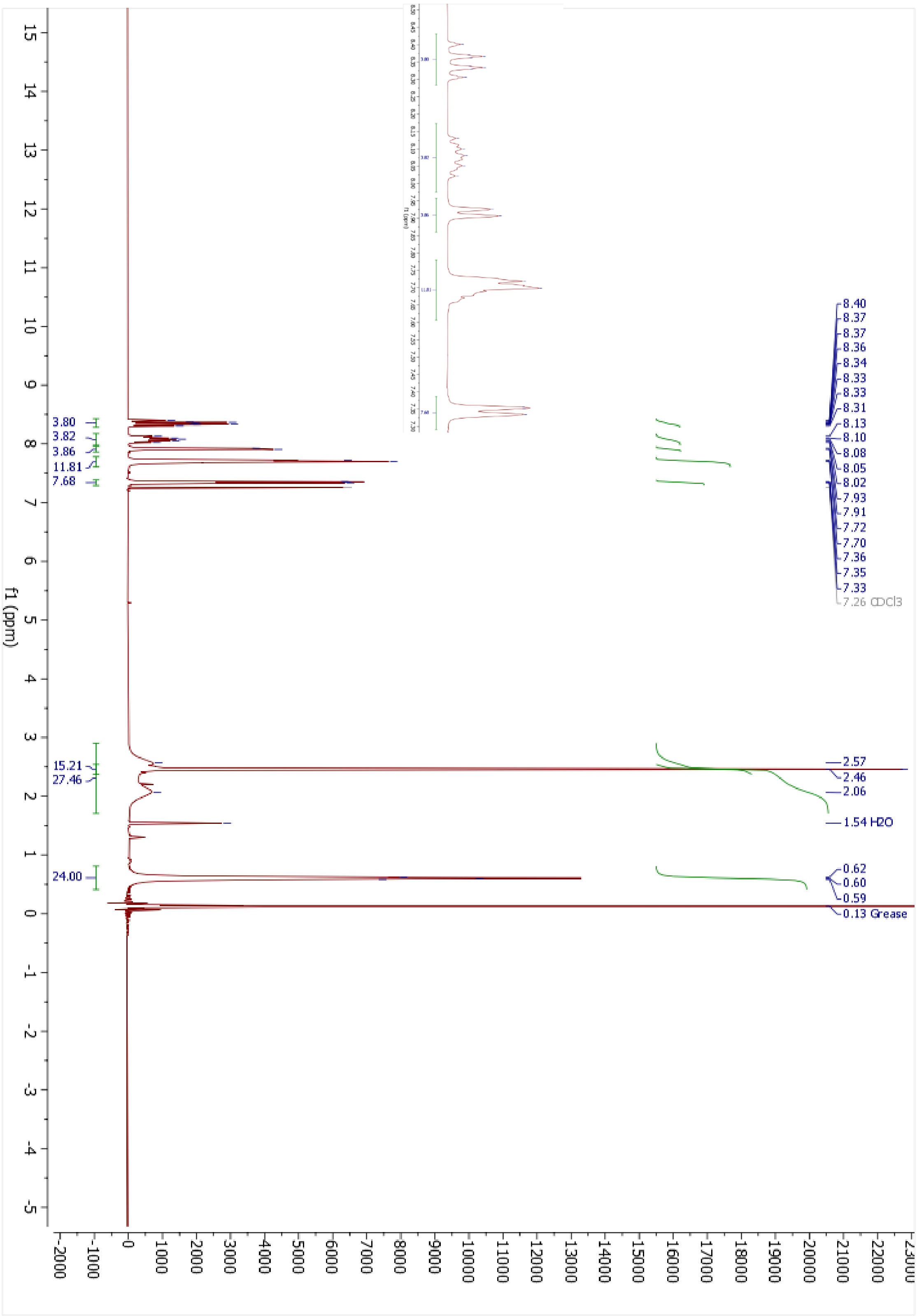

Figure S38: ^{1}H NMR ($CDCl_3$, 600 MHz) of porphyrin **44** including an embedded zoom of the aromatic region (δ = 8.50 – 7.30 ppm).

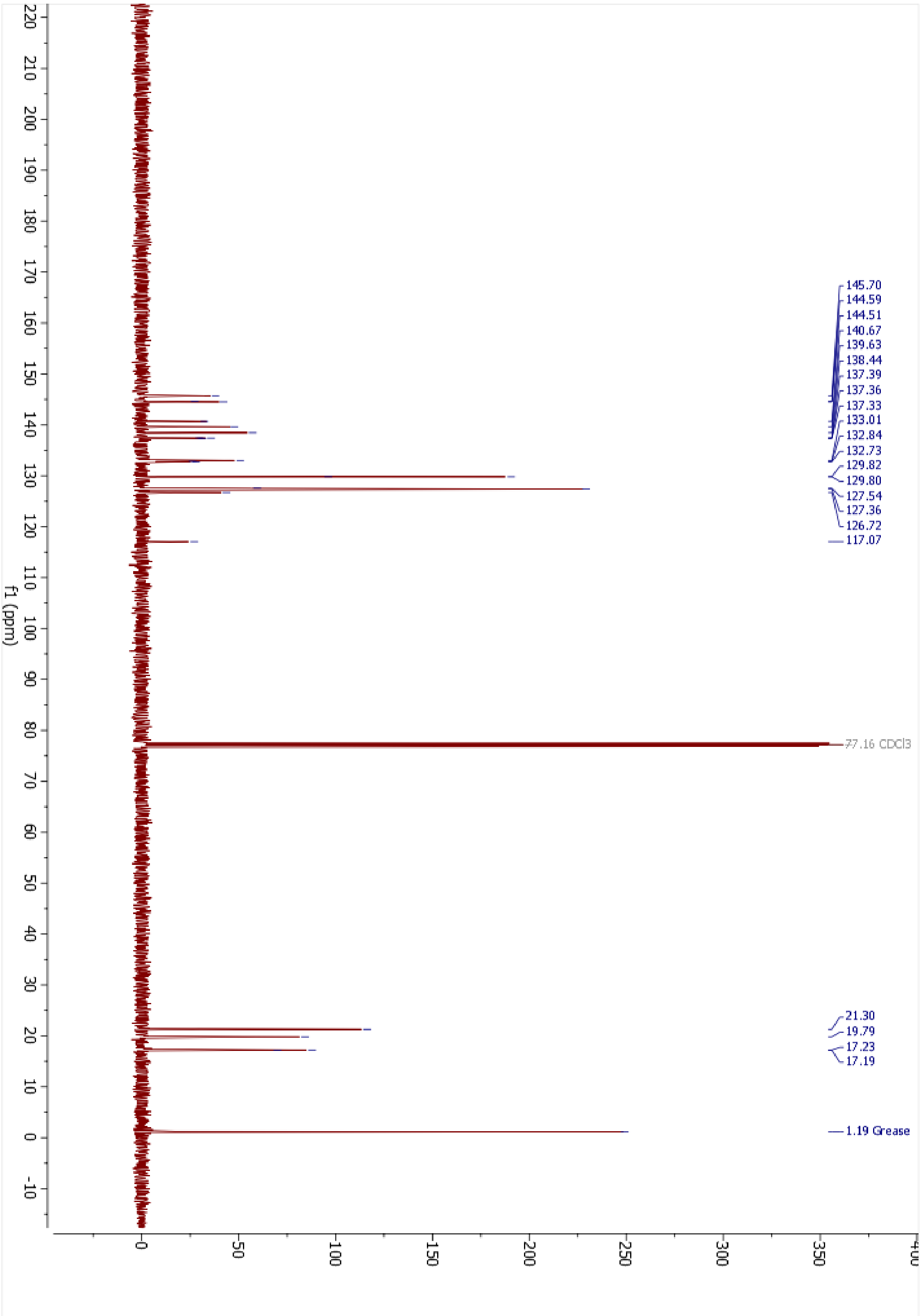

Figure S39: ^{13}C NMR ($CDCl_3$, 150 MHz) of porphyrin **44.**

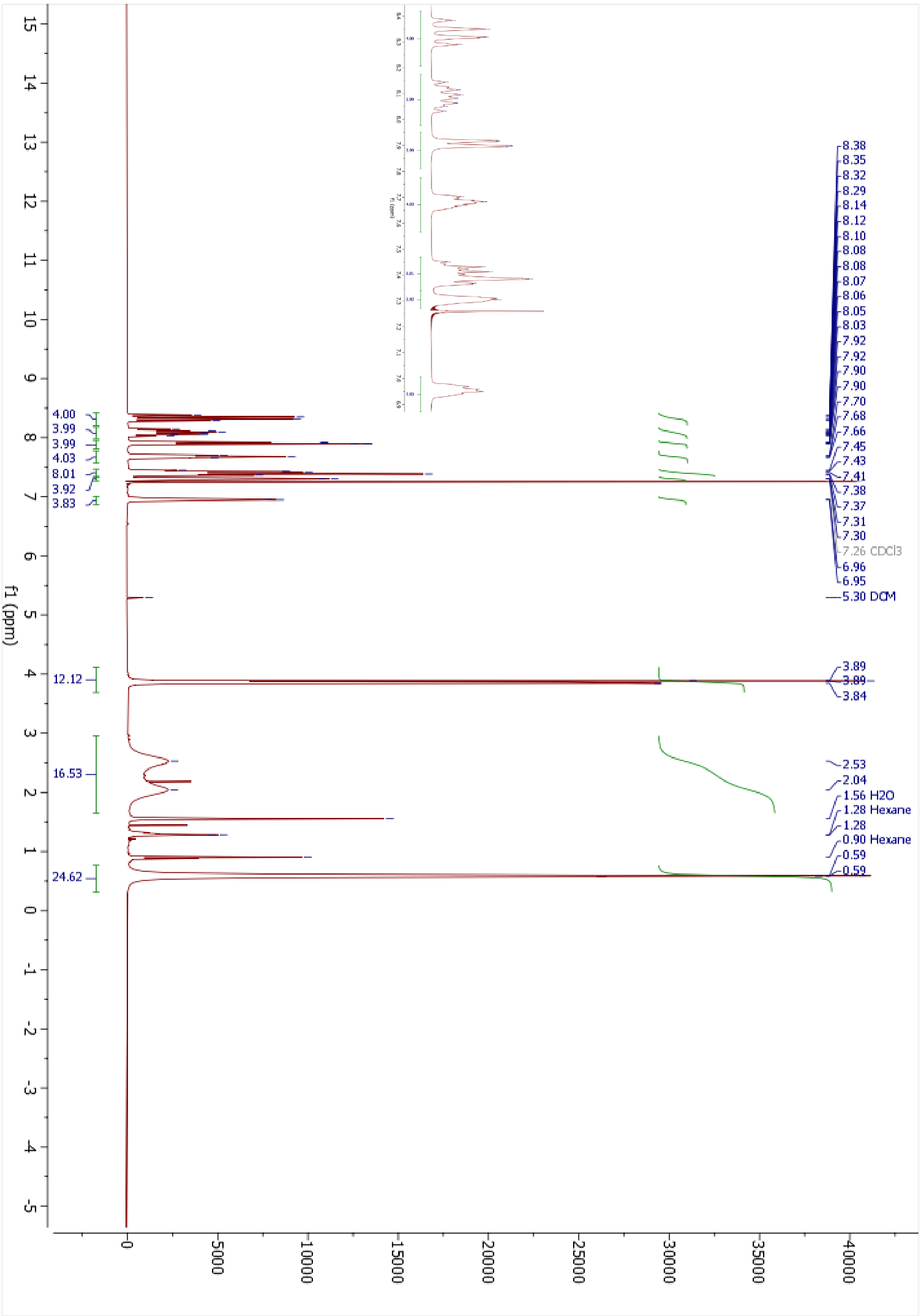

Figure S40: ^{1}H NMR ($CDCl_3$, 600 MHz) of porphyrin **45** including an embedded zoom of the aromatic region (δ = 8.40 – 6.90 ppm).

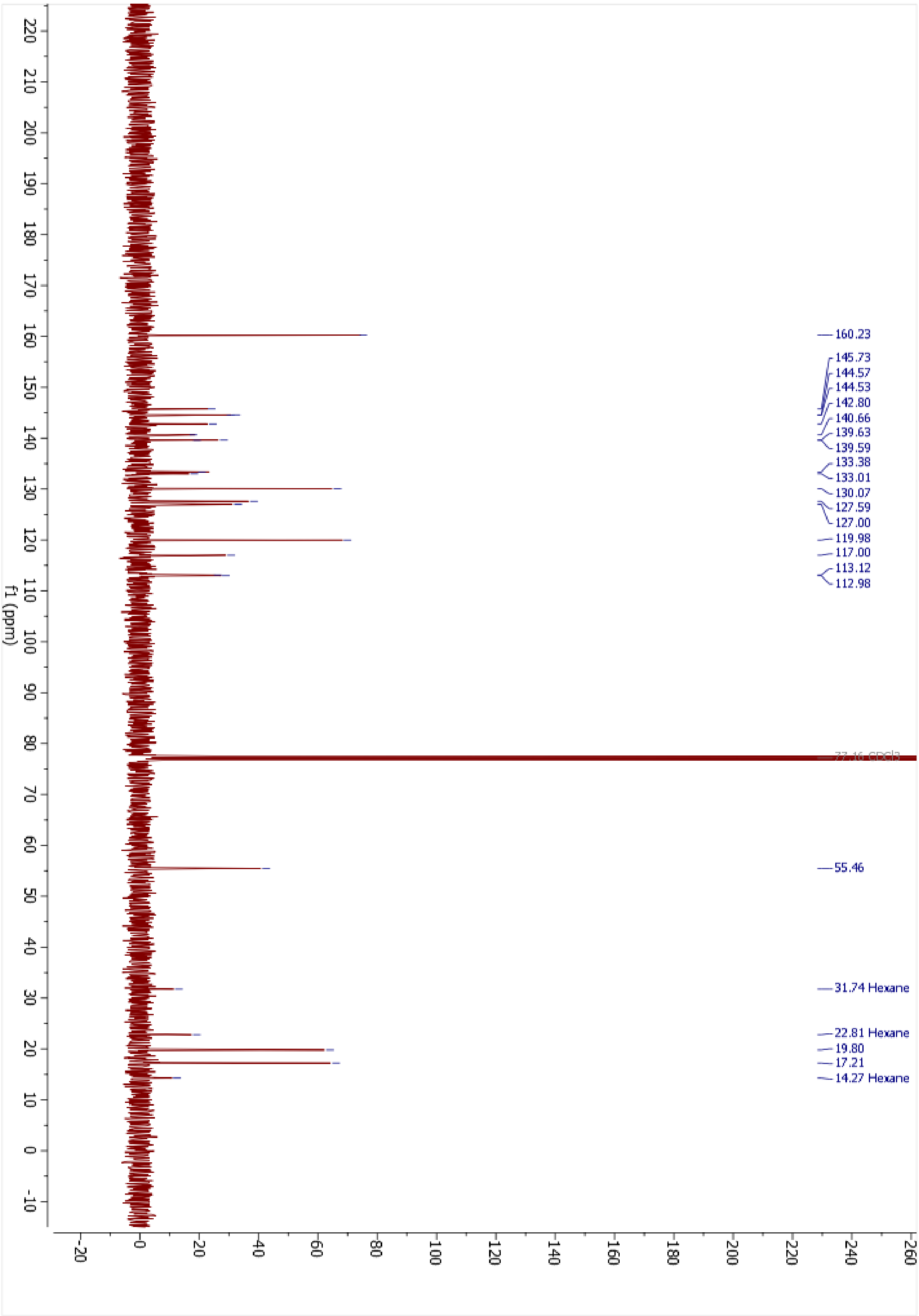

Figure S41: ^{13}C NMR ($CDCl_3$, 150 MHz) of porphyrin **45.**

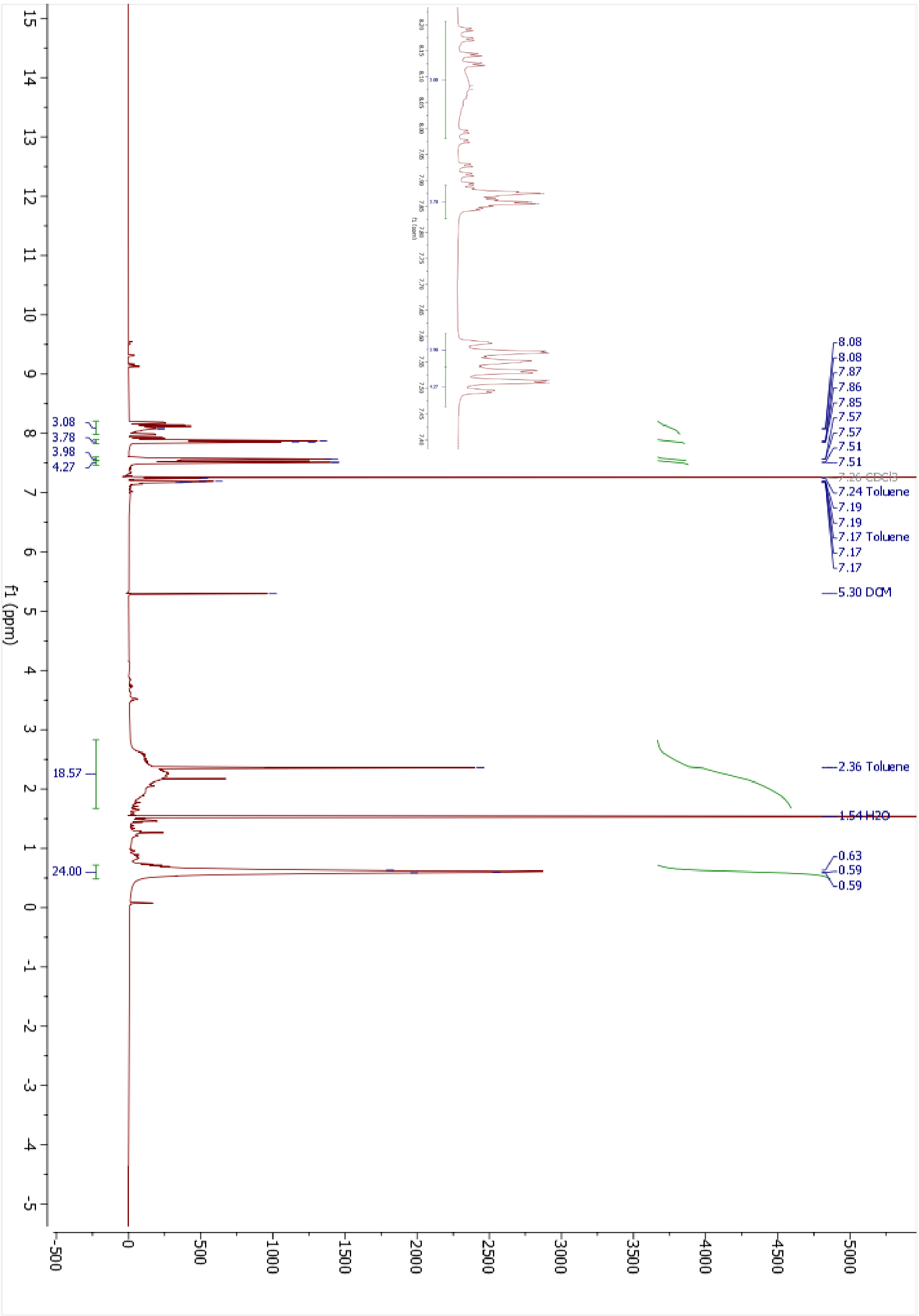

Figure S42: ^{1}H NMR ($CDCl_3$, 600 MHz) of porphyrin **11** including an embedded zoom of the aromatic region (δ = 8.20 – 7.40 ppm).

.

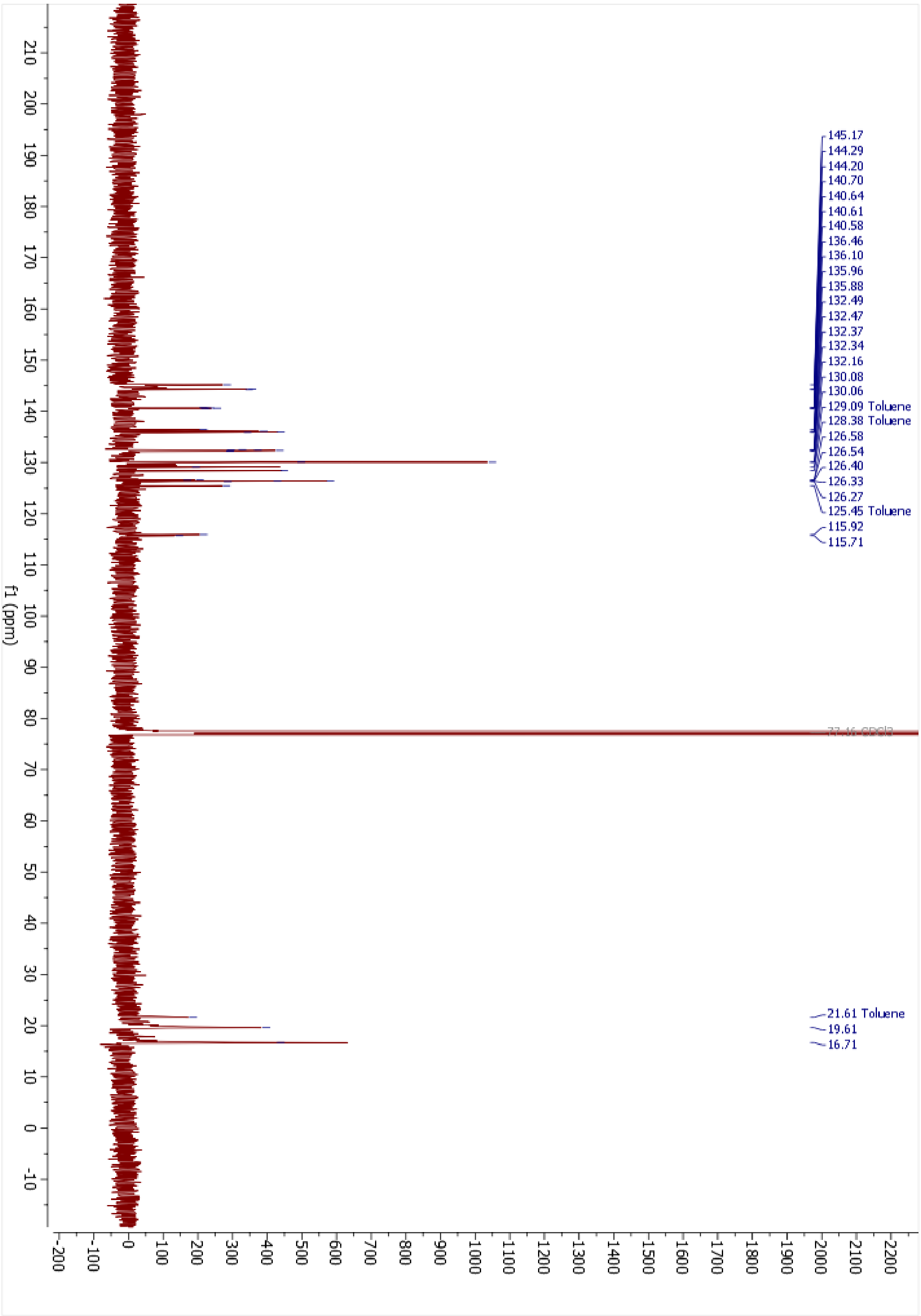

Figure S43: ^{13}C NMR ($CDCl_3$, 150 MHz) of porphyrin **11.**

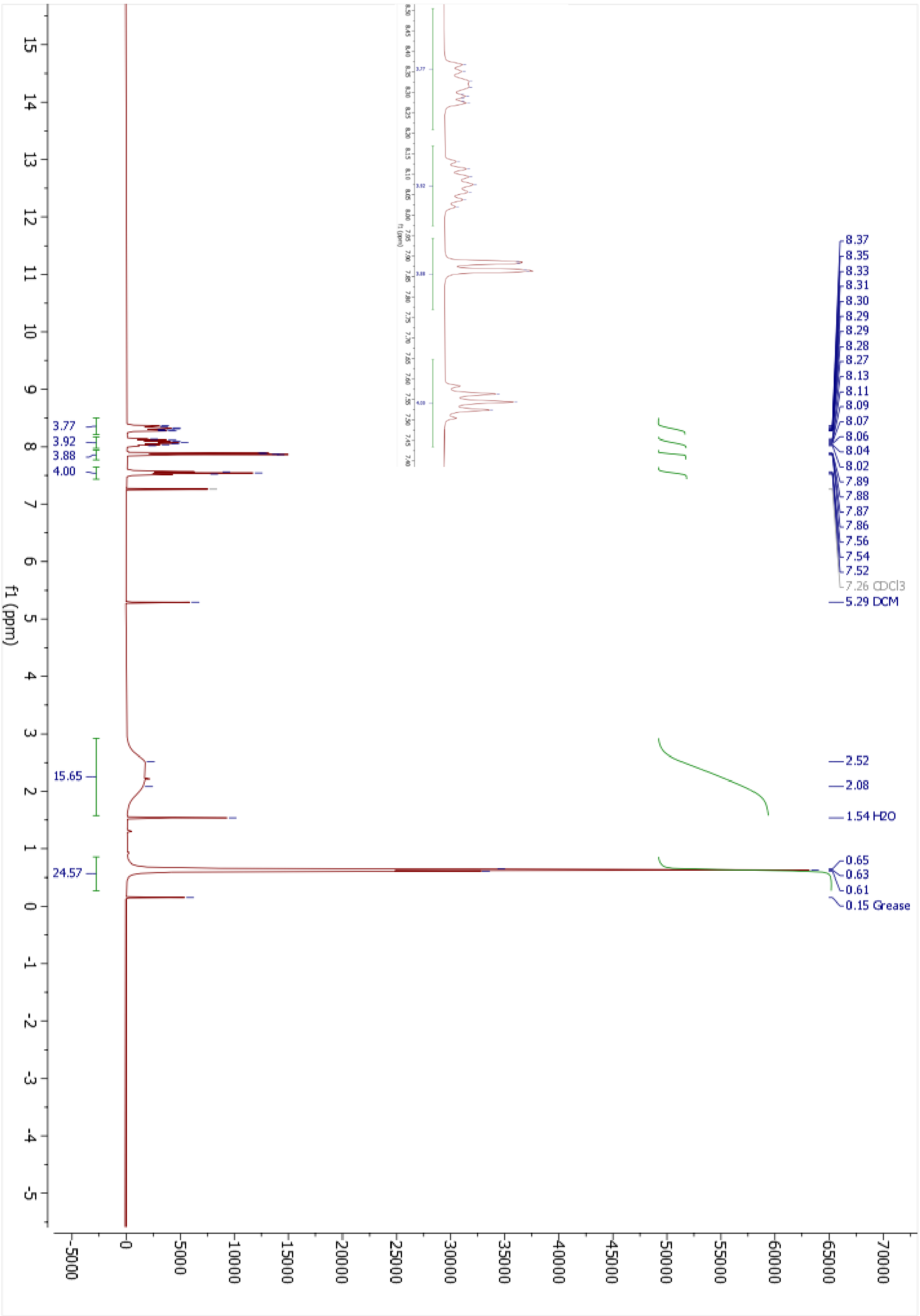

Figure S44: ^{1}H NMR ($CDCl_3$, 600 MHz) of porphyrin **12** including an embedded zoom of the aromatic region (δ = 8.50 – 7.40 ppm).

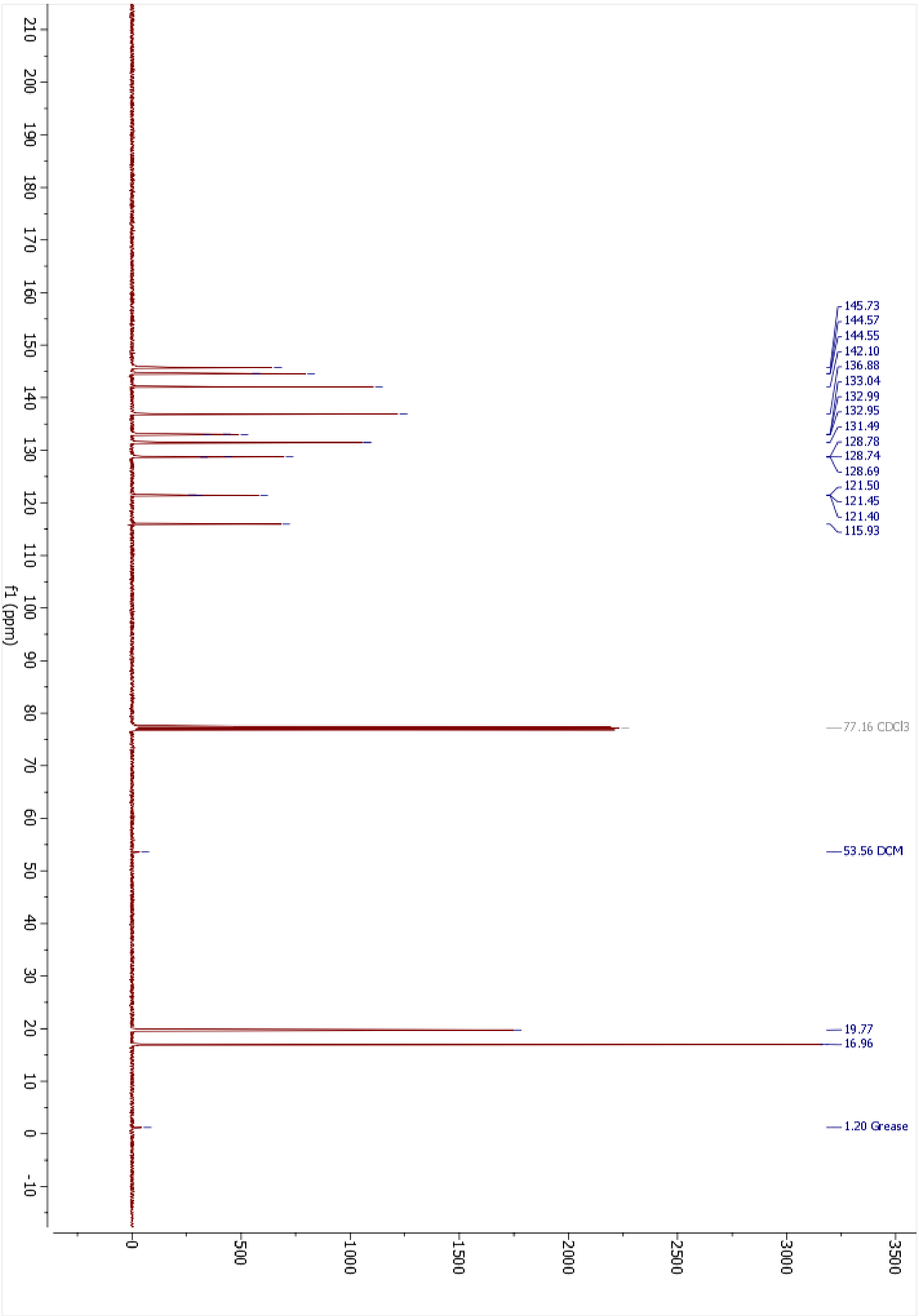

Figure S45: ^{13}C NMR ($CDCl_3$, 150 MHz) of porphyrin **12.**

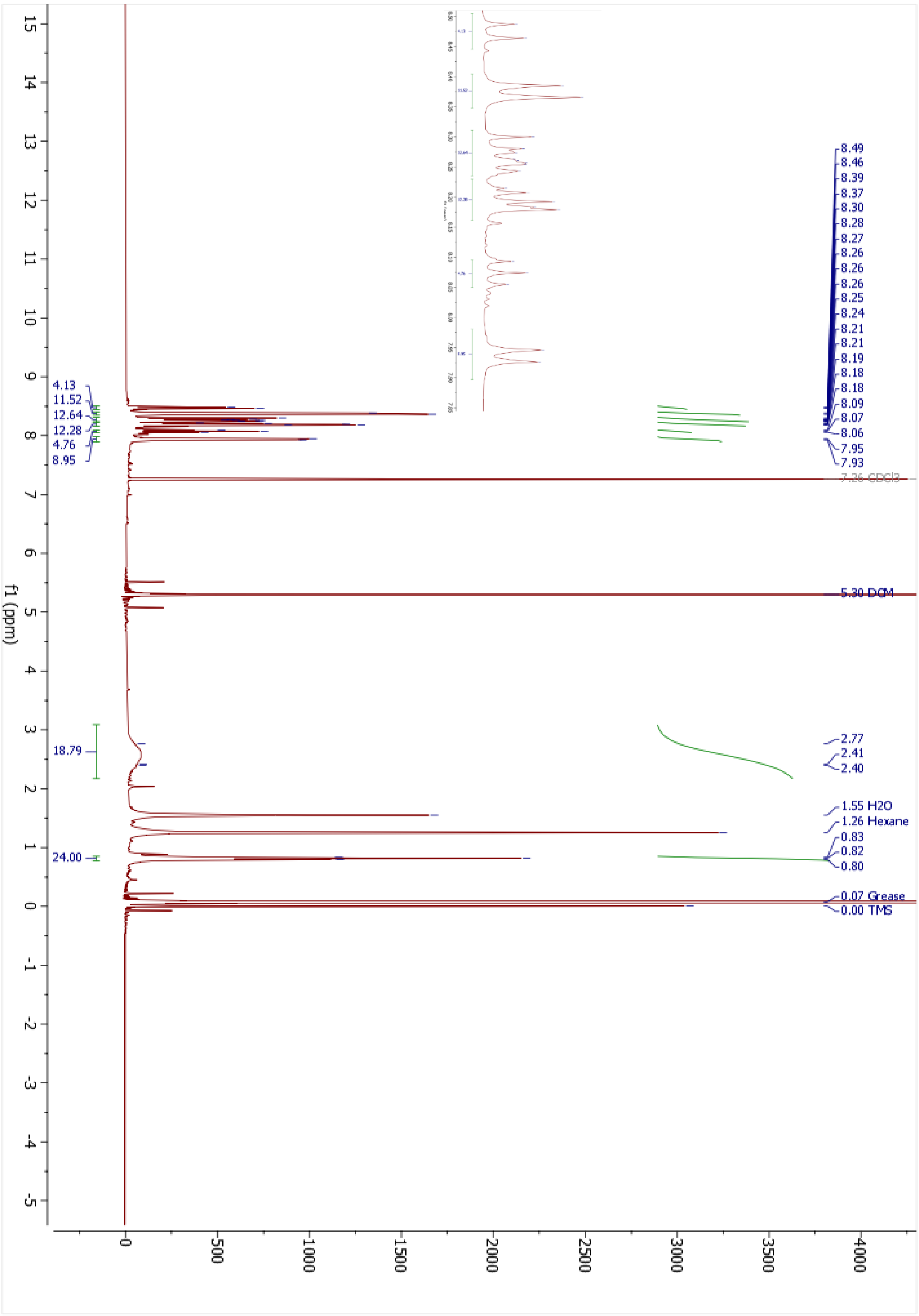

Figure S46: ^{1}H NMR ($CDCl_3$, 400 MHz) of porphyrin **47** including an embedded zoom of the aromatic region (δ = 8.50 – 7.85 ppm).

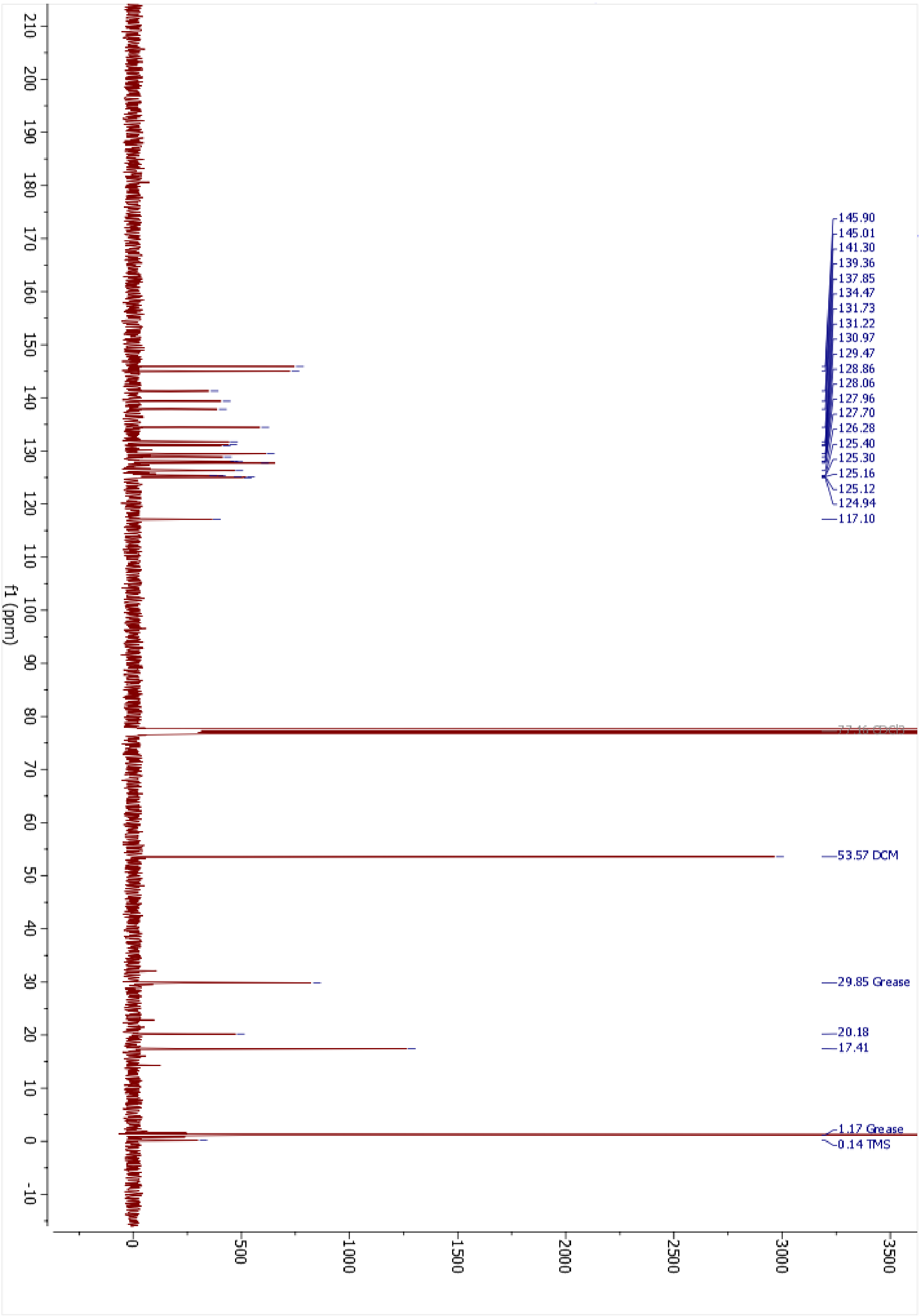

Figure S47: ^{13}C NMR ($CDCl_3$, 150 MHz) of porphyrin **47.**

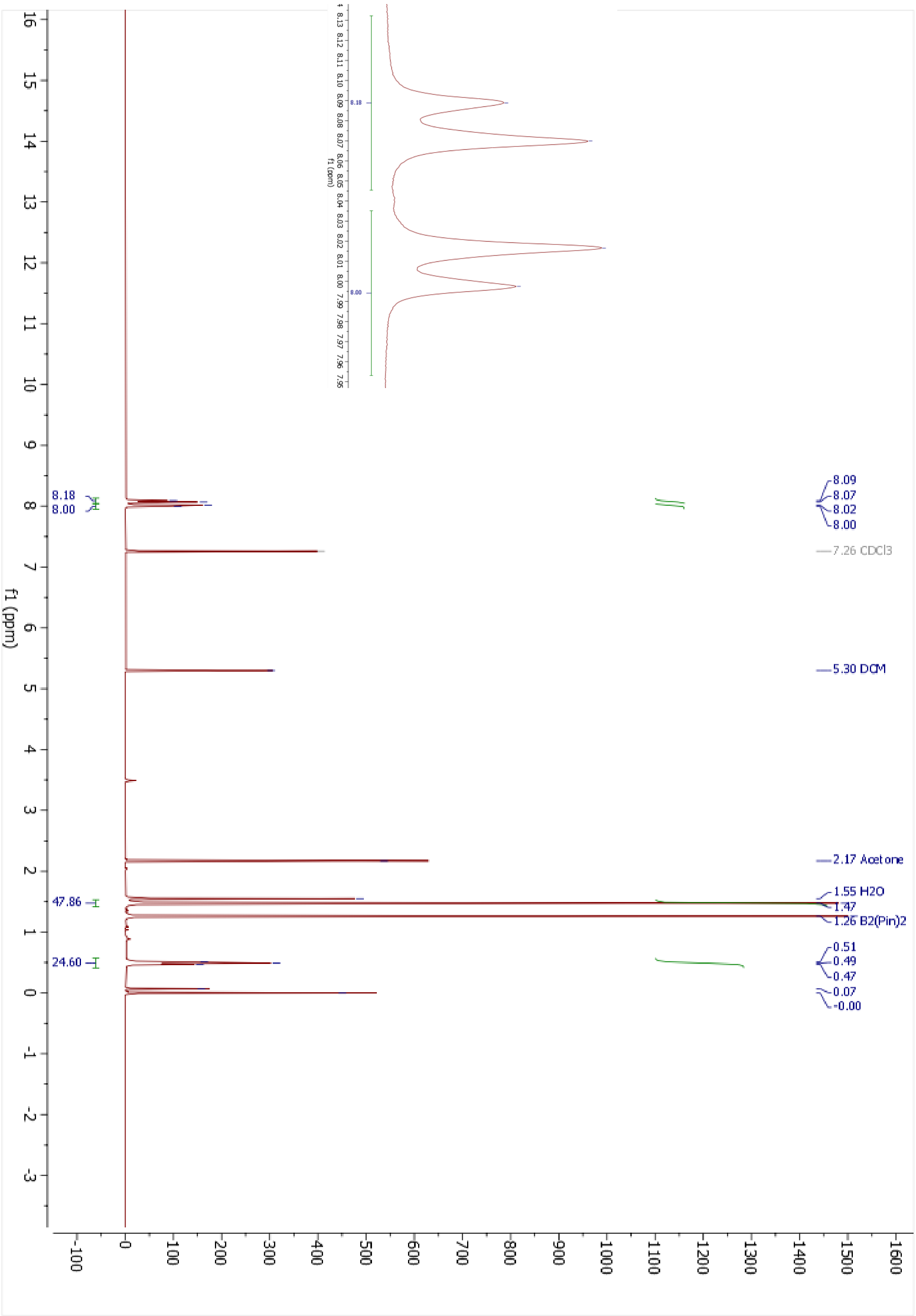

Figure S48: ^{1}H NMR ($CDCl_3$, 400 MHz) of porphyrin **46** including an embedded zoom of the aromatic region (δ = 8.13 – 7.95 ppm).

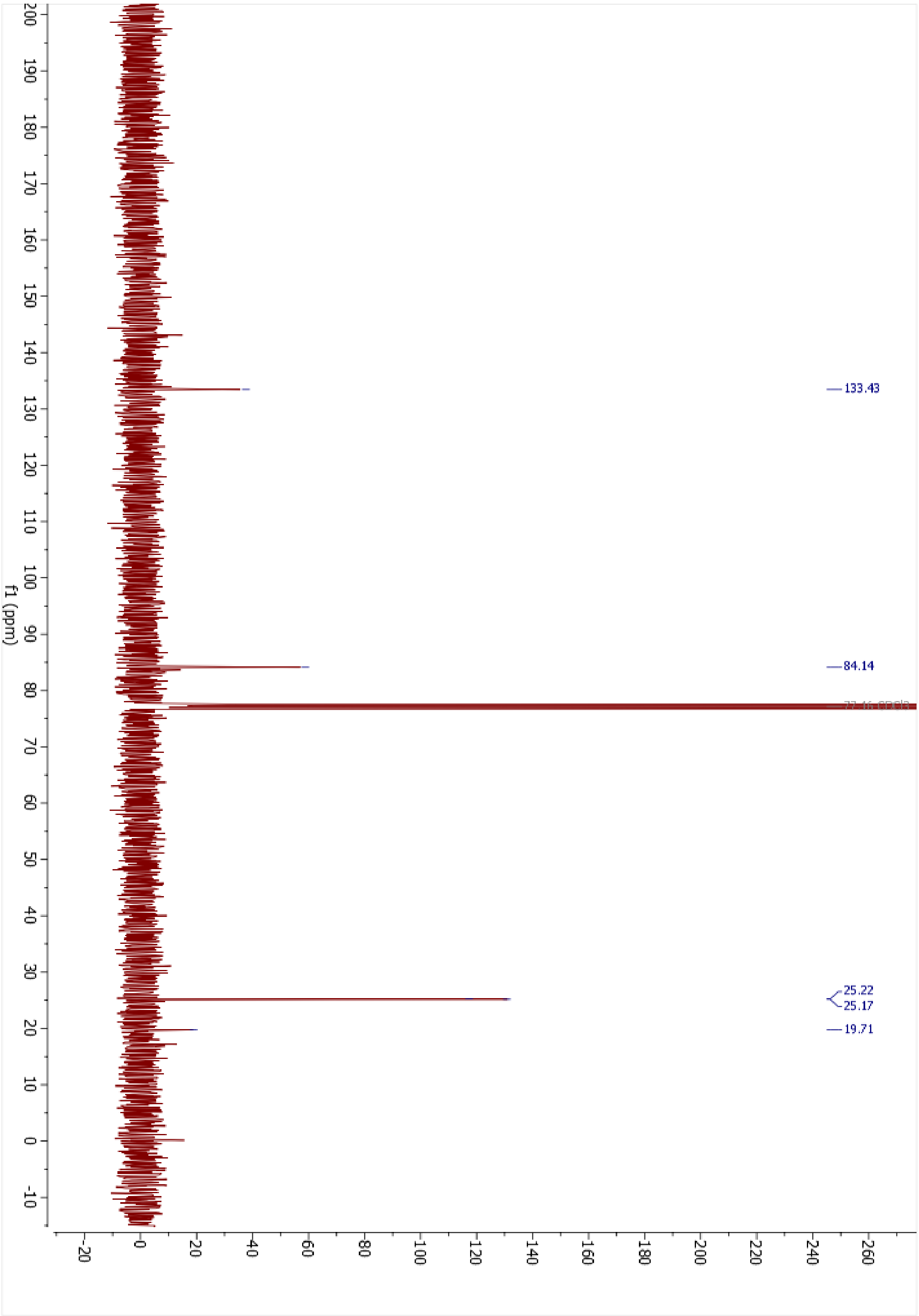

Figure S49: ^{13}C NMR ($CDCl_3$, 100 MHz) of porphyrin **46.**

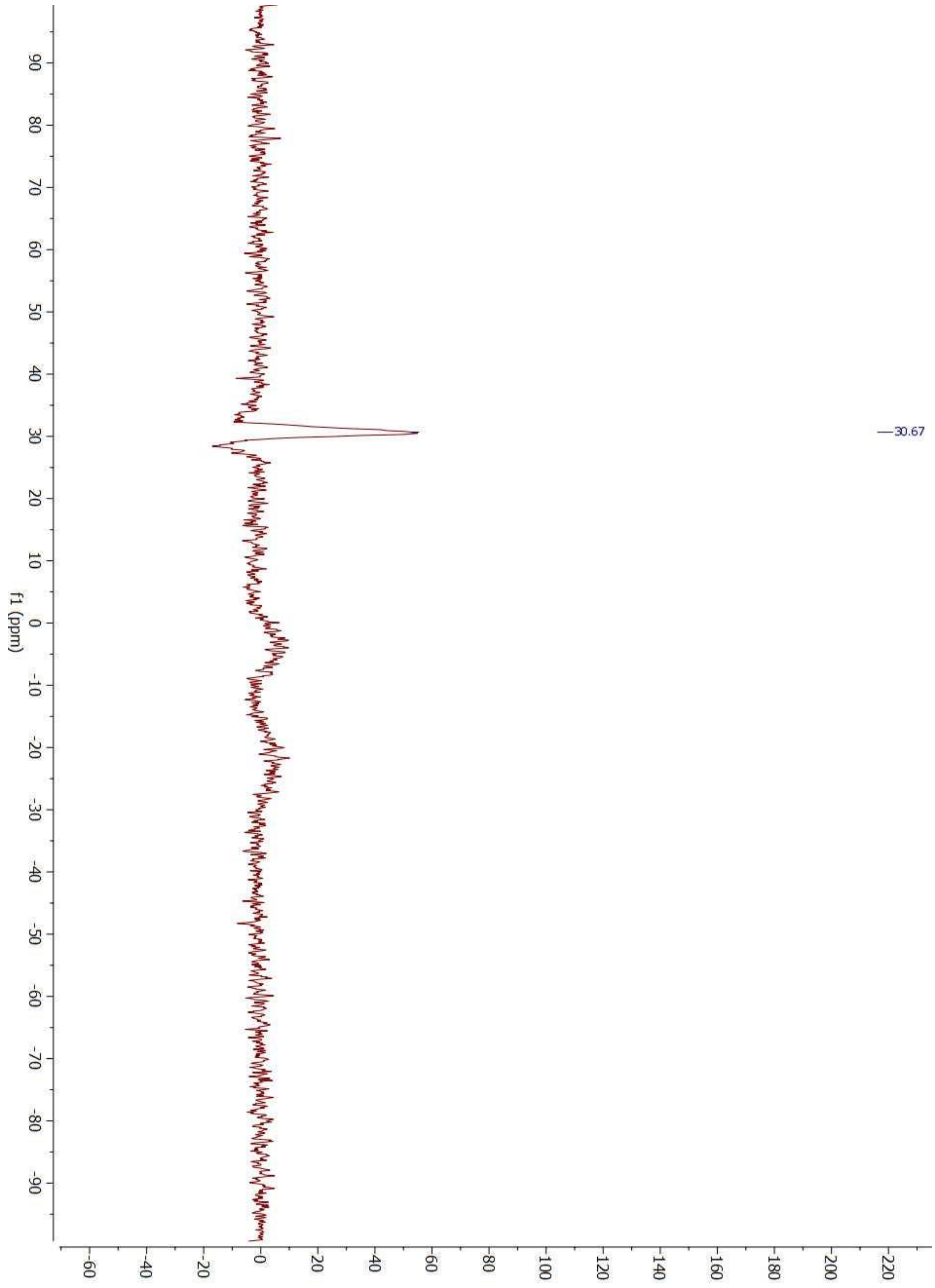

Figure S50: ^{11}B NMR ($CDCl_3$, 128 MHz) of porphyrin **46.**

Variable temperature NMR spectra:

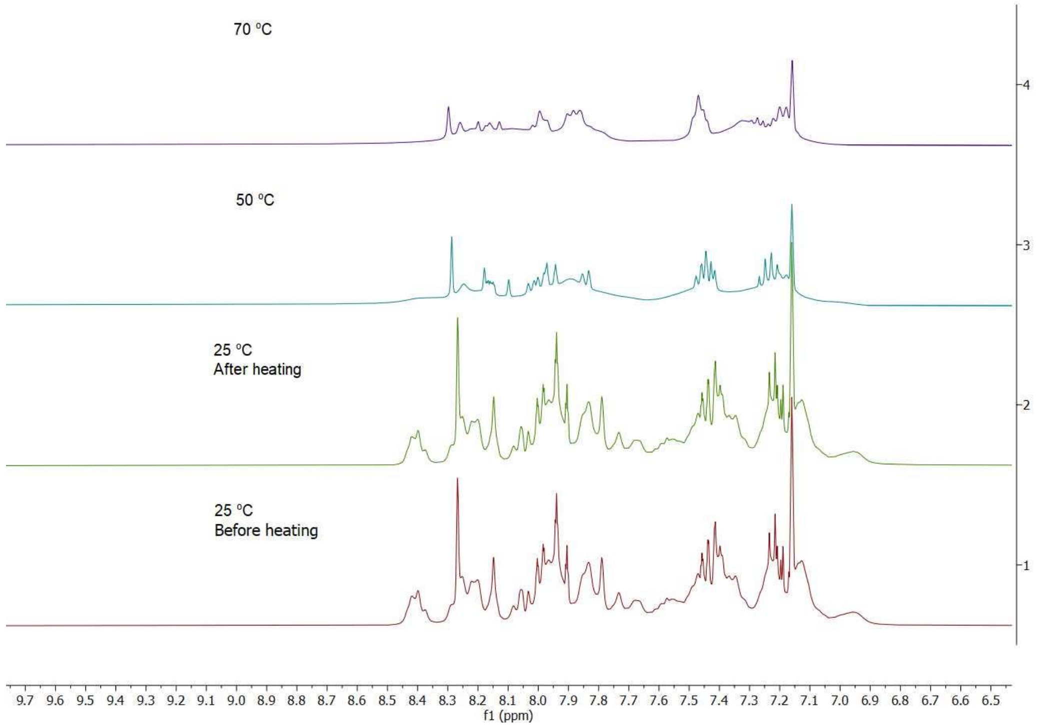

Figure S51: Variable temperature NMR studies of the aromatic region of porphyrin **37** in C_6D_6**.**

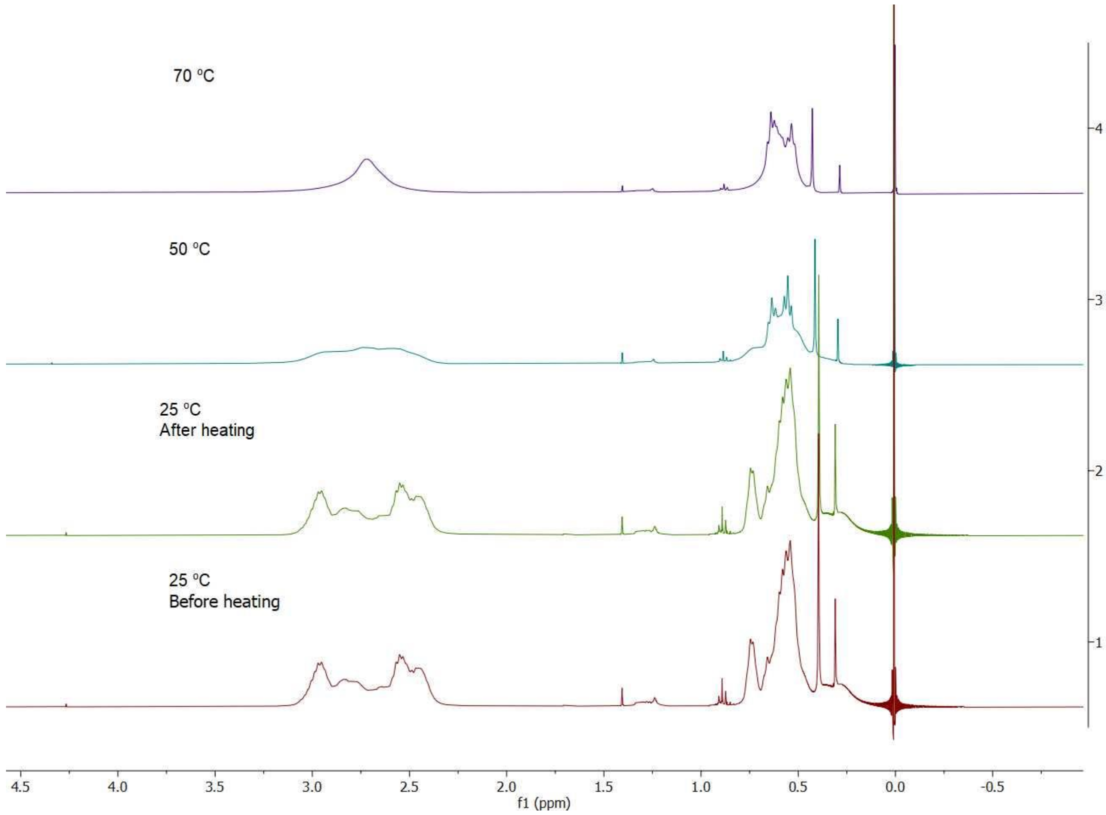

Figure S52: Variable temperature NMR studies of the aliphatic region of porphyrin **37** in C_6D_6.

Mass spectra:

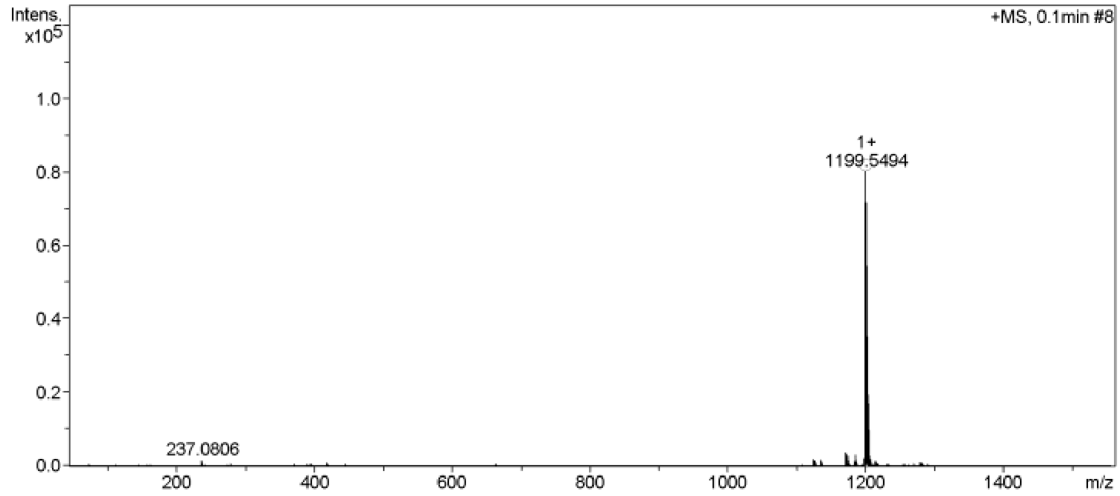

Meas. m/z	#	Ion Formula	m/z	err [mDa]	err [ppm]	rdb	N-Rule	e⁻ Conf	mSigma
1199.549383	1	C84H77N4Ni	1199.549618	0.2	0.2	48.5	ok	even	40.4

Figure S53: HRMS (m/z)-APCI spectrum of porphyrin **26.**

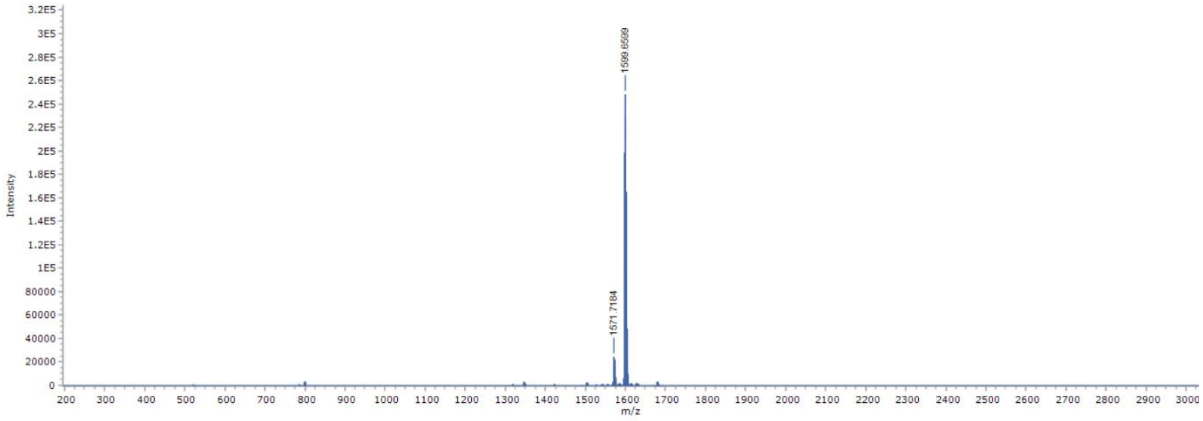

Figure S54: HRMS (m/z)-LIFDI spectrum of porphyrin **27.**

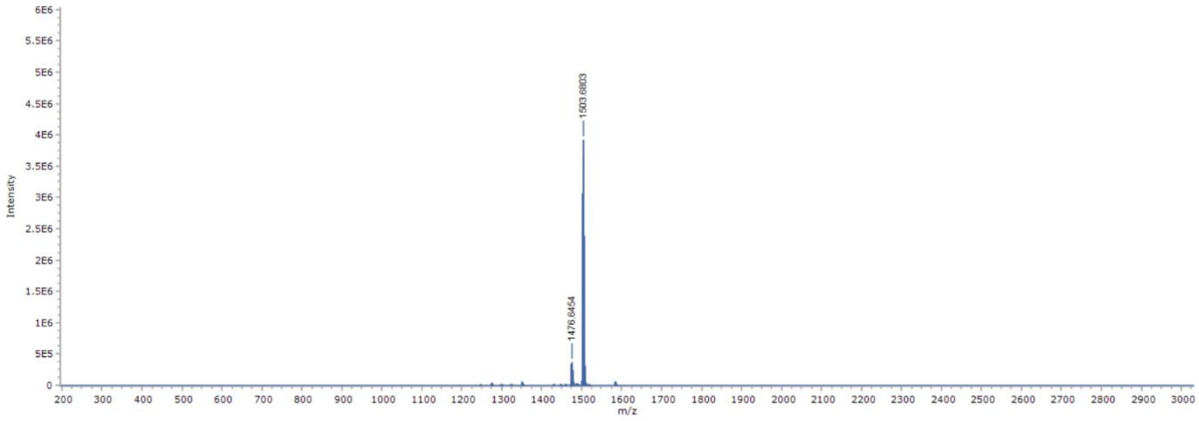

Figure S55: HRMS (m/z)-LIFDI spectrum of porphyrin **28.**

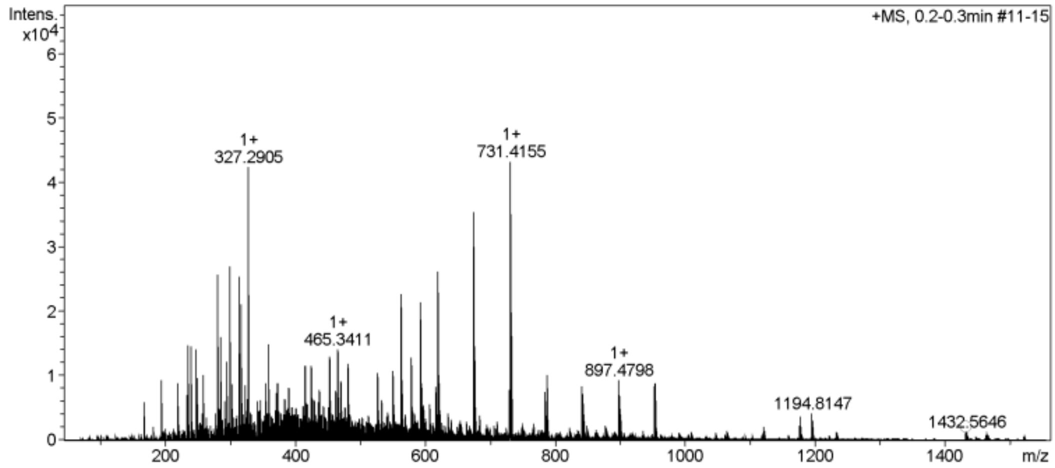

Meas. m/z	#	Ion Formula	m/z	err [mDa]	err [ppm]	rdb	N-Rule	e¯ Conf	mSigma
1431.564450	1	C92H85N4NiO8	1431.571535	7.1	4.9	52.5	ok	even	46.0

Figure S56: HRMS (m/z)-APCI spectrum of porphyrin **29.**

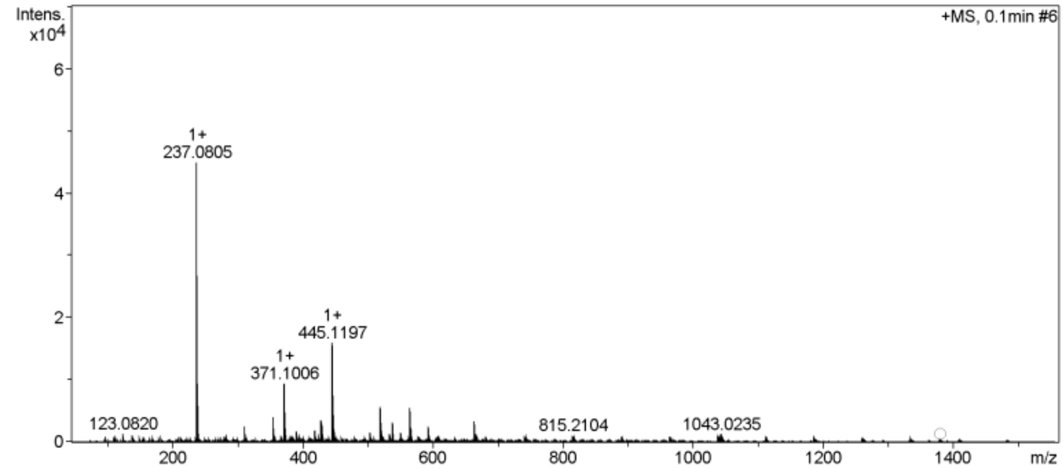

Meas. m/z	#	Ion Formula	m/z	err [mDa]	err [ppm]	rdb	N-Rule	e¯ Conf	mSigma
1379.489111	1	C84H73N8NiO8	1379.489931	-0.8	-0.6	52.5	ok	even	91.4

Figure S57: HRMS (m/z)-APCI spectrum of porphyrin **30.**

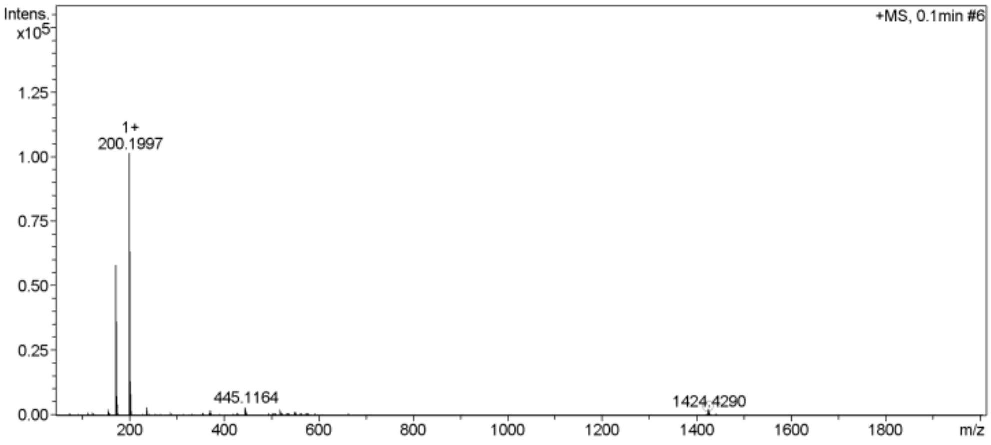

Meas. m/z	#	Ion Formula	m/z	err [mDa]	err [ppm]	rdb	N-Rule	e¯ Conf	mSigma
1423.436525	1	C92H77N4NiS4	1423.437902	-1.4	-1.0	56.5	ok	even	78.7

Figure S58: HRMS (m/z)-APCI spectrum of porphyrin **31.**

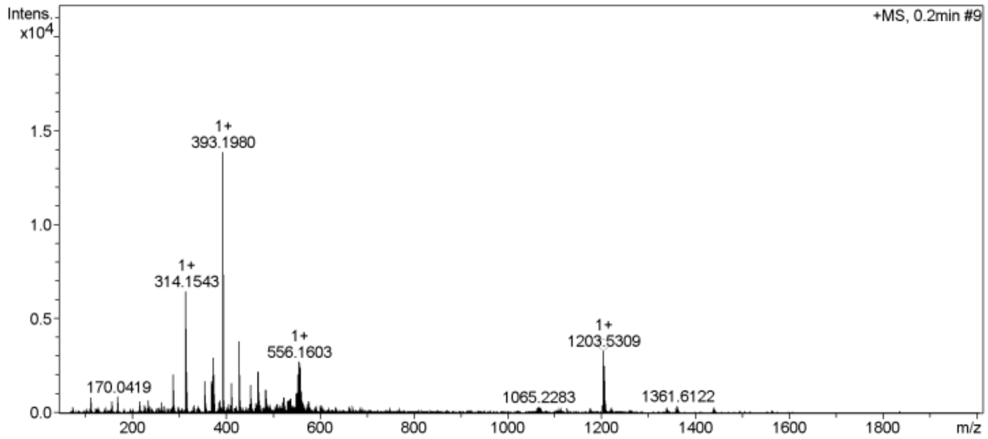

Meas. m/z	#	Ion Formula	m/z	err [mDa]	err [ppm]	rdb	N-Rule	e⁻ Conf	mSigma
1203.530926	1	C80H73N8Ni	1203.530614	-0.3	-0.3	48.5	ok	even	41.0

Figure S59: HRMS (m/z)-APCI spectrum of porphyrin **32.**

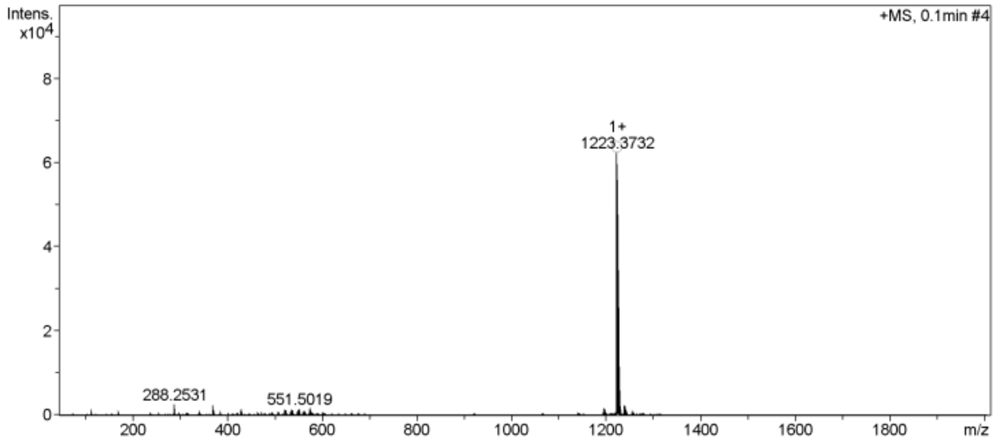

Meas. m/z	#	Ion Formula	m/z	err [mDa]	err [ppm]	rdb	N-Rule	e⁻ Conf	mSigma
1223.373174	1	C76H69N4NiS4	1223.375302	-2.1	-1.7	44.5	ok	even	50.3

Figure S60: HRMS (m/z)-APCI spectrum of porphyrin **33.**

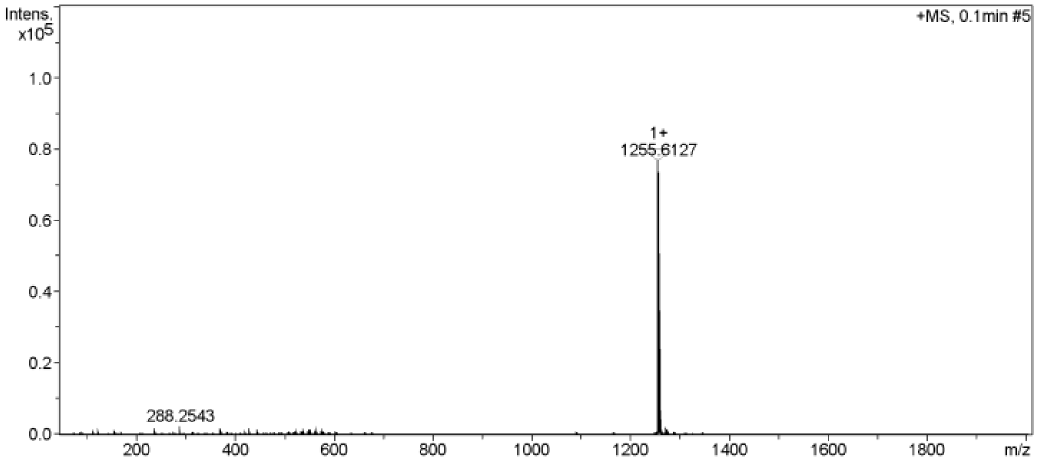

Meas. m/z	#	Ion Formula	m/z	err [mDa]	err [ppm]	rdb	N-Rule	e⁻ Conf	mSigma
1255.612680	1	C88H85N4Ni	1255.612218	-0.5	-0.4	48.5	ok	even	53.4

Figure S61: HRMS (m/z)-APCI spectrum of porphyrin **34.**

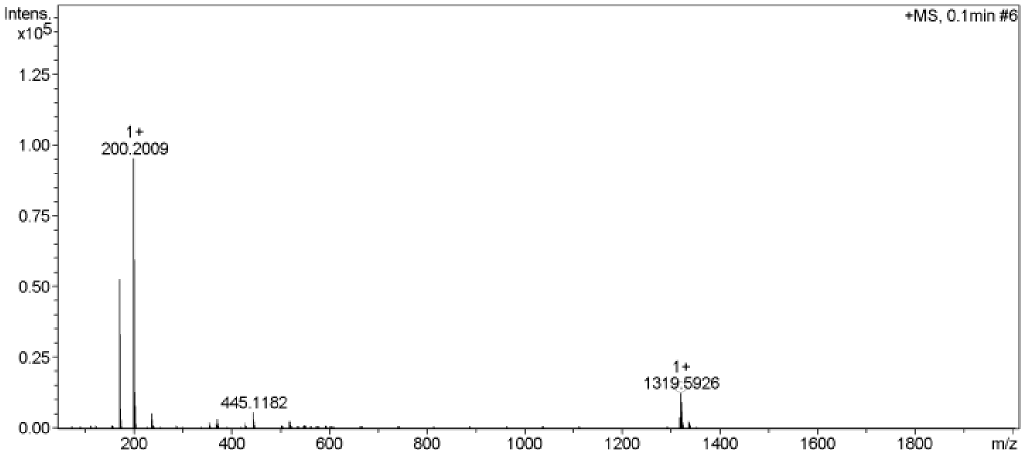

Meas. m/z	#	Ion Formula	m/z	err [mDa]	err [ppm]	rdb	N-Rule	e¯ Conf	mSigma
1319.592621	1	C88H85N4NiO4	1319.591877	-0.7	-0.6	48.5	ok	even	74.7

Figure S62: HRMS (m/z)-APCI spectrum of porphyrin **35.**

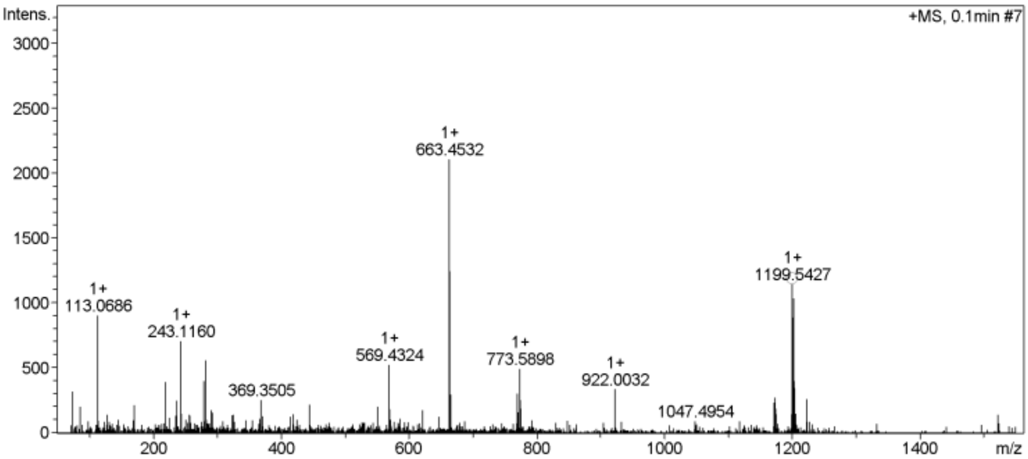

Figure S63: HRMS (m/z)-APCI spectrum of porphyrin **36.**

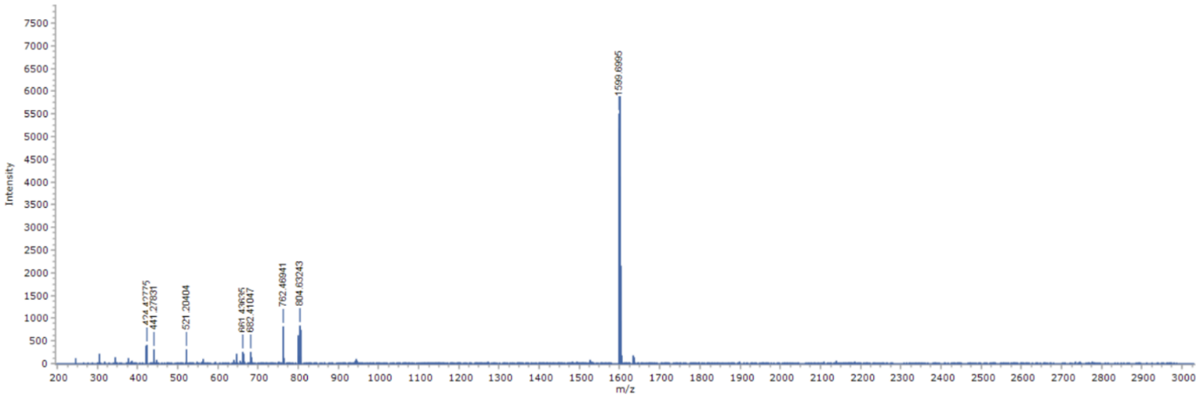

Figure S64: HRMS (m/z)-LIFDI spectrum of porphyrin **37.**

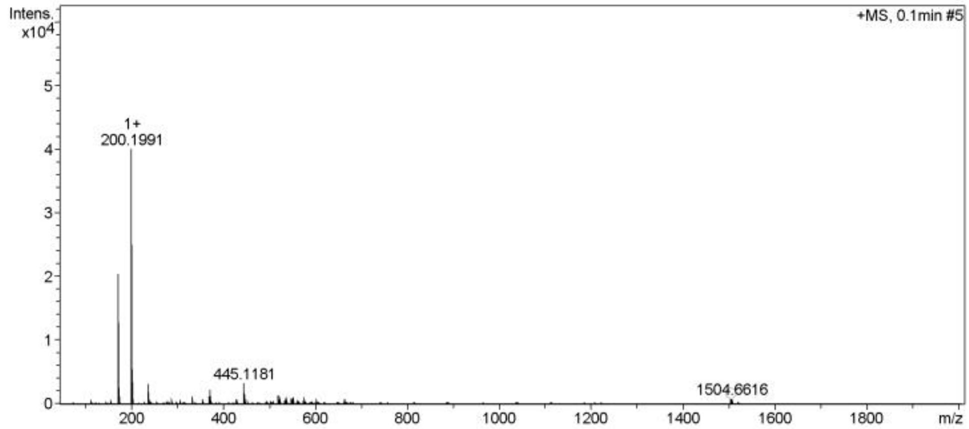

Meas. m/z	#	Ion Formula	m/z	err [mDa]	err [ppm]	rdb	N-Rule	e⁻ Conf	mSigma
1503.671858	1	C108H93N4Ni	1503.674818	-3.0	-2.0	64.5	ok	even	85.6

Figure S65: HRMS (m/z)-APCI spectrum of porphyrin **38.**

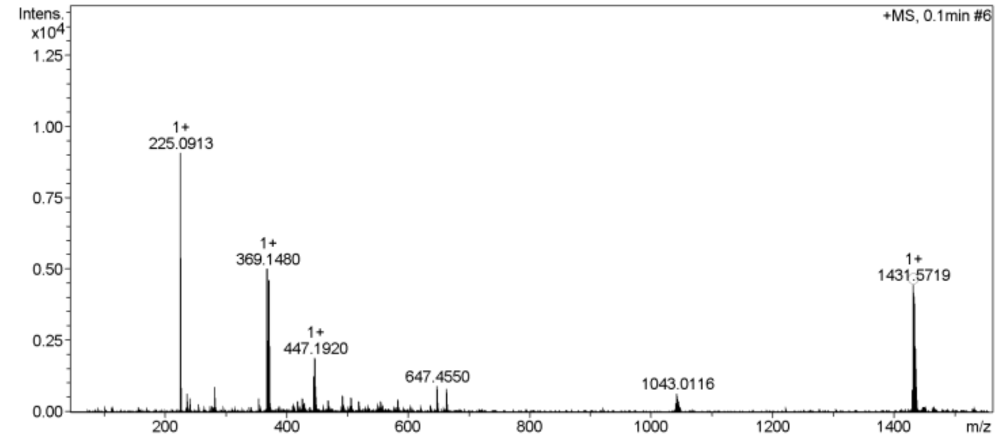

Meas. m/z	#	Ion Formula	m/z	err [mDa]	err [ppm]	rdb	N-Rule	e⁻ Conf	mSigma
1431.571921	1	C92H85N4NiO8	1431.571535	0.4	0.3	52.5	ok	even	66.5

Figure S66: HRMS (m/z)-APCI spectrum of porphyrin **39.**

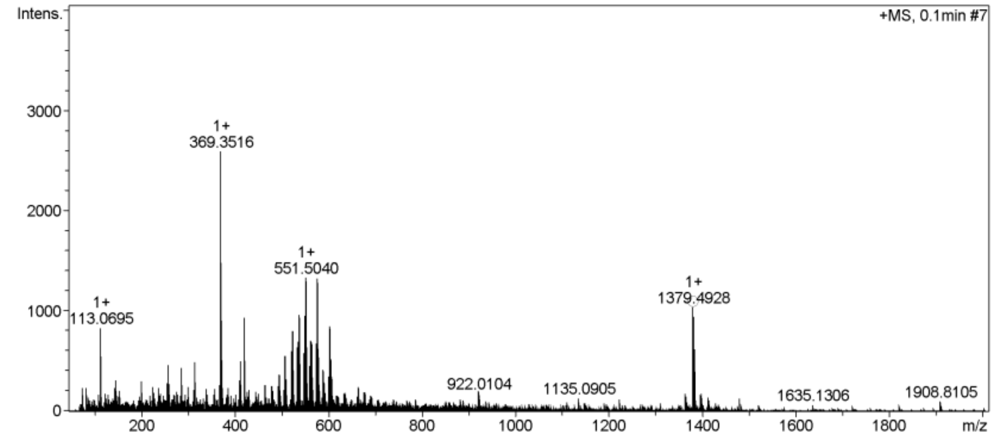

Meas. m/z	#	Ion Formula	m/z	err [mDa]	err [ppm]	rdb	N-Rule	e⁻ Conf	mSigma
1379.492818	1	C84H73N8NiO8	1379.489931	-2.9	-2.1	52.5	ok	even	31.8

Figure S67: HRMS (m/z)-APCI spectrum of porphyrin **40.**

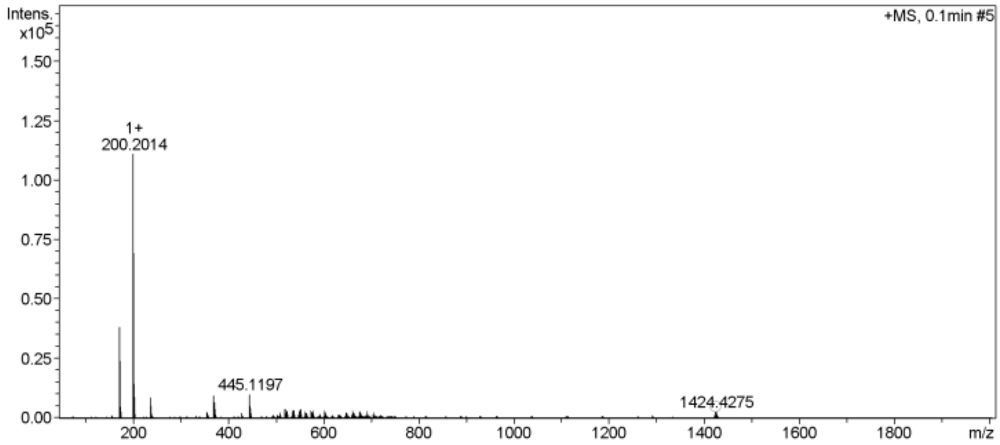

Meas. m/z	#	Ion Formula	m/z	err [mDa]	err [ppm]	rdb	N-Rule	e¯ Conf	mSigma
1423.435867	1	C92H77N4NiS4	1423.437902	-2.0	-1.4	56.5	ok	even	32.6

Figure S68: HRMS (m/z)-APCI spectrum of porphyrin **41.**

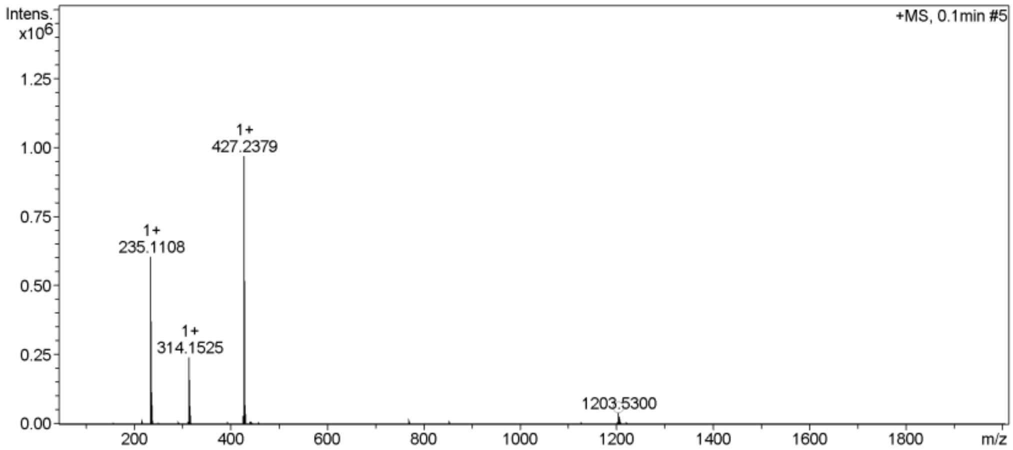

Meas. m/z	#	Ion Formula	m/z	err [mDa]	err [ppm]	rdb	N-Rule	e¯ Conf	mSigma
1203.530050	1	C80H73N8Ni	1203.530614	0.6	0.5	48.5	ok	even	42.5

Figure S69: HRMS (m/z)-APCI spectrum of porphyrin **42.**

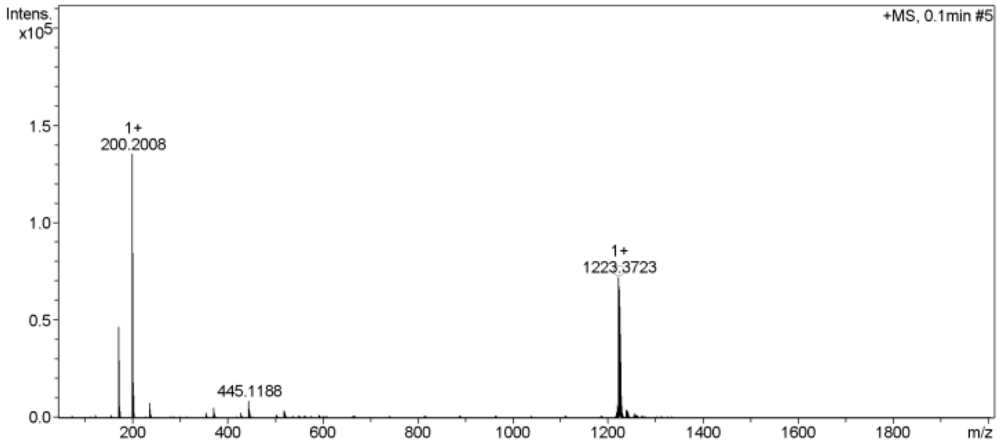

Meas. m/z	#	Ion Formula	m/z	err [mDa]	err [ppm]	rdb	N-Rule	e¯ Conf	mSigma
1223.372263	1	C76H69N4NiS4	1223.375302	-3.0	-2.5	44.5	ok	even	54.2

Figure S70: HRMS (m/z)-APCI spectrum of porphyrin **43.**

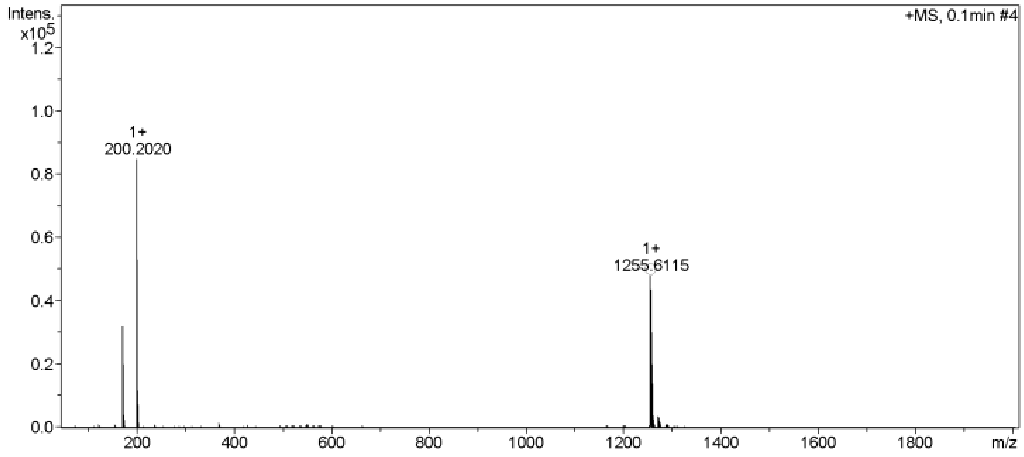

Meas. m/z	#	Ion Formula	m/z	err [mDa]	err [ppm]	rdb	N-Rule	e⁻ Conf	mSigma
1255.611450	1	C88H85N4Ni	1255.612218	0.8	0.6	48.5	ok	even	67.0

Figure S71: HRMS (m/z)-APCI spectrum of porphyrin **44.**

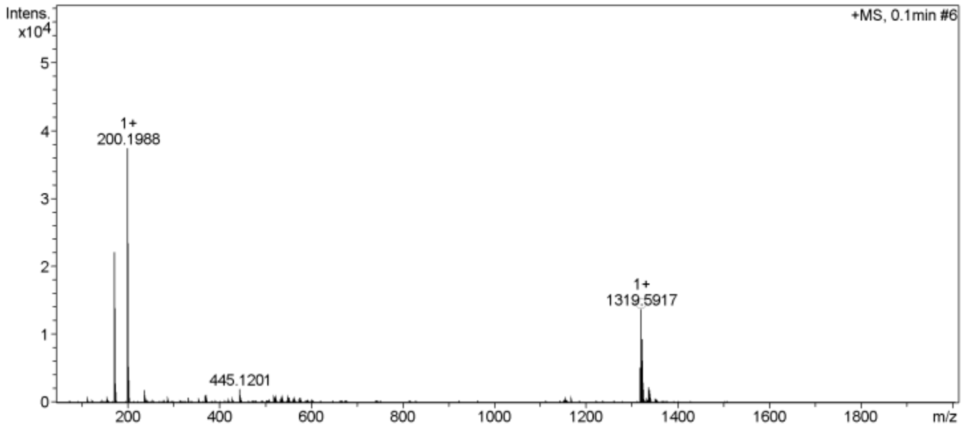

Meas. m/z	#	Ion Formula	m/z	err [mDa]	err [ppm]	rdb	N-Rule	e⁻ Conf	mSigma
1319.591740	1	C88H85N4NiO4	1319.591877	-0.1	-0.1	48.5	ok	even	96.0

Figure S72: HRMS (m/z)-APCI spectrum of porphyrin **45.**

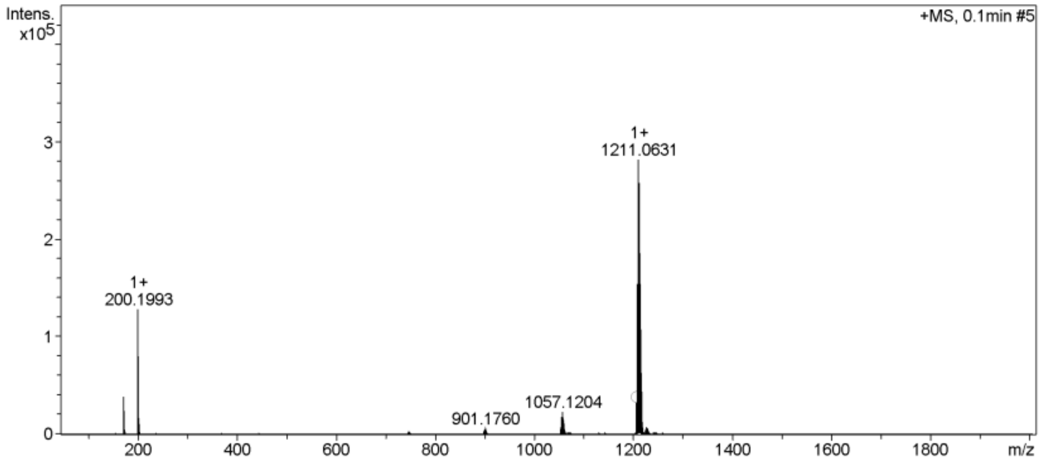

Meas. m/z	#	Ion Formula	m/z	err [mDa]	err [ppm]	rdb	N-Rule	e⁻ Conf	mSigma
1207.067446	1	C60H57Br4N4Ni	1207.066466	1.0	0.8	32.5	ok	even	46.3

Figure S73: HRMS (m/z)-APCI spectrum of porphyrin **11.**

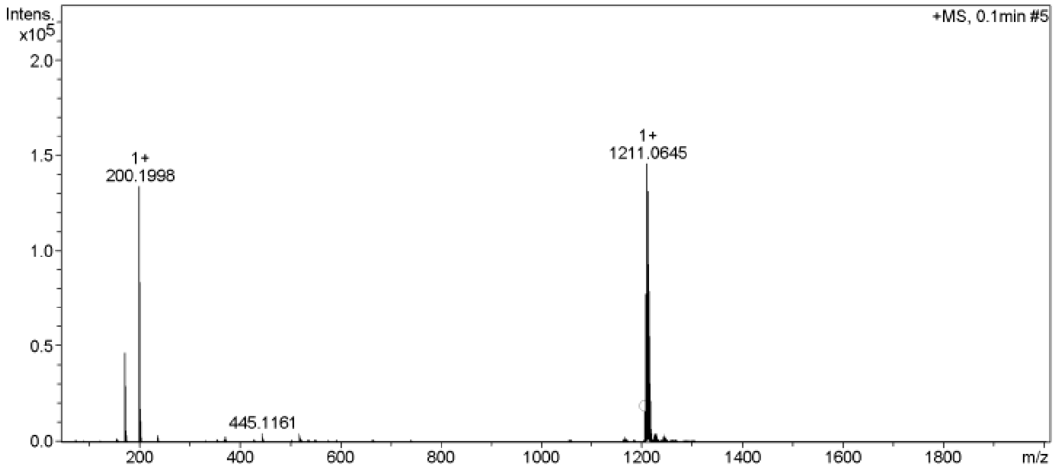

Meas. m/z	#	Ion Formula	m/z	err [mDa]	err [ppm]	rdb	N-Rule	e⁻ Conf	mSigma
1207.065800	1	C60H57Br4N4Ni	1207.066466	-0.7	-0.6	32.5	ok	even	54.8

Figure S74: HRMS (m/z)-APCI spectrum of porphyrin **12.**

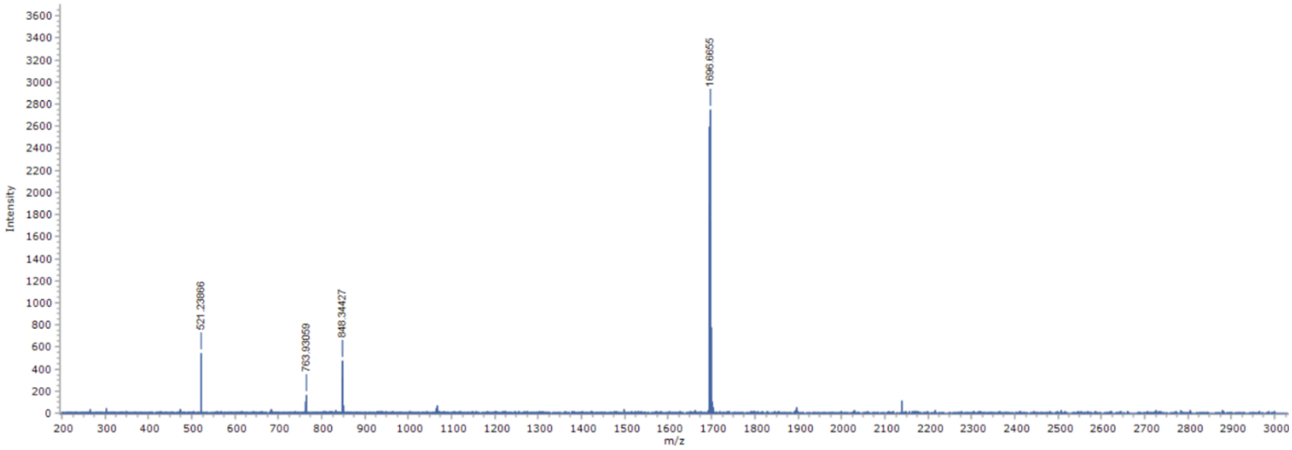

Figure S75: HRMS (m/z)-LIFDI spectrum of porphyrin **47.**

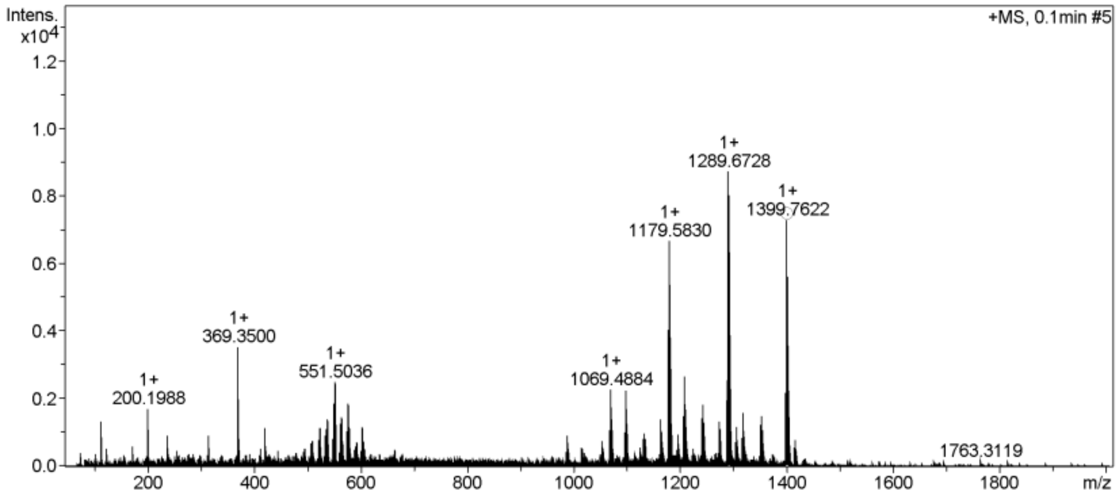

Meas. m/z	#	Ion Formula	m/z	err [mDa]	err [ppm]	rdb	N-Rule	e⁻ Conf	mSigma
1399.762155	1	C84H105B4N4NiO8	1399.769464	3.1	2.2	36.5	ok	even	68.2

Figure S76: HRMS (m/z)-APCI spectrum of porphyrin **46.**

UV–vis spectra:

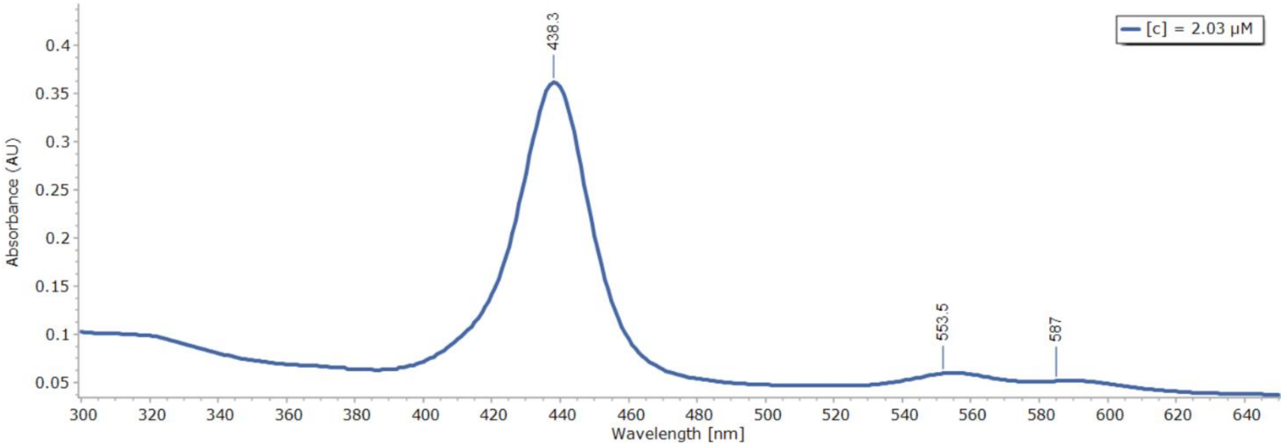

Figure S77: UV–vis spectrum of porphyrin **26** in CH_2Cl_2.

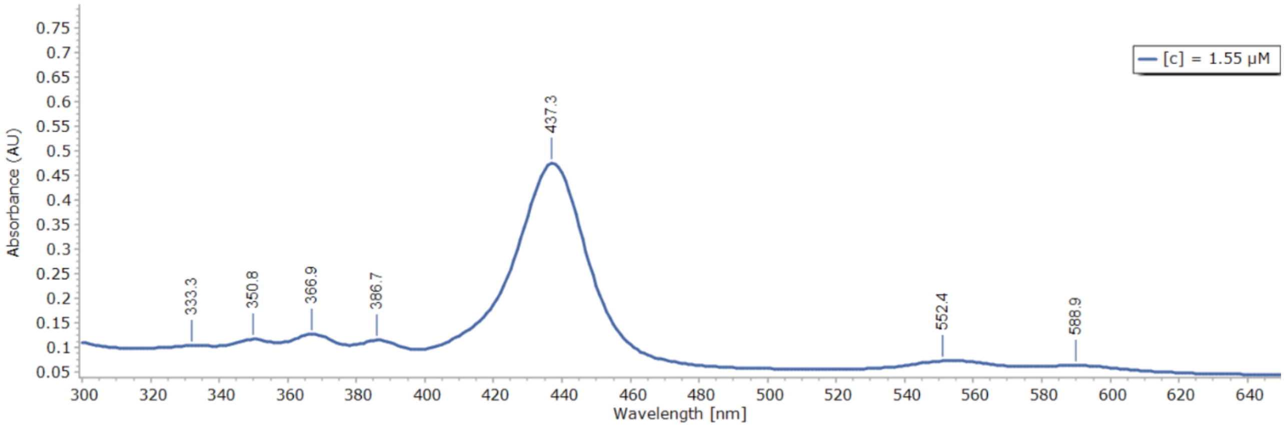

Figure S78: UV–vis spectrum of porphyrin **27** in CH_2Cl_2.

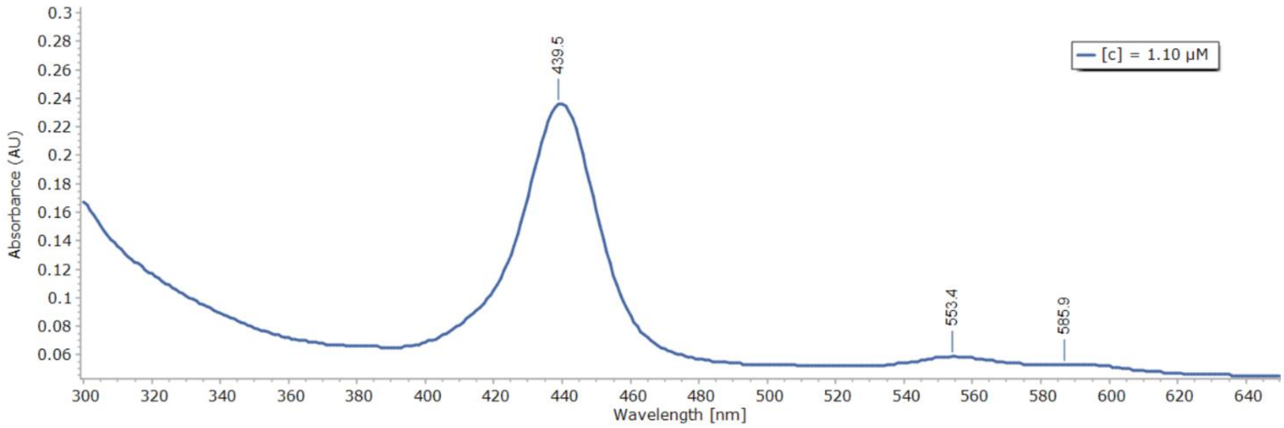

Figure S79: UV–vis spectrum of porphyrin **28** in CH_2Cl_2.

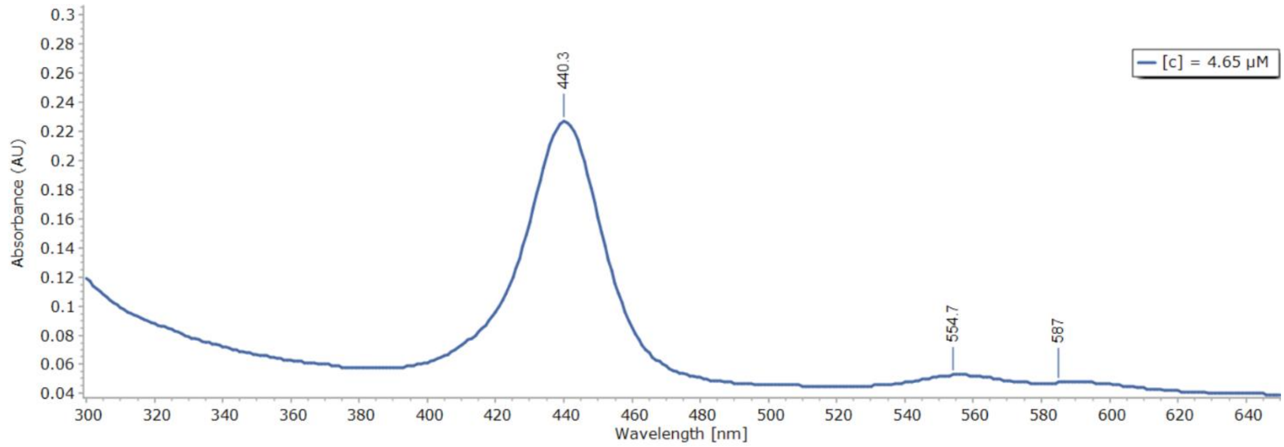

Figure S80: UV–vis spectrum of porphyrin **29** in CH_2Cl_2**.**

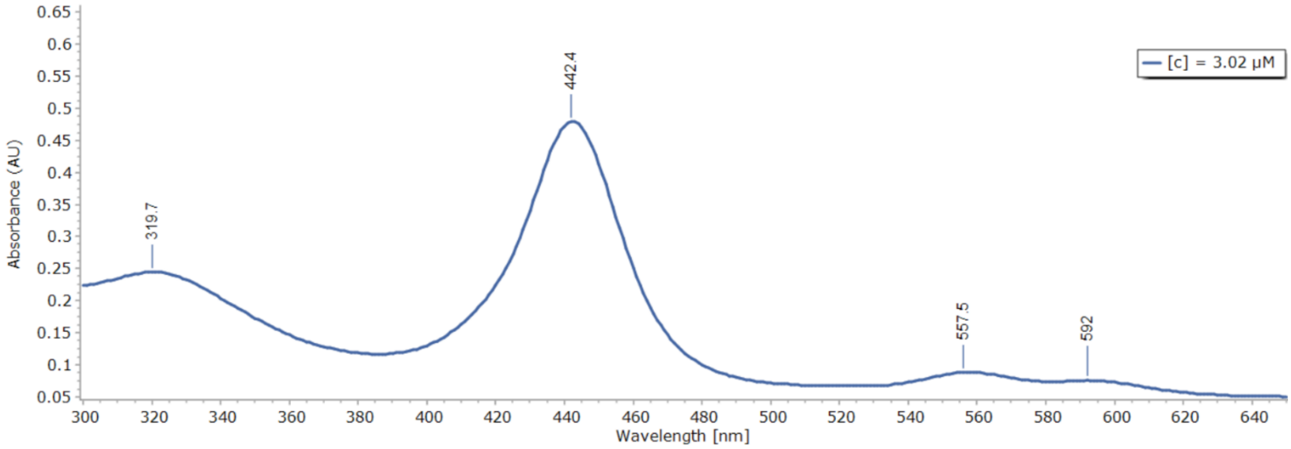

Figure S81: UV–vis spectrum of porphyrin **30** in CH_2Cl_2**.**

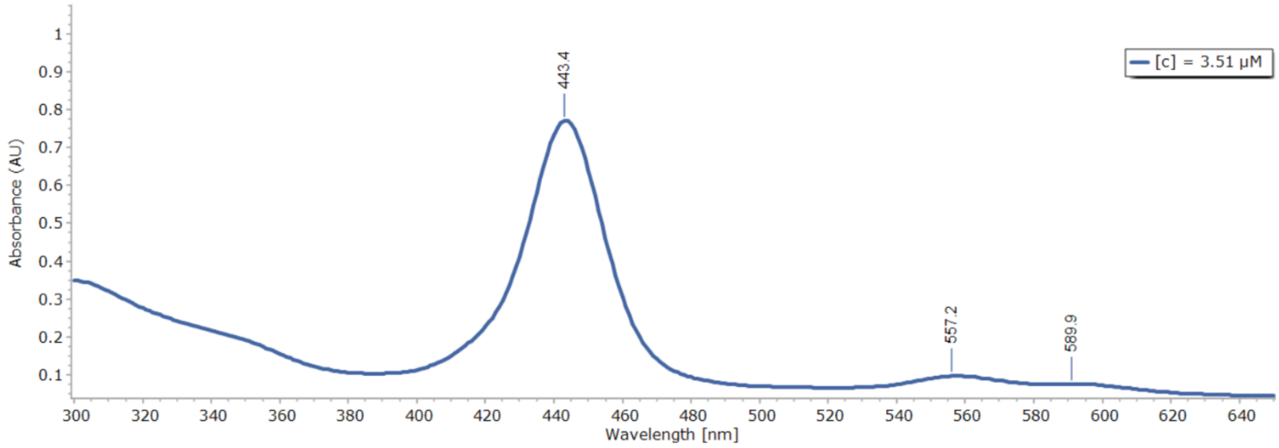

Figure S82: UV–vis spectrum of porphyrin **31** in CH_2Cl_2**.**

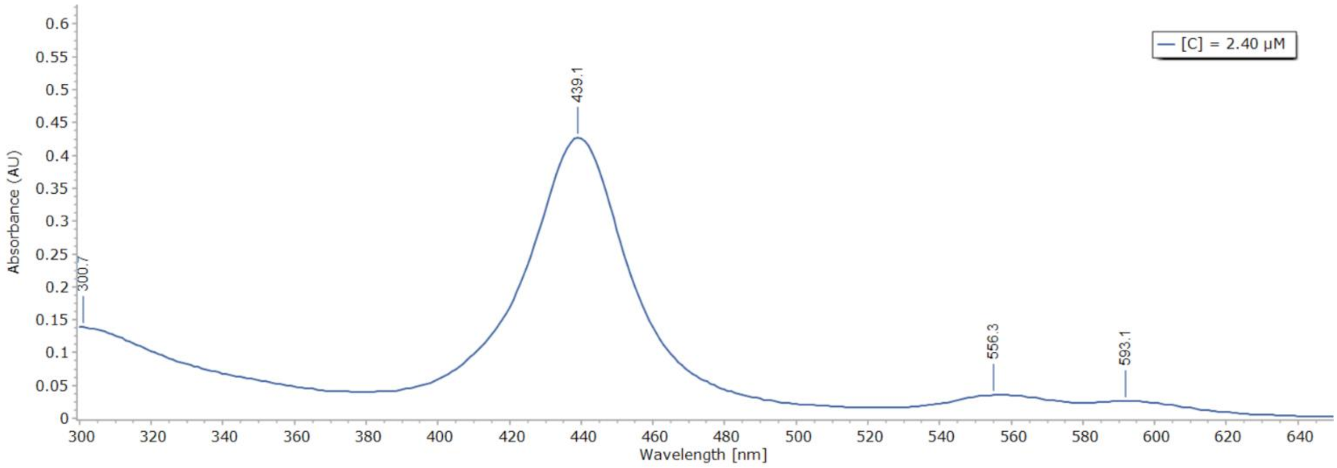

Figure S83: UV–vis spectrum of porphyrin **32** in CH_2Cl_2.

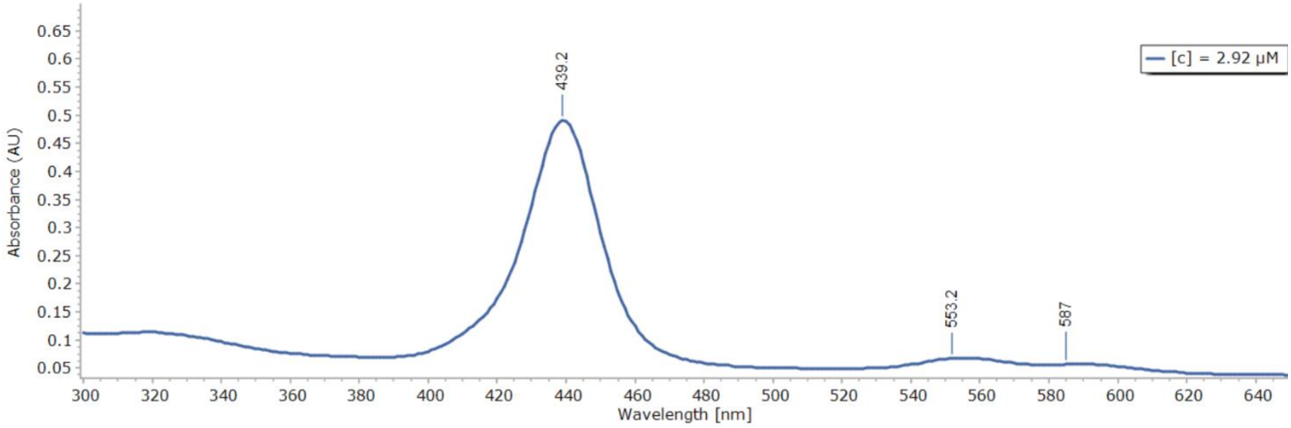

Figure S84: UV–vis spectrum of porphyrin **33** at in CH_2Cl_2.

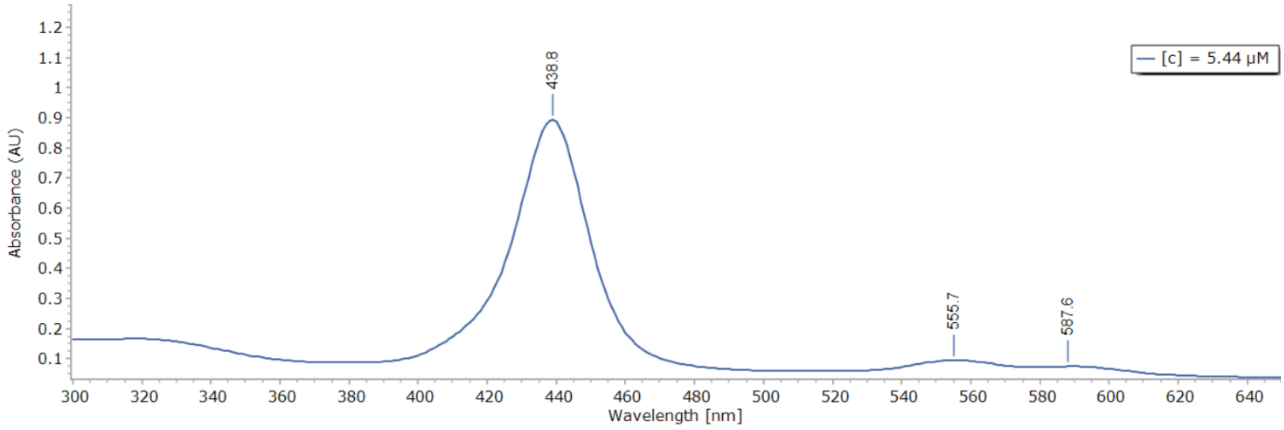

Figure S85: UV–vis spectrum of porphyrin **34** in CH_2Cl_2.

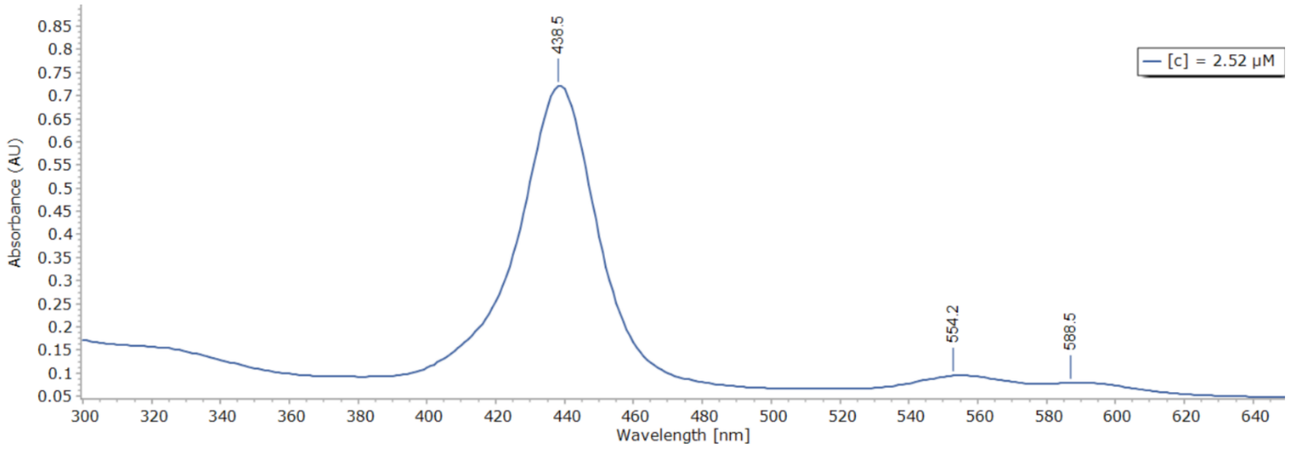

Figure S86: UV–vis spectrum of porphyrin **35** in CH_2Cl_2**.**

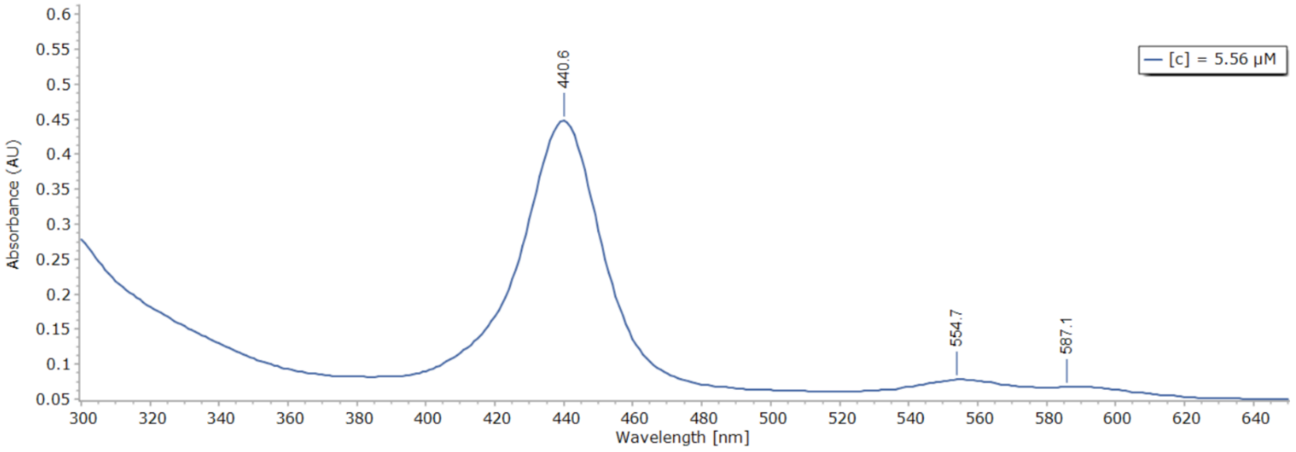

Figure S87: UV–vis spectrum of porphyrin **36** in CH_2Cl_2**.**

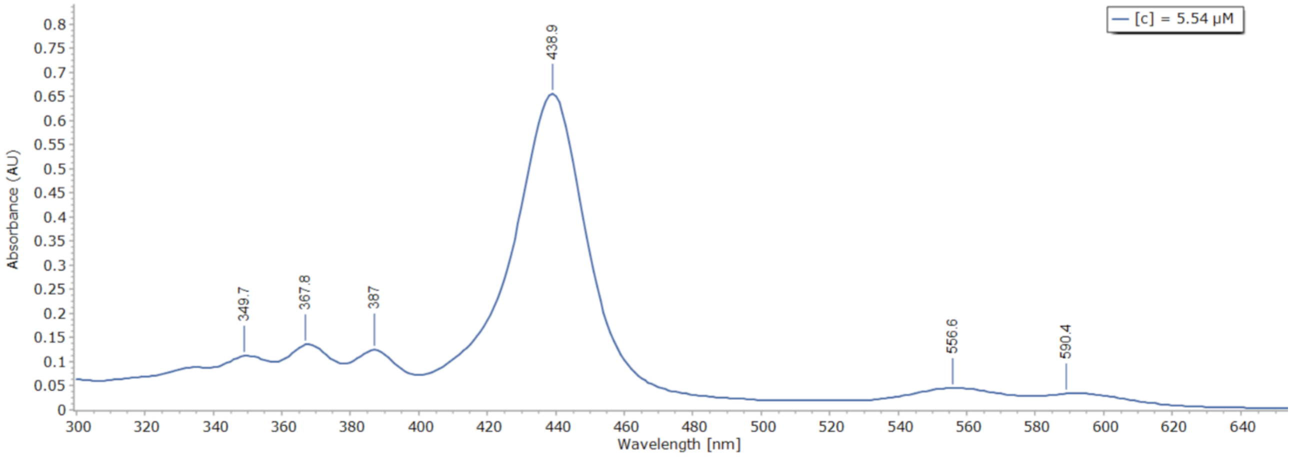

Figure S88: UV–vis spectrum of porphyrin **37** in CH_2Cl_2**.**

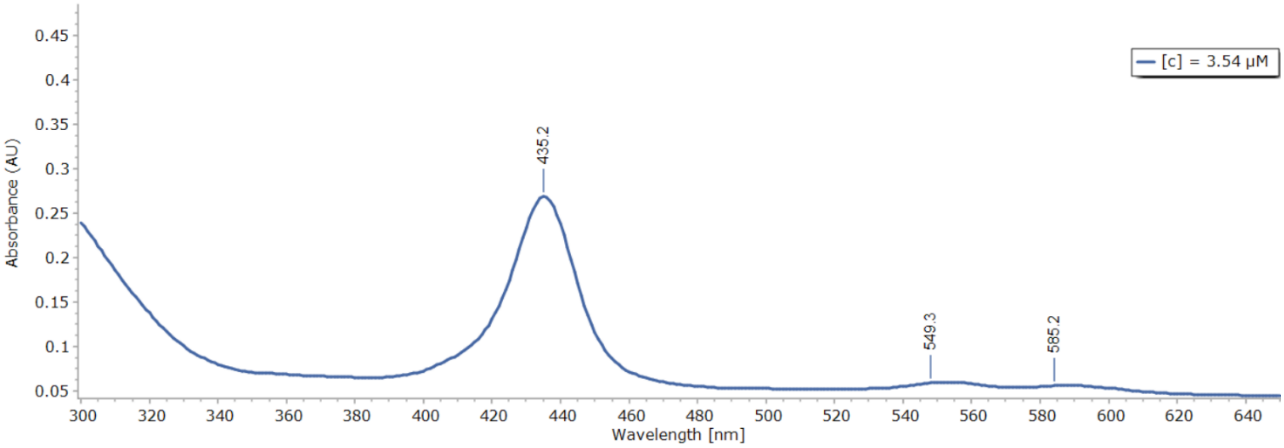

Figure S89: UV–vis spectrum of porphyrin **38** in CH_2Cl_2**.**

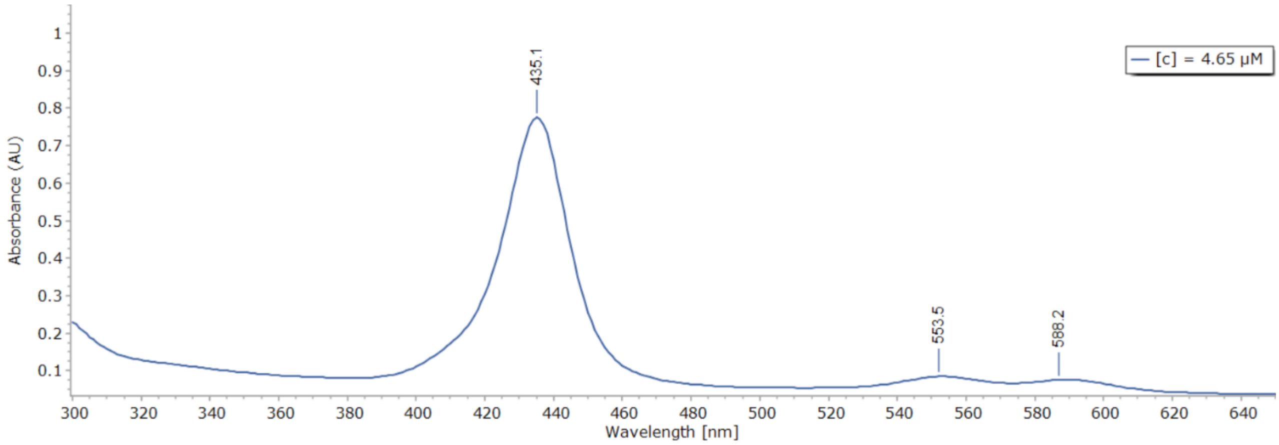

Figure S90: UV–vis spectrum of porphyrin **39** in CH_2Cl_2**.**

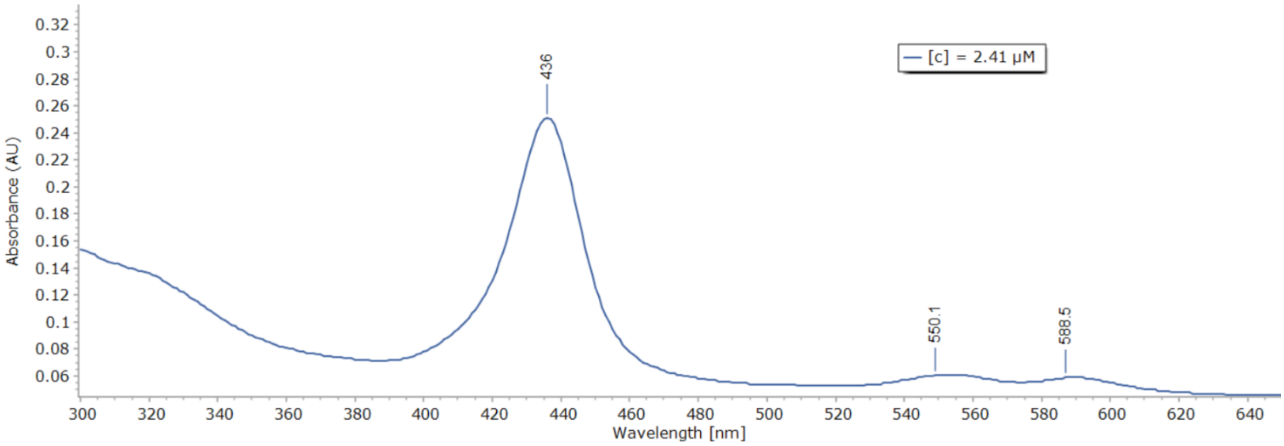

Figure S91: UV–vis spectrum of porphyrin **40** in CH_2Cl_2**.**

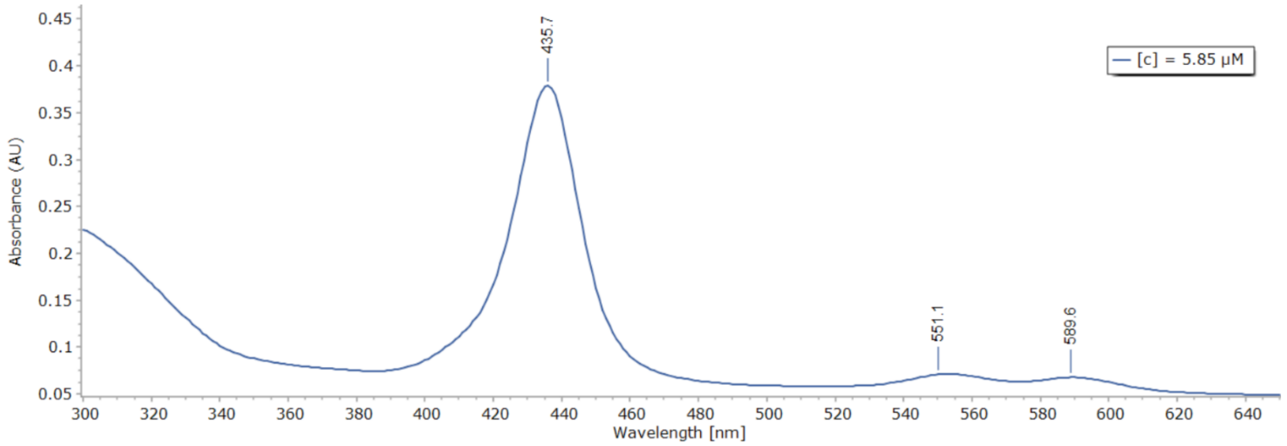

Figure S92: UV–vis spectrum of porphyrin **41** in CH_2Cl_2.

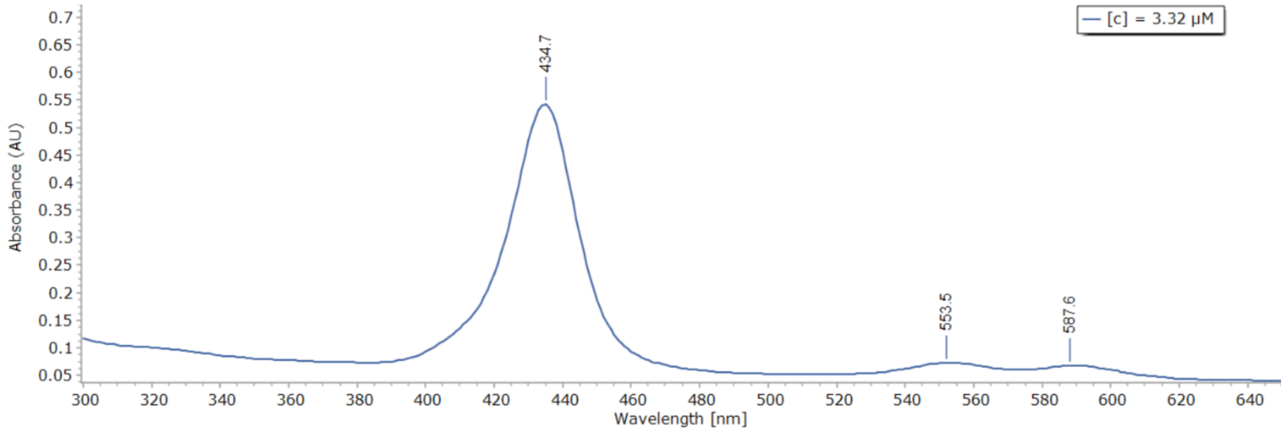

Figure S93: UV–vis spectrum of porphyrin **42** in CH_2Cl_2.

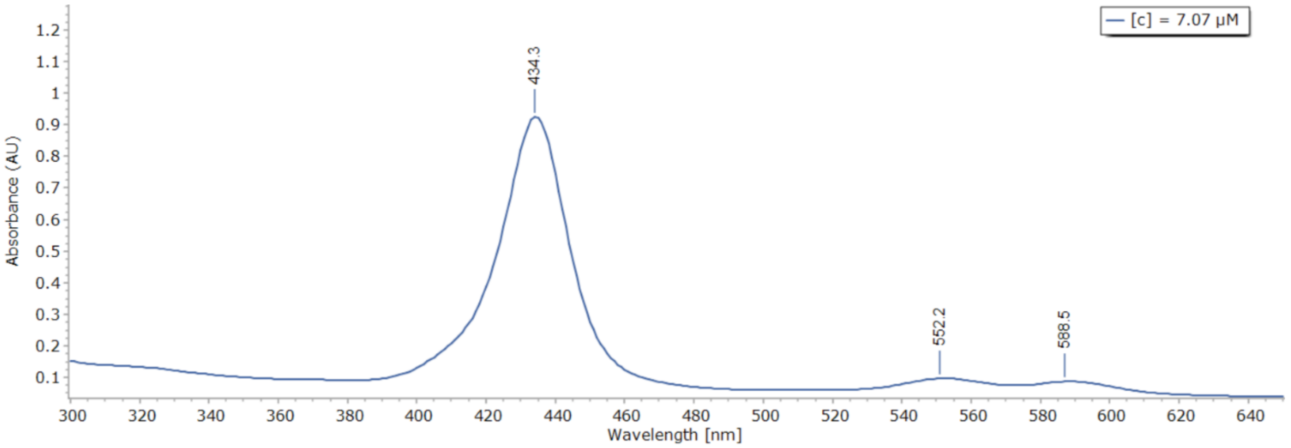

Figure S94: UV–vis spectrum of porphyrin **43** in CH_2Cl_2.

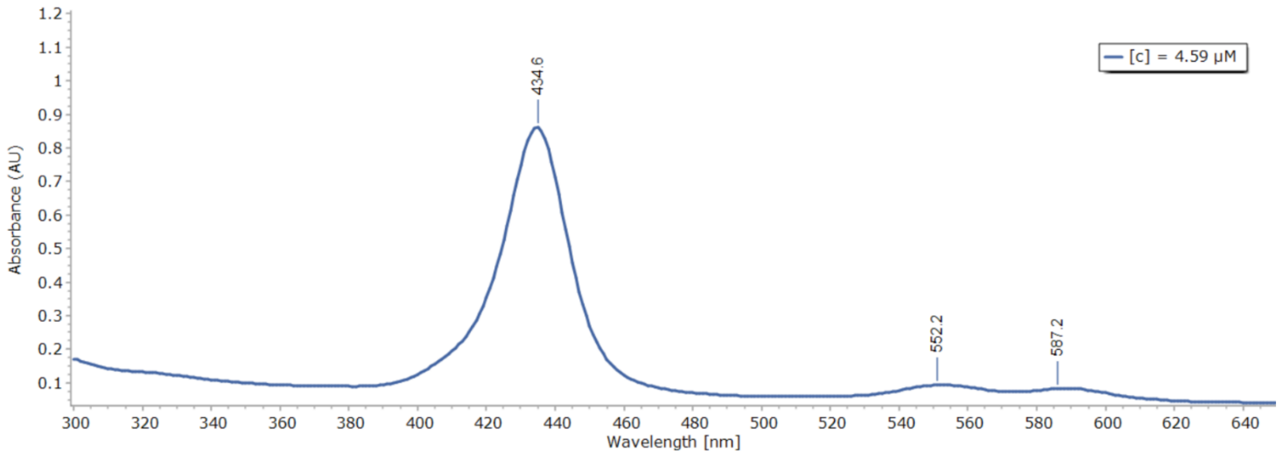

Figure S95: UV–vis spectrum of porphyrin **44** in CH_2Cl_2**.**

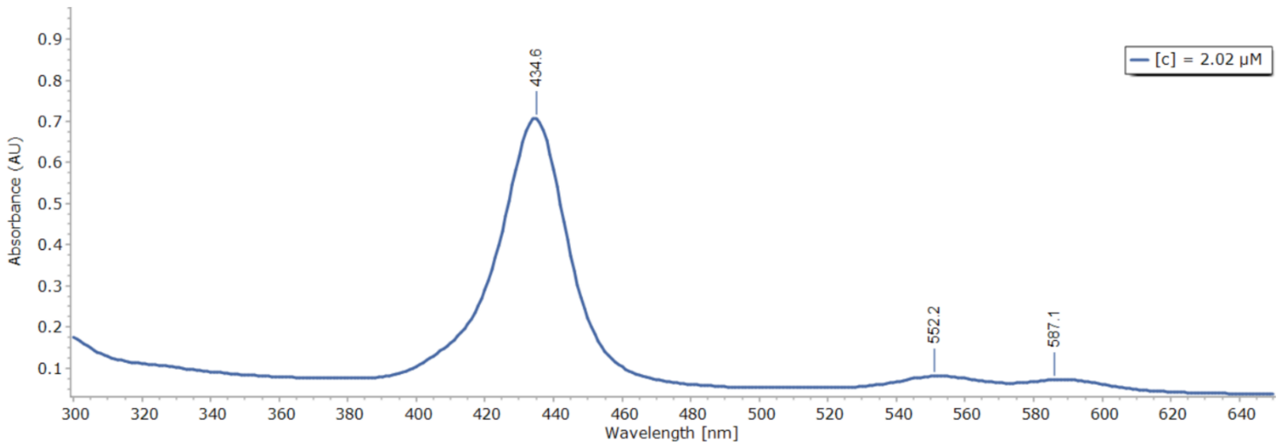

Figure S96: UV–vis spectrum of porphyrin **45** in CH_2Cl_2**.**

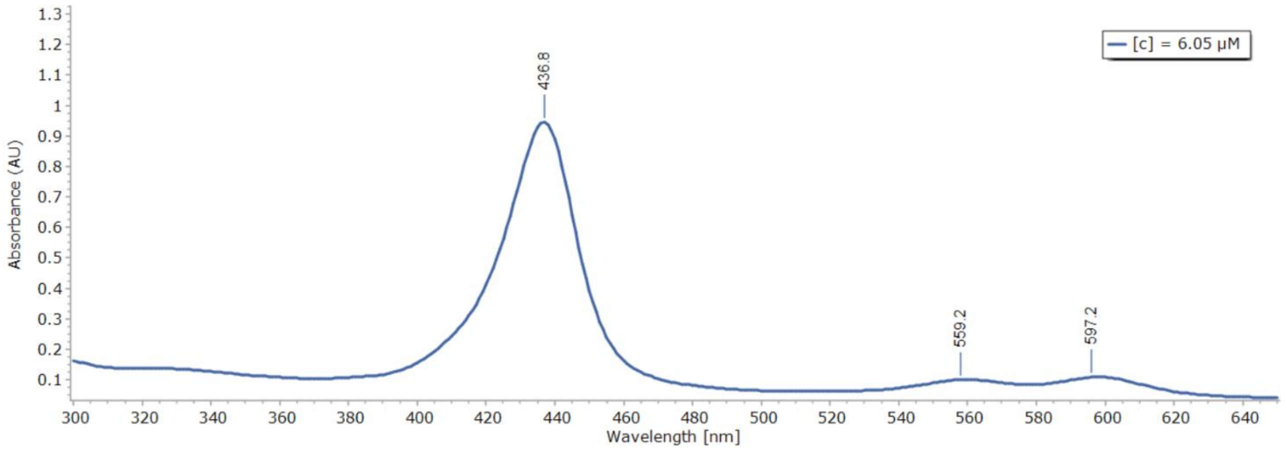

Figure S97: UV–vis spectrum of porphyrin **11** in CH_2Cl_2**.**

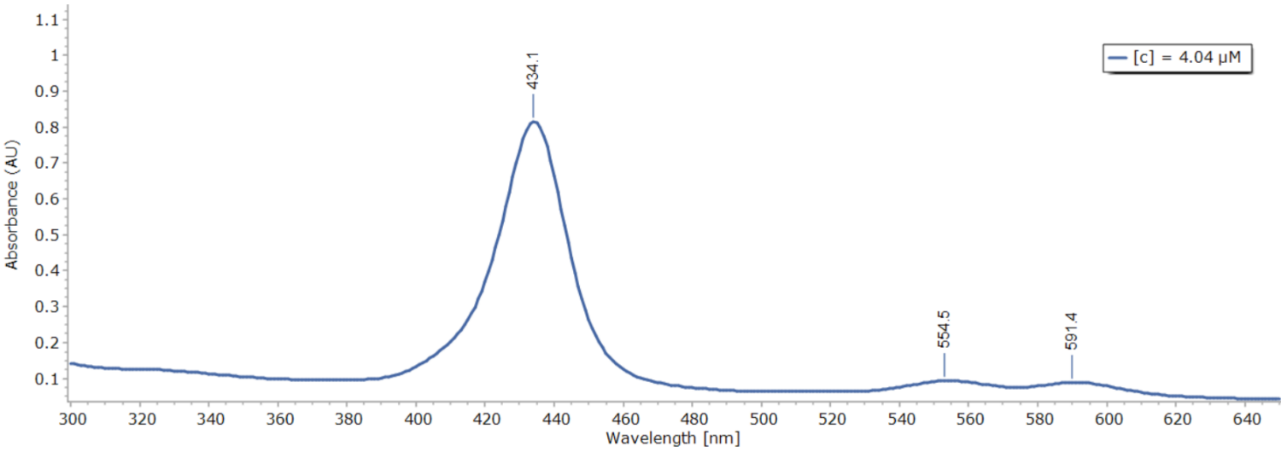

Figure S98: UV–vis spectrum of porphyrin **12** in CH_2Cl_2**.**

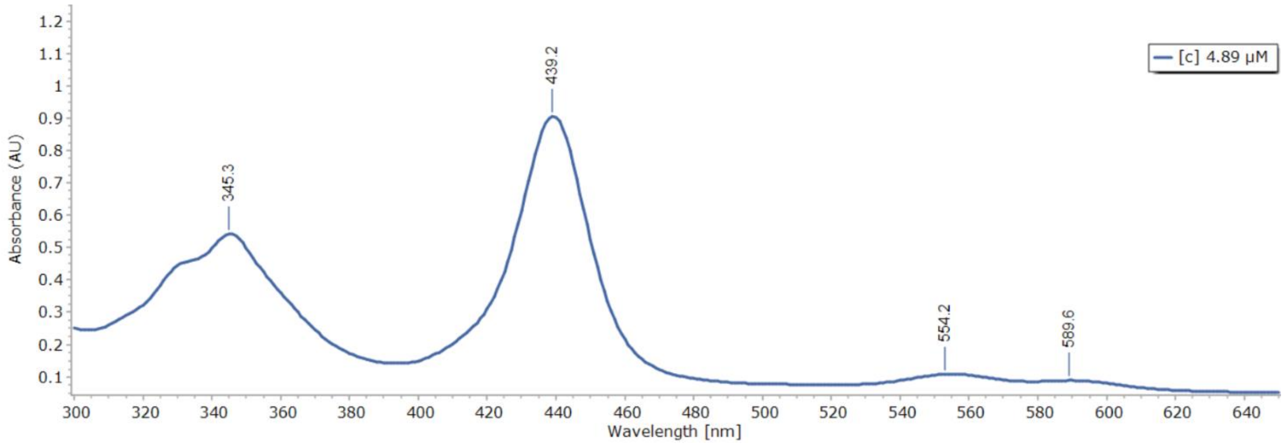

Figure S99: UV–vis spectrum of porphyrin **47** in CH_2Cl_2**.**

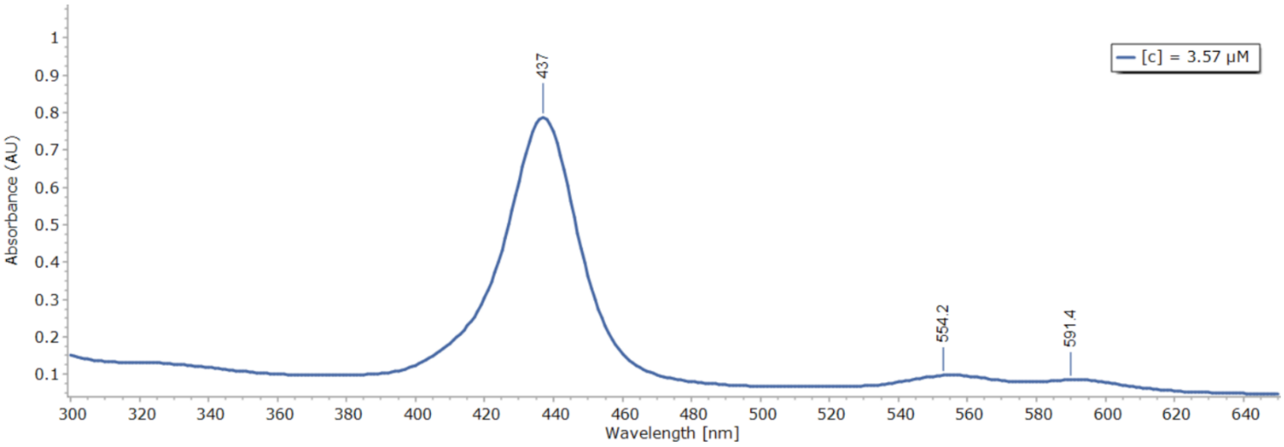

Figure S100: UV–vis spectrum of porphyrin **46** in CH_2Cl_2**.**

IR spectra:

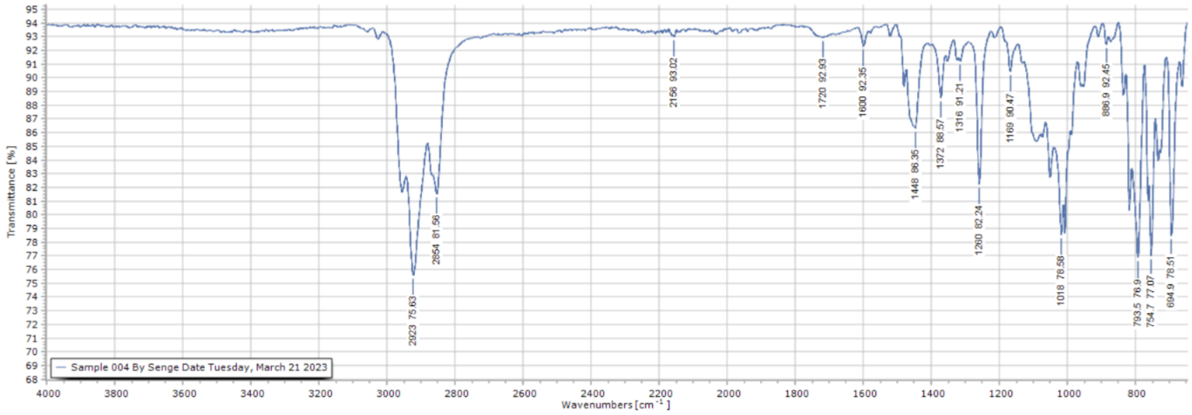

Figure S101: FTIR (ATR) spectrum of porphyrin **26.**

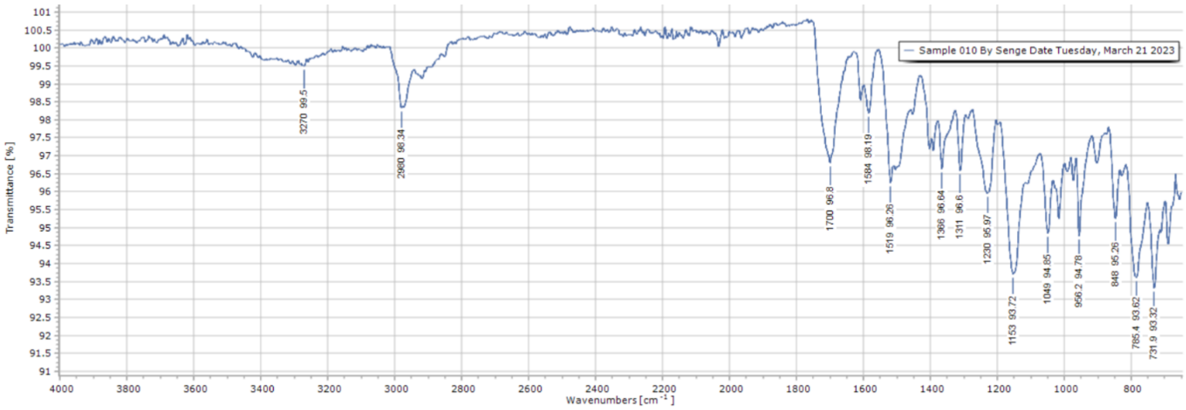

Figure S102: FTIR (ATR) spectrum of porphyrin **27.**

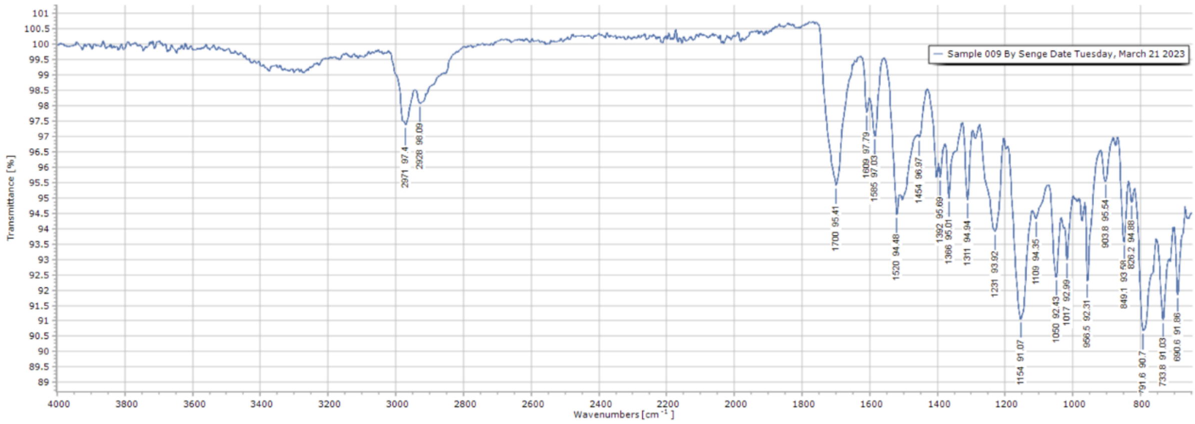

Figure S103: FTIR (ATR) spectrum of porphyrin **28.**

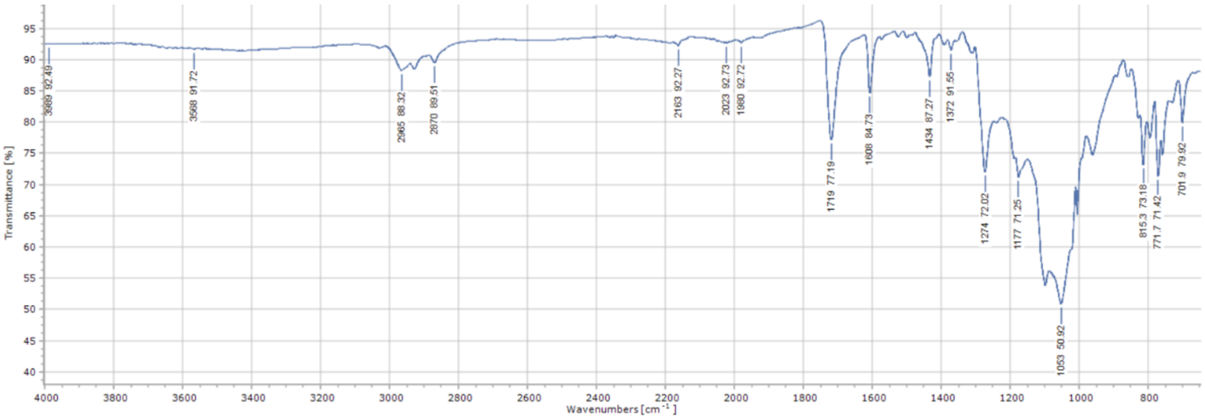

Figure S104: FTIR (ATR) spectrum of porphyrin **29.**

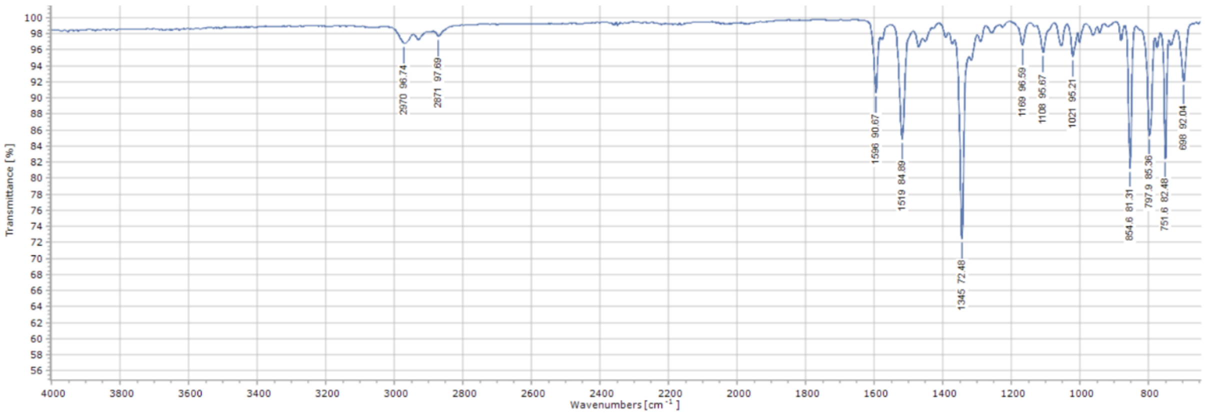

Figure S105: FTIR (ATR) spectrum of porphyrin **40.**

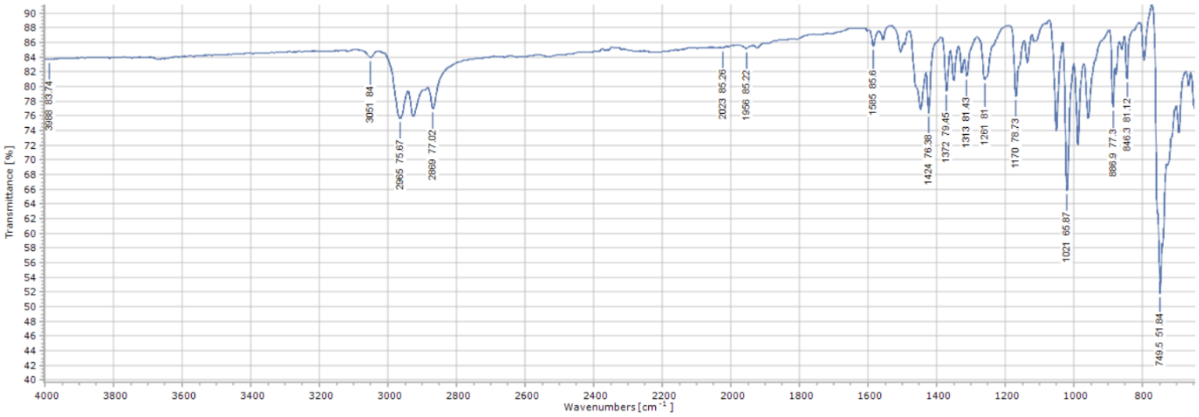

Figure S106: FTIR (ATR) spectrum of porphyrin **11.**

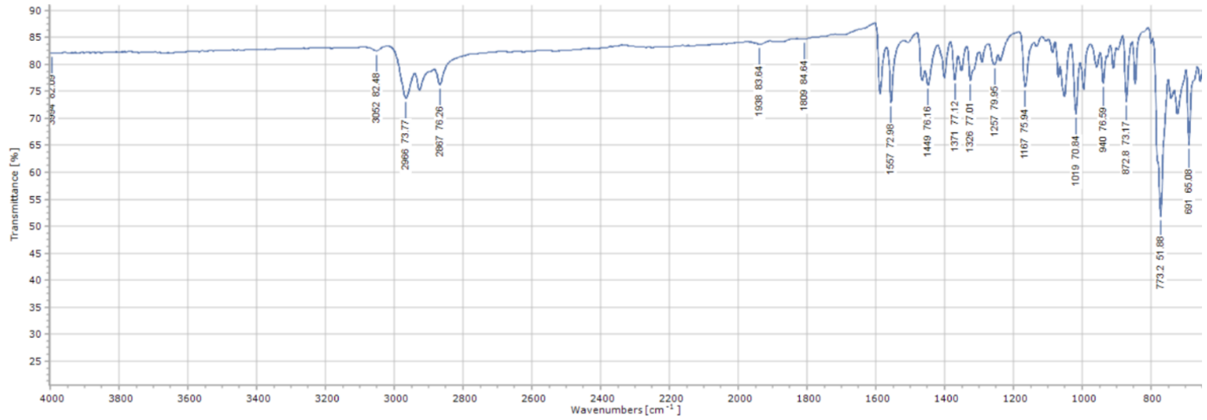

Figure S107: FTIR (ATR) spectrum of porphyrin **12.**

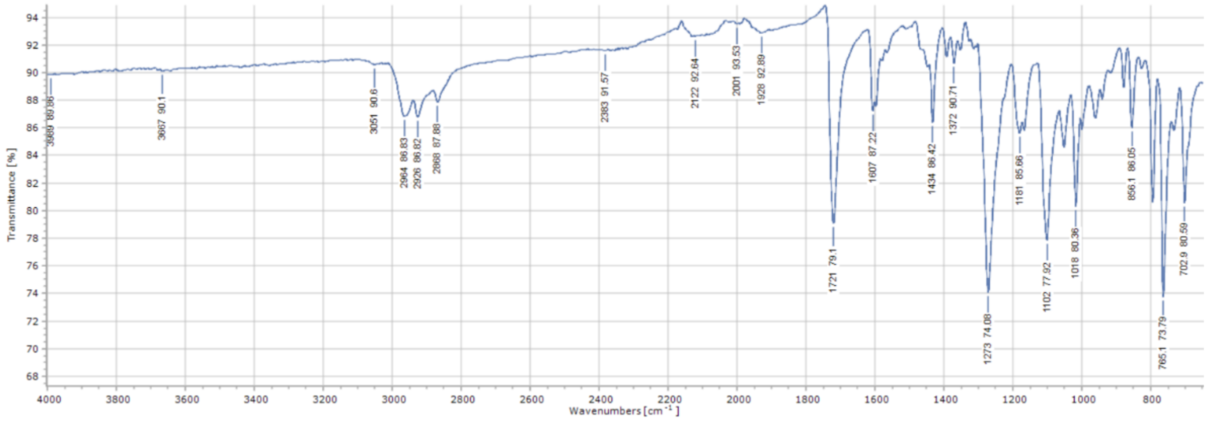

Figure S108: FTIR (ATR) spectrum of porphyrin **39.**

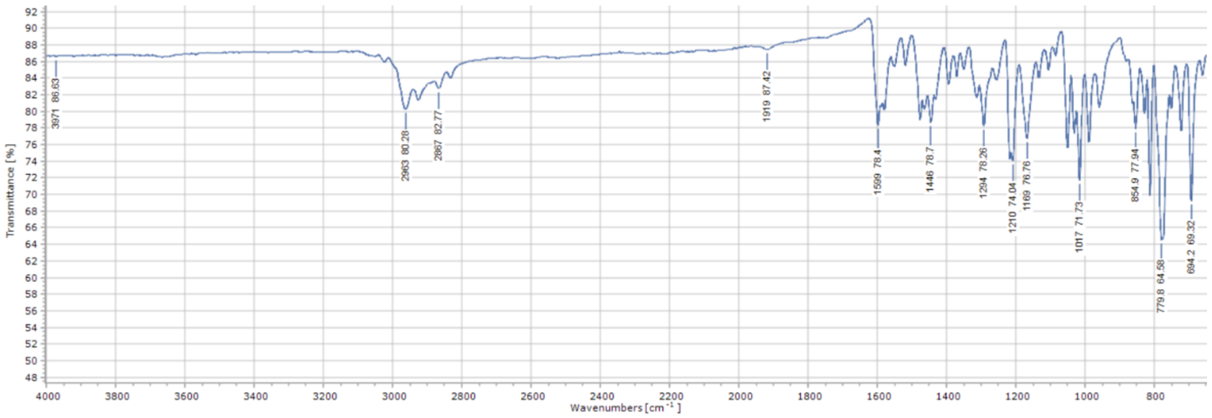

Figure S109: FTIR (ATR) spectrum of porphyrin **35.**

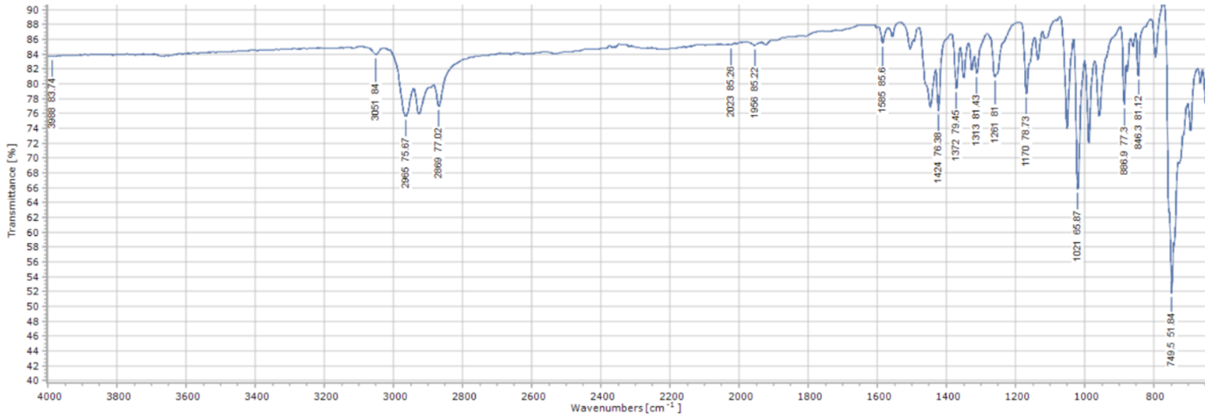

Figure S110: FTIR (ATR) spectrum of porphyrin **32.**

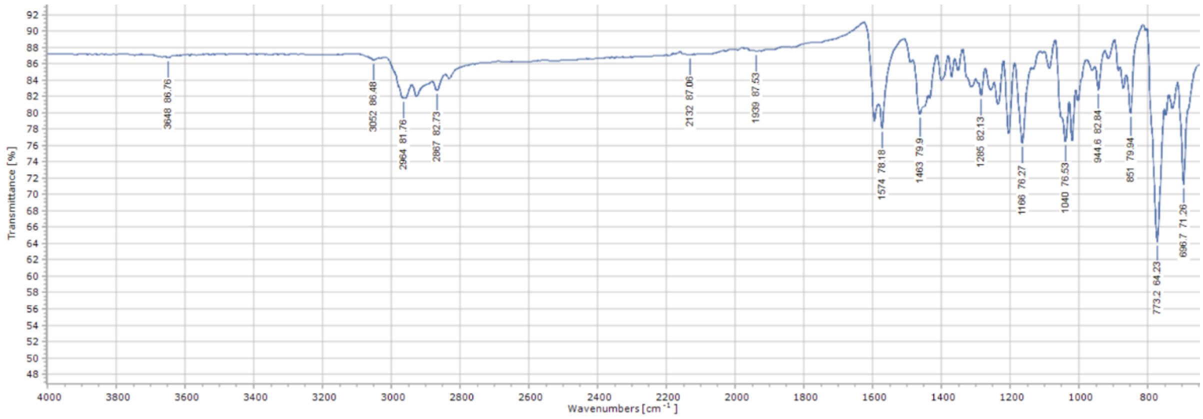

Figure S111: FTIR (ATR) spectrum of porphyrin **45.**

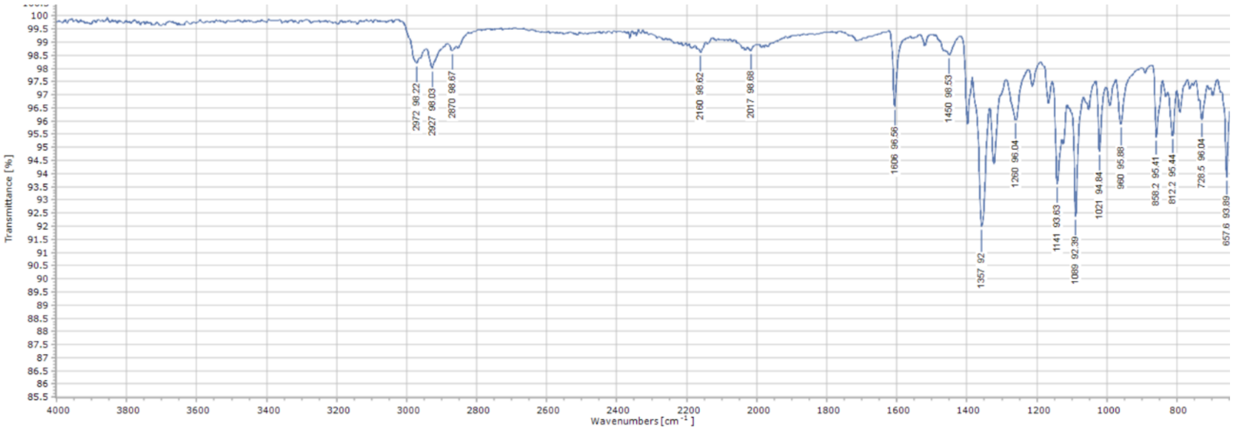

Figure S112: FTIR (ATR) spectrum of porphyrin **46.**

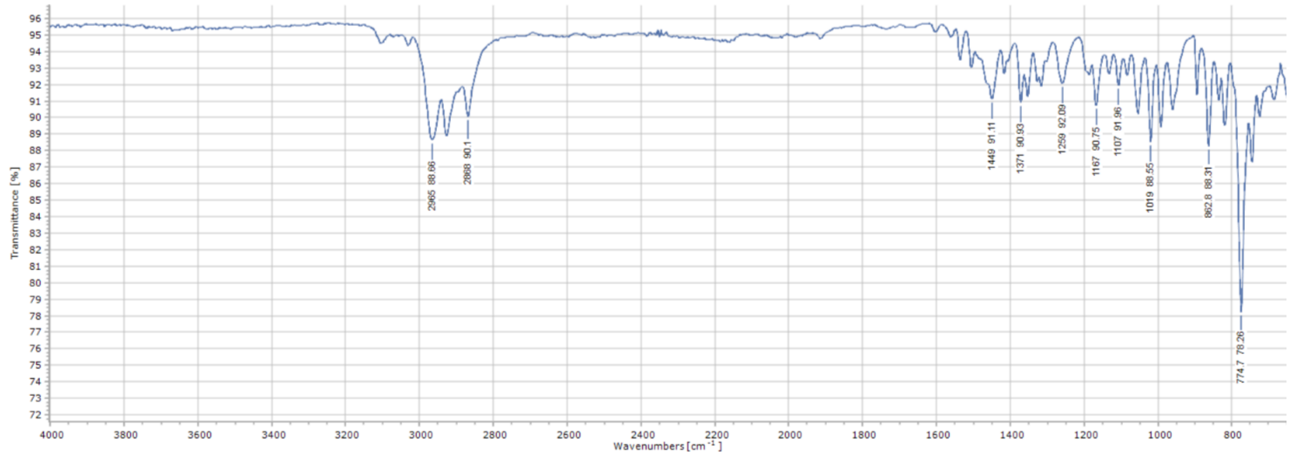

Figure S113: FTIR (ATR) spectrum of porphyrin **41.**

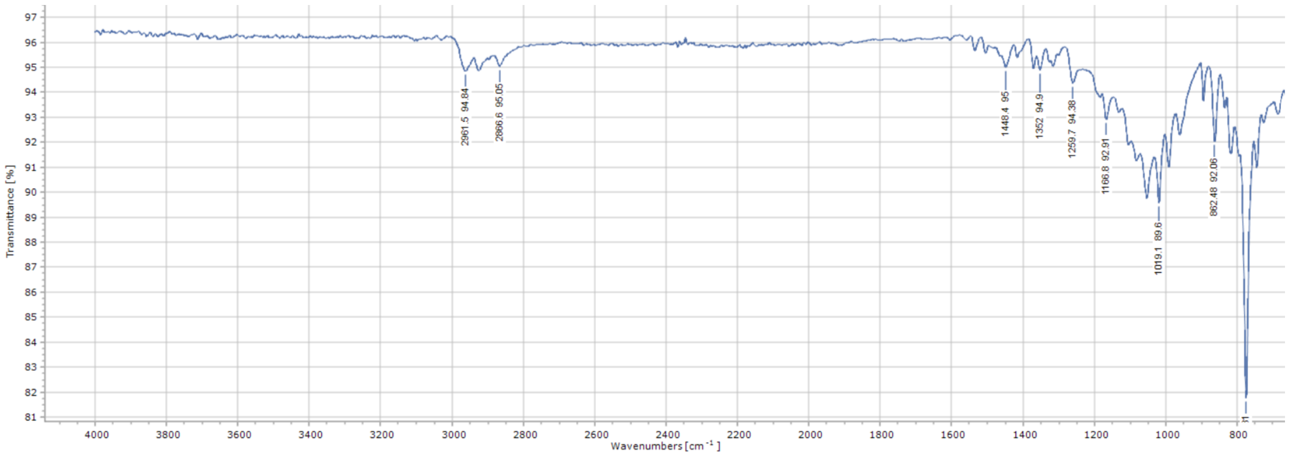

Figure S114: FTIR (ATR) spectrum of porphyrin **33.**

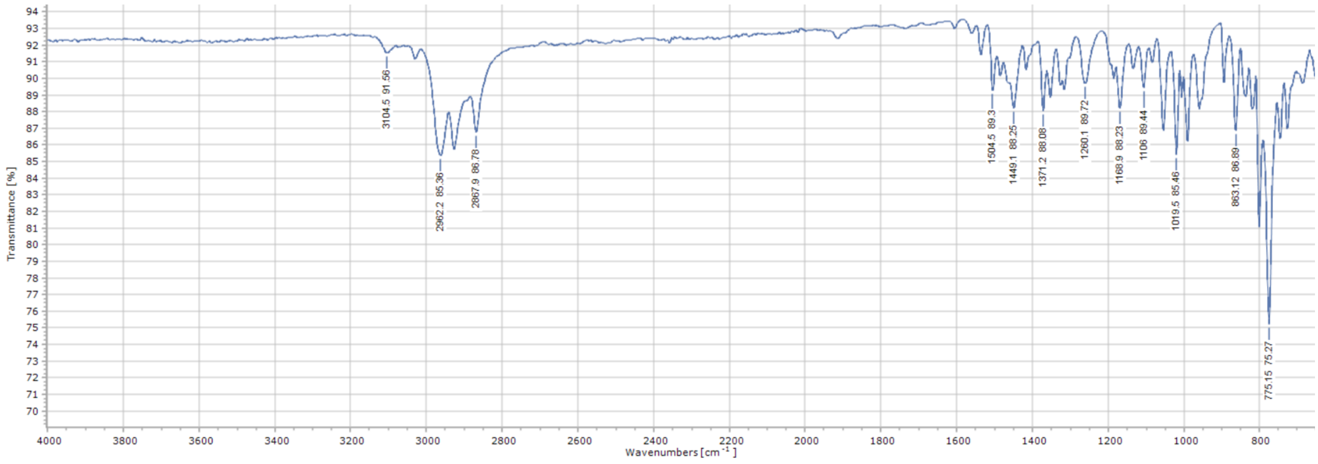

Figure S115: FTIR (ATR) spectrum of porphyrin **34.**

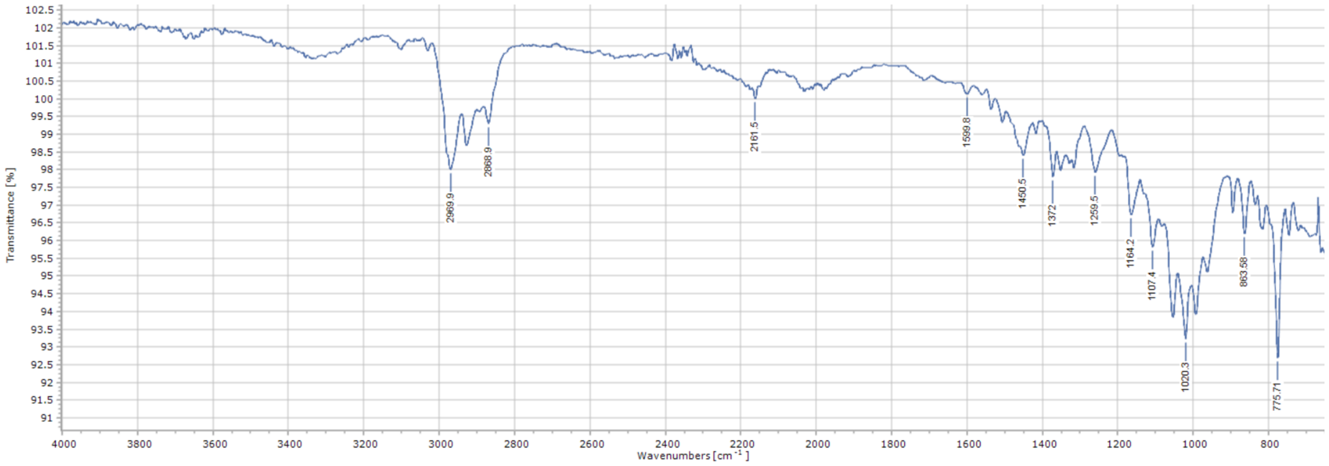

Figure S116: FTIR (ATR) spectrum of porphyrin **30.**

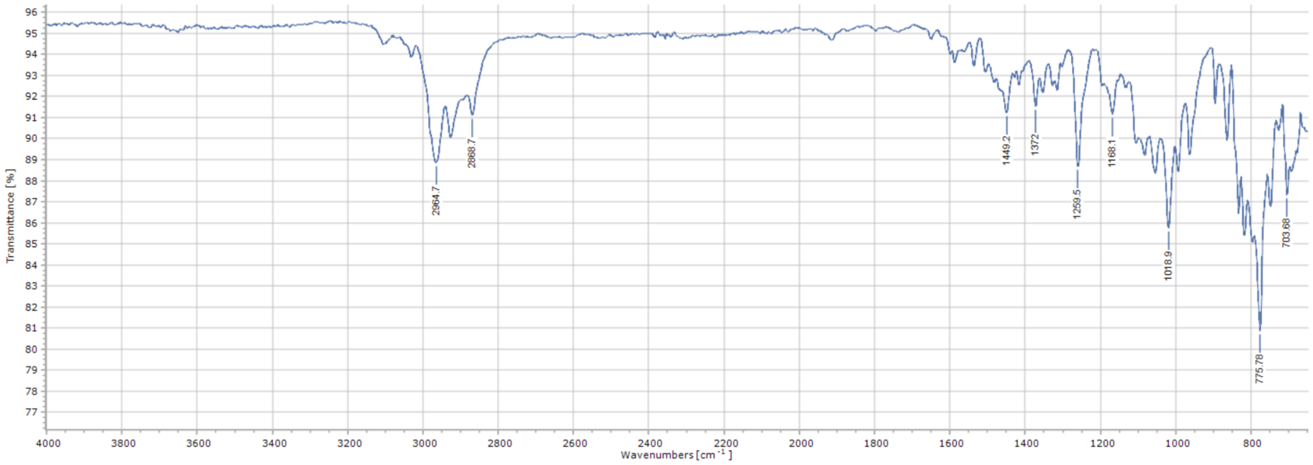

Figure S117: FTIR (ATR) spectrum of porphyrin **47.**

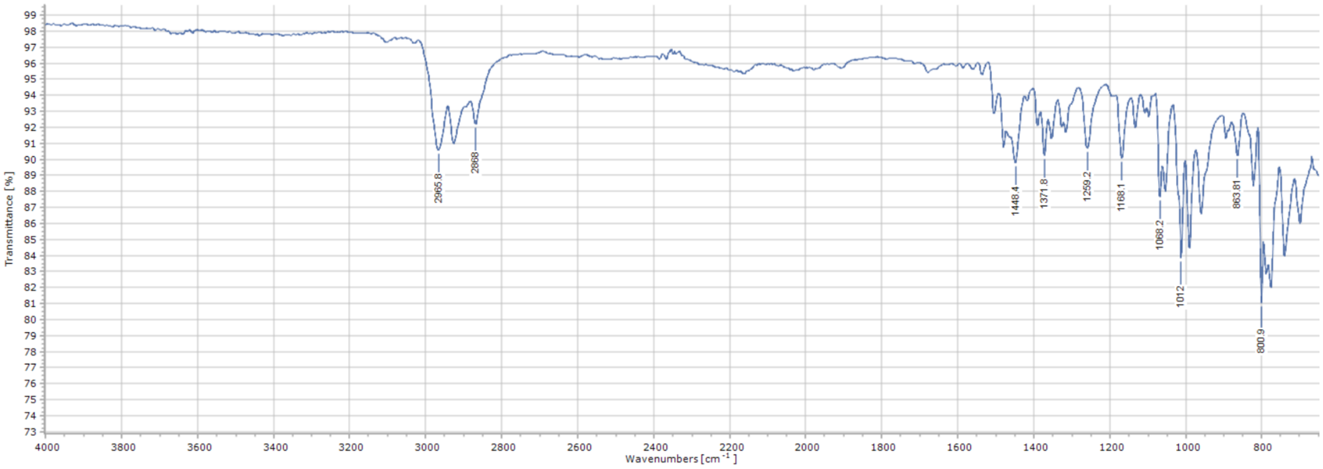

Figure S118: FTIR (ATR) spectrum of porphyrin **32.**

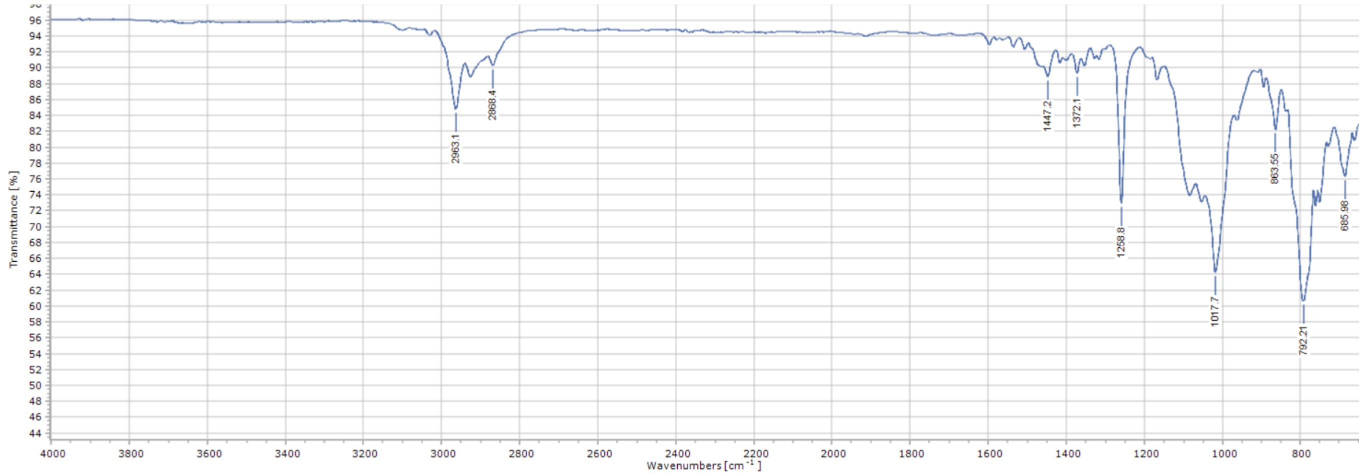

Figure S119: FTIR (ATR) spectrum of porphyrin **38.**

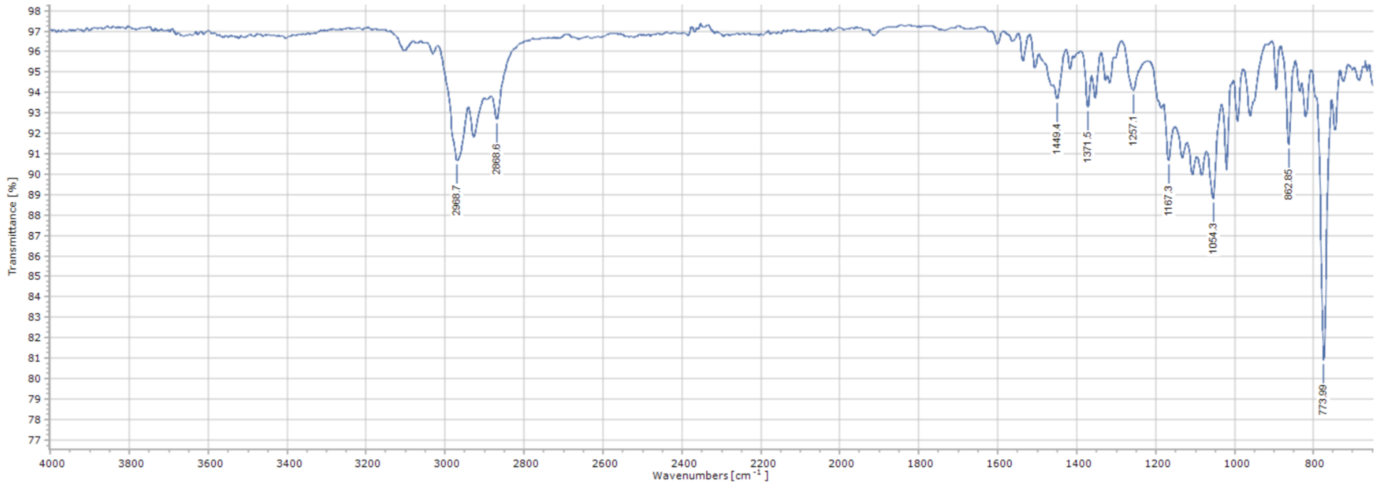

Figure S120: FTIR (ATR) spectrum of porphyrin **43.**

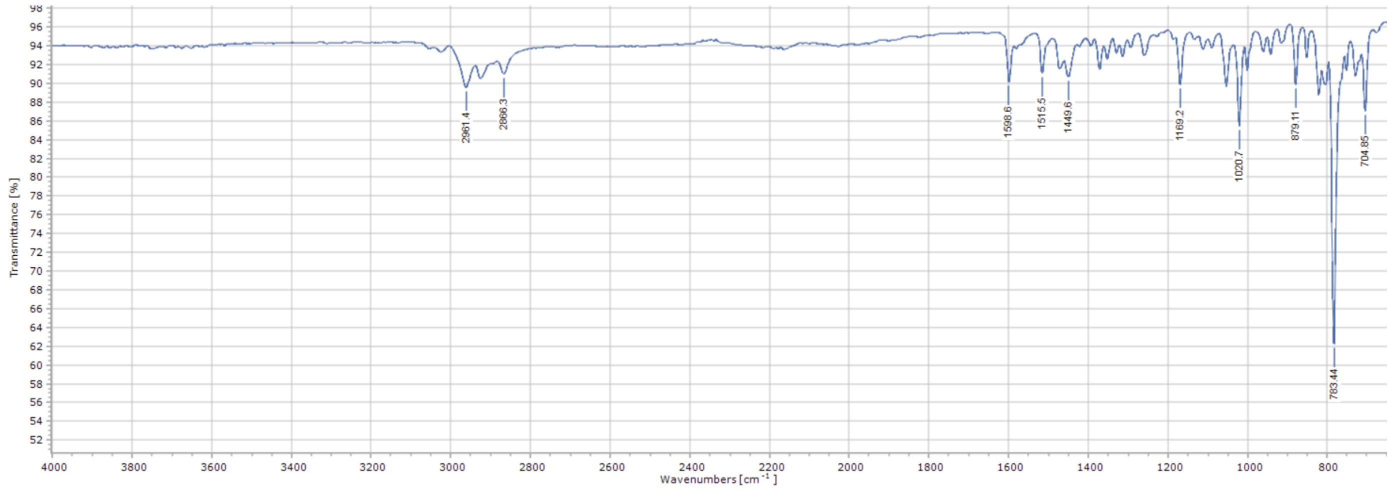

Figure S121: FTIR (ATR) spectrum of porphyrin **44.**

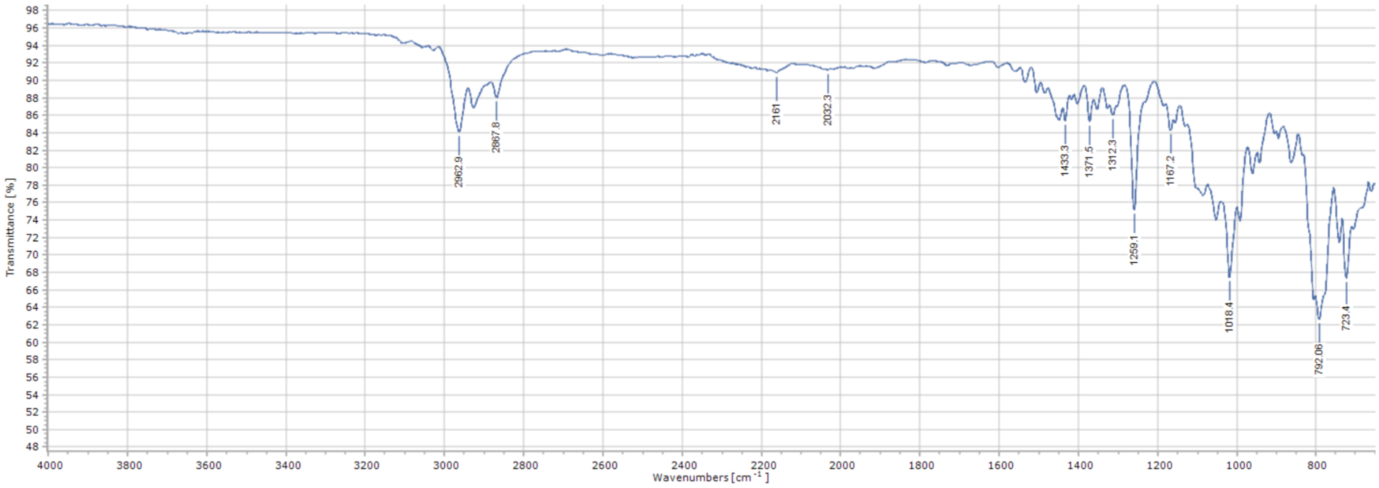

Figure S122: FTIR (ATR) spectrum of porphyrin **31.**

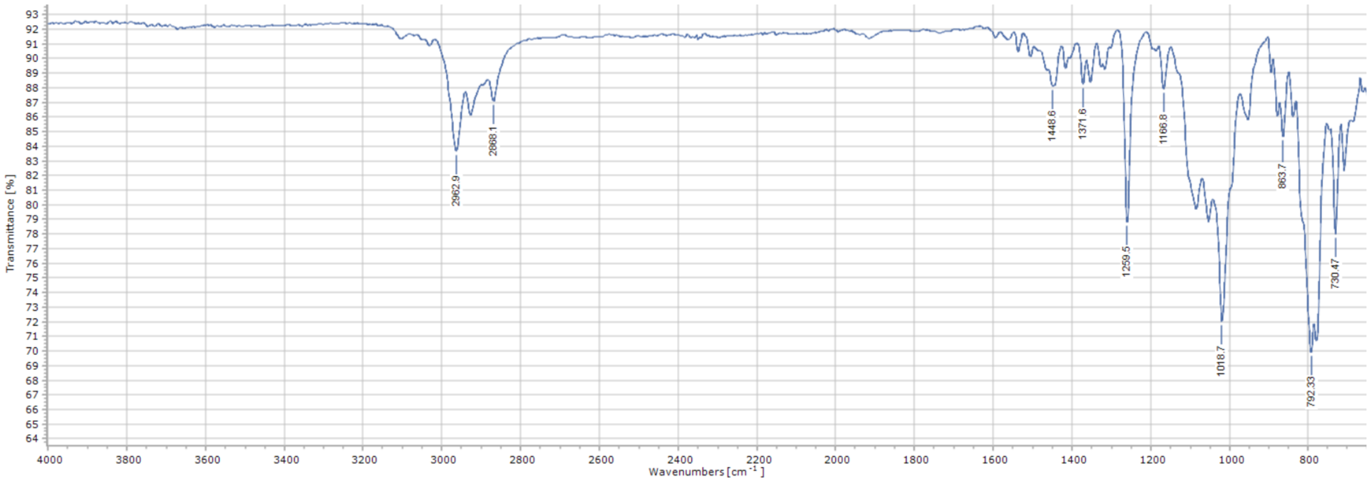

Figure S123: FTIR (ATR) spectrum of porphyrin **37.**

Single crystal X-ray crystallographic data and NSD analysis:

Crystals of porphyrins **11, 26, 27, 28, 29, 33, 36, 37**, and **46** were mounted on a MiTeGen micromount with NVH immersion oil. Data were collected from a shock-cooled single crystal at 100(2) K on a Bruker APEX2 Kappa Duo Kappa diffractometer with a microfocus sealed X-ray tube using mirror optics as a monochromator and an APEX2 detector. The diffractometer was equipped with an Oxford Cobra low temperature device and used Cu Kα radiation (λ = 1.54178 Å). All data were integrated with SAINT and a multi-scan absorption correction using SADABS was applied [1,2]. Structures were solved by dual methods using SHELXT and refined by full-matrix least-squares methods against F^2 by SHELXL using Olex2 [3,4]. All non-hydrogen atoms were refined with anisotropic displacement parameters. All C-bound hydrogen atoms were refined isotropic on calculated positions using a riding model with their U_{iso} values constrained to 1.5 times the U_{eq} of their pivot atoms for terminal sp^3 carbon atoms and 1.2 times for all other carbon atoms. Disordered moieties were refined using bond lengths restraints and displacement parameter restraints. All images were generated using Olex2 [5].

NSD symmetry analysis, in-plane and out-of-plane skeletal plots of the porphyrin core as well as generation of neoplastic representations of porphyrins **11**, **26**, **27**, **28**, **29**, **33**, **36**, **37**, and **46** were achieved by using the online NSD symmetry analysis tool, developed by Senge and co-workers [6].

Refinement details:

Crystal data for compound 11:

In the structure of **11**, each *ortho*-substituted phenyl ring on the porphyrin is disordered. In terms of occupancy of carbon atoms, for C25, two independent rings are occupied in a

70:30% ratio; for C35, two independent rings are in a 51:49% ratio; in C45, only the Br atoms are disordered 60:40%; in the case of C55, three independent rings with three separate occupancies of 36:37:27% are observed. Geometric and displacement restraints were also applied (AFIX, DFIX, SADI, ISOR, SIMU, RIGU), with AFIX being used for some phenyl rings. Additionally, the lattice void is occupied by disordered CH_2Cl_2, with a total occupancy of 50%, modelled over four positions using rigid groups (20:15:6:6%). Multiply occupied sites were also modelled using restraints (SUMP) to sum to either 50 or 100% occupancy.

Crystal data for compound 26:

Crystals of **26** showed weak diffraction with poor high angle data, despite low temperature collection and long exposures. Resolution was limited to d = 0.92 Å. The symmetry generated molecule contains a half occupied MeOH on the mirror plane and $CHCl_3$, 80% occupied in total, and disordered over two unique locations (35 and 5% each), the rest generated by symmetry. These were modelled using a rigid group and displacement restraints (SIMU, ISOR).

Crystal data for compound 27:

Crystals of **27** exhibited very weak diffraction, especially at higher angles using LT (100K) and long exposure times (100 s) with a copper microfocus source. The diffraction limit was set at 0.89, 2θ = 120°. There is also disorder in one of the β-ethyl groups which was modelled in two positions with 61:39% occupancy. Geometric restraints (SADI) and displacement constraints (EADP, C13) were used. The solvents in the lattice could not be modelled satisfactorily and were removed using the solvent mask routine in OLEX2. A total of 86 electrons in 339 Å^3 were removed from the ASU. This is approx. two CH_2Cl_2 molecules or a mixture of CH_2Cl_2/MeOH per half porphyrin.

Crystal data for compound 28:

Crystals of **28** grew as two independent molecules, one of the porphyrin complexes (Ni2) shows disorder in opposing terphenyl groups. One terminal phenyl was modelled in two positions with 60:40% occupancy, with geometrical (SADI) and displacement (SIMU) restraints. In the opposing terphenyl, the last two phenyl rings were modelled in two positions, 50% occupancy, with geometrical (SADI) and displacement (ISOR) restraints. Disordered CH_2Cl_2 is present in the lattice and was modelled in four locations. One site is fully occupied (C11, C12), the next site was modelled at 60% occupancy over two locations (36:24% occupied, C13, C14, C111, C112), the third site modelled over three locations with a total of 55% occupancy (C15, C16, 25%; C19, C110 20%; C115, C116, 10%) and the fourth site modelled over four locations, total of 40% occupancy, with 10% occupancy for each location. All CH_2Cl_2 molecules were modelled with rigid groups and displacement restraints (SIMU, ISOR). The weight was adjusted manually to reduce the GOOF due to poor quality of the data.

Crystal data for compound 29:

The structure of **29** was refined as a merohedral twin around (0 1 0) using the twin law (0 −1 0, −1 0 0, 0 0 −1) with a refined BASF 0.2158(16). One $CDCl_3$ molecule was found to be disordered and modelled over two locations (73:27% occupancy) with restraints (DFIX, SADI, RIGU, SIMU) and constraints (EADP, C11A, C11B) being implemented. A partially occupied CH_2Cl_2 was also modelled over two locations (18:2%, total 20% occupancy) using restraints (DFIX, DANG, SIMU, RIGU) and constraints (EADP, C48a/C48b and C14a/C14b/C15a/C15b).

Crystal data for compound 33:

Two thiophenyl moieties on the porphyrin **33** are disordered and were modelled over two locations each, S2 88:12% and S4 68:32% occupied, using restraints (SIMU, RIGU, SADI,

FLAT, ISOR) and constraints (EADP, EXYZ). Two ethyl groups were also found to be disordered and modelled over two locations C53/C54 and C68/C69 62:38% and 76:24% occupied, using restraints (SADI, RIGU, ISOR) and a constraint (EADP). There are two hexane solvent molecule locations, one above and one below the porphyrin, both are disordered and were modelled in two locations with C85-C90 50:50% occupied and C91-C96 80:20% occupied. Restraints (SADI, RIGU, ISOR, SIMU) and some constraints (EADP) were also used.

Crystal data for compound 36:

In the porphyrin molecule **36**, one meso-substituted biphenyl group is disordered over two positions (68:32%) and modelled using geometric (SADI) and displacement (SIMU, ISOR) restraints. The minor ring component was also modelled constrained to be a hexagon (AFIX). There are several solvent sites in the voids. One site is occupied by CH_2Cl_2 in two locations and MeOH (75:5:25% occupied, respectively). These are refined using geometric (DFIX) and displacement restraints (SIMU, ISOR). The next disordered solvent site involves the minor disordered component of the biphenyl group; there is hexane and CH_2Cl_2 (15:15% occupied) present, also modelled with displacement (SIMU, ISOR) restraints and constraints (EADP). The third site involves the major occupied biphenyl moiety with MeOH and CH_2Cl_2 (25:25% occupied) present and modelled using displacement (SIMU, ISOR) and geometric (RIGU) restraints. All hexane/CH_2Cl_2 solvents were modelled using rigid groups. The highest occupied moieties were placed in PART1 of the model. The moiety formula is $C_{84}H_{76}N_4Ni$, 0.9(CH_4O), 2.2(CH_2Cl_2), 0.3(C_6H_{14}).

Crystal data for compound 37:

Weakly diffracting crystal with little high angle data. Resolution limit d = 0.89 Å. Disorder in three peripheral anthracenyl and three phenyl groups. All modelled in two locations with C25, 64:36%. C49, 55::45%. C73, 61,39%. C10A/B 50:50%. All were refined using

restraints (RIGU, ISOR, SIMU, SADI, FLAT, DANG) and some constraints (EADP). Large voids in the ASU with diffuse electron density (550 cubic Å//140 electrons) present. This was removed from the model using the BYPASS routine in OLEX2.

Crystal data for compound 46:

Pinacol borate moiety on the porphyrin disordered and modelled over two locations (61:39% occupied) with restraints (SADI, RIGU, SIMU) and constraints (EADP, O1, O2). Half a bis(pinacolato)diboron is located in the void and was modelled in two positions in the ASU (13:12% occupancy) using restraints (RIGU, SIMU, DFIX, ISOR) and constraints (EADP).

Crystallographic data for the structures reported here have been deposited with the Cambridge Crystallographic Data Centre [7]. CCDC 2374316–2374324 contain the supplementary crystallographic data for this paper. These data can be obtained free of charge from The Cambridge Crystallographic Data Centre via www.ccdc.cam.ac.uk/ structures. This report and the CIF file were generated using FinalCif [8].

X-ray crystallographic data refinement tables:

Table S2: Details of XRD refinement.

Compound	26	27	28
**Local TCD number**	TCD2017	TCD2036	TCD2038
**CCDC number**	2374320	2374319	2374321
**Empirical formula**	$C_{85.30}H_{78.80}Cl_{2.40}N_4NiO_{0.50}$	$C_{116}H_{92}N_4Ni$	$C_{109.28}H_{94.55}Cl_{2.55}N_4Ni$
**Formula weight**	1311.71	1600.64	1612.84
**Temperature [K]**	100(2)	100(2)	100(2)
**Crystal system**	orthorhombic	monoclinic	monoclinic
**Space group (number)**	$Pnma$ (62)	$C2/c$ (15)	$P2_1/n$ (14)
a **[Å]**	15.5133(8)	33.9851(15)	19.8258(10)
b **[Å]**	33.8111(16)	16.2790(8)	28.6176(14)
c **[Å]**	12.9605(5)	20.3890(9)	30.9925(16)
**α [°]**	90	90	90
**β [°]**	90	115.052(3)	90.157(3)
**γ [°]**	90	90	90
**Volume [$Å^3$]**	6798.1(5)	10218.9(8)	17584.0(15)
Z	4	4	8
**ρ_{calc} [gcm^{-3}]**	1.282	1.040	1.218
**μ [mm^{-1}]**	1.665	0.624	1.422
***F*(000)**	2766	3376	6796
**Crystal size [mm^3]**	0.248×0.093×0.012	0.296×0.102×0.042	0.292×0.179×0.15
**Crystal colour**	orange	red	dark red
**Crystal shape**	plate	block	block
**Radiation**	Cu K_α (λ=1.54178 Å)	Cu K_α (λ=1.54178 Å)	Cu K_α (λ=1.54178 Å)
**2Θ range [°]**	5.23 to 113.79 (0.92 Å)	6.14 to 120.26 (0.89 Å)	5.28 to 122.58 (0.88 Å)
**Reflections collected**	94336	56540	196840
**Independent reflections**	4618<br>R_{int} = 0.2442<br>R_{sigma} = 0.0878	7580<br>R_{int} = 0.0867<br>R_{sigma} = 0.0598	26795<br>R_{int} = 0.1074<br>R_{sigma} = 0.0677
**Completeness**	99.3 %	99.4 %	98.9 %
**Data / Restraints / Parameters**	4618/42/484	7580/3/564	26795/711/2407
**Goodness-of-fit on F^2**	1.047	1.026	1.103
**Final *R* indexes [I≥2σ(I)]**	R_1 = 0.0744<br>wR_2 = 0.1723	R_1 = 0.0777<br>wR_2 = 0.2160	R_1 = 0.1216<br>wR_2 = 0.4022
**Final *R* indexes [all data]**	R_1 = 0.1321<br>wR_2 = 0.2031	R_1 = 0.1100<br>wR_2 = 0.2531	R_1 = 0.1850<br>wR_2 = 0.4797
**Largest peak/hole [$eÅ^{-3}$]**	0.37/-0.34	0.84/-0.41	0.80/-0.58
**Flack X parameter**	--	--	--
**Extinction coefficient**	0.00021(6)	--	--

Table S3: Details of XRD refinement.

Compound	**36**	**11**	**29**
**Local TCD Number**	TCD2056	TCD2100	TCD2127
**CCDC number**	2374323	2374316	2374317
**Empirical formula**	$C_{88.90}H_{88.20}Cl_{4.40}N_4NiO_{0.90}$	$C_{60.50}H_{57}Br_4ClN_4Ni$	$C_{96.40}H_{84.80}D_4Cl_{12.80}N_4NiO_8$
**Formula weight**	1441.72	1253.90	1947.80
**Temperature [K]**	100(2)	100(2)	100(2)
**Crystal system**	monoclinic	triclinic	tetragonal
**Space group (number)**	$C2/c$ (15)	$P\bar{1}$ (2)	$I\bar{4}$ (82)
a **[Å]**	13.7023(4)	13.7188(5)	25.4518(8)
b **[Å]**	27.8497(8)	13.8769(5)	25.4518(8)
c **[Å]**	21.0247(6)	16.3840(6)	14.2061(5)
**α [°]**	90	104.498(2)	90
**β [°]**	96.6680(16)	97.883(2)	90
**γ [°]**	90	107.767(2)	90
**Volume [Å^3]**	7968.9(4)	2797.54(18)	9202.6(7)
Z	4	2	4
**ρ_{calc} [gcm^{-3}]**	1.202	1.489	1.406
**μ [mm^{-1}]**	2.070	4.602	4.194
***F*(000)**	3038	1266	4019
**Crystal size [mm^3]**	0.5×0.089×0.029	0.195×0.077×0.021	0.239×0.232×0.058
**Crystal colour**	clear red	red	red
**Crystal shape**	blade	parallelepiped	fragment
**Radiation**	Cu K_α (λ=1.54178 Å)	Cu K_α (λ=1.54178 Å)	Cu K_α (λ=1.54178 Å)
**2Θ range [°]**	6.35 to 140.26 (0.82 Å)	5.73 to 136.88 (0.83 Å)	4.91 to 139.31 (0.82 Å)
**Reflections collected**	72210	42723	64163
**Independent reflections**	7533<br>R_{int} = 0.0822<br>R_{sigma} = 0.0403	10216<br>R_{int} = 0.0647<br>R_{sigma} = 0.0518	8669<br>R_{int} = 0.0526<br>R_{sigma} = 0.0291
**Completeness**	99.9 %	99.6 %	100.0 %
**Data / Restraints / Parameters**	7533/336/615	10216/1896/942	8669/134/608
**Goodness-of-fit on F^2**	1.067	0.954	1.060
**Final *R* indexes [I≥2σ(I)]**	R_1 = 0.0885<br>wR_2 = 0.2631	R_1 = 0.0800<br>wR_2 = 0.2273	R_1 = 0.0474<br>wR_2 = 0.1257
**Final *R* indexes [all data]**	R_1 = 0.1046<br>wR_2 = 0.2781	R_1 = 0.1135<br>wR_2 = 0.2611	R_1 = 0.0478<br>wR_2 = 0.1263
**Largest peak/hole [eÅ^{-3}]**	0.95/-0.60	1.04/-0.96	0.66/-0.59
**Flack X parameter**	--	--	-0.010(5)
**Extinction coefficient**	--	--	--

Table S4: Details of XRD refinement.

**Compound**	**46**	**37**	**33**
**Local TCD number**	TCD2153	TCD2277	TCD2288
**CCDC number**	2374318	2374322	2374324
**Empirical formula**	C90H116B5N4NiO10	C116H92N4Ni	C88H96N4NiS4
**Formula weight**	1526.62	1600.64	1396.63
**Temperature [K]**	100(2)	100(2)	100(2)
**Crystal system**	tetragonal	triclinic	monoclinic
**Space group (number)**	$P4_2/n$ (86)	$P\bar{1}$ (2)	Ia (9)
**a [Å]**	18.4918(5)	16.4989(7)	23.1134(19)
**b [Å]**	18.4918(5)	17.3045(8)	12.6098(10)
**c [Å]**	13.3536(6)	18.3343(8)	26.744(3)
**α [°]**	90	100.065(2)	90
**β [°]**	90	102.770(2)	101.779(4)
**γ [°]**	90	95.298(2)	90
**Volume [Å3]**	4566.2(3)	4979.5(4)	7630.7(12)
**Z**	2	2	4
**ρcalc [gcm−3]**	1.11	1.068	1.216
**μ [mm−1]**	0.739	0.64	1.746
**F(000)**	1634	1688	2976
**Crystal size [mm3]**	0.146×0.091×0.032	0.125×0.09×0.061	0.216×0.063×0.043
**Crystal colour**	red	red	red
**Crystal shape**	prism	block	plate
**Radiation**	Cu Kα (λ=1.54178 Å)	Cu Kα (λ=1.54178 Å)	Cu Kα (λ=1.54178 Å)
**2Θ range [°]**	8.17 to 133.31 (0.84 Å)	5.04 to 120.32 (0.89 Å)	7.81 to 133.46 (0.84 Å)
**Reflections collected**	38904	63667	40565
**Independent reflections**	4044	14851	13087
	Rint = 0.1239	Rint = 0.0613	Rint = 0.1305
	Rsigma = 0.0543	Rsigma = 0.0525	Rsigma = 0.1475
**Completeness**	99.90%	99.70%	99.00%
**Data / Restraints / Parameters**	4044/759/359	14851/5143/1269	13087/1019/1018
**Goodness-of-fit on F2**	1.04	1.25	0.984
**Final R indexes**	R1 = 0.0829	R1 = 0.1277	R1 = 0.0892
**[I≥2σ(I)]**	wR2 = 0.2282	wR2 = 0.3580	wR2 = 0.2244
**Final R indexes**	R1 = 0.1215	R1 = 0.1929	R1 = 0.1468
**[all data]**	wR2 = 0.2665	wR2 = 0.4148	wR2 = 0.2691
**Largest peak/hole [eÅ−3]**	0.83/-0.53	0.60/-0.38	0.77/-0.46
**Flack X parameter**	--	--	0.10(5)
**Extinction coefficient**	--	--	--

Molecular structure view and NSD analysis of porphyrin 26:

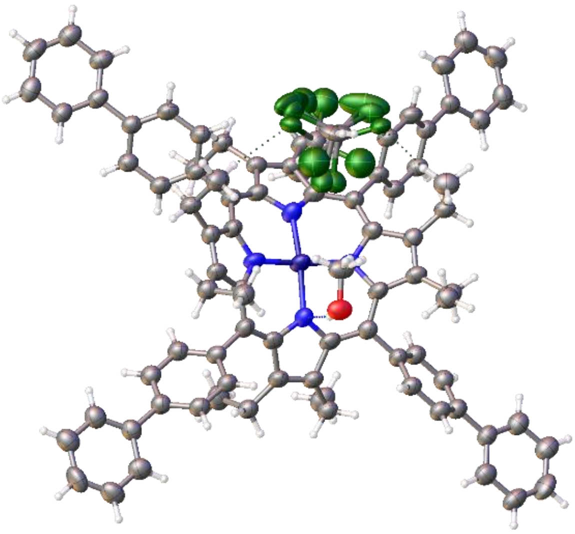

Figure S124: View of the molecular structure of **26** in the crystal, hydrogen atoms omitted for clarity, thermal ellipsoid shows, two solvents incorporated into the structure, MeOH (50%) and $CHCl_3$ (80%). Atomic displacement shown at 50% probability.

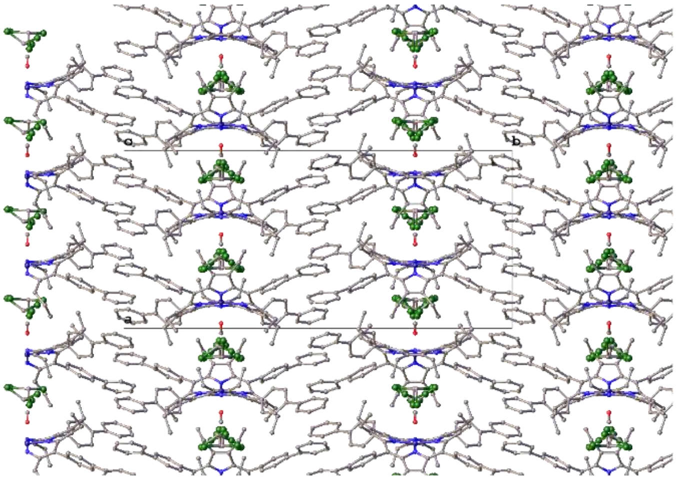

Figure S125: Schematic packing diagram of **26** with hydrogen atoms omitted for clarity.

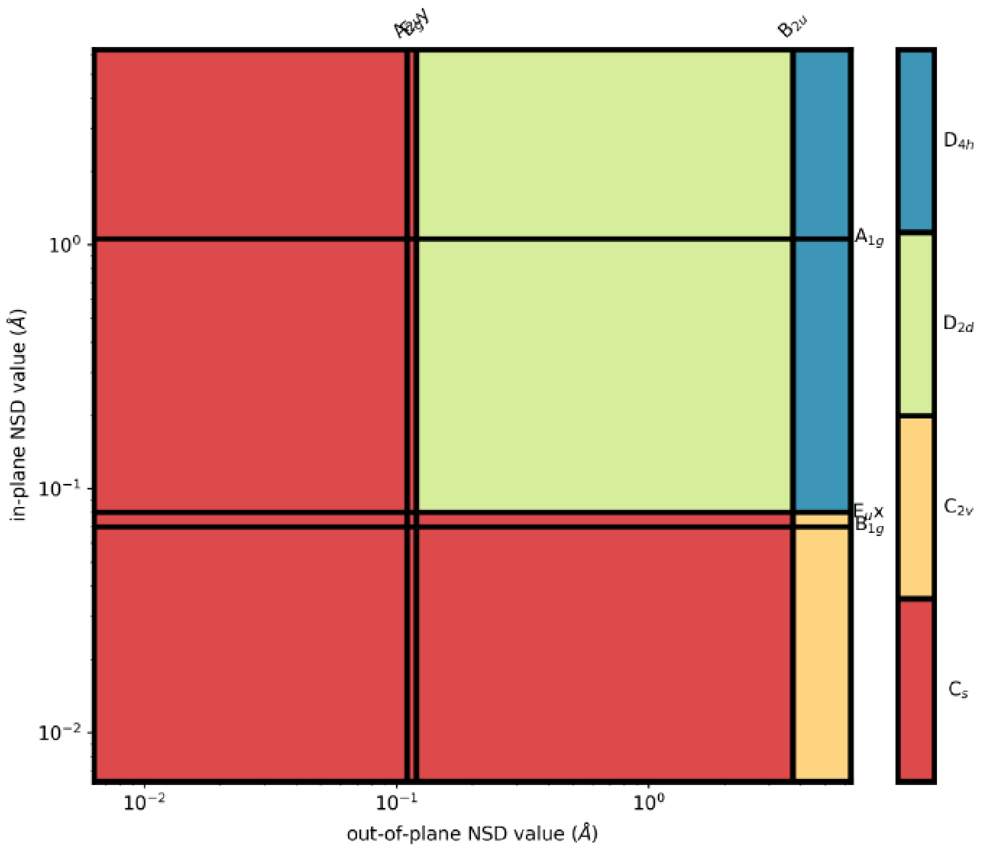

Figure S126: Neoplastic representation of the NSD symmetry elements for porphyrin **26**.

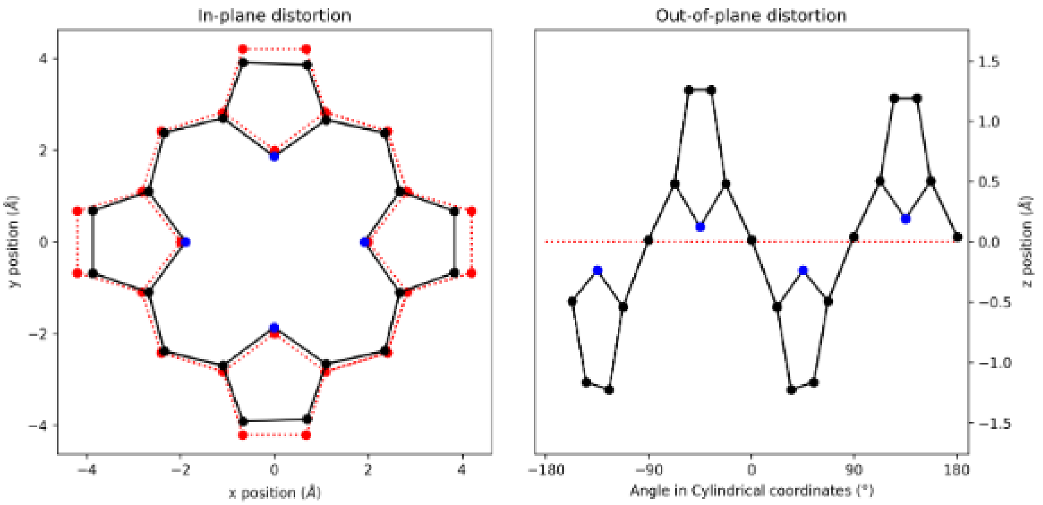

Figure S127: Out-of-plane and in-plane skeletal plots of the porphyrin core. Porphyrin **26** is represented in black (carbon) and blue (nitrogen), with the reference structure CuTPP in red dotted lines.

Table S5: Mean bond distances, angles, and deviations from planarity of porphyrin **26.**

**Bond Distances, Bond Angles, Atom Displacements**	**Mean Value (standard error)**	**Units**
N–C_a	1.39(6)	(Å)
C_a–C_b	1.449(13)	(Å)
C_a–C_m	1.398(6)	(Å)
C_b–C_b	1.359(13)	(Å)
∠$C_aC_bC_b$	106.8(2)	(°)
∠NC_aC_b	110.4(5)	(°)
∠NC_aC_m	120.6(6)	(°)
∠C_aNC_a	104.5(8)	(°)
∠$C_mC_aC_b$	128.5(5)	(°)
∠$C_aC_mC_a$	122.3(9)	(°)
Δ24	0.6098	(Å)
ΔN	0.2(4)	(Å)
ΔC_a	0.5(2)	(Å)
ΔC_b	1.21(4)	(Å)
ΔC_m	0.03(9)	(Å)
∠ pyrrole tilt	28(2)	(°)
N···N dist (adj)	2.695(6)	(Å)
N···N dist (opp)	3.77(4)	(Å)

Molecular structure view and NSD analysis of porphyrin 27:

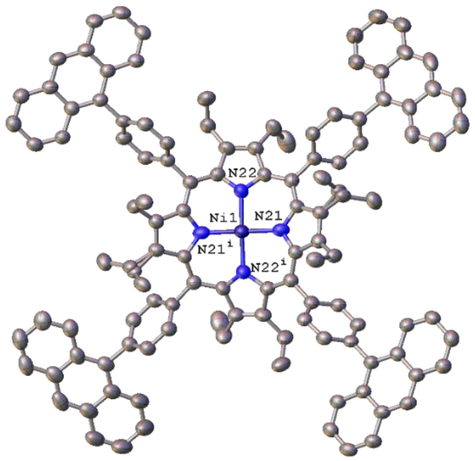

Figure S128: View of the molecular structure of **27** in the crystal, hydrogen atoms omitted for clarity. Disorder in a β-position ethyl is also shown. Atomic displacement shown at 50% probability.

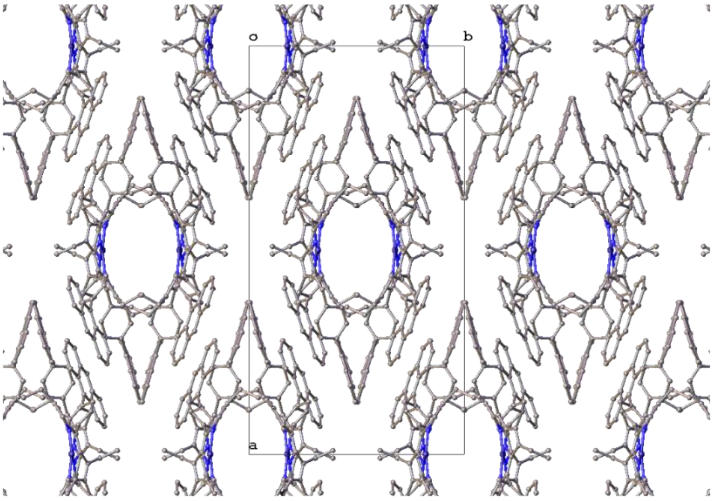

Figure S129: Schematic packing diagram of **27** viewed normal to the c-axis showing the channels in the lattice. Solvent molecule density removed using masking in OLEX2. Hydrogen atoms omitted for clarity.

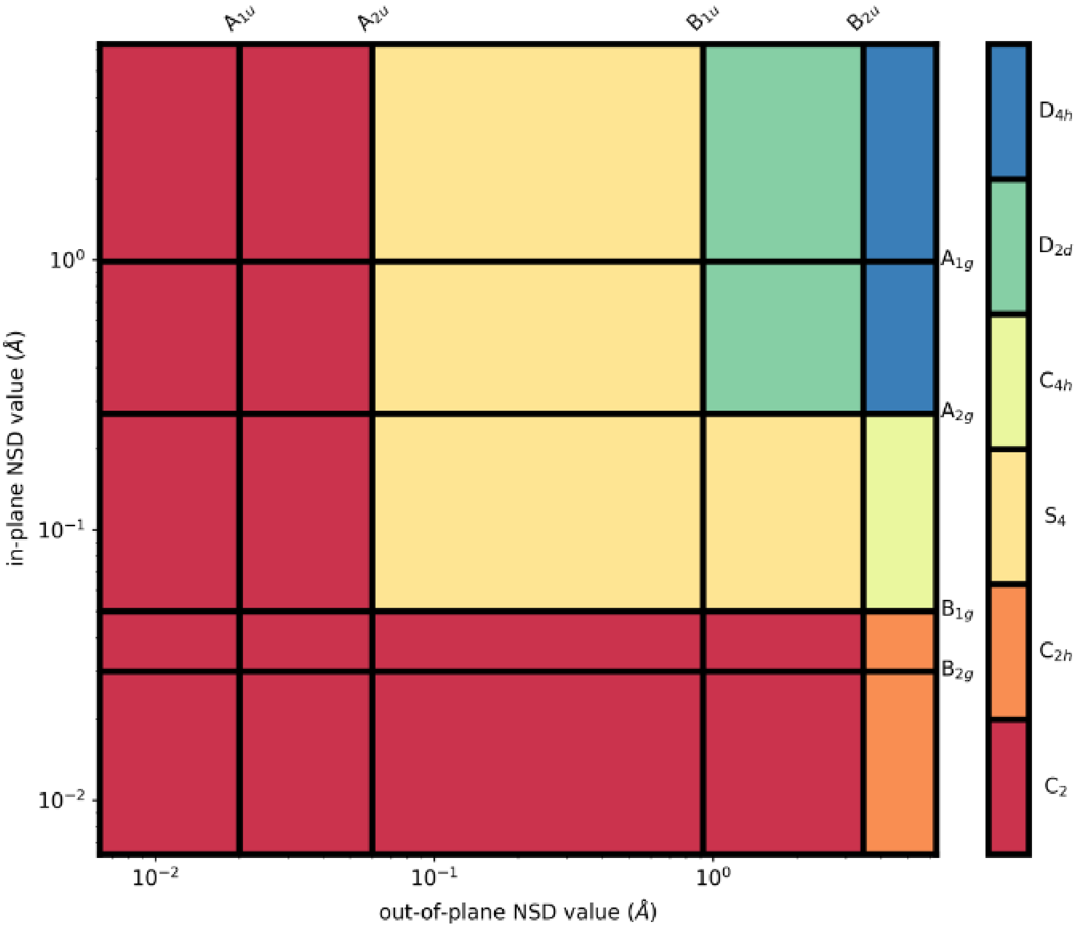

Figure S130: Neoplastic representation of the NSD symmetry elements for porphyrin **27**.

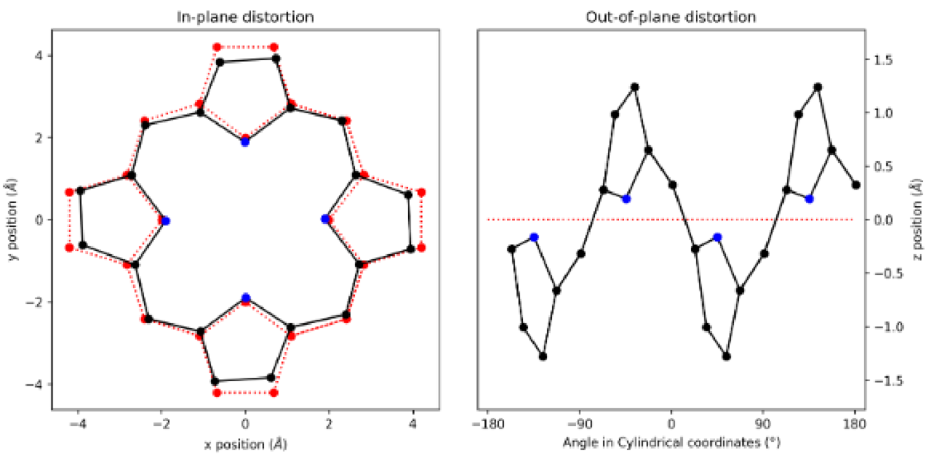

Figure S131: Out-of-plane and in-plane skeletal plots of the porphyrin core. Porphyrin **27** is represented in black (carbon) and blue (nitrogen), with the reference structure CuTPP in red dotted lines.

Table S6. Mean bond distances, angles, and deviations from planarity of porphyrin **27.**

**Bond Distances, Bond Angles, Atom Displacements**	**Mean Value (standard error)**	**Units**
N–C_a	1.376(7)	(Å)
C_a–C_b	1.456(7)	(Å)
C_a–C_m	1.399(4)	(Å)
C_b–C_b	1.356(9)	(Å)
∠$C_aC_bC_b$	106.8(6)	(°)
∠NC_aC_b	109.7(7)	(°)
∠NC_aC_m	122.1(4)	(°)
∠C_aNC_a	106.2(6)	(°)
∠$C_mC_aC_b$	127.5(7)	(°)
∠$C_aC_mC_a$	121.0(4)	(°)
Δ24	0.6141	(Å)
ΔN	0.179(20)	(Å)
ΔC_a	0.47(20)	(Å)
ΔC_b	1.13(14)	(Å)
ΔC_m	0.32(6)	(Å)
∠ pyrrole tilt	28.4(9)	(°)
N···N dist (adj)	2.708(8)	(Å)
N···N dist (opp)	3.795(9)	(Å)

Molecular structure view and NSD analysis of porphyrin 28:

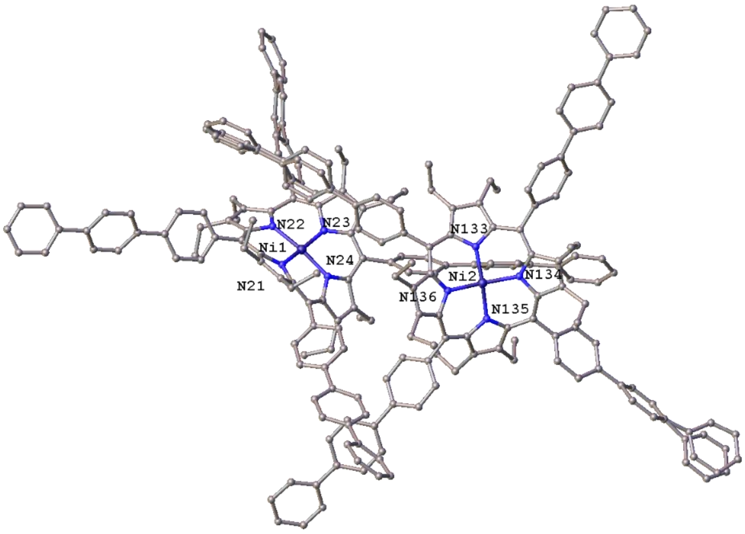

Figure S132: Ball and stick representation of the molecular structure of **28** in the crystal, hydrogen atoms and solvate CH_2Cl_2 omitted for clarity. Disorder in the terphenyl groups on porphyrin shown. Atomic displacement shown at 50% probability.

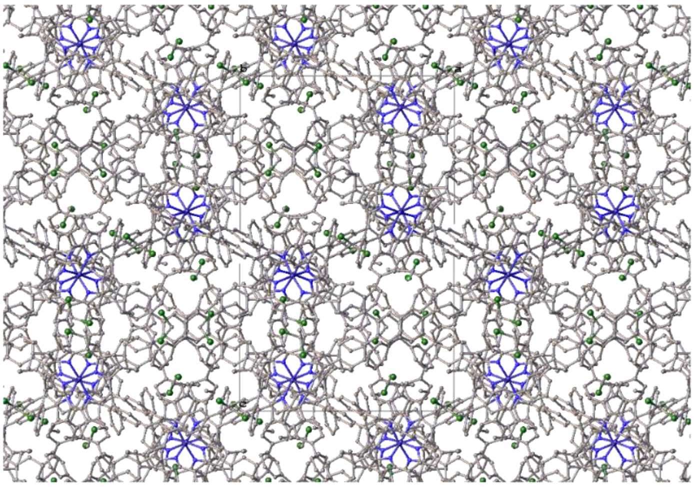

Figure S133: Schematic packing diagram of **28** viewed normal to the b-axis, showing the majority occupied moieties only. Hydrogen atoms omitted for clarity.

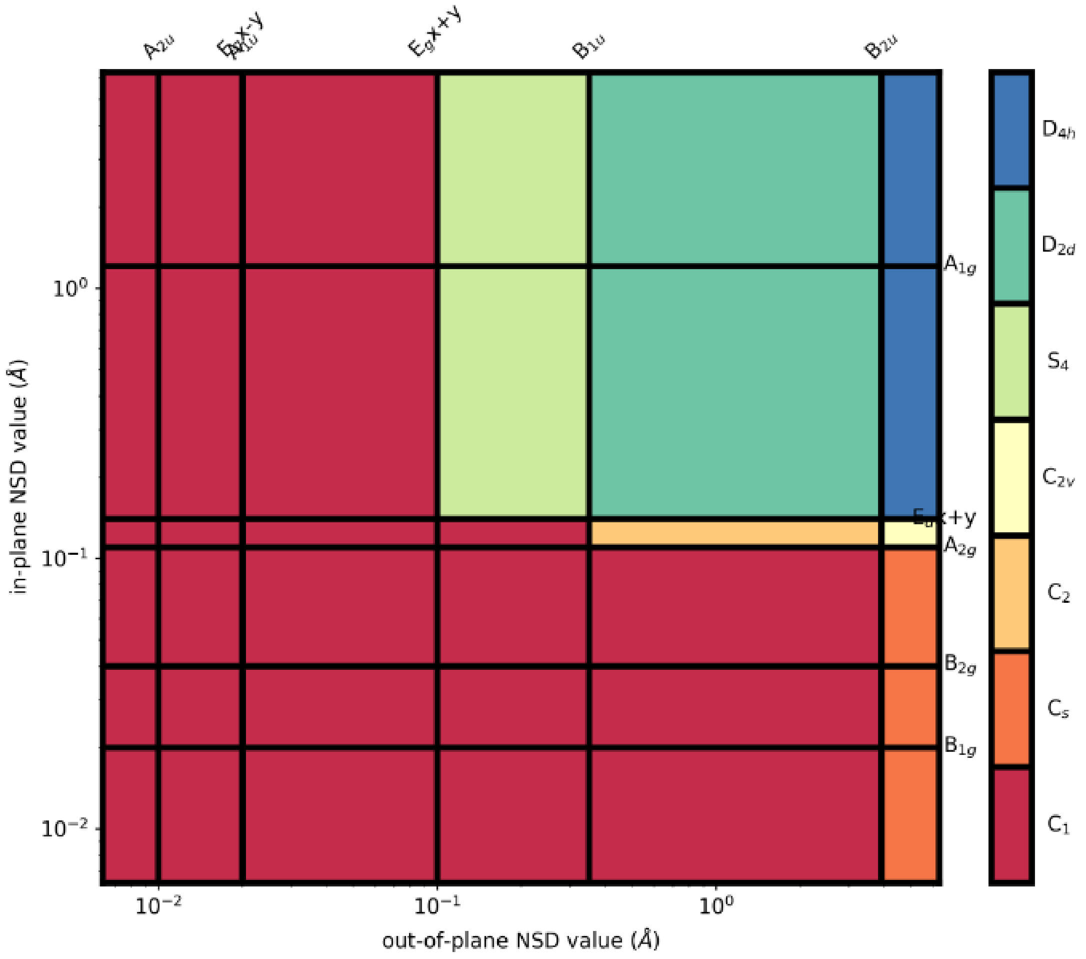

Figure S134: Neoplastic representation of the NSD symmetry elements for porphyrin **28**.

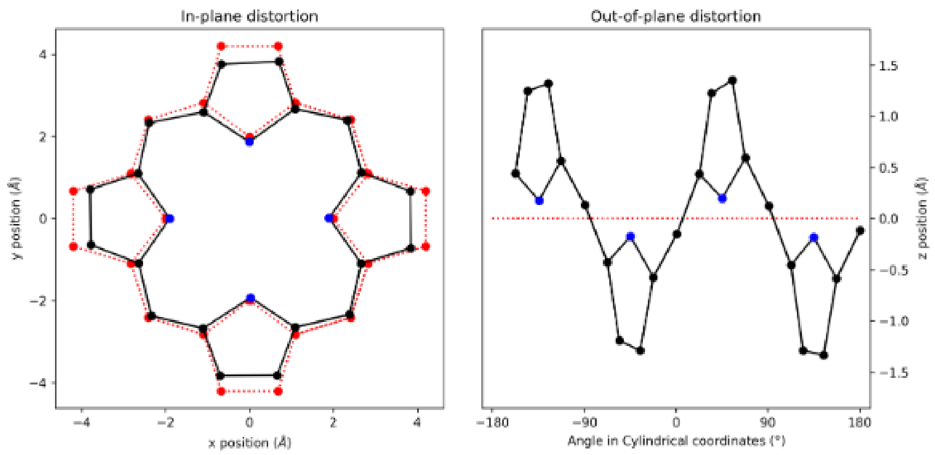

Figure S135: Out-of-plane and in-plane skeletal plots of the porphyrin core. Porphyrin **28** is represented in black (carbon) and blue (nitrogen), with the reference structure CuTPP in red dotted lines.

Table S7. Mean bond distances, angles, and deviations from planarity of porphyrin **28.**

Bond Distances, Bond Angles, Atom Displacements	Mean Value (standard error)	Units
N–C_a	1.372(19)	(Å)
C_a–C_b	1.453(11)	(Å)
C_a–C_m	1.397(9)	(Å)
C_b–C_b	1.369(15)	(Å)
∠$C_aC_bC_b$	106.6(8)	(°)
∠NC_aC_b	109.7(9)	(°)
∠NC_aC_m	121.6(7)	(°)
∠C_aNC_a	106.4(6)	(°)
∠$C_mC_aC_b$	128.3(12)	(°)
∠$C_aC_mC_a$	120.7(13)	(°)
Δ24	0.6485	(Å)
ΔN	0.182(11)	(Å)
$ΔC_a$	0.51(7)	(Å)
$ΔC_b$	1.28(5)	(Å)
$ΔC_m$	0.131(16)	(Å)
∠ pyrrole tilt	31.2(7)	(°)
N···N dist (adj)	2.71(3)	(Å)
N···N dist (opp)	3.802(2)	(Å)

Molecular structure view and NSD analysis of porphyrin 36:

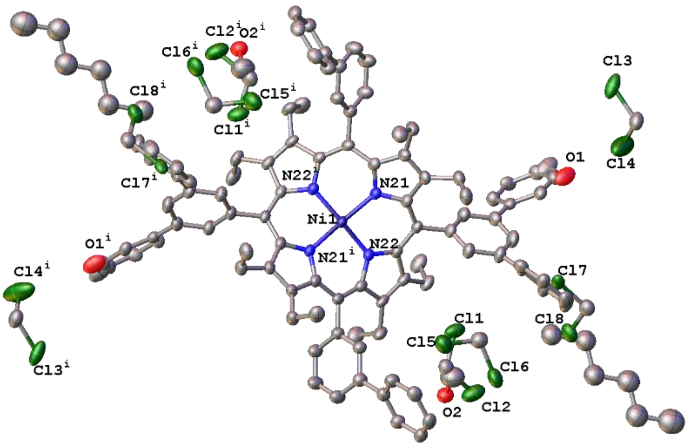

Figure S136: View of the molecular structure of **36** in the crystal, hydrogen atoms omitted for clarity, thermal ellipsoids show 50% probability, symmetry generated over inversion.

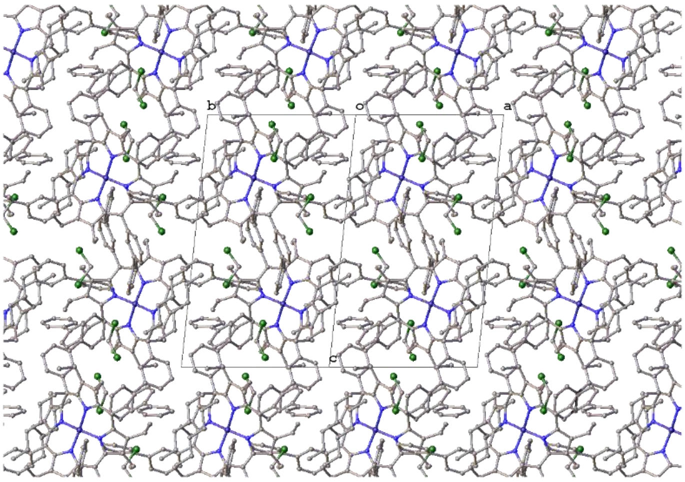

Figure S137: Schematic packing diagram of the majority occupied moieties of **36** viewed normal to the 110 direction.

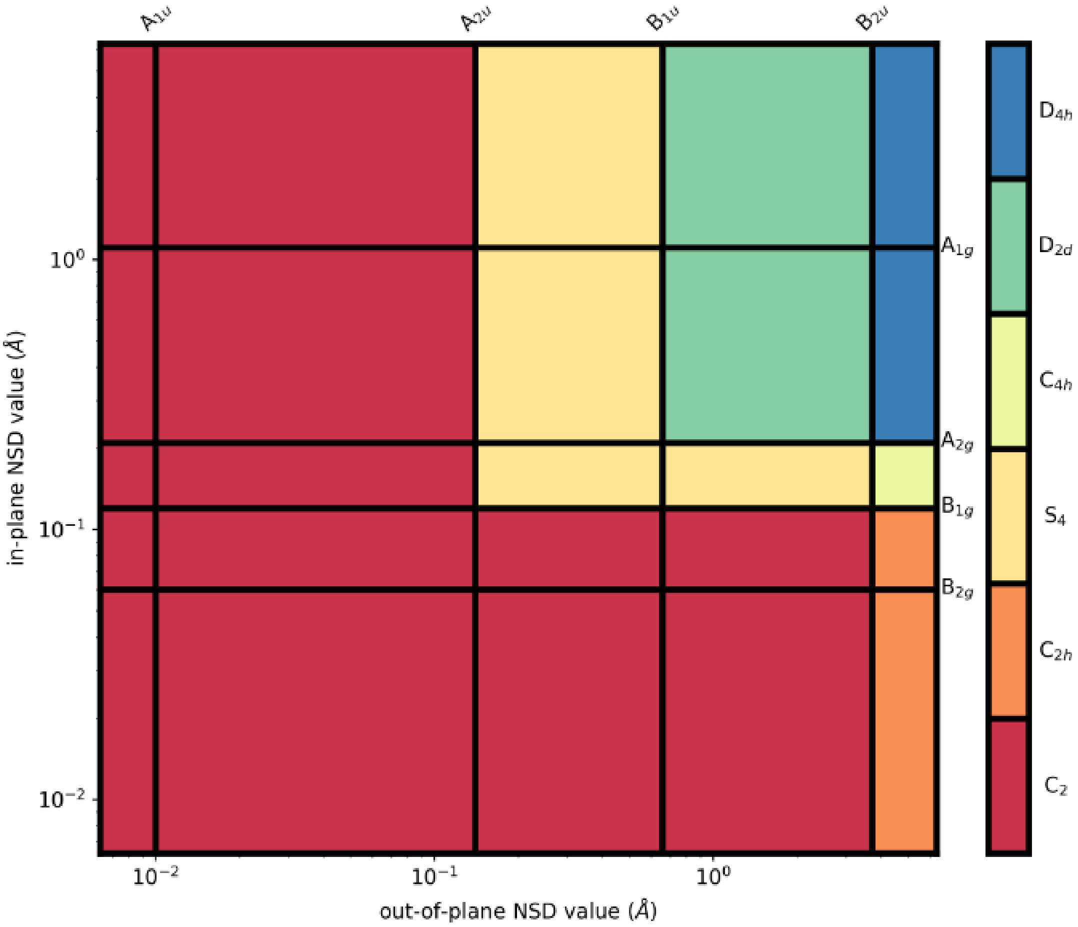

Figure S138: Neoplastic representation of the NSD symmetry elements for porphyrin **36**.

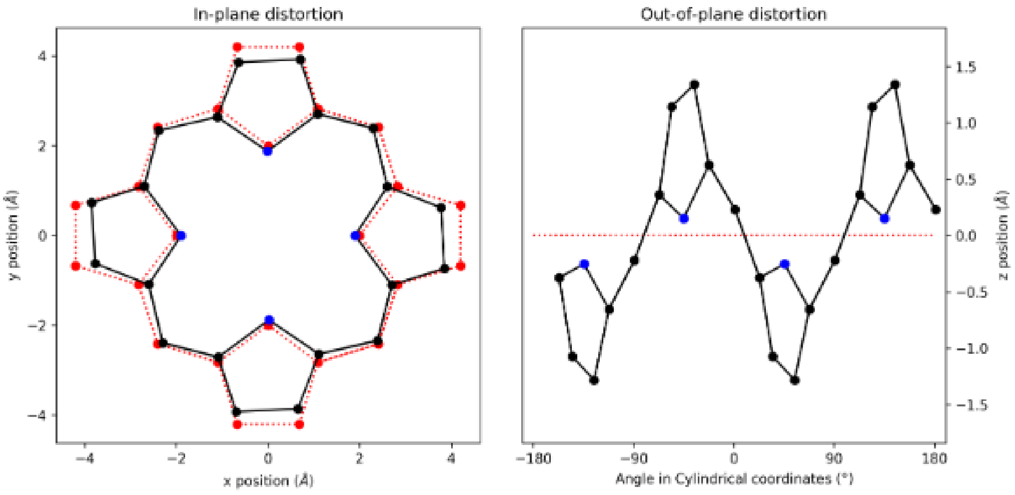

Figure S139: Out-of-plane and in-plane skeletal plots of the porphyrin core. Porphyrin **36** is represented in black (carbon) and blue (nitrogen), with the reference structure CuTPP in red dotted lines.

Table S8: Mean bond distances, angles, and deviations from planarity of porphyrin **36.**

**Bond Distances, Bond Angles, Atom Displacements**	**Mean Value (standard error)**	**Units**
$N–C_a$	1.384(5)	(Å)
$C_a–C_b$	1.447(6)	(Å)
$C_a–C_m$	1.393(7)	(Å)
$C_b–C_b$	1.368(6)	(Å)
$\angle C_aC_bC_b$	107.0(3)	(°)
$\angle NC_aC_b$	109.5(3)	(°)
$\angle NC_aC_m$	121.2(2)	(°)
$\angle C_aNC_a$	106.12(16)	(°)
$\angle C_mC_aC_b$	128.6(6)	(°)
$\angle C_aC_mC_a$	121.95(17)	(°)
Δ24	0.6423	(Å)
ΔN	0.2(5)	(Å)
ΔC_a	0.5(14)	(Å)
ΔC_b	1.21(11)	(Å)
ΔC_m	0.224(5)	(Å)
∠ pyrrole tilt	29(2)	(°)
N···N dist (adj)	2.705(9)	(Å)
N···N dist (opp)	3.782(20)	(Å)

Molecular structure view and NSD analysis of porphyrin 11:

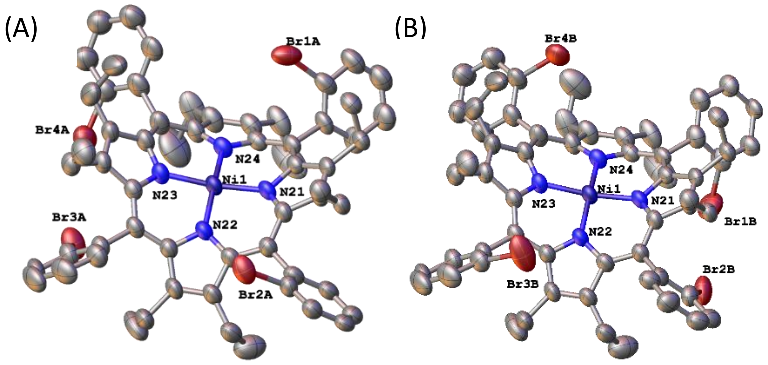

Figure S140: Individual representations of the two majority occupied moieties in **11** with (A) Br1a, 70%, Br2a, 51%, Br3a, 60% and Br4a 36% occupied and (B) Br1b, 30%, Br2b 49%, Br3b, 40% and Br4b, 37% occupied. Displacement parameters shown at 50% probability and heteroatoms labelled only. Hydrogen atoms omitted for clarity.

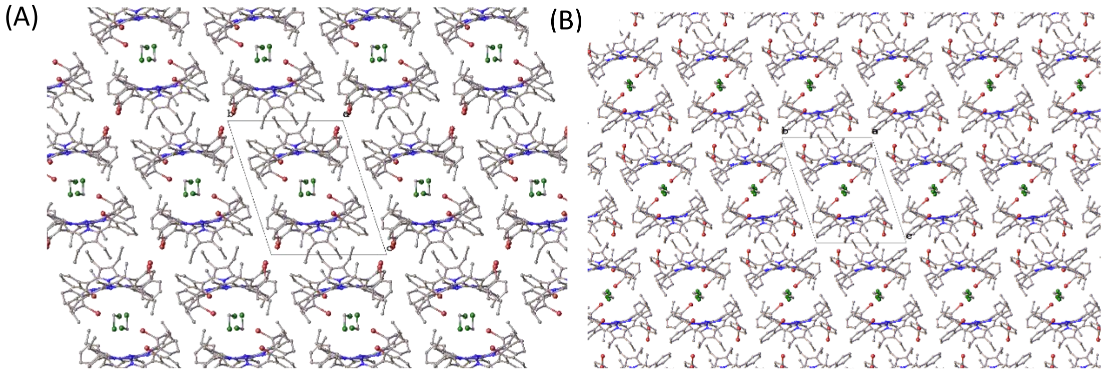

Figure S141: Schematic packing diagrams of porphyrin **11**, with majority (A) and minority (B) occupied moieties, viewed normal to the a-axis. Hydrogen atoms omitted for clarity.

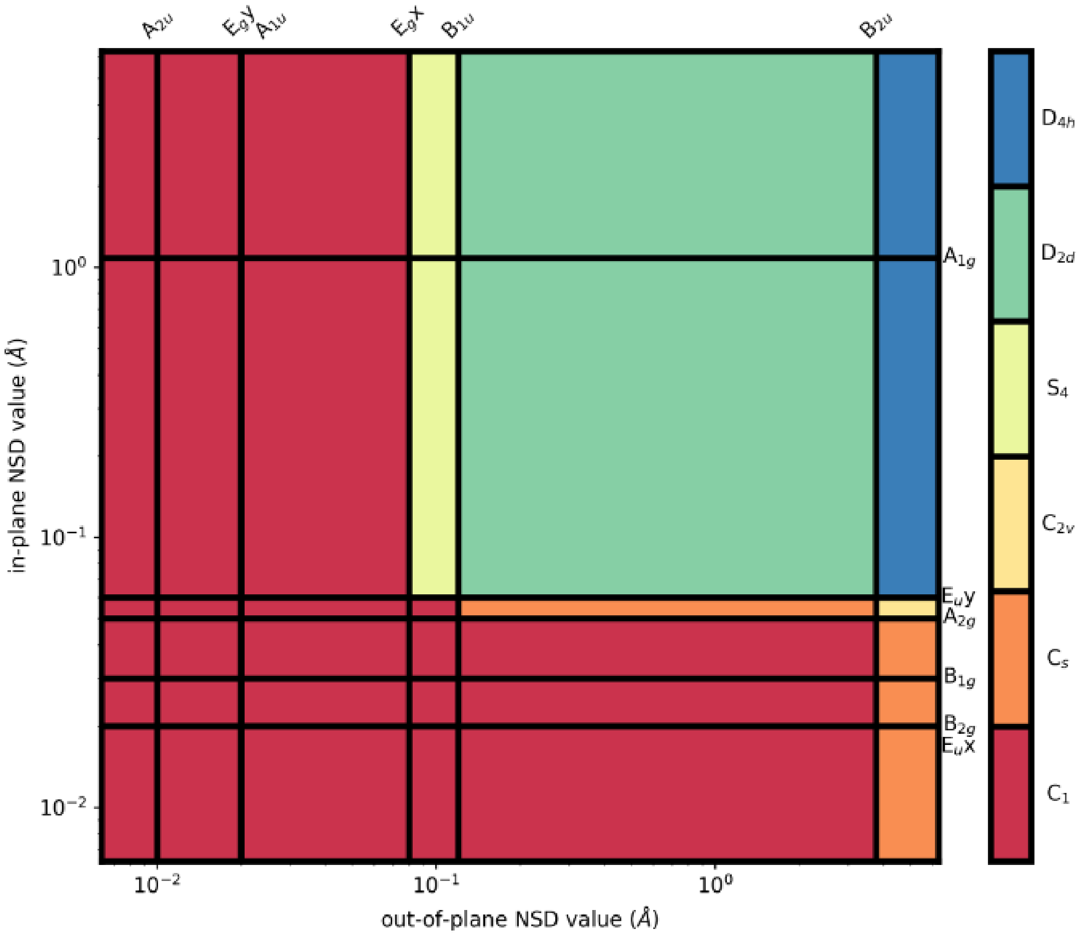

Figure S142: Neoplastic representation of the NSD symmetry elements for porphyrin **11**.

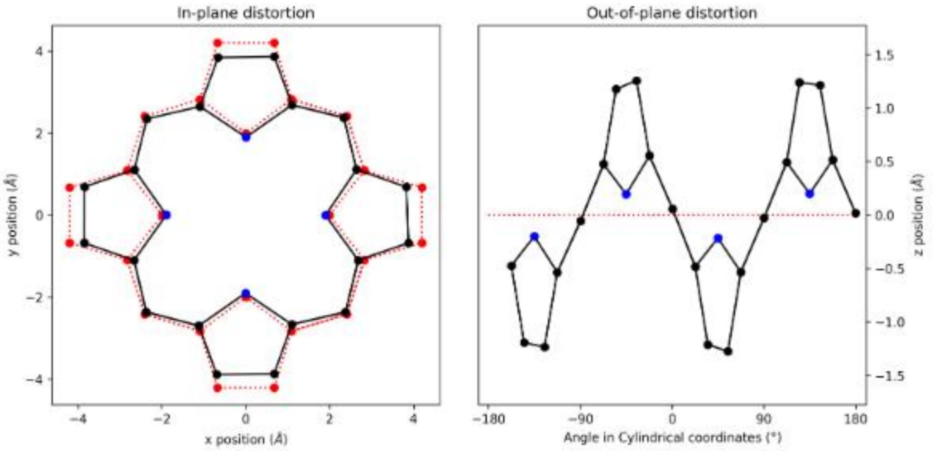

Figure S143: Out-of-plane and in-plane skeletal plots of the porphyrin core. Porphyrin **11** is represented in black (carbon) and blue (nitrogen), with the reference structure CuTPP in red dotted lines.

Table S9. Mean bond distances, angles, and deviations from planarity of porphyrin **11.**

Bond Distances, Bond Angles, Atom Displacements	Mean Value (standard error)	Units
N–C_a	1.38(10)	(Å)
C_a–C_b	1.454(9)	(Å)
C_a–C_m	1.391(9)	(Å)
C_b–C_b	1.362(12)	(Å)
∠$C_aC_bC_b$	106.9(5)	(°)
∠NC_aC_b	109.4(4)	(°)
∠NC_aC_m	121.3(5)	(°)
∠C_aNC_a	106.3(3)	(°)
∠$C_mC_aC_b$	128.6(8)	(°)
∠$C_aC_mC_a$	122.1(5)	(°)
Δ24	0.6178	(Å)
ΔN	0.202(8)	(Å)
ΔC_a	0.51(3)	(Å)
ΔC_b	1.22(3)	(Å)
ΔC_m	0.039(16)	(Å)
∠ pyrrole tilt	28.6(3)	(°)
N···N dist (adj)	2.717(3)	(Å)
N···N dist (opp)	3.8(2)	(Å)

Molecular structure view and NSD analysis of porphyrin 33:

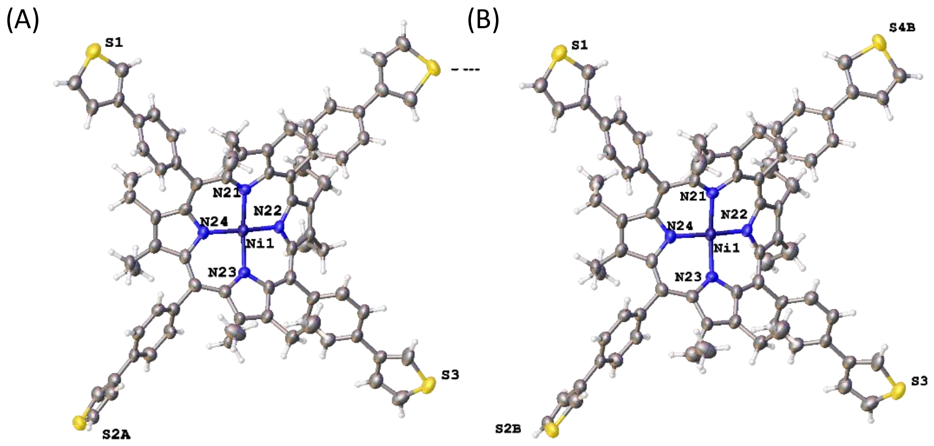

Figure S144: Individual representation of each disordered moiety in **33** with (A) majority occupied moiety with C53/C54 and C68/C69 62% and 76% occupied and (B) minority occupied moiety with C53/C54 and C68/C69 38% and 24% occupied. Displacement parameters shown at 50% probability and heteroatoms labelled only.

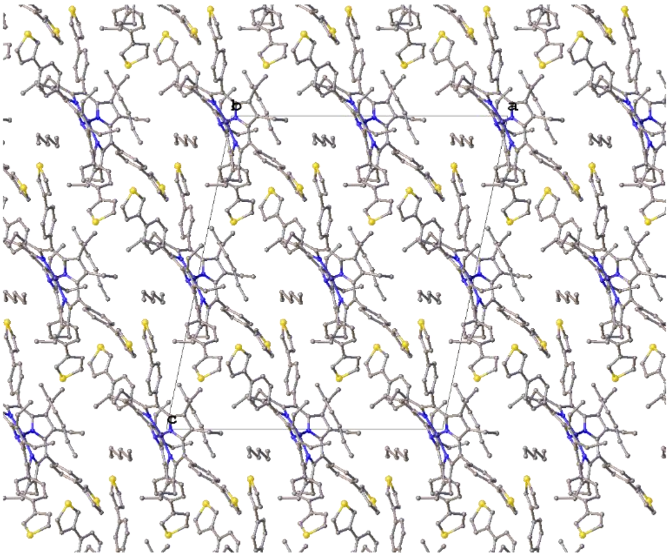

Figure S145: Schematic packing diagram of majority occupied moiety in **33**, viewed normal to the b-axis. Hydrogen atoms omitted for clarity.

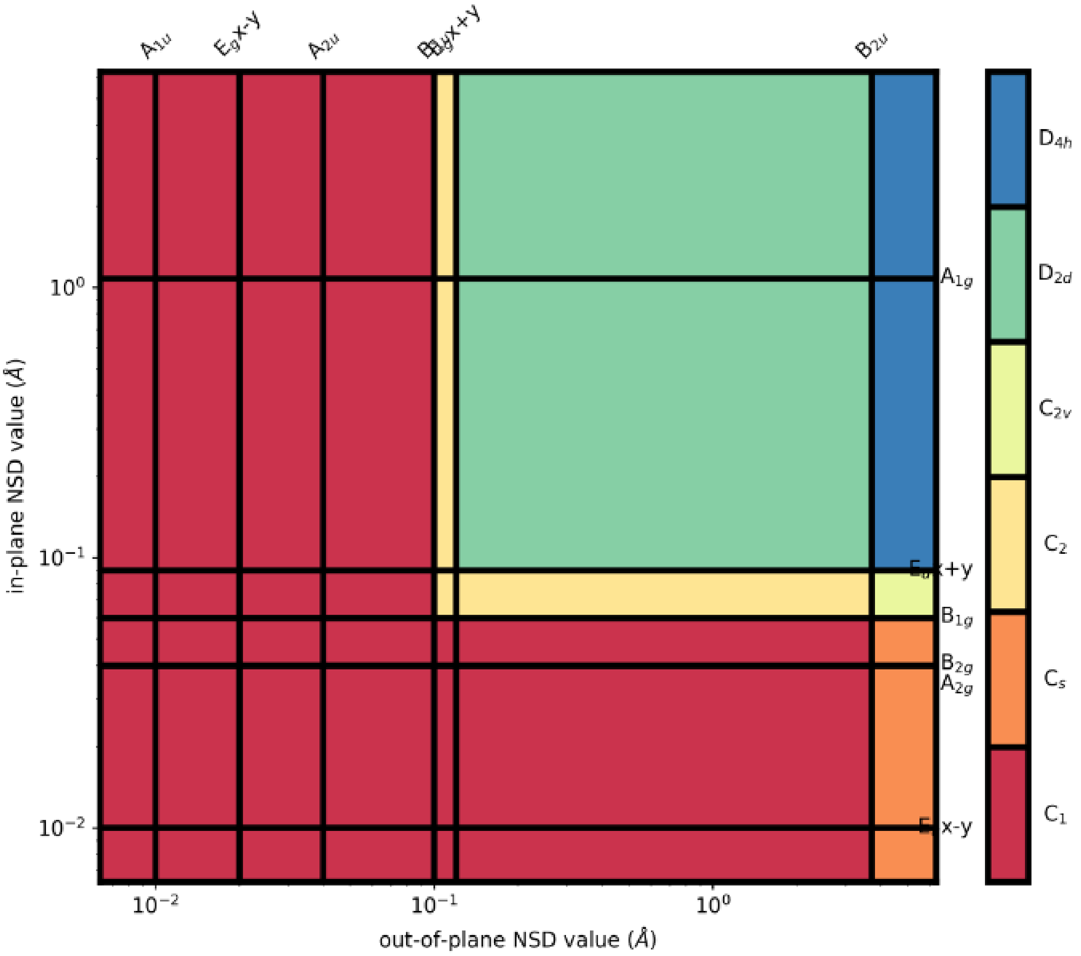

Figure S146: Neoplastic representation of the NSD symmetry elements for porphyrin **33**.

Table S10: Mean bond distances, angles, and deviations from planarity of porphyrin **33.**

**Bond Distances, Bond Angles, Atom Displacements**	**Mean Value (standard error)**	**Units**
N–C_a	1.37(2)	(Å)
C_a–C_b	1.444(16)	(Å)
C_a–C_m	1.396(17)	(Å)
C_b–C_b	1.365(17)	(Å)
∠$C_aC_bC_b$	106.8(8)	(°)
∠NC_aC_b	109.5(7)	(°)
∠NC_aC_m	121.6(5)	(°)
∠C_aNC_a	106.5(3)	(°)
∠$C_mC_aC_b$	128.3(8)	(°)
∠$C_aC_mC_a$	121.6(9)	(°)
Δ24	0.6056	(Å)
ΔN	0.178(19)	(Å)
ΔC_a	0.5(2)	(Å)
ΔC_b	1.21(3)	(Å)
ΔC_m	0.03(2)	(Å)
∠ pyrrole tilt	29.0(12)	(°)
N···N dist (adj)	2.732(10)	(Å)
N···N dist (opp)	3.83(2)	(Å)

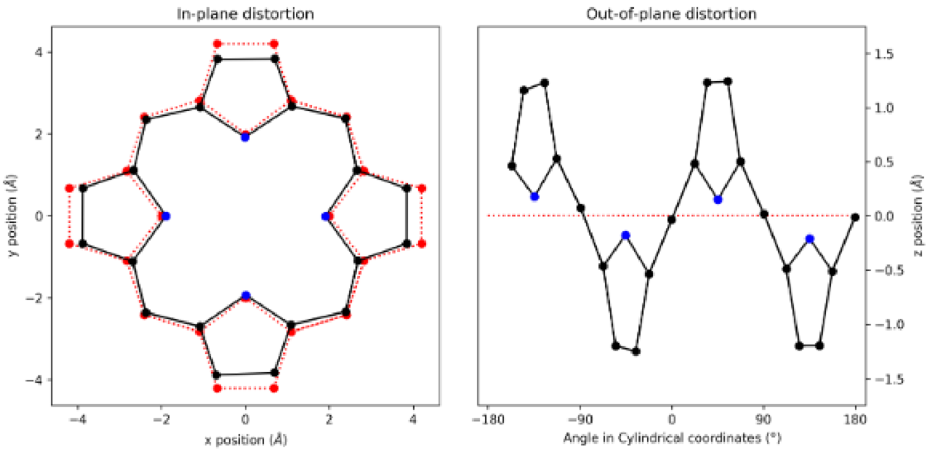

Figure S147: Out-of-plane and in-plane skeletal plots of the porphyrin core. Porphyrin **33** is represented in black (carbon) and blue (nitrogen), with the reference structure CuTPP in red dotted lines.

Molecular structure view and NSD analysis of porphyrin 46:

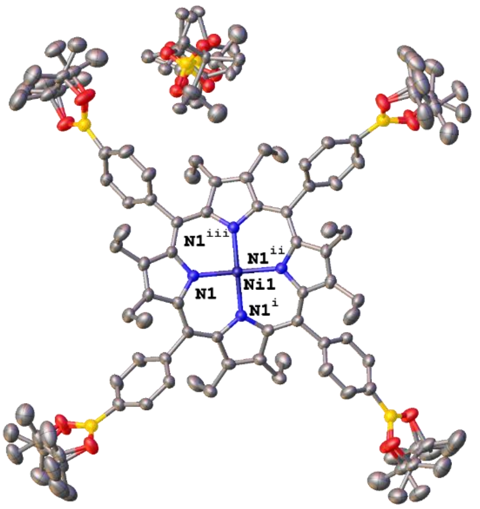

Figure S148: View of the molecular structure of **46** in the crystal. Displacement parameters shown at 50% probability and heteroatoms labelled only. Hydrogen atoms omitted for clarity. Symmetry transformations: i = +Y, 3/2-X, 3/2-Z; ii = 3/2-X, 3/2-Y, +Z; iii = 3/2-Y,+X,3/2-Z.

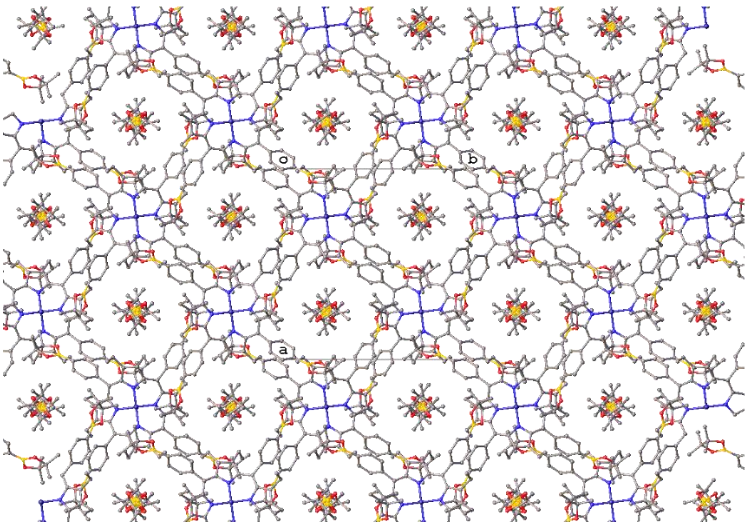

Figure S149: Schematic packing diagram of majority occupied moiety in **46**, viewed normal to the c-axis. Hydrogen atoms omitted for clarity.

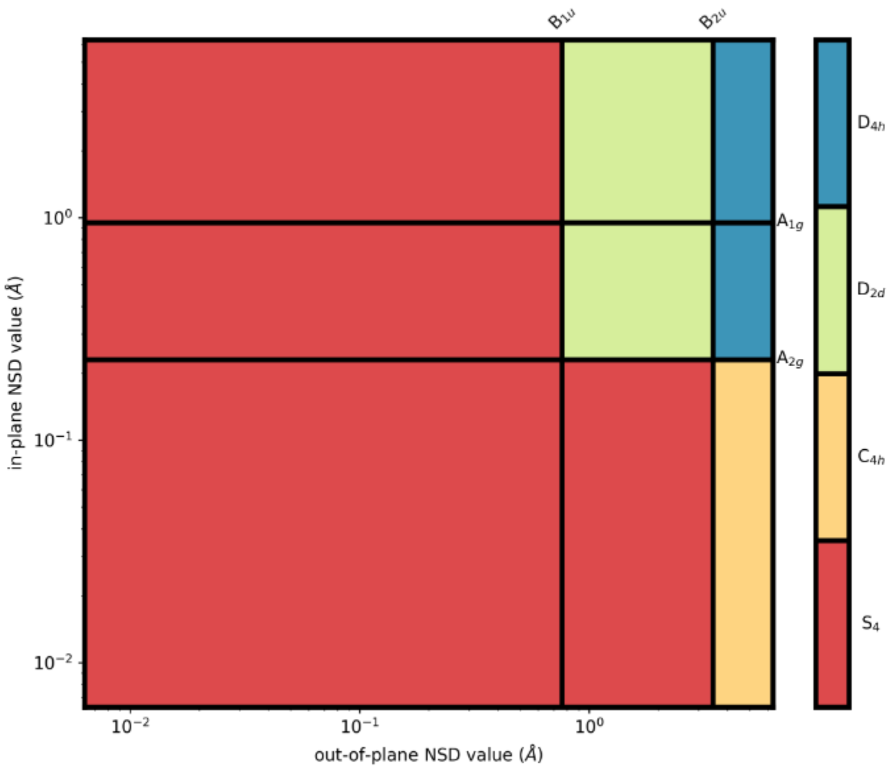

Figure S150: Neoplastic representation of the NSD symmetry elements for porphyrin **46**.

Table S11: Mean bond distances, angles, and deviations from planarity of porphyrin **46.**

Bond Distances, Bond Angles, Atom Displacements	Mean Value (standard error)	Units
N–C_a	1.384(2)	(Å)
C_a–C_b	1.4541(19)	(Å)
C_a–C_m	1.3912(17)	(Å)
C_b–C_b	1.3594(5)	(Å)
∠$C_aC_bC_b$	106.99(9)	(°)
∠NC_aC_b	109.59(20)	(°)
∠NC_aC_m	121.52(19)	(°)
∠C_aNC_a	105.982(4)	(°)
∠$C_mC_aC_b$	128.1(6)	(°)
∠$C_aC_mC_a$	122.49(4)	(°)
Δ24	0.608	(Å)
ΔN	0.2005(17)	(Å)
ΔC_a	0.48(16)	(Å)
ΔC_b	1.12(12)	(Å)
ΔC_m	0.258(3)	(Å)
∠ pyrrole tilt	27.027(2)	(°)
N⋯N dist (adj)	2.71	(Å)
N⋯N dist (opp)	3.79	(Å)

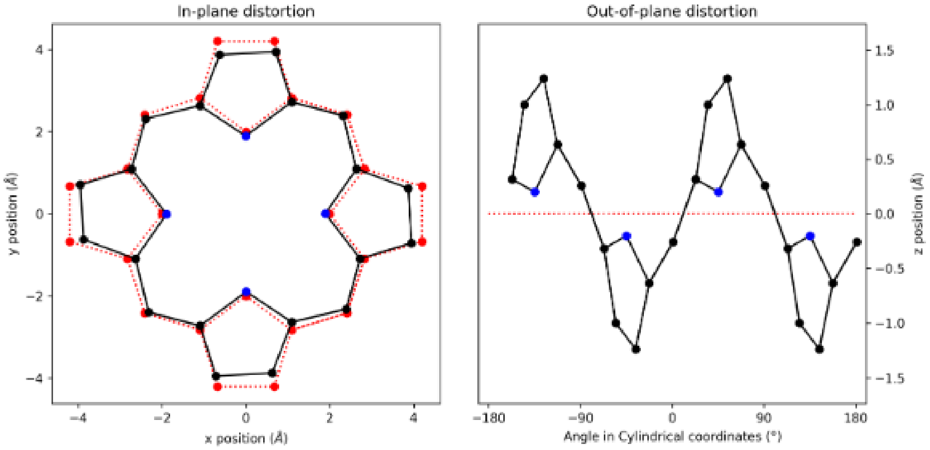

Figure S15: Out-of-plane and in-plane skeletal plots of the porphyrin core. Porphyrin **47** is represented in black (carbon) and blue (nitrogen), with the reference structure CuTPP in red dotted lines.

Molecular structure view and NSD analysis of porphyrin 29:

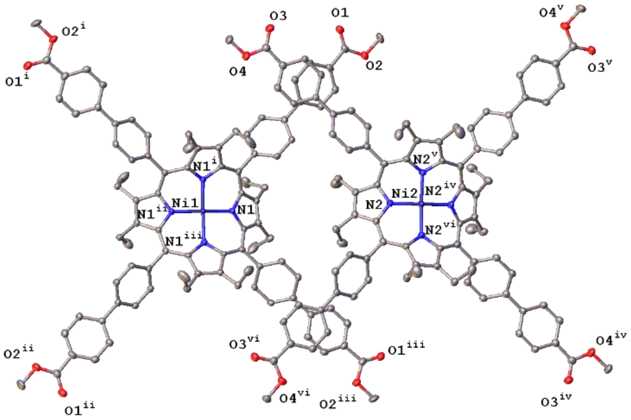

Figure S152: Symmetry generated molecular structure of **29** in the crystal disordered solvent and hydrogen atoms omitted for clarity. Displacement parameters shown at 50% probability and heteroatoms labelled only. Symmetry transformations i = -Y, +X, -Z; ii = -X, -Y, +Z; iii = +Y, -X, -Z; iv = 1-X, -Y, +Z; v = 1/2+Y, 1/2-X, 1/2-Z; vi = 1/2-Y, -1/2+X, 1/2-Z.

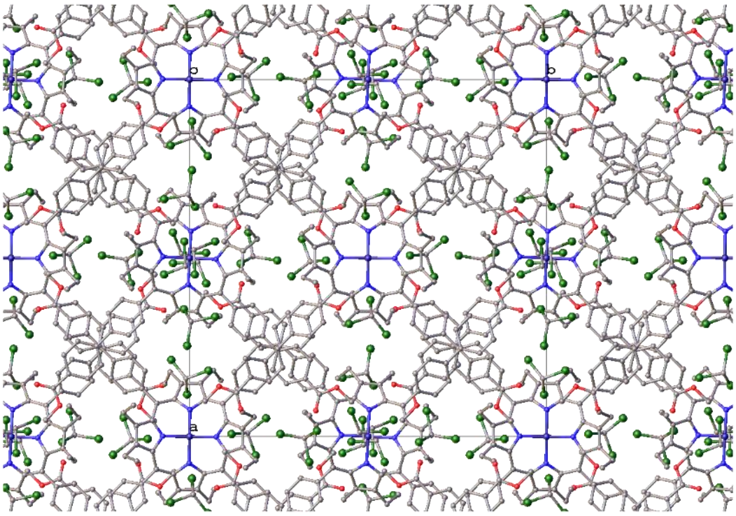

Figure S153: Schematic packing diagram of the majority occupied moiety in **29**, viewed normal to the c-axis.

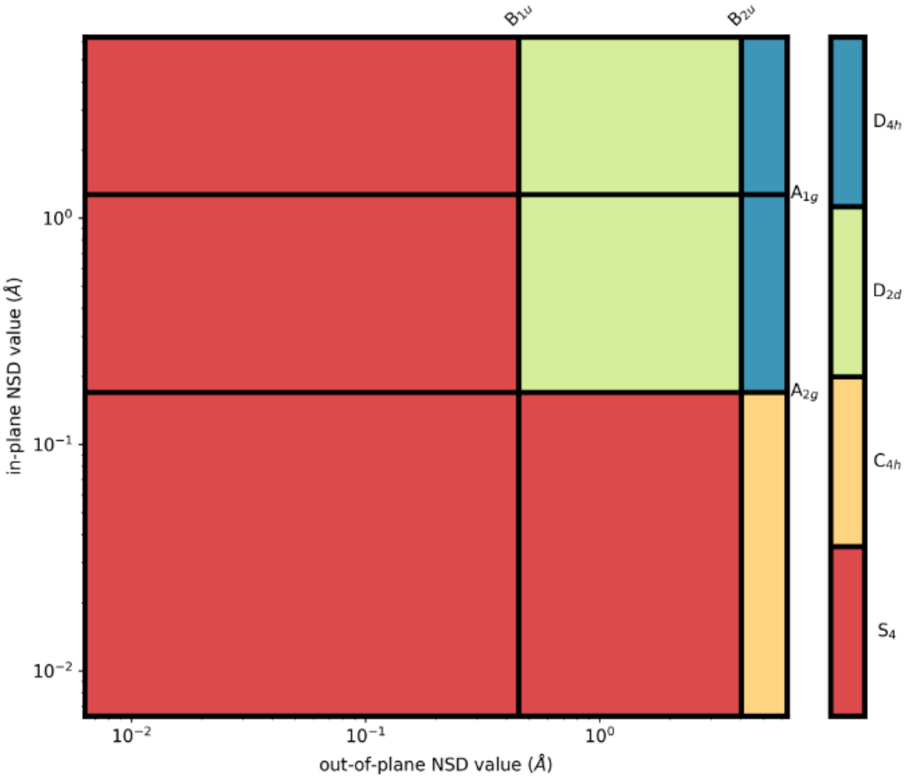

Figure S154: Neoplastic representation of the NSD symmetry elements for porphyrin **29**.

Table S12: Mean bond distances, angles, and deviations from planarity of porphyrin **29.**

Bond Distances, Bond Angles, Atom Displacements	Mean Value (standard error)	Units
N–C_a	1.377(5)	(Å)
C_a–C_b	1.452(5)	(Å)
C_a–C_m	1.396(3)	(Å)
C_b–C_b	1.3612	(Å)
∠$C_aC_bC_b$	107.0(3)	(°)
∠NC_aC_b	109.12(15)	(°)
∠NC_aC_m	120.86(17)	(°)
∠C_aNC_a	106.72	(°)
∠$C_mC_aC_b$	129.48(9)	(°)
∠$C_aC_mC_a$	121.4	(°)
Δ24	0.6712	(Å)
ΔN	0.179	(Å)
$ΔC_a$	0.53(10)	(Å)
$ΔC_b$	1.32(7)	(Å)
$ΔC_m$	0.151	(Å)
∠ pyrrole tilt	32.85	(°)
N···N dist (adj)	2.72	(Å)
N···N dist (opp)	3.81	(Å)

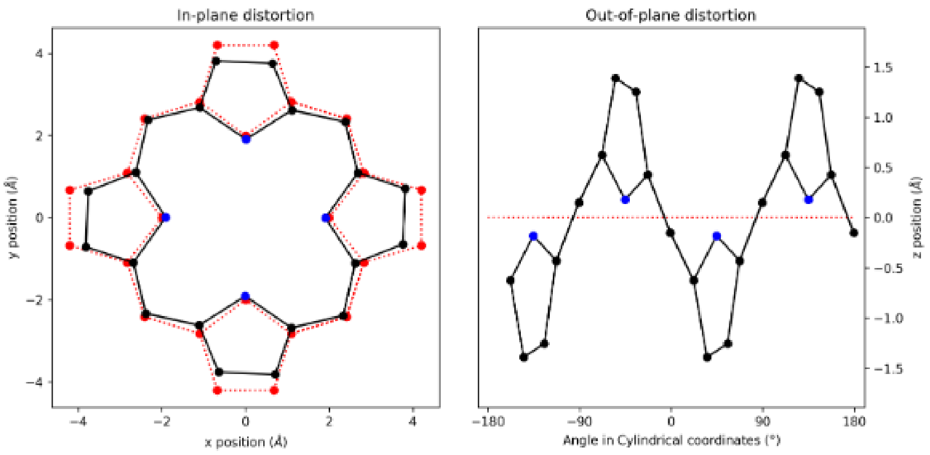

Figure S155: Out-of-plane and in-plane skeletal plots of the porphyrin core. Porphyrin **29** is represented in black (carbon) and blue (nitrogen), with the reference structure CuTPP in red dotted lines.

Molecular structure view and NSD analysis of porphyrin 37:

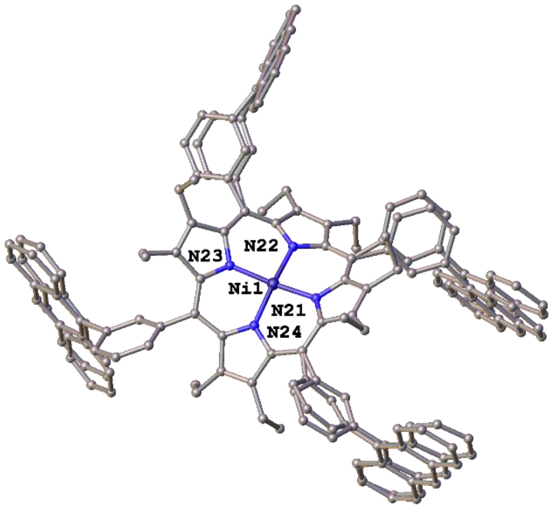

Figure S156: Ball and stick representation of **37** showing the disorder in substituents with only the heteroatoms labelled. Hydrogen atom omitted for clarity.

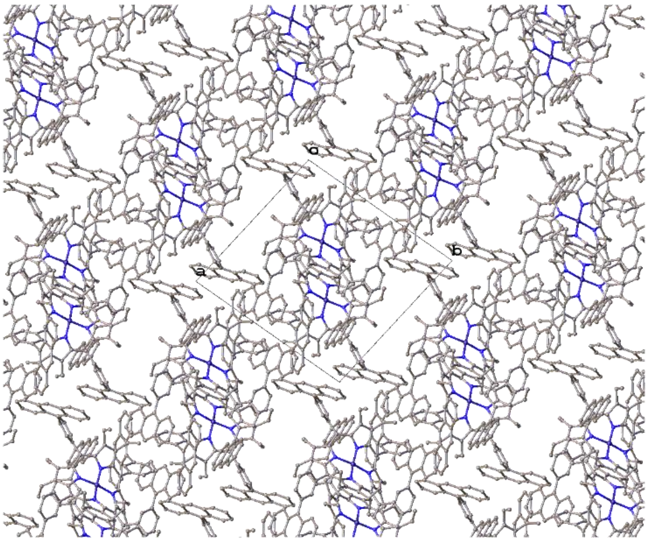

Figure S157: Schematic packing diagram of majority occupied moiety in **37**, viewed normal to the c-axis. Hydrogen atoms omitted for clarity.

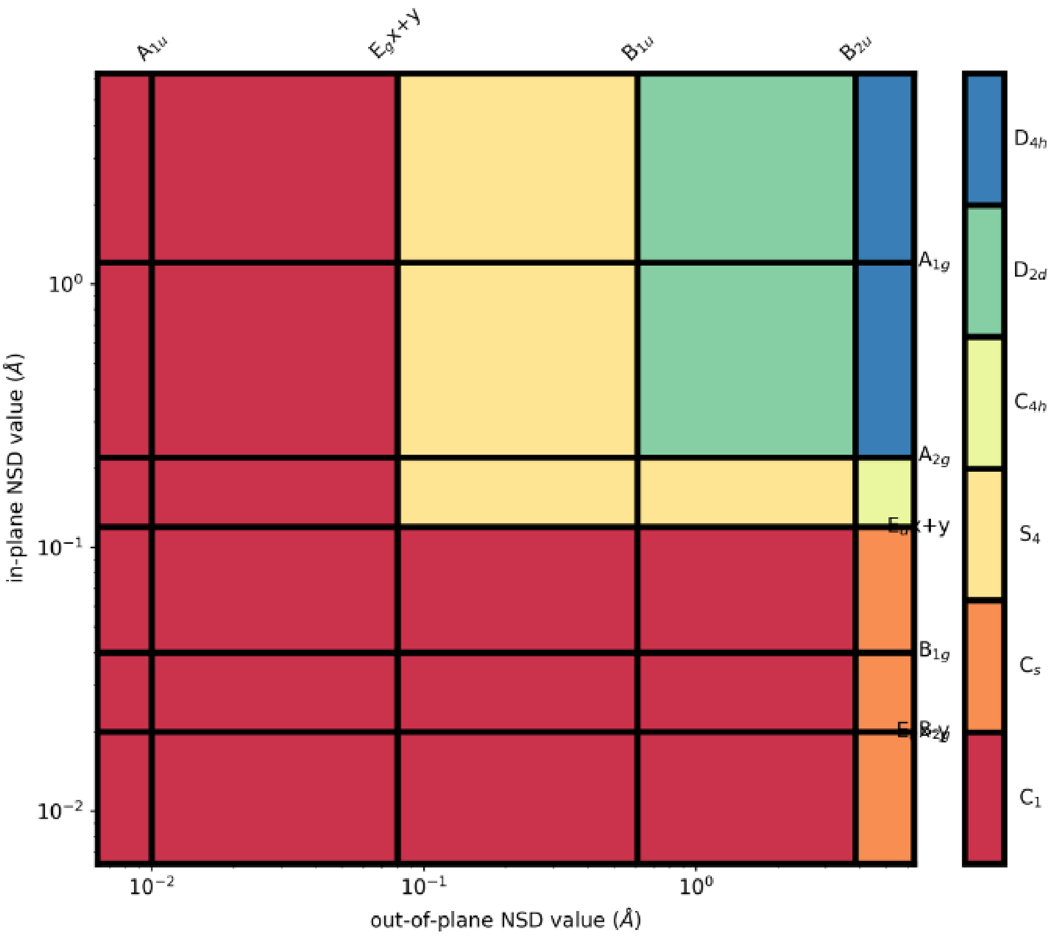

Figure S158: Neoplastic representation of the NSD symmetry elements for porphyrin **37**.

Table S13: Mean bond distances, angles, and deviations from planarity of porphyrin **37.**

Bond Distances, Bond Angles, Atom Displacements	Mean Value (standard error)	Units
N–C_a	1.373(9)	(Å)
C_a–C_b	1.451(17)	(Å)
C_a–C_m	1.398(18)	(Å)
C_b–C_b	1.37(2)	(Å)
∠$C_aC_bC_b$	106.7(10)	(°)
∠NC_aC_b	109.5(7)	(°)
∠NC_aC_m	121.6(11)	(°)
∠C_aNC_a	106.7(9)	(°)
∠$C_mC_aC_b$	128.2(14)	(°)
∠$C_aC_mC_a$	120.5(13)	(°)
Δ24	0.6578	(Å)
ΔN	0.183(11)	(Å)
ΔC_a	0.51(13)	(Å)
ΔC_b	1.26(9)	(Å)
ΔC_m	0.217(14)	(Å)
∠ pyrrole tilt	31.1(4)	(°)
N···N dist (adj)	2.708(8)	(Å)
N···N dist (opp)	3.79(3)	(Å)

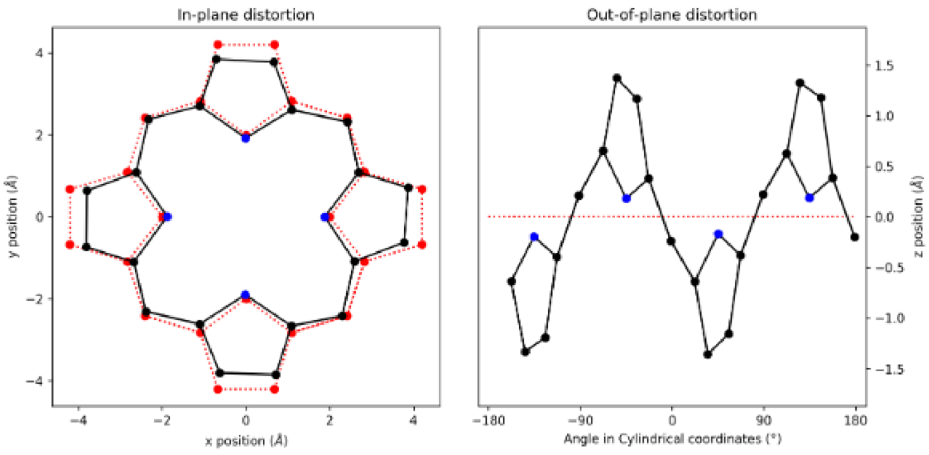

Figure S159: Mean bond distances, angles, and deviations from planarity of porphyrin **37.**

References

1. Bruker AXS Inc., M. Bruker APEX2, SADABS, XPREP and SAINT-Plus. **2004**.

2. Krause, L.; Herbst-Irmer, R.; Sheldrick, G. M.; Stalke, D. *J. Appl. Crystallogr.* **2015**, *48*, 3–10.

3. Sheldrick, G. *Acta Crystallogr., Sect. A: Struct. Chem.* **2015**, *71*, 3–8.

4. Sheldrick, G. *Acta Crystallogr., Sect. C: Struct. Chem.* **2015**, *71*, 3–8.

5. Dolomanov, O. V.; Bourhis, L. J.; Gildea, R. J.; Howard, J. A. K.; Puschmann, H. *J. Appl. Crystallogr.* **2009**, *42*, 339–341.

6. Kingsbury, C. J.; Senge, M. O. *Angew. Chem. Int. Ed.* **2024**, *63*, e202403754.

7. Groom, C. R.; Bruno, I. J.; Lightfoot, M. P.; Ward, S. C. *Acta Crystallogr., Sect. B: Struct. Chem.* **2016**, *72*, 171–179.

8. Kratzert, D. *FinalCif*. https://dkratzert.de/finalcif.html.
